# What Drives Radical Halogenation versus Hydroxylation in Mononuclear Nonheme Iron Complexes? A Combined Experimental and Computational Study

**DOI:** 10.1021/jacs.2c01375

**Published:** 2022-05-10

**Authors:** Emilie F. Gérard, Vishal Yadav, David P. Goldberg, Sam P. de Visser

**Affiliations:** † Manchester Institute of Biotechnology, The University of Manchester, 131 Princess Street, Manchester M1 7DN, United Kingdom; ‡ Department of Chemical Engineering, The University of Manchester, Oxford Road, Manchester M13 9PL, United Kingdom; § Department of Chemistry, The Johns Hopkins University, 3400 North Charles Street, Baltimore, Maryland 21218, United States

## Abstract

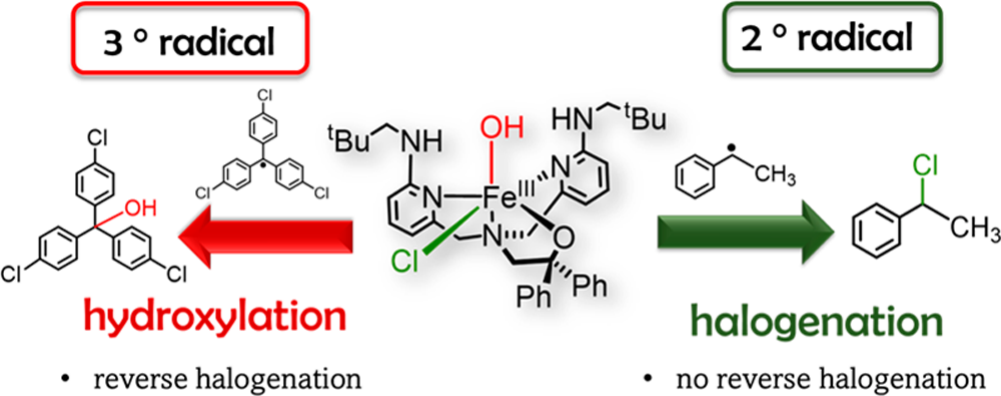

Nonheme iron halogenases are unique enzymes in nature that selectively activate an aliphatic C–H bond of a substrate to convert it into C–X (X = Cl/Br, but not F/I). It is proposed that they generate an Fe^III^(OH)(X) intermediate in their catalytic cycle. The analogous Fe^III^(OH) intermediate in nonheme iron hydroxylases transfers OH^•^ to give alcohol product, whereas the halogenases transfer X^•^ to the carbon radical substrate. There remains significant debate regarding what factors control their remarkable selectivity of the halogenases. The reactivity of the complexes Fe^III^(BNPA^Ph2^O)(OH)(X) (X = Cl, Br) with a secondary carbon radical (R^•^) is described. It is found that X^•^ transfer occurs with a secondary carbon radical, as opposed to OH^•^ transfer with tertiary radicals. Comprehensive computational studies involving density functional theory were carried out to examine the possible origins of this selectivity. The calculations reproduce the experimental findings, which indicate that halogen transfer is not observed for the tertiary radicals because of a nonproductive equilibrium that results from the endergonic nature of these reactions, despite a potentially lower reaction barrier for the halogenation pathway. In contrast, halogen transfer is favored for secondary carbon radicals, for which the halogenated product complex is thermodynamically more stable than the reactant complex. These results are rationalized by considering the relative strengths of the C–X bonds that are formed for tertiary versus secondary carbon centers. The computational analysis also shows that the reaction barrier for halogen transfer is significantly dependent on secondary coordination sphere effects, including steric and H-bonding interactions.

## Introduction

The formation and synthesis of carbon–halogen bonds are a relatively rare reaction in nature, yet a number of enzymes catalyze this type of transformation, including halogenases and haloperoxidases, and several of those have been identified in recent years.^1−8^ These enzymes are widespread in marine organisms, fungi, plants, and bacteria and are involved in the biosynthesis of natural products that function as deterrents, pesticides, irritants, and for food gathering.^9,10^ Several antibiotics have been found that contain organohalogen compounds, including vancomycin, which is a glycopeptide with the chlorine atom bound to an aromatic ring.^11^ One of the first haloperoxidases structurally and functionally characterized was chloroperoxidase, a heme peroxidase in fungi that binds H_2_O_2_ and forms an iron(IV)–oxo heme cation radical intermediate, called Compound I (CpdI), during its catalytic cycle.^12−15^ CpdI reacts with halides (X = Cl^–^, Br^–^, I^–^) to form the corresponding hypohalide (OX^–^), which then reacts with a substrate to form the organohalogen product. The biosynthesis of hypohalide from H_2_O_2_ and halide also is catalyzed by the vanadium haloperoxidases and flavin haloperoxidases, but they have a different cofactor and their hypohalide biosynthesis mechanisms are different.^16−22^

**Scheme 1:**
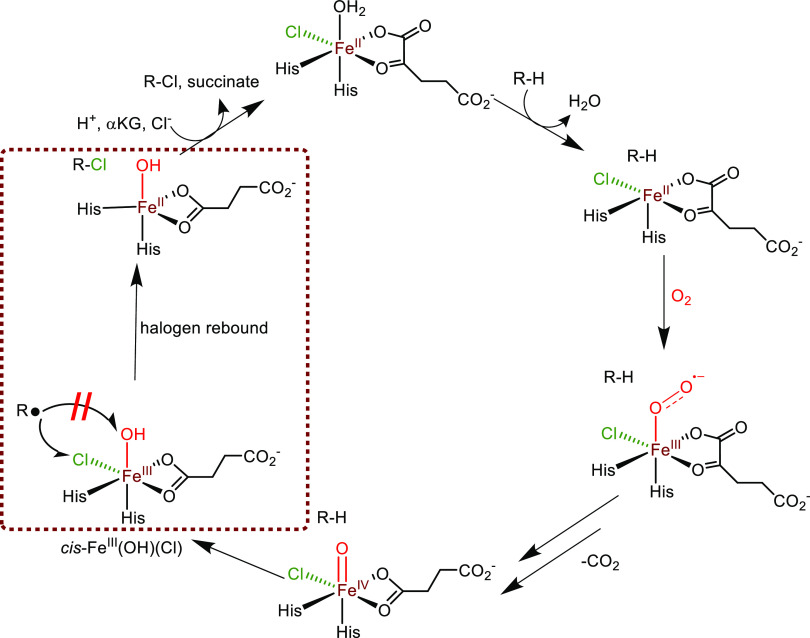
General Reaction Catalyzed by Nonheme Iron Halogenases, with αKG = α-Ketoglutarate and R–H as the Substrate

The α-ketoglutarate-dependent nonheme iron halogenases are fascinating enzymes that activate an aliphatic C–H bond of a substrate selectively using molecular oxygen and an α-ketoglutarate (αKG) cosubstrate on an iron center.^23−29^ In many of these enzymes, the overall reaction is highly stereo- and regiospecific, processes that are still poorly understood. The nonheme iron halogenases contain a nonheme iron(II) center that is bound to the protein via two histidine amino acid groups, see Scheme 1. In contrast to the αKG-dependent nonheme iron hydroxylases, which bind the protein through interactions with two histidine and one carboxylate group of either Asp or Glu, the latter interaction is missing in the nonheme iron halogenases (Scheme 1).^30^ Instead, a halogen (usually Cl^–^, but there are also reports with Br^–^) binds the iron(II) center directly. These enzymes use αKG as a cosubstrate and dioxygen to form succinate, CO_2_, and a high-valent iron(IV)–oxo (Fe^IV^(O)) species. This species was trapped and characterized with spectroscopic methods for the nonheme iron halogenase SyrB2 and shown to react with an aliphatic group of a substrate through hydrogen atom abstraction to give a putative *cis-*Fe^III^(OH)(Cl) intermediate and carbon radical (R^•^), see Scheme 1.^31^ A halogen “rebound” step then follows, in which the halogen (Cl^•^) is transferred to the nearby radical to give the halogenated R–Cl product, with concomitant reduction of the metal center to Fe^II^. The reaction is often stereospecific and enables the enantioselective synthesis of halogenated substrates efficiently. Interestingly, the competitive OH^•^ rebound to form alcohol (ROH) does not occur. The overall halogenase mechanism is summarized in Scheme 1.

A range of computational studies on the reaction mechanism of αKG-dependent nonheme iron halogenases have been reported.^32−45^ The factors that control the key, bifurcation rebound pathway remain controversial, despite much computational and experimental effort. Several possible ideas have been suggested, including substrate positioning,^31^ oxidant isomerization,^32^ CO_2_ attack on iron(III)–hydroxo,^33^ iron(III)–hydroxo protonation,^34^ electrostatic interactions of the second-coordination sphere,^40^ the relative redox potentials of OH^–^ versus X^–^,^42^ and energetics of the frontier molecular orbitals.^38^ Addressing these different hypotheses experimentally is challenging because the putative *cis*-Fe^III^(OH)(X) intermediate is too short-lived to be observed directly in the enzymatic system under catalytic conditions.

**Scheme 2:**
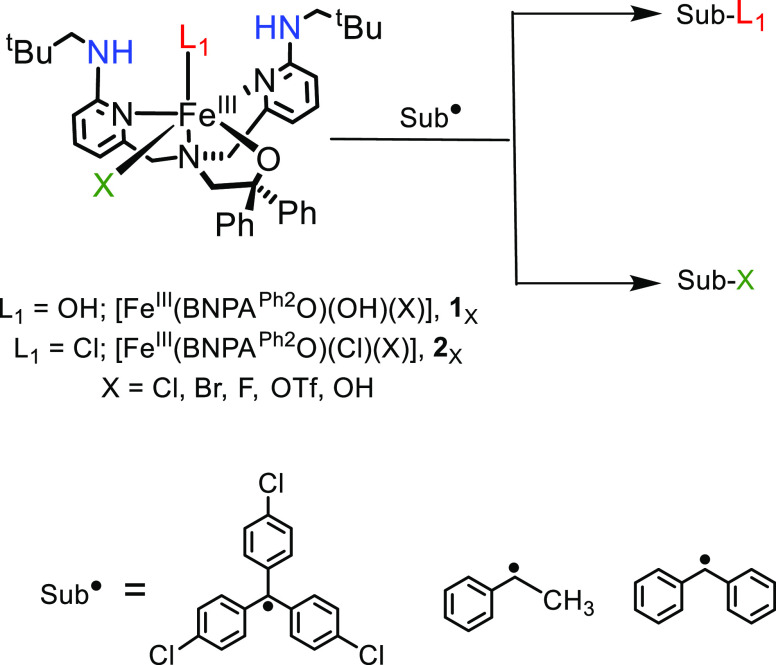
Iron Complexes, Carbon Radicals, and Reactions Studied in This Work

A few groups have synthesized iron complexes that model aspects of the nonheme iron halogenases, although their reactivity varies dramatically.^46−52^ Previous efforts by Goldberg and co-workers have led to the synthesis and structural characterization of model complexes of the postulated *cis*-Fe^III^(OH)(X) intermediate.^53^ These complexes, [Fe^III^(BNPA^Ph2^O)(OH)(X)] (BNPA^Ph2^O = 2-(bis(6-(neopentylamino)pyridin-2-yl)methyl)amino-1,1-diphenylethanolate; X = Cl, Br), see Scheme 2, take advantage of a new ligand with sterically encumbered, second-coordination sphere hydrogen-bonding groups that stabilize the terminal hydroxide ligand. The tetradentate ligand also leaves an open site *cis* to the OH group for coordination of a wide range of anionic donors, including halogens (e.g., Cl^–^, Br^–^). The first study with these *cis*-Fe^III^(OH)(X) complexes involved examining their reactivity toward tertiary carbon radical derivatives (*p*-Y-C_6_H_4_)_3_C^•^ (Y = H, OMe, Cl).^53^ The reactions led exclusively to OH^•^ transfer to give alcohol, and no evidence for halogen transfer was observed. However, the corresponding dichloride complex [Fe^III^(BNPA^Ph2^O)(Cl)_2_] did react with (*p*-Y-C_6_H_4_)_3_C^•^ through halogen transfer to give (*p*-Y-C_6_H_4_)_3_C–Cl and [Fe^II^(BNPA^Ph2^O)(Cl)], indicating that X^•^ transfer, with concomitant reduction of Fe^III^ to Fe^II^, was possible in these systems. The experiments triggered an important question, namely, why do the mixed *cis*-Fe^III^(OH)(X) complexes react with the 3° carbon radicals to give only hydroxylated products, whereas in the enzymes only halogenation is seen? One possibility is that substrate positioning in the enzymes dominates the selectivity; however, a number of computational studies have suggested that there should be an inherent kinetic selectivity for halogen rebound with *cis*-Fe^III^(OH)(X) species even in the absence of an enzyme pocket.^32−38^

In this work, a combined experimental and computational approach has been taken to address the factors that may control the selectivity of hydroxyl versus halogen rebound in the *cis*-Fe^III^(OH)(X) model complexes. One hypothesis is that the nature of the carbon radical influences the outcome of these reactions, and this hypothesis was tested by examining the reactivity of a secondary carbon radical substrate. Detailed computational studies were carried out on these systems analyzing the bifurcated radical rebound pathway, including hypothetical structural derivatives that allowed us to test the influence of hydrogen bonding, steric bulk, positioning (equatorial versus axial) of OH^–^ versus X^–^, and the nature of the carbon radical (3° versus 2°) on rebound selectivity. Taken together, the experimental and computational results lead to significant insights regarding the inherent rebound selectivity of these well-defined model complexes.

## Methods

### Materials

All structures were synthesized and manipulated in a N_2_-filled drybox (under an atmosphere with the following conditions: [O_2_] < 0.2 ppm, [H_2_O] < 0.5 ppm) or using standard Schlenk techniques under an atmosphere of argon unless otherwise noted. Fluorobenzene, acetonitrile, and pentane were distilled in CaH_2_. Tetrahydrofuran was dried over Na/benzophenone and subsequently distilled. All other nondeuterated solvents were obtained from a Pure-solv solvent purification system from Innovative Technologies, Inc. All solvents were degassed by a minimum of three freeze–pump–thaw cycles and stored over freshly activated 3 Å molecular sieves in the drybox following distillation. All other reagents were purchased from commercial vendors and used without further purification. The ligand BNPA^Ph2^OH was prepared by a literature procedure^54^ and was dried over P_2_O_5_ for 12 h under vacuum before metalation. The complexes [Fe^III^(BNPA^Ph2^O)(OH)(Cl)], [Fe^III^(BNPA^Ph2^O)(OH)(Br)], and [Fe^III^(BNPA^Ph2^O)(Cl)_2_] were synthesized by previously reported procedures.^53^ The secondary radical precursors [C_6_H_5_CH(CH_3_)N=N(CH_3_)CHC_6_H_5_] and the chlorinated compound [C_6_H_5_CH(Cl)CH_3_] were synthesized according to a reported procedure.^55^

### Instrumentation

The ^1^H nuclear magnetic resonance (NMR) spectra were recorded on a Bruker 300 MHz or a Bruker 400 MHz NMR spectrometer. Chemical shifts were referenced to reported solvent resonances.^56^

### Reactivity Studies

[Fe^III^(BNPA^Ph2^O)(OH)(Cl)] or [Fe^III^(BNPA^Ph2^O)(Cl)_2_] crystals (70 mg, 0.10 mmol) were dissolved in fluorobenzene (4 mL) with stirring. The solution was transferred to a high-pressure vessel and a fluorobenzene solution (2 mL) of C_6_H_5_CH(CH_3_)N=N(CH_3_)CHC_6_H_5_ (124 mg, 0.52 mmol, 5 equiv) was added. The reaction mixture was heated at 90 °C for 12 h. The reaction mixture was cooled to room temperature and passed through a silica gel column to remove metal impurities. Purification of the chlorinated product C_6_H_5_CH(Cl)CH_3_ by silica gel column chromatography (2% C_2_H_5_OAc/hexane) gave an isolated yield of 40% for [Fe^III^(BNPA^Ph2^O)(OH)(Cl)] and 45% for [Fe^III^(BNPA^Ph2^O)(Cl)_2_]. The identity of the product was confirmed by ^1^H NMR spectroscopy. We attempted the reaction with **1**_Cl_ and **2**_Cl_ at a lower temperature (∼60 °C) but did not see any reaction occur with the iron complex at this temperature.

### Reaction of Fe^II(^BNPA^Ph2^O)(OH) with (*p*-Cl–C_6_H_4_)_3_C–Br

Crystalline Fe^II^(BNPA^Ph2^O)(OH) (10 mg, 0.016 mmol) was dissolved in THF (1 mL). To that solution, excess (*p*-Cl–C_6_H_4_)_3_C–Br (68 mg, 0.16 mmol, 10 equiv) was added and a color change from red to orange was noted. The reaction was stirred for 5 min and then the solvent was removed under vacuum. The orange residue was dissolved in CD_3_CN, and a ^1^H NMR spectrum was recorded. The ^1^H NMR spectrum shows the complete disappearance of the sharp, paramagnetically shifted peaks corresponding to Fe^II^(BNPA^Ph2^O)(OH) and the appearance of the broad peaks that match the previously reported Fe^II^(BNPA^Ph2^O)(Br) complex.^54^

### Computation

All calculations were carried out using the Gaussian-09 software package.^57^ The model was created from the crystal structure coordinates of [Fe^III^(BNPA^Ph2^O)(OH)(Cl)], and hydrogen atoms were added in GaussView.^53,58^ Geometry optimizations and analytical frequencies were done using the unrestricted B3LYP density functional approach with a basis set containing LANL2DZ (with core potential) on iron and 6-31G on the rest of the atoms: basis set BS1.^59−63^ Initially, geometry optimizations were done in the gas phase followed by single-point calculations with a larger basis set (basis set BS2) and solvent included; however, this resulted in low energy barriers that often dropped below the reactant complexes. We, therefore, reoptimized all local minima and transition states and the subsequent frequency calculations with a solvent model included at UB3LYP/BS1 level of theory using the continuum polarized conductor model with a dielectric constant mimicking a tetrahydrofuran solvent.^64^ Basis set BS2 has a triple-ζ quality LACV3P + basis set (with core potential) on iron and 6-311+G* on the rest of the atoms. Some structures were also optimized at UB3LYP/BS2 (Supporting Information, Figure S7); however, this gave only small changes to the structures and energies. Transition states were confirmed by running an analytical frequency calculation that showed a single imaginary mode for the correct transition. Additional intrinsic reaction coordinate (IRC) scans were performed for a selection of transition states and further confirmed their characterization. In particular, the IRCs led to the reactant complexes in one direction and the product complexes in the other direction.

The computational methods and approaches follow previous studies from our groups and have been extensively tested and validated against experimental data and were shown to correctly reproduce spectroscopic parameters, product selectivities, and reaction rates.^65−67^

## Results and Discussion

### Experiment

The reactivity of **1**_Cl_ was previously examined with the tertiary carbon radicals (*p*-Y-C_6_H_4_)_3_C^•^ (Y = OMe, H, Cl), resulting in dominant OH^•^ transfer to form the (*p*-Y-C_6_H_4_)_3_COH products with yields >85%.^53^ By contrast, the dichloro compound, [Fe^III^(BNPA^Ph2^O)(Cl)_2_] (**2**_Cl_), reacted through halogen transfer to give (*p*-Y-C_6_H_4_)_3_CCl with yields of 75–85%, demonstrating that halogen transfer was feasible. Our hypothesis to explain this selectivity was that the formation of the halogenated product was thermodynamically unfavorable because of the weak nature of the C–Cl bonds in trityl derivatives, in contrast to the C–OH product. To test this hypothesis, we sought a secondary carbon radical that could be reacted directly with **1**_Cl_ to give a product in which the C–Cl bond is significantly more thermodynamically stable. This 2° radical might then favor halogen transfer. The phenylmethylmethyl radical was selected for study because of its ease of generation from a diazo precursor.^48^

**Figure 1:**
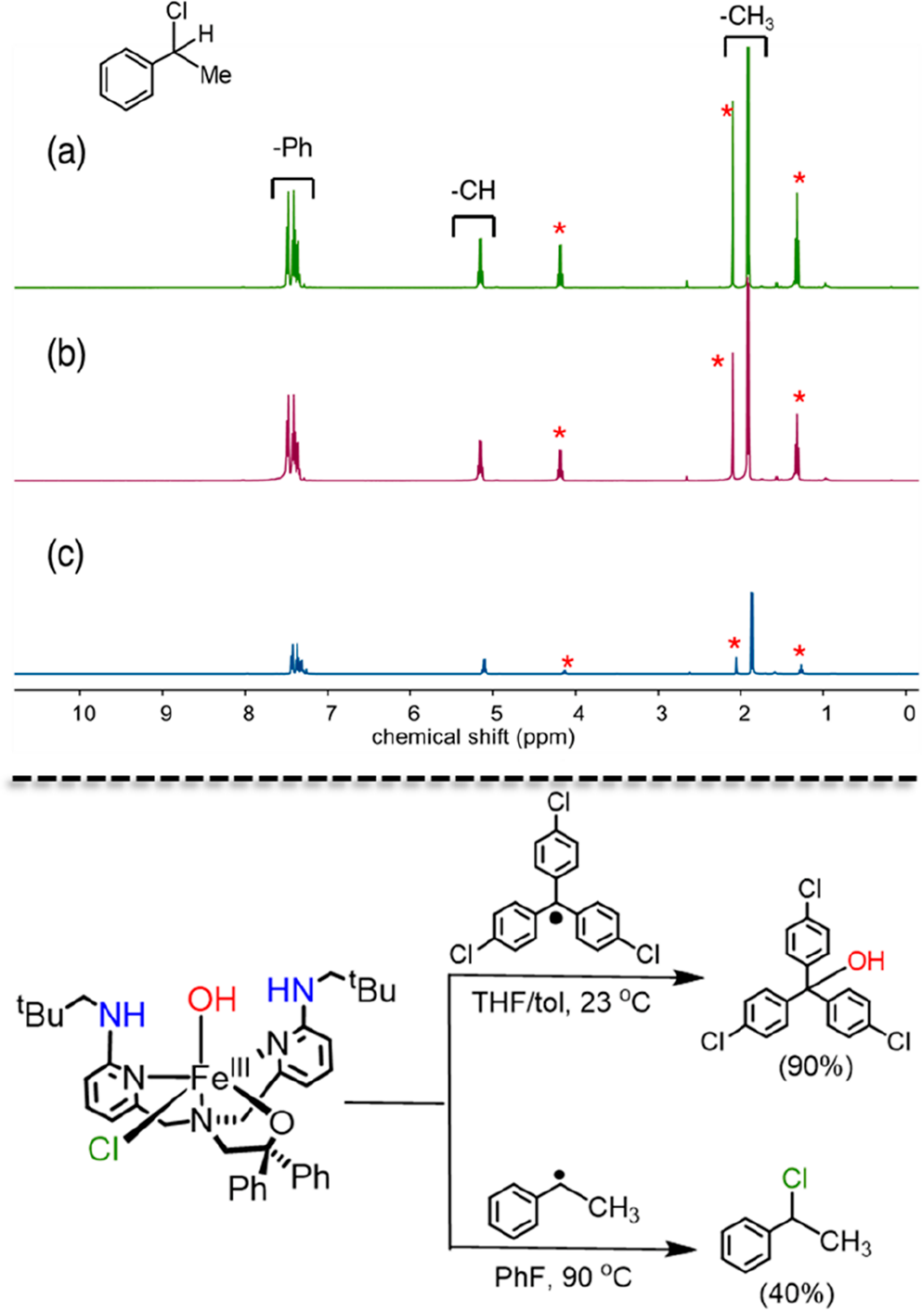
Top: ^1^H NMR spectra obtained in CDCl_3_ of isolated C_6_H_5_CH(Cl)CH_3_ from the reaction of **1**_Cl_ with C_6_H_5_CH(CH_3_)N=N(CH_3_)CHC_6_H_5_ (a), from the reaction of **2**_Cl_ with C_6_H_5_CH(CH_3_)N=N(CH_3_)CHC_6_H_5_ (b), and authentic C_6_H_5_CH(Cl)CH_3_ (c). Residual solvent signals are marked with a red asterisk (*). Bottom: Reactivity of **1**_Cl_ with tertiary and secondary carbon radicals.

Reaction of **1**_Cl_ with phenylmethylmethyl radical was performed in fluorobenzene and product distributions were measured with ^1^H NMR. As can be seen in Figure 1, the complex **1**_Cl_ reacts selectively with phenylmethylmethyl radical to form 1-chloro-1-phenylethane. Although the yield for the halogenated product was only 40%, there was no evidence for the corresponding alcohol or ketone products that would arise from OH^•^ transfer. The dichloro complex **2**_Cl_ was also investigated in a reaction with phenylmethylmethyl radical and gave the halogenated product (45% yield). These results support our hypothesis; changing the substrate from 3° to 2° carbon radical leads to halogenation. One possibility is that the halogenation of the secondary carbon radical arises from a free radical pathway, especially given the elevated temperature of the reaction conditions. Although complex **1**_Cl_ appears to be stable for at least 12 h at 90 °C (see the Methods Section), it is difficult to rule out completely the release of a small amount of free Cl^•^, which could react with R^•^ to give the chlorinated product. However, control experiments in which **1**_Cl_ was heated under the same conditions in the presence of compounds with weak C–H bonds (e.g., 9,10-dihydroanthracene, xanthene, triphenylmethane) did not lead to any chlorinated organic products and did not show any significant decomposition of **1**_Cl_ as observed by ^1^H NMR spectroscopy. Chlorination of even stronger, aliphatic C–H bonds would be expected if free Cl^•^ was generated.^68^

**Scheme 3:**
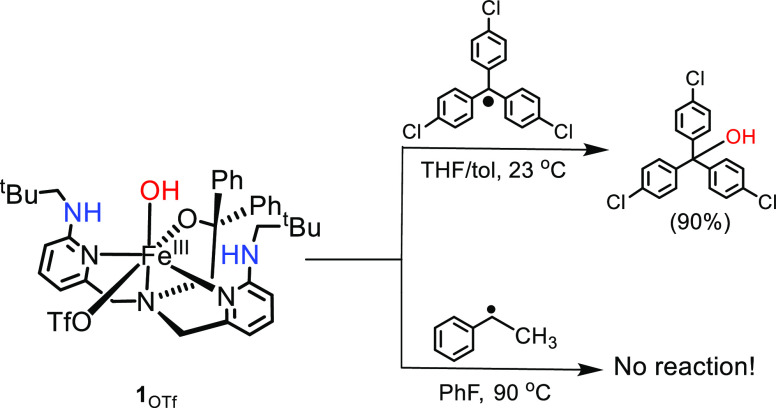
Reactions of 1_OTf_ with Tertiary and Secondary Carbon Radicals

With the halogen-transfer results observed for the 2° carbon radical, we questioned whether OH^•^ transfer could be observed for the same radical if an Fe^III^ species were employed that lacks a competitive rebound partner. To address this question, we examined the triflate-ligated [Fe^III^(BNPA^Ph2^O)(OH)(OTf)] (**1**_OTf_). No reaction was observed between **1**_OTf_ and phenylmethylmethyl radical under the same conditions. On the other hand, reaction of **1**_OTf_ with the 3° carbon radical, i.e., (*p*-Cl–C_6_H_4_)_3_C^•^, leads to (*p*-Cl–C_6_H_4_)_3_COH in good yield (Scheme 3).^53^ The decomposition pathways for the 2° carbon radical (e.g., dimerization, desaturation) are much faster than the decomposition pathways for (*p*-Cl–C_6_H_4_)_3_C^•^, and these results suggest that there is a kinetic barrier for OH^•^ transfer that is outcompeted by one or more of the 2° carbon radical decomposition pathways.

### Theory

**Figure 2:**
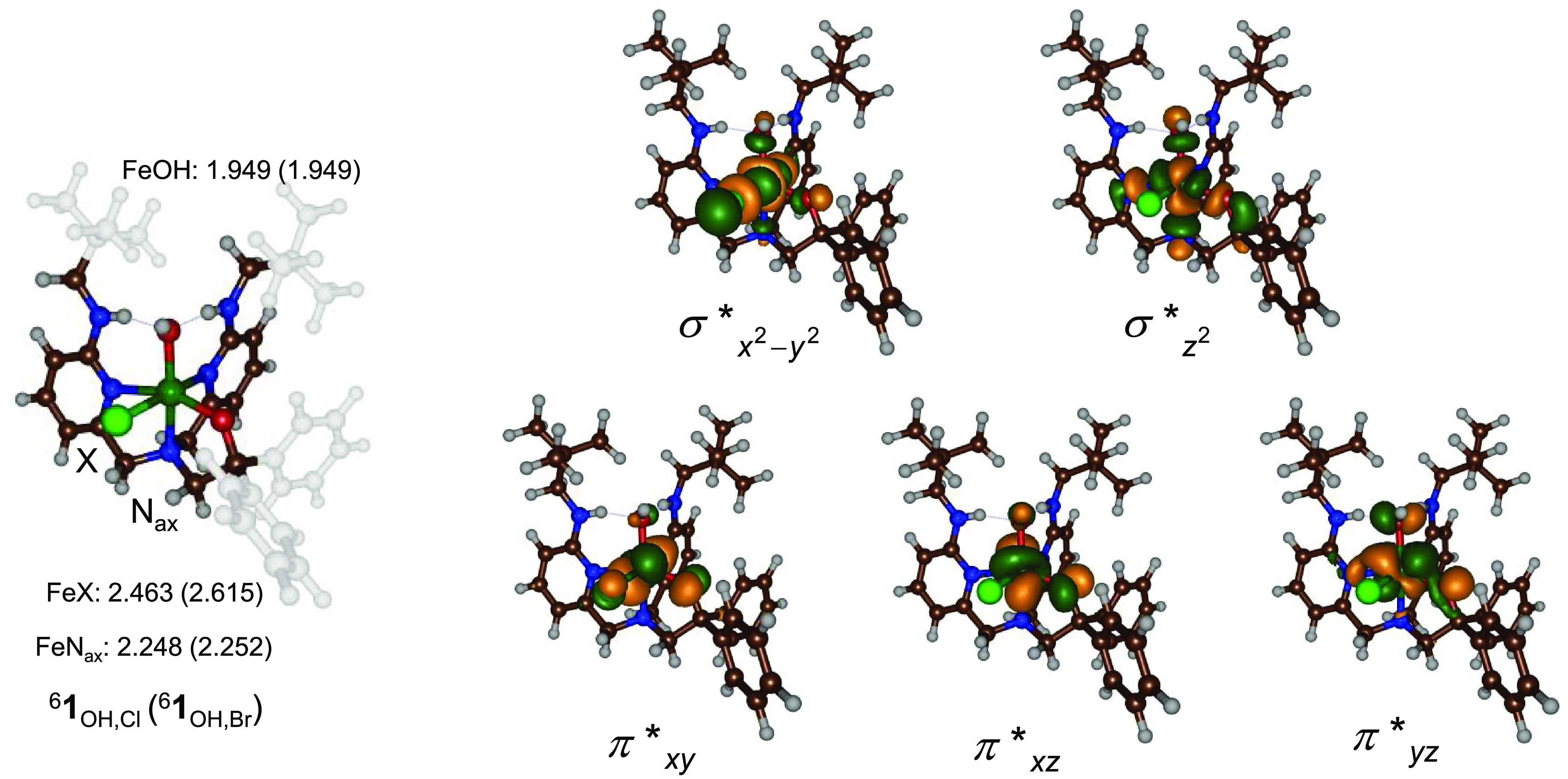
UB3LYP/BS1-optimized geometries of the isolated reactants ^6^**1**_Cl_/^6^**1**_Br_ as obtained in Gaussian with bond lengths given in Å. Relevant molecular valence orbitals are shown on the right.

A density functional theory study was undertaken to examine the halogenation versus hydroxylation bifurcation pathways for the nonheme iron(III) model complexes. We started with calculating the reactant complexes **1**_Cl_ and **1**_Br_, and their optimized geometries are shown in Figure 2. These complexes have the OH ligand trans to the axial nitrogen atom of the BNPA^Ph2^O^–^ ligand (N_ax_). Several possible spin states were tested for both complexes, see Tables S1–S5, and the lowest energy conformation was found to be the sextet spin state with five metal-type orbitals singly occupied. This result is in good agreement with experimental data,^53^ and with previous calculations on nonheme iron complexes that typically assign the iron(III)–hydroxo species as a high-spin state.^69−74^ The molecular orbitals of ^6^**1**_Cl_ are shown on the right-hand side of Figure 2. These are dominated by the metal 3d contribution and labeled π*_xy_, π*_xz_, π*_yz_, σ*_z2_, and σ*_x2–*y*2_, whereby the *z*-axis is defined along the O–Fe axis. The three π* orbitals represent the antibonding interactions of the hydroxo ligand and iron atom through the mixing of the atomic 2p orbitals on oxygen with 3d*_xy_*, 3d*_xz_*, and 3d*_yz_* on iron. Higher in energy are the two σ* orbitals for the antibonding interactions along the *z*-axis and in the *xy*-plane: σ*_z2_ and σ*_x2–*y*2_. Thus, the σ*_z2_ orbital represents the interaction of the iron 3d_*z*2_ with 2p*_z_* orbitals on O(alkyl) and N_ax_, while the σ*_x2–*y*2_ orbital mixes the 3d_*x*2–*y*2_ orbital on iron with 2p*_x_* and 2p*_y_* orbitals of the N/O atoms in the equatorial plane and 3p/4p orbitals on Cl/Br. The quartet spin state ^4^**1**_Cl_ is 7.4 kcal mol^–1^ higher in energy than the sextet spin state and has configuration π*_xy_^2^ π*_xz_^1^ π*_yz_^1^ σ*_x2–*y*2_^1^, while the doublet spin state ^2^**1**_Cl_ with π*_xy_^2^ π*_xz_^2^ π*_yz_^1^ is 10.9 kcal mol^–1^ higher lying. Therefore, we focused the work on the sextet spin complexes only.

The hydrogen-bonding interactions of the pendant amine groups hold the hydroxo ligand in a tight orientation. The Fe–O distance is relatively long at 1.949 Å for both ^6^**1**_Cl_ and ^6^**1**_Br_, while the axial ligand distance (Fe–N_ax_) is 2.248/2.252 Å for ^6^**1**_Cl_/^6^**1**_Br_. Nevertheless, our calculated values of ^6^**1**_Cl_/^6^**1**_Br_ match the crystal structure coordinates and DFT-optimized structures of ref (53) well. Most likely, the origin of the long Fe–O distances comes from the hydrogen-bonding interactions in the model as calculations for analogous nonheme iron(III)−hydroxo complexes without these interactions present much shorter Fe−O distances are found.^75,76^ Indeed, experimental measurements put Fe–OH distances of biomimetic models in the range between 1.8 and 2.0 Å.^77−79^ The Fe–Cl/Fe–Br distances are 2.463/2.615 Å, respectively, and compare well to the experimentally determined crystal structure coordinates of 2.3945/2.5835 Å.^53^ Overall, the geometries of ^6^**1**_Cl_ and ^6^**1**_Br_ are also in good agreement with calculated analogous nonheme iron halogenase enzyme structures and biomimetic model complexes.^32−41,80,81^ We also calculated an isomer of **1**_Cl_, designated **1′**_Cl_, whereby the equatorial ligands are in a different position and the alkoxide group is trans to the halide in the xy-plane. Isomer ^6^**1′**_Cl_ is 7.3 kcal mol^–1^ higher in energy than **1**_Cl_ and hence was not considered further. These calculations match the crystal structure observations that only **1**_Cl_ is observed.

Subsequently, we created a reactant complex containing ^6^**1**_X_ (X = Cl, Br, F) with ^2^(*p*-Cl-C_6_H_4_)_3_C^•^, i.e.,^5^**Re**_1X_, and ran a geometry optimization. Transition states were located for halogen transfer (^5^**TS**_Cl,1X_) and hydroxyl transfer (^5^**TS**_OH,1X_), leading to the iron(II) complexes with either bound (*p*-Cl–C_6_H_4_)_3_CCl (^5^**Pr**_Cl,1X_) or (*p*-Cl–C_6_H_4_)_3_COH (^5^**Pr**_OH,1X_). Generally, nonheme iron hydroxylases and model complexes typically give an iron(III)–hydroxo intermediate in a high-spin state coupled to a substrate radical with down-spin. In particular, we considered an overall spin multiplicity of quintet spin and an electronic configuration of ^5^σ = π*_xy_^1^ π*_xz_^1^ π*_yz_^1^ σ*_z2_^1^ σ*_x2–*y*2_^1^ π_Sub_^1^ with π_Sub_ the radical on the substrate.^73−88^ As such, this intermediate has the metal-type unpaired electrons as up-spin while the radical on the substrate is down-spin in an overall quintet spin state. For our reactant complexes, all structures indeed have this electronic configuration. We attempted to swap molecular orbitals and obtain a reactant complex for the ^3^π- and ^5^π-configuration with orbital occupation π*_xy_^2^ π*_xz_^1^ π*_yz_^1^ σ*_x2–*y*2_^1^ π_Sub_^1^; however, during the SCF convergence in the quintet spin state it returned to the ^5^σ configuration. We then ran the calculation in the triplet spin state and located the antiferromagnetically coupled state with π*_xy_^2^ π*_xz_^1^ π*_yz_^1^ σ*_x2–*y*2_^1^ π_Sub_^1^ configuration. However, this triplet state ^3^**Re**_1Cl_ was considerably higher in energy than ^5^**Re**_1Cl_, and the same ordering was found for **Re**_1Br_. Therefore, we did not consider states along the ^3,5^π-pathways further. For several models, we calculated the triplet spin mechanism for OH and halogen transfer, but for all of these systems, the triplet spin reactants and barriers were found to be considerably higher in energy than those on the quintet spin state surface (Tables S2 and S5), and hence we will focus on the quintet spin results only. These results match experimental observation of a high-spin ground state for reactant and product complexes. Finally, a septet spin reactant complex was optimized (^7^**Re**_1Cl_) with the same electronic configuration as the quintet spin state but all unpaired electrons ferromagnetically coupled. This state is within 1 kcal mol^–1^ of the quintet spin state; however, due to the absence of available low-lying molecular orbitals to accept an α-spin electron, these septet spin states generally have high barriers for substrate activation and we did not consider its mechanism further.^89^

**Figure 3:**
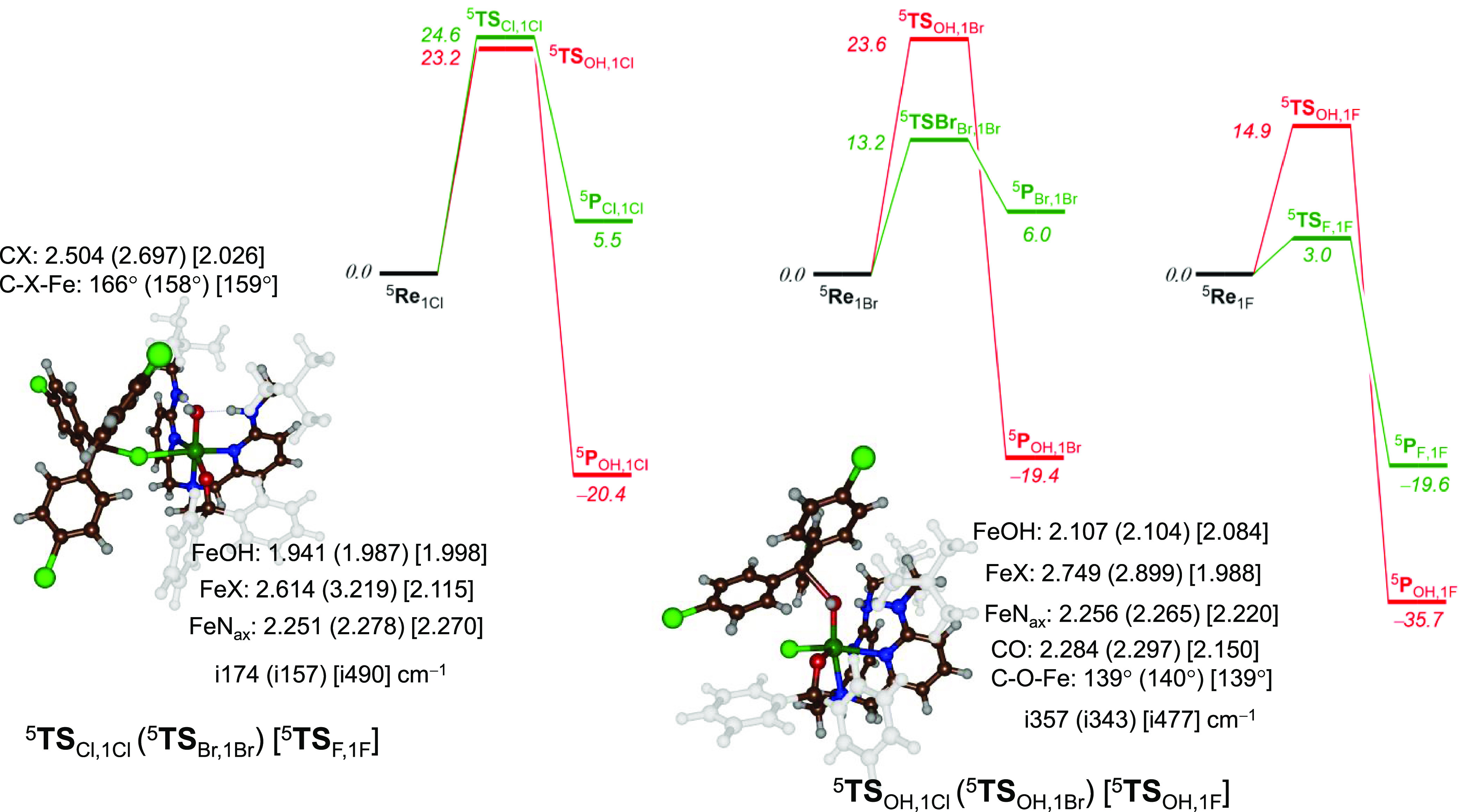
Potential energy landscape for halogen versus hydroxyl transfer in complexes **1**_Cl_/**1**_Br_/**1**_F_ as obtained at UB3LYP/BS2//UB3LYP/BS1 level of theory with the solvent and zero-point corrections included. Energies relative to the reactant complexes are in kcal mol^–1^, while transition-state structures give bond lengths in Å, angles in degrees, and the imaginary frequency in cm^–1^.

The potential energy landscapes for the competitive halogen and hydroxyl rebound pathways for **1**_Cl_, **1**_Br_, and **1**_F_ are shown in Figure 3. For all halogens, the reactions contain a single concerted group transfer step for either OH or X rebound. Consequently, the electron transfer and bond-formation processes occur simultaneously in transition states. For all complexes **1**_Cl_/**1**_Br_/**1**_F_, the formation of a reactant complex from isolated iron(III) and substrate constituents is found when (*p*-Cl–C_6_H_4_)_3_C^•^ radical is added to the system. No charge transfer is seen and a spin density of close to −1.00 is found on the substrate in all reactant complexes. The iron atom remains in the iron(III) oxidation state. It can be envisaged that the reactant complex has a large solvent cage that surrounds it. The reactant complexes show the same general structural features as isolated reactants, although the Fe–OH distance drops by 0.014 and 0.019 Å between isolated reactants and ^5^**Re**_1Cl_ and ^5^**Re**_1Br_ (Supporting Information, Figure S2), respectively. At the same time, Fe–Cl and Fe–Br distances elongate in the reactant complexes to values of 2.561 and 2.719 Å.

The lowest energy group transfer barrier for the reaction of ^5^**Re**_1Cl_ with (*p*-Cl-C_6_H_4_)_3_C^•^ as a substrate is via ^5^**TS**_OH,1Cl_, whereas for ^5^**Re**_1Br_ and ^5^**Re**_1F_, the halogen-transfer barrier is lower. In particular, for **1**_Cl_, the two transition states are close in energy with the OH transfer at 23.2 kcal mol^–1^ above ^5^**Re**_1Cl_, while the chlorine transfer barrier is 24.6 kcal mol^–1^ above ^5^**Re**_1Cl_. The order of transition states is reversed for **Re**_1Br_ and **Re**_1F_ with the lowest one leading to halogen transfer via a barrier ^5^**TS**_Br,1Br_ of 13.2 kcal mol^–1^ above ^5^**Re**_1Br_, while the fluorine transfer barrier is 3.0 kcal mol^–1^ above ^5^**Re**_1F_. By contrast, the OH rebound barriers for these processes are 23.6 and 14.9 kcal mol^–1^ above the ^5^**Re**_1Br_ and ^5^**Re**_1F_ complexes, respectively. The OH rebound barriers for the reactions of **1**_Cl_ and **1**_Br_ with (*p*-Cl–C_6_H_4_)_3_C^•^ are very similar and an equatorial Cl or Br ligand does not seem to affect the transition-state structure and energetics dramatically.

The transition states for the hydroxylation reactions with **1**_Cl_ and **1**_Br_ have similar electronic configurations and exhibit a large amount of charge transfer from the substrate to oxidant: ρ_sub_ = −0.27 in ^5^**TS**_OH,1Cl_ and ρ_sub_ = −0.25 in ^5^**TS**_OH,1Br_. Much less charge transfer is observed for ^5^**TS**_OH,1F_ (ρ_sub_ = −0.40), consistent with an earlier transition state on the potential energy surface. The halogen-transfer barriers are significantly lower in energy for **1**_Br_/**1**_F_ than for **1**_Cl._ This trend is seen along a series of S_N_2 reactions for a halide with methanol.^90^ The halogen-transfer barriers are electronically different from the hydroxylation barriers, with a ρ_sub_ = −0.75 in ^5^**TS**_Cl,1Cl_, while ρ_sub_ = −0.27 or −0.35 for ^5^**TS**_Br_,_1Br_ and ^5^**TS**_F_,_1F_. Generally, transition states with more charge transfer are lower in energy in agreement with what is seen here.^66^

After the transition states, the systems relax to a product complex of alcohol with the iron(II) complex or the halogenated substrate with iron(II). The halogen-transfer product complexes for **1**_Cl_ and **1**_Br_, however, are higher in energy than the reactant complexes by 5.5 kcal mol^–1^ (Cl) and 6.0 kcal mol^–1^ (Br) and consequently have relatively small reverse barriers to return the systems back to the reactant complexes. These calculations are consistent with the reactant (^5^**Re**_1X_) and halide-substituted products (^5^**Pr**_1X_) for X = Cl/Br being in equilibrium during their lifetime in the solvent cages. The hydroxylation reaction, by contrast, is highly exothermic by ∼20 kcal mol^–1^ and leads to reverse barriers from products back to reactants of well over 40 kcal mol^–1^. The latter high-energy barriers are inaccessible at room temperature, making the hydroxylation an irreversible process, while the halogen-transfer step is in equilibrium. Unfortunately, it is not possible to measure experimental kinetics profiles for this system due to the lack of suitable UV–vis absorption bands.

Interestingly, the pathway for fluorine transfer from ^5^**Re**_1F_ has a small barrier and is strongly exothermic by almost 20 kcal mol^–1^. These calculations predict that **1**_F_ should react through fluorine transfer efficiently and form the (*p*-Cl–C_6_H_4_)_3_CF product. Attempts to synthesize the Fe^III^(OH)(F) complex have been unsuccessful thus far, and hence this prediction has not yet been tested experimentally.

The transition states for OH rebound by **1**_Cl_/**1**_Br_/**1**_F_ are similar in structure. The Fe–O distances are 2.107, 2.104, and 2.084 Å for the ^5^**TS**_OH,1Cl_,^5^**TS**_OH,1Br_ and ^5^**TS**_OH,1F_ structures, respectively, while the C–O distances are 2.284, 2.297, and 2.150 Å. The substrate attacks from the side in all three transition states, with an angle Fe–O–C ranging from 139 to 140°. The imaginary frequency in all OH transfer transition states represents the Fe–O–C stretch vibration and has magnitudes of i357 cm^–1^ for ^5^**TS**_OH,1Cl_, i343 cm^–1^ for ^5^**TS**_OH,1Br_, and i477 cm^–1^ for ^5^**TS**_OH,1F_. These values are typical for OH rebound transition states and of the same order of magnitude as compared to analogous ones from the literature.^91,92^ The transition states for halogen transfer have much lower imaginary frequencies of i174 cm^–1^ for ^5^**TS**_Cl,1Cl_ and i157 cm^–1^ for ^5^**TS**_Br,1Br_, while the fluorine transfer has an imaginary frequency of i490 cm^–1^. These imaginary frequencies are low for X = Cl/Br as a heavy atom is transferred, while for lighter elements like F as well as a OH group, the imaginary frequencies are larger. As Cl is next to sulfur in the periodic table with similar mass, this warrants a comparison of the Cl-transfer barriers with those for sulfoxidation. In particular, sulfoxidation barriers by iron(IV)–oxo complexes usually have a small imaginary frequency around i100–i200 cm^–1^ in line with what is seen for Cl transfer.^93^ Although the Fe–O and Fe–N_ax_ (axial nitrogen atom) distances are very similar for the ^5^**TS**_Cl,1Cl_,^5^**TS**_Br,1Br_, and ^5^**TS**_F,1F_ complexes, there are distinct differences calculated for the Fe–X and C–X distances. These differences correlate with the size of the halide that is being transferred. The C–Cl, C–Br, and C–F distances are 2.504, 2.697, and 2.026 Å, while the Fe–Cl, Fe–Br, and Fe–F distances are 2.614, 3.219, and 2.115 Å, respectively. The C–X–Fe angle is 166° for X = Cl, while it is slightly shorter for X = Br/F with values of 158/159°.

Taken together, the DFT calculations provide an excellent theoretical framework to explain the experimental results. The fact that no halogenation was observed for **1**_Cl_/**1**_Br_ with (*p*-Y-C_6_H_4_)_3_C^•^ can be rationalized by a rapid equilibrium between the endergonic halogenation product and reactant complexes, together with a highly exergonic, irreversible hydroxylation pathway, as shown by the energetics in Figure 3. These calculations also support our original hypothesis that hydroxylation is favored over halogenation for the 3° radical because of the weak C–Cl versus C–OH bonds formed in the products. Furthermore, the DFT calculations imply that the reactions of ^5^**Re**_1Br_ and ^5^**Re**_1F_ show kinetics opposite to the Bell–Evans–Polanyi principle, where the barriers for halogen transfer are significantly lower than those for hydroxyl transfer, which is opposite to the ordering of the thermochemistry.

**Scheme 4:**
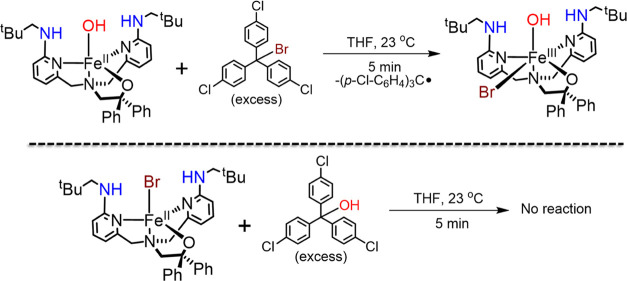
Halogenated and Hydroxylated Product Complexes Showing Feasible Reverse Reactions for Br Transfer and Not for OH Transfer

The reversible equilibrium suggested by the calculations for the halogen-transfer step is an important piece of the puzzle for understanding the preferred hydroxylation selectivity in the model complexes. We set out to obtain experimental evidence for this idea by examining the separate reaction between the iron(II) complex Fe^II^(BNPA^Ph2^O)(OH) and the alkyl halide (*p*-Cl–C_6_H_4_)_3_C–Br (Scheme 4). Upon addition of excess alkyl halide (10 equiv) to the ferrous precursor in THF, an immediate color change was noted. Analysis by ^1^H NMR spectroscopy indicated that the Fe^II^ complex was converted into the ferric species Fe^III^(BNPA^Ph2^O)(OH)(Br). In contrast, no reaction is observed between Fe^II^(BNPA^Ph2^O)(Br) and (*p*-Cl–C_6_H_4_)_3_C–OH. These data provide direct experimental support for the equilibrium hypothesis, where back electron transfer from the Fe^II^(OH) species to the alkyl halide results in homolytic C–X bond cleavage and production of the *cis*-Fe^III^(OH)(X) reactant complex.

**Figure 4:**
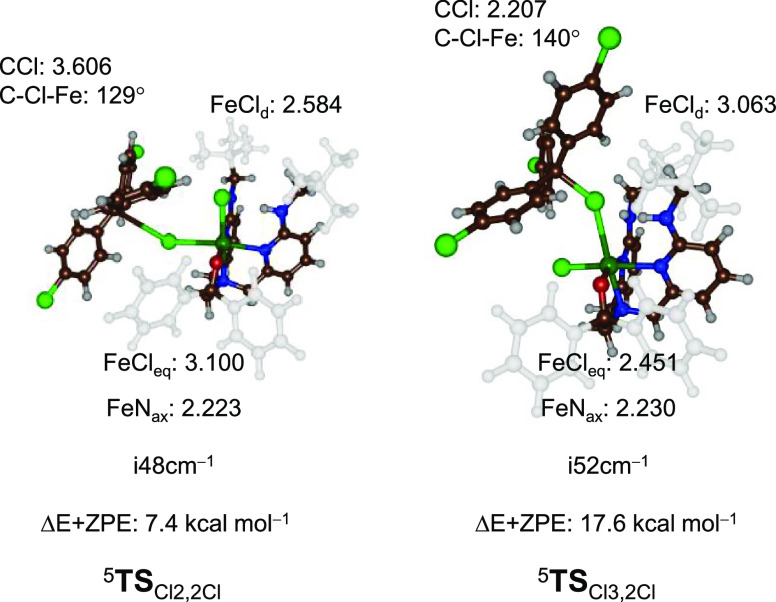
Optimized geometries of **TS**_Cl2,2Cl_ and **TS**_Cl3,2Cl_ as obtained in Gaussian. Energies (Δ*E* + ZPE) are relative to the reactant complexes in kcal mol^–1^, while transition-state structures give bond lengths in Å, angles in degrees, and the imaginary frequency in cm^–1^.

**Scheme 5:**
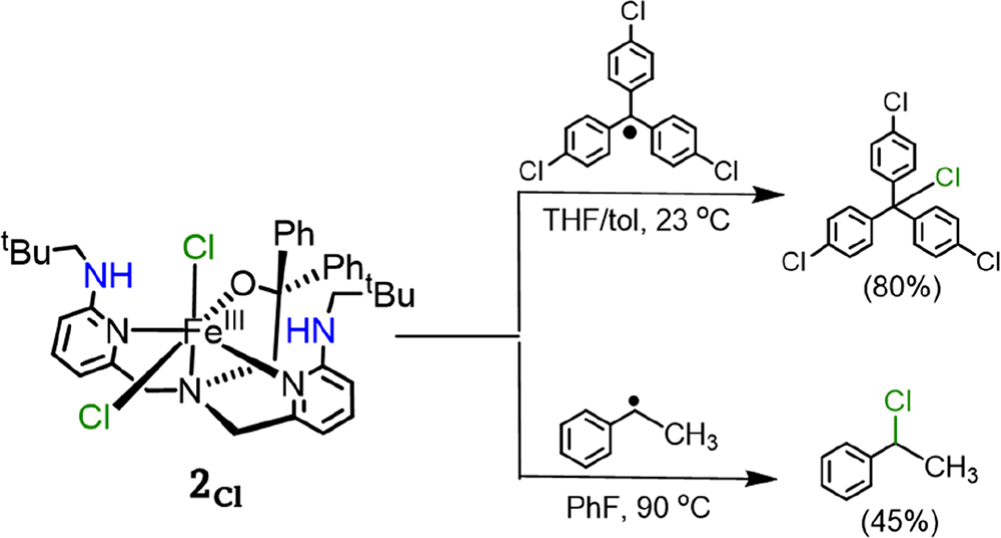
Reactions of 2_Cl_ with Tertiary and Secondary Carbon Radicals

The reaction of the dichloro compound **2**_Cl_ with carbon radical was examined computationally to determine if the mechanism of halogen transfer differed for an equatorial versus distal Cl ligand. Experimentally, the reaction of **2**_**Cl**_ with (*p*-Cl–C_6_H_4_)_3_C^•^ and phenylmethylmethyl radicals (Scheme 5) gives efficient halogen transfer with yields of 80 and 45%, respectively. The transition states for the Cl-transfer reactions from the equatorial (**TS**_Cl2,2Cl_) and distal (**TS**_Cl3,2Cl_) positions in ^5^**Re**_2Cl_ were located (Figure 4). Transfer of the equatorial Cl ligand via ^5^**TS**_Cl2,2Cl_ has a small barrier of 7.4 kcal mol^–1^, while the distal Cl ligand transfer barrier via ^5^**TS**_Cl3,2Cl_ is much higher in energy, i.e., at 17.6 kcal mol^–1^. These calculations confirm that halogen transfer from the equatorial position of iron(III)–hydroxo(halide) complexes should be more likely. The chlorine transfer transition-state structures for the chlorine in different positions in **2**_Cl_ exhibit significant differences. The ^5^**TS**_Cl2,2Cl_ transition state has the transferring halogen midway between the donor and acceptor group with Fe–Cl and C–Cl distances of 3.100 and 3.606 Å. In contrast, the barrier for transfer of the distal chlorine atom ^5^**TS**_Cl3,2Cl_ has a much shorter C–Cl distance of 2.207 Å, and the interaction angle Fe–Cl–C is more bent at 140°.

**Figure 5:**
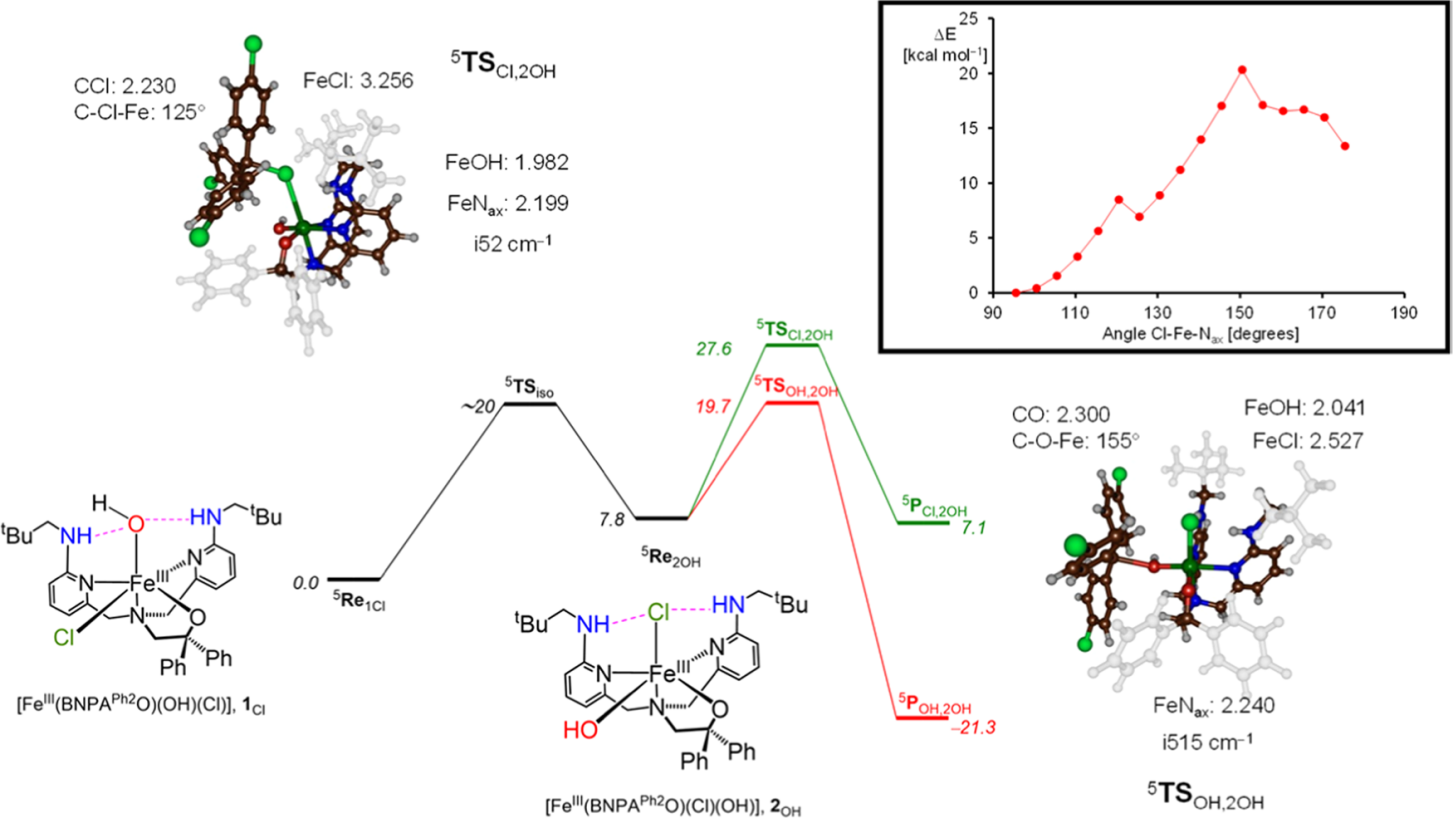
Potential energy landscape for halogen versus hydroxyl transfer after isomerization of complex **1**_Cl_ into **2**_OH_ as obtained at UB3LYP/BS2//UB3LYP/BS1 level of theory with the solvent and zero-point corrections included. Energies are in kcal mol^–1^, while structures give bond lengths in Å, angles in degrees, and the imaginary frequency in cm^–1^. The constraint geometry scan for isomerization from **1**_Cl_ to **2**_OH_ is shown in the inset.

Borowski and co-workers suggested a mechanism for nonheme iron halogenases,^32^ where only the ligand trans to the axial nitrogen atom, i.e., the distal group, is transferred. As the reactant structure has the halide in nonheme iron halogenases in the equatorial position, an isomerization would be needed in the iron(IV)–oxo(halide) or iron(III)–hydroxo(halide) system that swaps the positions of the hydroxo and halide groups. A barrier of about 12 kcal mol^–1^ for the isomerization was calculated for the enzyme.^32^ For biomimetic models without second-coordination sphere perturbations, barriers of around 12 kcal mol^–1^ were obtained for the isomerization of changing positions of a hydroxo and halide group to an iron(III) system.^80^ An isomer of [Fe^III^(BNPA^Ph2^O)(Cl)(OH)], in which the OH and Cl groups have been exchanged in the distal and equatorial positions, ([Fe^III^(BNPA^Ph2^O)(Cl)(OH)], **2**_OH_) was examined computationally. A reactant complex (^5^**Re**_2OH_) with (*p*-Cl–C_6_H_4_)_3_C^•^ was calculated, as well as the chlorine and hydroxyl-transfer transition states (^5^**TS**_Cl,2OH_ and ^5^**TS**_OH,2OH_) leading to products (^5^**Pr**_Cl,2OH_ and ^5^**Pr**_OH,2OH_). In addition, we calculated an isomerization pathway to convert ^5^**Re**_1Cl_ into ^5^**Re**_2OH_ through a constraint geometry scan that fixes the Cl–Fe–N_ax_ angle and calculates the energies of all structures. The geometry scan for the conversion of ^5^**Re**_1Cl_ into ^5^**Re**_2OH_ through rotation of their halide and hydroxyl groups prior to the reaction with (*p*-Cl–C_6_H_4_)_3_C^•^ is shown in Figure 5. The geometry scan shows that the isomerization from ^6^**1**_Cl_ to form ^6^**2**_OH_ costs a considerable amount of energy, i.e., at least 20 kcal mol^–1^. No change in electronic configuration happens during the scan, but minor steric interactions are seen that affect the energetics.

The isomerization is competitive with halogen and hydroxyl transfer in ^5^**Re**_1Cl_ as ^5^**TS**_OH,1Cl_ and ^5^**TS**_Cl,1Cl_ are of similar energy (see Figure 3). Note that ^5^**Re**_2OH_ is less stable than ^5^**Re**_1Cl_ by 7.8 kcal mol^–1^. The iron(III)–hydroxo(chloride) isomer **2**_OH_ has favorable OH rebound by 8 kcal mol^–1^ over chlorine transfer, and consequently, hydroxyl rebound in ^5^**Re**_2OH_ will give dominant hydroxylation products. The OH rebound barrier from ^5^**Re**_2OH_ is 11.9 kcal mol^–1^, while chlorine transfer has a barrier of 19.8 kcal mol^–1^ above ^5^**Re**_2OH_, indicating that both **1**_Cl_ and **2**_OH_ should give preferential OH rebound over halogenation and dominant (*p*-Cl–C_6_H_4_)_3_COH products. Isomerization therefore does not change the chemoselectivity of the reaction for this biomimetic model complex.

Optimized geometries of ^5^**TS**_Cl,2OH_ and ^5^**TS**_OH,2OH_ transition states are shown in Figure 5. The halogen-transfer transition state has a very small imaginary frequency of i52 cm^–1^ for the C–Cl–Fe stretch vibration and is productlike with a long Fe–Cl bond of 3.256 Å and short C–Cl distance of 2.230 Å. The substrate approach angle is considerably bent, namely, the Fe–Cl–C angle is 125°. On the other hand, the ^5^**TS**_OH,2OH_ structure is more reactant-like with a short Fe–O distance of 2.041 Å and a relatively long C–O interaction of 2.300 Å. The substrate approach is much closer to linearity for ^5^**TS**_OH,2OH_ than for ^5^**TS**_Cl,2OH_ with an Fe–O–C angle of 155°. Taken together, the results from the computational study on the **2**_OH_ isomer indicate that if the conversion to this isomer were occurring for **1**_Cl_, it would not influence the final product selectivity of the reactions with the 3° carbon radicals. Thus, a mechanism involving isomerization in solution for **1**_Cl_ cannot be ruled out, although there is no evidence to suggest that such a mechanism plays an important role here. It is also interesting to note that the OH rebound barrier is lowered from 23.2 to 11.9 kcal mol^–1^ between model **1**_Cl_ and **2**_OH_, while the corresponding Cl-transfer barriers are similar for the two complexes. These computational results imply that H-bonding has a much stronger influence on OH^•^ transfer than Cl^•^ transfer, which does correlate nicely with the stronger H-bond accepting ability of OH^–^ versus Cl^–^.

**Figure 6:**
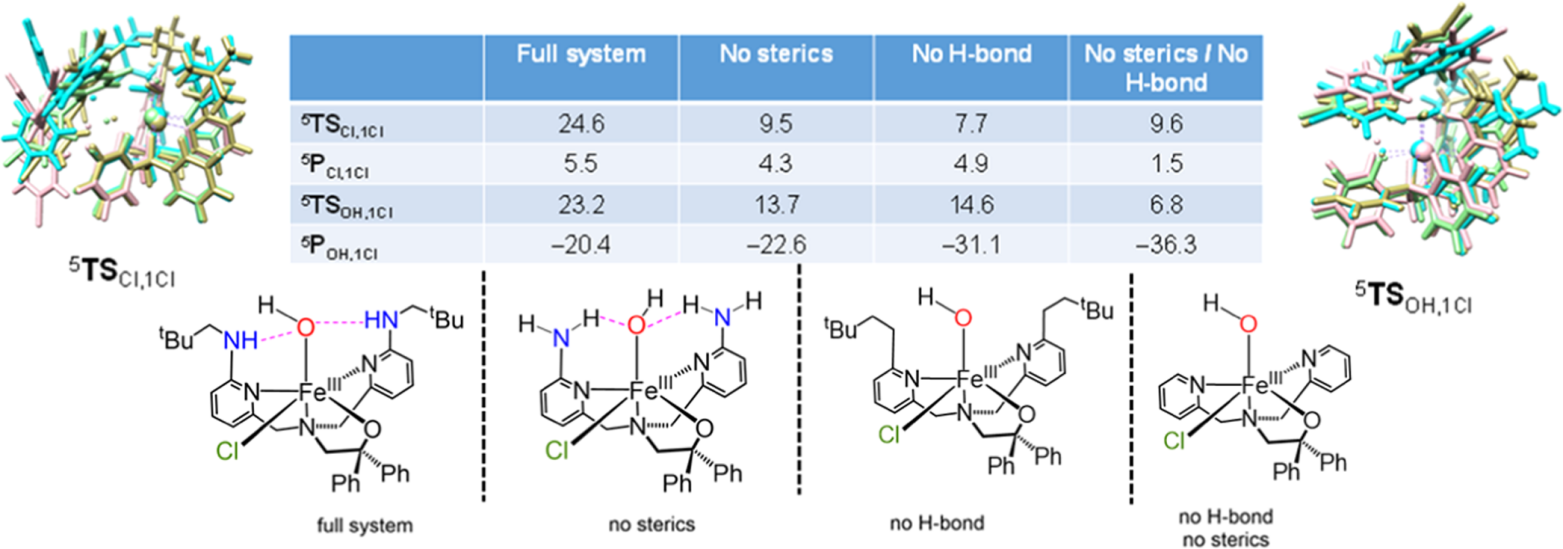
OH and Cl rebound barriers from ^5^**Re**_1Cl_ and truncated models with steric or hydrogen-bonding interactions (or both) removed. Energies obtained at UB3LYP/BS2//UB3LYP/BS1 with zero-point and solvent corrections included. Data in kcal mol^–1^ with respect to the reactant complexes. Also shown are overlays of the Cl transfer and OH transfer transition states with the following color coding: full system (khaki), without sterics (pink), without H-bond (cyan), and without sterics/H-bond (light green).

Second-coordination sphere effects on the relative energies of OH versus Cl rebound were examined with a set of calculations on models of ^5^**Re**_1Cl_ in which the second-coordination sphere substituents were systematically modified. The neopentyl substituents of the amine groups were replaced with a hydrogen atom to give the “no sterics” model (model NS). The amine groups were replaced by CH_2_ groups to give the “no H-bonds” model (model NH). The combined effects of removing the steric and hydrogen-bonding interactions were examined in a minimal model in which only hydrogen atoms were included on the periphery of pyridine rings, giving the “neither” model (model NE). The reactants, products, and transition states for OH and Cl-transfer were reoptimized for these modified models. The barrier heights with respect to the truncated reactants complexes are shown in Figure 6, while individual structures are given in Figure S3.

As discussed above, the full model has the OH rebound barrier as a highly exothermic OH transfer, while the Cl transfer is endothermic and inaccessible. This is the same for the product complexes with sterics and/or H-bonding removed. In addition, removal of the neopentyl substituents to the amine groups lowers both OH rebound and Cl-transfer barriers dramatically and makes ^5^**TS**_Cl,1Cl_ the lowest in energy by 4.2 kcal mol^–1^. A similar observation is found for removal of the hydrogen-bonding interactions from the ligand scaffold, and ^5^**TS**_CL,1Cl_ becomes the lowest transition state by 6.9 kcal mol^–1^. Finally, the removal of all second-coordination sphere perturbations makes the OH rebound barrier the lowest in energy by 2.8 kcal mol^–1^. As such, the halogen-transfer barrier of the three truncated models are close in energy, while a major shift in the barrier is seen for the OH rebound barriers. These calculations show that second-coordination sphere perturbations affect the bifurcation pathways dramatically, leading to hydroxylation or halogenation. Thus, the minimal cluster model with all second-coordination sphere perturbations removed will follow the Bell–Evans–Polanyi principle and give a low barrier and large exothermicity for hydroxyl transfer and a much higher barrier and lesser exothermicity for halogen transfer.^94,95^ The calculations in Figure 6 also show that the Bell–Evans–Polanyi principle can be overruled by second-coordination sphere effects such as hydrogen-bonding interactions to the hydroxo group or steric interactions restricting the substrate approach. This is in line with calculations reported on nonheme iron halogenase enzymes that reported strong second-coordination sphere effects on the bifurcation pathways of halogenation versus hydroxylation.^32,35,36,40^ Interestingly, the combined perturbations do not favor halogenation but still give hydroxylation, although by a smaller amount than with steric and H-bonding perturbations removed. Clearly, the effect of sterics and hydrogen-bonding interactions restricts and affects the halogen transfer as well. Furthermore, it appears that the combined effect of sterics and H-bonding removal almost linearly affects the hydroxyl transfer, but not the halogen transfer. This is most likely as a result of the fact that we did full geometry optimizations with these perturbations removed. As a consequence, the changes in geometry lead to differences in oxidant–substrate interactions that strongly affect the halogen-transfer pathway.

On the far left and far right-hand side of Figure 6 are given overlays of the OH and Cl-transfer transition states for the reaction of (*p*-Cl–C_6_H_4_)_3_C^•^ with **1**_Cl_ and truncated models, while Figure S3 gives the individual structures. The four OH rebound barriers are geometrically very similar, and an overlay puts the substrate in approximately the same position in the four transition states. The effect of second-coordination sphere effects on the OH rebound is as expected and leads to a small barrier for the fully truncated model, an increase in the barrier with either the hydrogen bonding or steric perturbations added and a further increase of the barriers with both effects included.

Although the halogen atom that is in the equatorial plane is not directly in contact with the hydrogen-bond donors and steric groups, actually the approach on the halide by a large and bulky substrate such as (*p*-Cl–C_6_H_4_)_3_C^•^ is influenced by the removal of steric and hydrogen-bonding perturbations. Indeed, the position of the substrate in the overlay of the four transition-state structures for chlorine transfer shows the halide and substrate in very different positions in the four transition states. As such, the halide transfer is influenced by these perturbations. Removal of one of the perturbations, therefore, has a much more dramatic effect on the barrier height of Cl transfer than OH transfer. The results displayed in Figure 6 show that the second-coordination sphere has a major effect on the halogenation versus hydroxylation selectivity and, particularly, the use of a bulky substrate like (*p*-Cl–C_6_H_4_)_3_C^•^ may struggle to optimally reach the halide and form a chemical bond. This is also seen in the dichloro compound **2**_Cl_ above (Figure 4), where the equatorial Cl group is easier to transfer than the distal Cl group by 10.2 kcal mol^–1^ due to lesser steric interactions for equatorial rather than distal approach.

Halogen transfer for Cl/Br appears to be an endothermic, reversible process with the tertiary carbon radical substrates even in the systems with steric and H-bonding interactions removed. These results are consistent with the relatively weak C–X bonds formed in the halogenated products. Moreover, the experimental data shows only hydroxylation products for the 3° carbon radical substrates. As the removal of steric and H-bonding interactions from the second-coordination sphere does not lead to halogen transfer, we hypothesized that calculations on 2° carbon radicals may reveal an exothermic process that favored halogen transfer.

**Figure 7:**
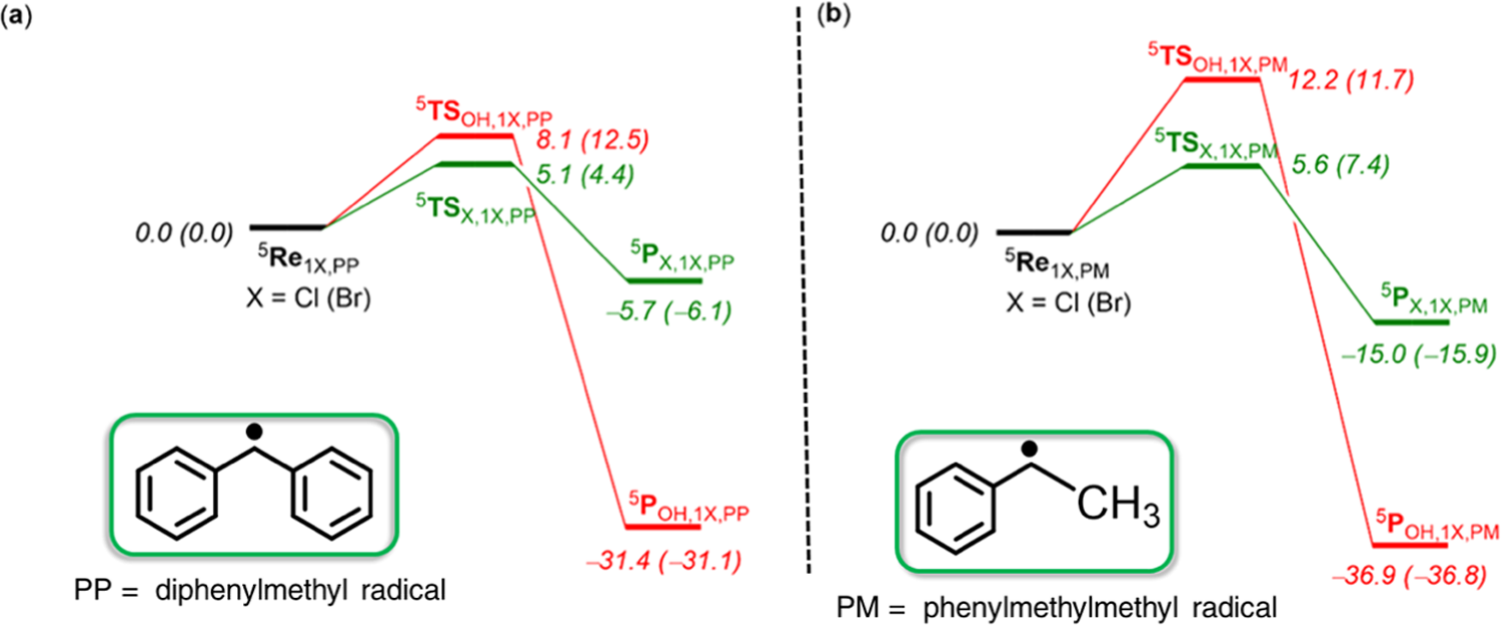
Potential energy landscape for halogen versus hydroxyl transfer from complexes **Re**_1Cl_/**Re**_1Br_ to the secondary radicals (C_6_H_5_)_2_CH^•^ (PP) and (C_6_H_5_)(CH_3_)CH^•^ (PM) as calculated at UB3LYP/BS2//UB3LYP/BS1 level of theory with the solvent and zero-point corrections included. Energies are in kcal mol^–1^. Values in parentheses are for the reaction from **Re**_1Br_.

Reactions with the 2° carbon radicals diphenylmethyl (C_6_H_5_)_2_CH^•^ and phenylmethylmethyl (C_6_H_5_)(CH_3_)CH^•^ were examined by DFT calculations. The potential energy landscapes for the reactions of ^6^**1**_Cl_ and ^6^**1**_Br_ with these two substrates are shown in Figure 7. Both substrates react with substantially lower halogen- and hydroxyl-transfer barriers as compared to the 3° carbon radical (*p*-Cl–C_6_H_4_)_3_C^•^, and halogen-transfer barriers are lower in energy than the hydroxyl-transfer reactions. For the diphenylmethyl radical reaction the energy of ^5^**TS**_Cl,1Cl,PP_ is 5.1 kcal mol^–1^ above ^5^**Re**_1Cl,PP_, while the ^5^**TS**_OH,1Cl,PP_ barrier is at 8.1 kcal mol^–1^. A similar pattern for this substrate with ^6^**1**_Br_ is seen with barriers for ^5^**TS**_Br,1Br,PP_ and ^5^**TS**_OH,1Br,PP_ of 4.4 and 12.5 kcal mol^–1^, respectively. The same ordering and trends are seen for (C_6_H_5_)(CH_3_)CH^•^ as a substrate (Figure 7b). These barriers are sufficiently low that they will not compete with the isomerization pathway through flipping of the positions of the hydroxyl and halide groups. Moreover, the results predict chemoselective halogen transfer over hydroxyl transfer for the *cis*-Fe^III^(OH)(X) complexes and the 2° carbon radical substrates. They also predict exothermic processes for halogen transfer as anticipated, making the reverse reactions unlikely in these cases. Taken together, the calculations are in good agreement with the experimental results in Figure 1, and indicate that biomimetic *cis*-Fe^III^(OH)(X) complexes are able to exhibit halogenase-like activity with appropriate carbon radical substrates.

**Figure 8:**
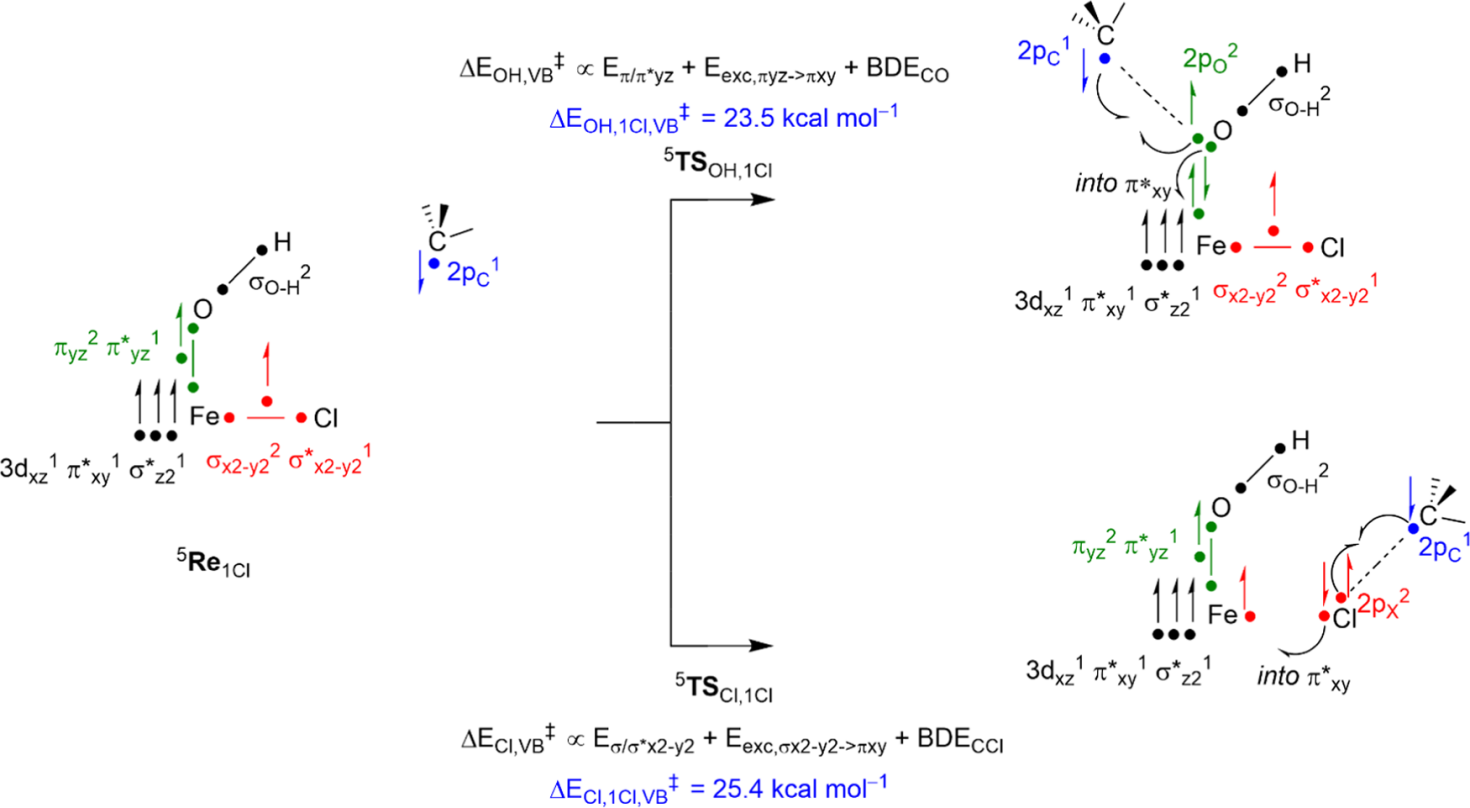
Valence bond diagram for the prediction of OH and Cl-transfer barriers in ^5^**Re**_1Cl_. Dots are electrons and a bar between two dots is a chemical bond occupied with two electrons.

To rationalize the bifurcation pathways and energies and gain insight into the intrinsic properties of the oxidant and substrate that determine the selectivity, we created a valence bond (VB) diagram for C–Cl versus C–OH bond-formation reaction channels, as described in Figure 8. We have used these VB diagrams in a number of cases previously to predict a series of hydrogen atom abstraction and double-bond epoxidation reactions as well as for the bifurcation pathways of desaturation versus hydroxylation and group transfer reactions.^96−98^ Thus, we analyze all molecular orbitals in the reactant and product complexes and determine which orbitals break or form during each reaction pathway. The energies of the breaking and forming of the orbitals that determine the height of the barrier for the reaction are estimated. In the OH transfer reaction, the Fe–O bond is along the molecular *z*-axis and is based on the three-electron bond resulting from two electrons in the π*_yz_* orbital and a single electron in π*_yz_. However, when these orbitals break during the OH transfer reaction, one of the electrons pairs up with the incoming radical to form the C–O bond (the σ_CO_ orbital), the second one becomes the 3d*_yz_* orbital on iron with one electron, and the third electron is promoted into the π*_xy_ orbital. Based on the orbital energies in the reactant structures, we estimated the energy to break the π*_yz_*/π*_yz_ pair of orbitals into atomic orbitals (*E*_π/π*yz_) from the energy gap between the π*_yz_* and π*_yz_ orbitals. We estimated the π*_yz_ → π*_xy_ excitation energy (*E*_exc, π*yz* → π*xy*_) from their orbital energy differences in the reactant complexes. In addition, the OH rebound barrier depends on the bond dissociation energy of the C–O bond (BDE_CO_) that is formed and the driving force for the reaction. The driving force was determined from the difference in energy of the Fe–Cl and C–Cl bonds (halogen transfer) or the difference in energy of the Fe–OH and C–OH bonds (OH transfer) in the reactant and product complexes. As iron complexes sometimes undergo drastic geometric changes when a ligand is removed, we calculated diabatic bond energies for breaking of the Fe–Cl and Fe–OH bonds in ^6^**Re**_1Cl_ by doing single-point calculations on each of the individual fragments. The bond dissociation energies (BDE) for the C–O bond in the alcohol complex and the C–Cl bond in the halogenated products were determined from a DFT calculation on the isolated products and the optimized geometries of the product with Cl/OH removed. Subsequently, we used the procedure from ref (81) to estimate the OH rebound barriers and summarized in eq 1, whereby the driving force for the reaction is given as Δ*E*_rp_ and the resonance energy *B* taken is one-half of the weakest bond that is broken or formed.
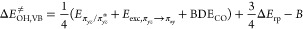
1

In a similar way to the OH rebound barriers, we also estimated the Cl-transfer barriers using our VB approach, see eq 2. As the Fe–Cl bond in the reactant complex is in the equatorial plane, the bond cleavage is not dependent on the π_yz_/π*_yz_ pair of orbitals but on the three-electron bond in the equatorial plane with occupation σ_*x*2–*y*2_^2^ σ*_x2–*y*2_^1^. We determined the energy gap (*E*_σ*x*2–*y*2/σ*x2–*y*2_) from the orbital energy differences in the reactant complex as well as the excitation energy from σ_*x*2–*y*2_ to π*_xy_.
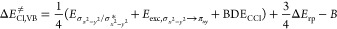
2Based on these electronic values in the reactant and product complexes, we estimated the Cl ad OH transfer barriers with our VB approach and found values of Δ*E*_Cl,1Cl,VB_^‡^ = 25.4 kcal mol^–1^ and Δ*E*_OH,1Cl,VB_^‡^ = 23.5 kcal mol^–1^. These values are in excellent agreement with the DFT calculated barriers in Figure 3 for the same processes. Thus, even though a much stronger C–O bond is formed in the (*p*-Cl–C_6_H_4_)_3_COH product complex than in the (*p*-Cl–C_6_H_4_)_3_CCl complex, i.e., BDE_CO_ = 59.2 kcal mol^–1^ and BDE_CCl_ = 46.2 kcal mol^–1^, this advantage is canceled out by a similar difference of Fe–O versus Fe–Cl bond strengths. The homolytic bond energies of the C–O and C–Cl bonds in the possible product complexes are summarized in Table 1. Furthermore, our diabatic bond energies for the ^5^**Re**_1Cl_ complex are BDE_FeO_ = 75.0 kcal mol^–1^ and BDE_FeCl_ = 63.8 kcal mol^–1^. These bond strengths follow the same ordering as those of an [Fe^III^(OH)(Cl)(TPA)] complex with TPA = tris(2-pyridylmethyl)amine, although the diabatic bond strengths were much weaker.^80^ Therefore, the hydrogen bonding and steric interactions strengthen the Fe–OH bond in **1**_Cl_ by about 10 kcal mol^–1^. Moreover, the three-electron bond for the interaction of the σ_*x*2–*y*2_ and σ*_x2–*y*2_ orbitals appears weaker than the π*_yz_*/π*_yz_ interaction, and we find the α-orbitals separated by 88.8 and 99.2 kcal mol^–1^. Therefore, a weak halogen ligand in the equatorial plane affects the orbitals in the *xy*-plane.

**Table 1:** UB3LYP/6-311+G* Calculated Homolytic Bond Dissociation Energies^a^^b^

reaction	F	Cl	Br	OH	OTf
(*p*-Cl–C_6_H_4_)_3_C^•^ + X^•^ → (*p*-Cl–C_6_H_4_)_3_CX	83.2	46.2	34.3	59.2	47.0
(C_6_H_5_)_2_CH^•^ + X^•^ → (C_6_H_5_)_2_CHX	91.3	55.4	43.6	67.6	56.8
(C_6_H_5_)(CH_3_)CH^•^ + X^•^ → (C_6_H_5_)(CH_3_)CHX	95.8	63.7	52.8	73.3	68.1

a Data calculated at Δ*E* + ZPE level of theory with the solvent model included.

b Values in kcal mol^–1^.

Subsequently, we took the reactant complexes of truncated models with the (*p*-Cl–C_6_H_4_)_3_C^•^ substrate without H-bonding (^5^**Re**_1Cl,NH_), with sterics removed (^5^**Re**_1Cl,NS_) and with both sterics and hydrogen bonding removed (^5^**Re**_1Cl,NE_) and repeated the VB analysis. The analysis shows that little changes in the orbital energies are found for the π*_yz_*, π*_yz_, σ_*x*2–*y*2_, and σ*_x2–*y*2_ orbitals, and therefore, the relative barrier heights for Cl and OH transfer with removal of either hydrogen bonding or steric interactions or both are only dependent on the driving forces for the reactions with the truncated complexes. Thus, for the full system **1**_Cl_, the thermodynamics for the adiabatic driving forces Δ*E*_rp_ for OH and Cl transfer were calculated and are close in energy, i.e., 15.8 and 17.6 kcal mol^–1^. However, removal of the hydrogen-bonding interactions weakens the Fe–OH bond and drops the driving force for OH transfer to −1.1 kcal mol^–1^, while removing of sterics has only a small effect and gives a driving force of 16.2 kcal mol^–1^. Consequently, removing the hydrogen-bonding interactions from **1**_Cl_ results in a major lowering of the OH transfer barriers and preferential OH rebound by a major amount. On the other hand, to obtain favorable halide transfer over OH rebound, hydrogen-bonding interactions to the hydroxyl group strengthen the Fe–O bond and slow OH release from the complex dramatically.

Next, using the VB models, we predicted barriers for OH and Cl transfer from **1**_Cl_ to (C_6_H_5_)_2_CH^•^ and (C_6_H_5_)(CH_3_)CH^•^ radicals. As shown in Table 1, these substrates in a reaction with chlorine lead to stronger C–Cl bonds of 55.4 and 63.7 kcal mol^–1^, respectively. As a result of that, the driving force for Cl transfer is lowered and the VB predicted barriers are much lower than with (*p*-Cl–C_6_H_4_)_3_C^•^ as a substrate, namely, Δ*E*_Cl,VB_^‡^ = 16.3 kcal mol^–1^ for the reaction of **1**_Cl_ with (C_6_H_5_)_2_CH^•^ and Δ*E*_Cl,VB_^‡^ = 7.9 kcal mol^–1^ for the reaction of **1**_Cl_ with (C_6_H_5_)(CH_3_)CH^•^. By contrast, the VB predicted corresponding OH rebound barriers for these complexes are 15.1 and 9.4 kcal mol^–1^, respectively. These calculations indeed show that halogen transfer becomes competitive with OH rebound for secondary radicals as the difference in energy between the C–Cl and C–OH bonds is smaller and hence the driving force becomes exothermic. In particular, the VB model as well as the DFT calculations predicts a phenylmethylmethyl radical to react with **1**_Cl_ through dominant halide transfer in agreement with experimental observation.

## Conclusions

The successful synthesis and structural characterization of the nonheme iron complexes Fe^III^(BNPA^Ph2^O)(OH)(X) (X = Cl, Br) seemed to provide an ideal system to test the preferential reactivity of *cis*-Fe^III^(OH)(X) species and carbon radicals, with the aim of modeling the critical rebound step in the nonheme iron halogenases. However, the initial study showed only evidence for hydroxyl transfer to tertiary carbon radicals and no evidence of halogenation.^53^ These results fueled speculation regarding what key characteristics might separate our system from the enzymes themselves. Although the lack of an enzyme pocket was an obvious difference, there were a few examples of halogenation mediated by similar nonheme iron complexes that lacked substrate binding pockets.^42,47,99^ In addition, several computational studies indicated that there was likely an inherent reactivity preference for halogenation over hydroxylation, in contradiction to the Bell–Evans–Polanyi principle, which predicts that the thermodynamically favored hydroxylation process should be kinetically preferred as well.^35,38,80^

The computational analysis reported in the current study provides a satisfying explanation for the observed reactivity of the tertiary carbon radicals. It predicts a nonproductive equilibrium for the halogenation (X = Cl, Br but not F) pathway, which drives the outcome of the reaction toward the more thermodynamically stable, hydroxylated product. We were pleased to find that this prediction was experimentally verified by showing that back electron transfer occurs between Fe^II^(BNPA^Ph2^O)(OH) and the alkyl halide (*p*-Cl–C_6_H_4_)_3_C–Br to give the Fe^III^(OH)(Br) complex. The calculations also showed that halogenation of an appropriate, secondary carbon radical substrate should not be affected by the same nonproductive equilibrium, at least in part because of the significantly stronger C–Cl bond to be formed. These calculations also nicely fit the experimental data: the secondary carbon radical (C_6_H_5_)(CH_3_)CH^•^ reacts to give a halogenated product, showing for the first time that an isolated *cis*-Fe^III^(OH)(X) complex can selectively transfer the *cis-*halogen ligand to a carbon radical partner.

The nonheme iron halogenases typically react with substrates tethered to carrier proteins, such as seen in SyrB2. This type of fixed substrate precludes the loss of inherent selectivity for halogenation that might ensue if the enzyme was to operate on different, freestanding substrates. However, a relatively new member of the halogenase family, WelO5, does halogenate a number of freestanding substrates. A recent computational study suggests that controlling the conformation of the active center is crucial for halogenation selectivity in WelO5, where a broad substrate scope is required.^100^ In contrast, the substrate in the classical halogenase SyrB2 can provide much of its own, inherent selectivity to favor halogenation. The combined experimental and computational results presented here emphasize the importance of the immediate structure of the radical carbon atom in controlling selectivity. In particular, our work highlights that it will be unlikely for nonheme iron halogenases to activate tertiary C–H bonds, and it would be interesting if this point could be confirmed experimentally. These results also indicate that substrate structure needs to be carefully considered in the design of any future enzymatic or synthetic halogenation catalysts. The computational results predict that fluorine transfer to aliphatic C–H bonds may be favored for a wide range of carbon radical substrates; however, the iron(III)–hydroxo(fluoro)complex has eluded synthetic isolation thus far and this idea requires future testing.

## Supporting Information Available

All data generated during this study are provided in the Supporting Information. Tables with calculated absolute and relative energies and group spin densities and charges of all complexes discussed in this work; Cartesian coordinates of all structures (PDF)
