# Supplementary material for: What Drives Radical Halogenation versus Hydroxylation in Mononuclear Nonheme Iron Complexes? A Combined Experimental and Computational Study
Source: J Am Chem Soc. 2022 May 10;144(24):10752–67. doi: 10.1021/jacs.2c01375 (PMC9228086; doi:10.1021/jacs.2c01375)
Supplement: Supplementary file 1 — ja2c01375_si_001.pdf [file ja2c01375_si_001.pdf]

Supporting Information

What Drives Radical Halogenation Versus Hydroxylation in Mononuclear Nonheme Iron Complexes? A Combined Experimental and Computational Study

Emilie F. Gérard,[§⊥‡] Vishal Yadav,[&‡] David P. Goldberg*[&] and Sam P. de Visser*[§⊥]

[§] Manchester Institute of Biotechnology, The University of Manchester, 131 Princess Street, Manchester M1 7DN, United Kingdom

[⊥] Department of Chemical Engineering, The University of Manchester, Oxford Road, Manchester M13 9PL, United Kingdom

[&] Department of Chemistry, The Johns Hopkins University, 3400 North Charles Street, Baltimore, Maryland 21218, United States.

Email: sam.devisser@manchester.ac.uk (SPdV); dpg@jhu.edu (DPG).

Computational Data:

Table S1. Absolute energies, zero-point energies and free energies (in a.u.) of optimized geometries for the reaction of $[Fe^{III}(BNPA^{Ph2}O)(OH)(Cl)]$ (**1**$_{Cl}$) with the radical substrate (p-Cl-C_6H_4)$_3$C• as obtained in Gaussian-09 in the quintet spin state. All calculations were done with a continuum polarized conductor model (CPCM) included with the solvent THF that has a dielectric constant of 7.6.

	E [au]	ZPE [au]	G [au]	E [au]
	B3LYP/BS1	B3LYP/BS1	B3LYP/BS1	B3LYP/BS2
$^6[Fe^{III}(BNPA^{Ph2}O)(OH)(Cl)]$	-2407.708188	0.78465	-2407.003296	-2403.445439
$^4[Fe^{III}(BNPA^{Ph2}O)(OH)(Cl)]$	-2407.697646	0.78595	-2406.990971	-
$^2[Fe^{III}(BNPA^{Ph2}O)(OH)(Cl)]$	-2407.692934	0.78682	-2406.983804	-
(p-Cl-C_6H_4)$_3$-C•	-2111.594199	0.25193	-2111.391251	-2112.063601
^{5}**Re**$_{1Cl}$	-4519.316207	1.036712	-4518.392554	-4515.545298
^{3}**Re**$_{1Cl}$	-4519.302823	1.038072	-4518.374585	-
^{7}**Re**$_{1Cl}$	-4519.316206	1.036714	-4518.392855	-
^{5}**TS**$_{Cl,1Cl}$	-4519.288773	1.035939	-4518.356487	-4515.505285
^{5}**Pr**$_{Cl,1Cl}$	-4519.296217	1.035849	-4518.372350	-4515.535718
^{5}**TS**$_{OH,1Cl}$	-4519.272426	1.037323	-4518.341912	-4515.508975
^{5}**Pr**$_{OH,1Cl}$	-4519.331573	1.039142	-4518.405548	-4515.580205
^{3}**TS**$_{OH,1Cl}$	-4519.248011	1.039075	-4518.313034	-4515.458101
^{3}**Pr**$_{OH,1Cl}$	-4519.304936	1.040375	-4518.375100	-4515.535061
^{3}**Pr**$_{Cl,1Cl}$	-4519.274651	1.037250	-4518.348422	-4515.484995

Table S2. Relative energies, zero-point energies and free energies (in kcal mol^{-1}) of optimized geometries for the reaction of $[Fe^{III}(BNPA^{Ph2}O)(OH)(Cl)]$ (**1**$_{Cl}$) with the radical substrate (p-Cl-C_6H_4)$_3$C• as obtained in Gaussian-09 in the quintet spin state.

	ΔE	ΔE+ZPE	ΔG	ΔE	ΔE+ZPE	ΔG
	BS1	BS1	BS1	BS2	BS2	BS2
$^6[Fe^{III}(BNPA^{Ph2}O)(OH)(Cl)]$	0.00	0.00	0.00	0.00	0.00	0.00
$^4[Fe^{III}(BNPA^{Ph2}O)(OH)(Cl)]$	6.61	7.43	7.73	-	-	-
$^2[Fe^{III}(BNPA^{Ph2}O)(OH)(Cl)]$	9.57	10.93	12.23	-	-	-
^{5}**Re**$_{1Cl}$	0.00	0.00	0.00	0.00	0.00	0.00
^{3}**Re**$_{1Cl}$	8.40	9.25	11.28	-	-	-
^{7}**Re**$_{1Cl}$	0.00	0.00	-0.19	-	-	-
^{5}**TS**$_{Cl,1Cl}$	17.22	16.73	22.63	25.11	24.62	30.53
^{5}**Pr**$_{Cl,1Cl}$	12.54	12.00	12.68	6.01	5.47	6.15
^{5}**TS**$_{OH,1Cl}$	27.47	27.86	31.78	22.79	23.18	27.10
^{5}**Pr**$_{OH,1Cl}$	-9.64	-8.12	-8.15	-21.90	-20.38	-20.42
^{3}**TS**$_{OH,1Cl}$	42.79	44.28	49.90	54.72	56.20	61.82
^{3}**Pr**$_{OH,1Cl}$	7.07	9.37	10.95	6.42	8.72	10.30
^{3}**Pr**$_{Cl,1Cl}$	26.08	26.41	27.69	37.84	38.18	39.46

Table S3. Group spin densities of UB3LYP/BS1+solvent optimized geometries for the reaction pathway of [Fe^{III}($BNPA^{Ph2}$O)(OH)(Cl)] (**1**$_{Cl}$) with the radical substrate (*p*-Cl-C_6H_4)$_3$C• as obtained in Gaussian-09 in the quintet spin state.

	ρ_{Fe}	ρ_{ligand}	ρ_{OH}	ρ_{Cl}	ρ_{Trityl}
^{5}**Re**$_{1Cl}$	4.02	0.65	0.19	0.14	-1.00
^{5}**TS**$_{Cl,1Cl}$	3.95	0.52	0.17	0.11	-0.75
^{5}**Pr**$_{Cl,1Cl}$	3.69	0.24	0.07	0.00	0.00
^{5}**TS**$_{OH,1Cl}$	3.79	0.30	0.12	0.06	-0.27
^{5}**Pr**$_{OH,1Cl}$	3.68	0.29	0.00	0.03	0.00

Table S4. Absolute energies, zero-point energies and free energies (in a.u.) of optimized geometries for the reaction of $[Fe^{III}(BNPA^{Ph2}O)(OH)(Br)]$ (**1**$_{Br}$) with the radical substrate (p-Cl-$C_6H_4)_3C$• as obtained in Gaussian-09 in the quintet spin state. All calculations were done with a continuum polarized conductor model (CPCM) included with the solvent THF that has a dielectric constant of 7.6.

	E [au]	ZPE [au]	G [au]	E_{solv} [au]
	B3LYP/BS1	B3LYP/BS1	B3LYP/BS1	B3LYP/BS2
$[Fe^{III}(BNPA^{Ph2}O)(OH)(Br)]$	-4518.908211	0.784449	-4518.203936	-4517.354653
(p-Cl-$C_6H_4)_3$-C•	-2111.594199	0.251930	-2111.391251	-2112.063601
^{5}**Re**$_{1Br}$	-6630.516109	1.036479	-6629.594789	-6629.465928
^{5}**TS**$_{Br,1Br}$	-6630.492889	1.035075	-6629.569810	-6629.443573
^{5}**Pr**$_{Br,1Br}$	-6630.499814	1.035645	-6629.578175	-6629.455477
^{5}**TS**$_{OH,1Br}$	-6630.472378	1.037407	-6629.540904	-6629.429199
^{5}**Pr**$_{OH,1Br}$	-6630.532807	1.038817	-6629.608811	-6629.499173
^{3}**Re**$_{1Br}$	-6630.504381	1.037678	-6629.581199	-
^{3}**TS**$_{OH,1Br}$	-6630.447891	1.039140	-6629.512545	-
^{3}**TS**$_{Br,1Br}$	-6630.473500	1.036114	-6629.549958	-6629.390046
^{3}**Pr**$_{Br,1Br}$	-6630.478697	1.036852	-6629.553925	-6629.404881

Table S5. Relative energies, zero-point energies and free energies (in kcal mol^{-1}) of optimized geometries for the reaction of $[Fe^{III}(BNPA^{Ph2}O)(OH)(Br)]$ (**1**$_{Br}$) with the radical substrate (p-Cl-$C_6H_4)_3C$• as obtained in Gaussian-09 in the quintet spin state.

	ΔE	ΔE+ZPE	ΔG	ΔE	ΔE+ZPE	ΔG
	BS1	BS1	BS1	BS2	BS2	BS2
^{5}**Re**$_{1Br}$	0.00	0.00	0.00	0.00	0.00	0.00
^{5}**TS**$_{Br,1Br}$	14.57	13.69	15.67	14.03	13.15	15.13
^{5}**Pr**$_{Br,1Br}$	10.23	9.70	10.43	6.56	6.03	6.76
^{5}**TS**$_{OH,1Br}$	27.44	28.02	33.81	23.05	23.63	29.42
^{5}**Pr**$_{OH,1Br}$	-10.48	-9.01	-8.80	-20.86	-19.39	-19.18
^{3}**Re**$_{1Br}$	7.36	8.11	8.53	-	-	-
^{3}**TS**$_{OH,1Br}$	42.81	44.48	51.61	-	-	-
^{3}**TS**$_{Br,1Br}$	26.74	26.51	28.13	47.62	47.39	49.01
^{3}**Pr**$_{Br,1Br}$	23.48	23.71	25.64	38.31	38.54	40.47

Table S6. Group spin densities of UB3LYP/BS1+solvent optimized geometries for the reaction pathway of $[Fe^{III}(BNPA^{Ph2}O)(OH)(Br)]$ (**1**$_{Br}$) with the radical substrate (p-Cl-$C_6H_4)_3C$• as obtained in Gaussian-09 in the quintet spin state.

	ρ_{Fe}	ρ_{ligand}	ρ_{OH}	ρ_{Br}	ρ_{Trityl}
^{5}**Re**$_{1Br}$	4.00	0.65	0.19	0.15	-1.00
^{5}**TS**$_{Br,1Br}$	3.79	0.32	0.10	0.06	-0.27
^{5}**Pr**$_{Br,1Br}$	3.70	0.24	0.07	0.00	0.00
^{5}**TS**$_{OH,1Br}$	3.78	0.30	0.12	0.06	-0.25
^{5}**Pr**$_{OH,1Br}$	3.68	0.29	0.00	0.03	0.00

Table S7. Absolute energies, zero-point energies and free energies (in a.u.) of optimized geometries for the reaction of [Fe^{III}($BNPA^{Ph2}$O)(OH)(F)] ($\mathbf{1}_F$) with the radical substrate (*p*-Cl-C_6H_4)$_3$C• as obtained in Gaussian-09 in the quintet spin state. All calculations were done with a continuum polarized conductor model (CPCM) included with the solvent THF that has a dielectric constant of 7.6.

	E [au]	ZPE [au]	G [au]	E_{solv} [au]
	B3LYP/BS1	B3LYP/BS1	B3LYP/BS1	B3LYP/BS2
[Fe^{III}($BNPA^{Ph2}$O)(OH)(F)]	-2047.333087	0.785089	-2046.627815	-2043.058184
(*p*-Cl-C_6H_4)$_3$-C•	-2111.594199	0.251930	-2111.391251	-2112.063601
^{5}**Re**$_{1F}$	-4158.945642	1.037465	-4158.016452	-4155.158682
^{3}**Re**$_{1F}$	-4158.934229	1.040304	-4158.001853	-
^{5}**TS**$_{F,1F}$	-4158.918480	1.036512	-4157.987208	-4155.152980
^{5}**Pr**$_{F,1F}$	-4158.943593	1.037417	-4158.018410	-4155.189953
^{5}**TS**$_{OH,1F}$	-4158.895274	1.038019	-4157.962729	-4155.135486
^{5}**Pr**$_{OH,1F}$	-4158.976543	1.041215	-4158.042794	-4155.219276

Table S8. Relative energies, zero-point energies and free energies (in kcal mol^{-1}) of optimized geometries for the reaction of [Fe^{III}($BNPA^{Ph2}$O)(OH)(F)] ($\mathbf{1}_F$) with the radical substrate (*p*-Cl-C_6H_4)$_3$C• as obtained in Gaussian-09 in the quintet spin state.

	ΔE	ΔE+ZPE	ΔG	ΔE	ΔE+ZPE	ΔG
	BS1	BS1	BS1	BS2	BS2	BS2
^{5}**Re**$_{1F}$	0.00	0.00	0.00	0.00	0.00	0.00
^{3}**Re**$_{1F}$	7.16	8.94	9.16	-	-	-
^{5}**TS**$_{F,1F}$	17.04	16.45	18.35	3.58	2.98	4.88
^{5}**Pr**$_{F,1F}$	1.29	1.26	-1.23	-19.62	-19.65	-22.14
^{5}**TS**$_{OH,1F}$	31.61	31.95	33.71	14.56	14.90	16.66
^{5}**Pr**$_{OH,1F}$	-19.39	-17.04	-16.53	-38.02	-35.67	-35.16

Table S9. Group spin densities of UB3LYP/BS1+solvent optimized geometries for the reaction pathway of [Fe^{III}($BNPA^{Ph2}$O)(OH)(F)] ($\mathbf{1}_F$) with the radical substrate (*p*-Cl-C_6H_4)$_3$C• as obtained in Gaussian-09 in the quintet spin state.

	ρ_{Fe}	ρ_{ligand}	ρ_{OH}	ρ_{F}	ρ_{Trityl}
^{5}**Re**$_{1F}$	4.06	0.59	0.17	0.18	-1.00
^{5}**TS**$_{F,1F}$	3.86	0.33	0.09	0.07	-0.35
^{5}**Pr**$_{F,1F}$	3.70	0.24	0.06	0.00	0.00
^{5}**TS**$_{OH,1F}$	3.87	0.31	0.12	0.10	-0.40
^{5}**Pr**$_{OH,1F}$	3.71	0.23	0.00	0.06	0.00

Table S10. Absolute energies, zero-point energies and free energies (in a.u.) of optimized geometries for the reaction of $[Fe^{III}(BNPA^{Ph2}O)(Cl)_2]$ (**2**$_{Cl}$) with the radical substrate (p-Cl-C_6H_4)$_3$C• as obtained in Gaussian-09 in the quintet spin state. All calculations were done with a continuum polarized conductor model (CPCM) included with the solvent THF that has a dielectric constant of 7.6.

	E [au]	ZPE [au]	G [au]	E_{solv} [au]
	B3LYP/BS1	B3LYP/BS1	B3LYP/BS1	B3LYP/BS2
$[Fe^{III}(BNPA^{Ph2}O)(Cl)_2]$	-2792.103183	0.773089	-2791.410716	-2787.857975
(p-Cl-C_6H_4)$_3$-C•	-2111.594199	0.251930	-2111.391251	-2112.063601
^{5}**Re**$_{2Cl}$	-4903.713585	1.024756	-4902.805019	-4899.966105
^{5}**TS**$_{Cl2,2Cl}$	-4903.707111	1.024493	-4902.794971	-4899.954113
^{5}**Pr**$_{Cl2,2Cl}$	-4903.714399	1.024648	-4902.805605	-4899.958965
^{5}**TS**$_{Cl3,2Cl}$	-4903.687458	1.023891	-4902.771050	-4899.937154
^{5}**Pr**$_{Cl3,2Cl}$	-4903.714994	1.025256	-4902.804005	-4899.958046

Table S11. Relative energies, zero-point energies and free energies (in kcal mol^{-1}) of optimized geometries for the reaction of $[Fe^{III}(BNPA^{Ph2}O)(Cl)_2]$ (**2**$_{Cl}$) with the radical substrate (p-Cl-C_6H_4)$_3$C• as obtained in Gaussian-09 in the quintet spin state.

	ΔE	ΔE+ZPE	ΔG	ΔE	ΔE+ZPE	ΔG
	BS1	BS1	BS1	BS2	BS2	BS2
^{5}**Re**$_{2Cl}$	0.00	0.00	0.00	0.00	0.00	0.00
^{5}**TS**$_{Cl2,2Cl}$	4.06	3.90	6.31	7.53	7.36	9.77
^{5}**Pr**$_{Cl2,2Cl}$	-0.51	-0.58	-0.37	4.48	4.41	4.62
^{5}**TS**$_{Cl3,2Cl}$	16.39	15.85	21.32	18.17	17.62	23.09
^{5}**Pr**$_{Cl3,2Cl}$	-0.88	-0.57	0.64	5.06	5.37	6.58

Table S12. Absolute energies, zero-point energies and free energies (in a.u.) of optimized geometries for the reaction of $[Fe^{III}(BNPA^{Ph2}O)(Cl)(OH)]$ (**2**$_{OH}$) isomer with the radical substrate (p-Cl-$C_6H_4)_3C$• as obtained in Gaussian-09 in the quintet spin state. All calculations were done with a continuum polarized conductor model (CPCM) included with the solvent THF that has a dielectric constant of 7.6.

	E [au]	ZPE [au]	G [au]	E_{solv} [au]
	B3LYP/BS1	B3LYP/BS1	B3LYP/BS1	B3LYP/BS2
$[Fe^{III}(BNPA^{Ph2}O)(Cl)_2]$	-2407.685600	0.783964	-2406.982874	-2403.424345
(p-Cl-$C_6H_4)_3$-C•	-2111.594199	0.251930	-2111.391251	-2112.063601
^{5}**Re**$_{2OH}$	-4519.296285	1.036547	-4518.372435	-4515.532779
^{5}**TS**$_{Cl,2OH}$	-4519.247782	1.035133	-4518.321116	-4515.499687
^{5}**Pr**$_{Cl,2OH}$	-4519.297923	1.036587	-4518.373219	-4515.533885
^{5}**TS**$_{OH,2OH}$	-4519.275833	1.035731	-4518.348815	-4515.512957
^{5}**Pr**$_{OH,2OH}$	-4519.331956	1.039224	-4518.403987	-4515.581839

Table S13. Relative energies, zero-point energies and free energies (in kcal mol^{-1}) of optimized geometries for the reaction of $[Fe^{III}(BNPA^{Ph2}O)(Cl)(OH)]$ (**2**$_{OH}$) isomer with the radical substrate (p-Cl-$C_6H_4)_3C$• as obtained in Gaussian-09 in the quintet spin state.

	ΔE	ΔE+ZPE	ΔG	ΔE	ΔE+ZPE	ΔG
	BS1	BS1	BS1	BS2	BS2	BS2
^{5}**Re**$_{2OH}$	0.00	0.00	0.00	0.00	0.00	0.00
^{5}**TS**$_{Cl,2OH}$	30.44	29.55	32.20	20.77	19.88	22.53
^{5}**Pr**$_{Cl,2OH}$	-1.03	-1.00	-0.49	-0.69	-0.67	-0.16
^{5}**TS**$_{OH,2OH}$	12.83	12.32	14.82	12.44	11.93	14.43
^{5}**Pr**$_{OH,2OH}$	-22.38	-20.70	-19.80	-30.79	-29.11	-28.20

Secondary-coordination sphere tests

Table S14. Absolute energies, zero-point energies and free energies (in a.u.) of optimized geometries for the reaction of $[Fe^{III}(neither)(OH)(Cl)]$ ($\mathbf{1}_{Cl,neither}$) with the radical substrate (p-Cl-C_6H_4)$_3$C• as obtained in Gaussian-09 in the quintet spin state. All calculations were done with a continuum polarized conductor model (CPCM) included with the solvent THF that has a dielectric constant of 7.6.

	E [au]	ZPE [au]	G [au]	E_{solv} [au]
	B3LYP/BS1	B3LYP/BS1	B3LYP/BS1	B3LYP/BS2
$[Fe^{III}(neither)(OH)(Cl)]$	-1903.963365	0.464642	-1903.561416	-1899.436598
(p-Cl-C_6H_4)$_3$-C•	-2111.594199	0.25193	-2111.391251	-2112.063601
$^5\mathbf{Re}_{1Cl,NE}$	-4015.571261	0.716681	-4014.950486	-4011.5491934
$^5\mathbf{TS}_{Cl,1Cl,NE}$	-4015.536506	0.716283	-4014.912953	-4011.533447
$^5\mathbf{Pr}_{Cl,1Cl,NE}$	-4015.546410	0.717103	-4014.924517	-4011.5471936
$^5\mathbf{TS}_{OH,1Cl,NE}$	-4015.550818	0.717643	-4014.92297	-4011.5393864
$^5\mathbf{Pr}_{OH,1Cl,NE}$	-4015.604067	0.720026	-4014.978789	-4011.6104239

Table S15. Relative energies, zero-point energies and free energies (in kcal mol^{-1}) of optimized geometries for the reaction of $[Fe^{III}(neither)(OH)(Cl)]$ ($\mathbf{1}_{Cl,neither}$) with the radical substrate (p-Cl-C_6H_4)$_3$C• as obtained in Gaussian-09 in the quintet spin state.

	ΔE	ΔE+ZPE	ΔG	ΔE	ΔE+ZPE	ΔG
	BS1	BS1	BS1	BS2	BS2	BS2
$^5\mathbf{Re}_{1Cl,NE}$	0.00	0.00	0.00	0.00	0.00	0.00
$^5\mathbf{TS}_{Cl,1Cl,NE}$	21.81	21.56	23.55	9.88	9.63	11.62
$^5\mathbf{Pr}_{Cl,1Cl,NE}$	15.59	15.86	16.30	1.25	1.52	1.96
$^5\mathbf{TS}_{OH,1Cl,NE}$	12.83	13.43	17.27	6.15	6.76	10.59
$^5\mathbf{Pr}_{OH,1Cl,NE}$	-20.59	-18.49	-17.76	-38.42	-36.32	-35.60

Table S16. Absolute energies, zero-point energies and free energies (in a.u.) of optimized geometries for the reaction of $[Fe^{III}(noH)(OH)(Cl)]$ ($\mathbf{1}_{Cl,noH}$) with the radical substrate (*p*-Cl-$C_6H_4)_3C$• as obtained in Gaussian-09 in the quintet spin state. All calculations were done with a continuum polarized conductor model (CPCM) included with the solvent THF that has a dielectric constant of 7.6.

	E [au]	ZPE [au]	G [au]	E_{solv} [au]
	B3LYP/BS1	B3LYP/BS1	B3LYP/BS1	B3LYP/BS2
$[Fe^{III}(noH)(OH)(Cl)]$	-2375.606417	0.806662	-2374.880938	-2371.301371
(*p*-Cl-$C_6H_4)_3$-C•	-2111.594199	0.251930	-2111.391251	-2112.063601
$^5\mathbf{Re}_{1Cl,NH}$	-4487.214416	1.058884	-4486.268432	-4483.4131793
$^5\mathbf{TS}_{Cl,1Cl,NH}$	-4487.189896	1.057917	-4486.245446	-4483.39989
$^5\mathbf{Pr}_{Cl,1Cl,NH}$	-4487.190566	1.058011	-4486.249744	-4483.4045611
$^5\mathbf{TS}_{OH,1Cl,NH}$	-4487.185154	1.058882	-4486.233926	-4483.3899695
$^5\mathbf{Pr}_{OH,1Cl,NH}$	-4487.242978	1.061592	-4486.296148	-4483.4653703

Table S17. Relative energies, zero-point energies and free energies (in kcal mol^{-1}) of optimized geometries for the reaction of $[Fe^{III}(noH)(OH)(Cl)]$ ($\mathbf{1}_{Cl,noH}$) with the radical substrate (*p*-Cl-$C_6H_4)_3C$• as obtained in Gaussian-09 in the quintet spin state.

	ΔE	ΔE+ZPE	ΔG	ΔE	ΔE+ZPE	ΔG
	BS1	BS1	BS1	BS2	BS2	BS2
$^5\mathbf{Re}_{1Cl,NH}$	0.00	0.00	0.00	0.00	0.00	0.00
$^5\mathbf{TS}_{Cl,1Cl,NH}$	15.39	14.78	14.42	8.34	7.73	7.38
$^5\mathbf{Pr}_{Cl,1Cl,NH}$	14.97	14.42	11.73	5.41	4.86	2.17
$^5\mathbf{TS}_{OH,1Cl,NH}$	18.36	18.36	21.65	14.56	14.56	17.86
$^5\mathbf{Pr}_{OH,1Cl,NH}$	-17.92	-16.22	-17.39	-32.75	-31.05	-32.22

Table S18 Absolute energies, zero-point energies and free energies (in a.u.) of optimized geometries for the reaction of [Fe^{III}(nosterics)(OH)(Cl)] (**1**$_{Cl,nosterics}$) with the radical substrate (*p*-Cl-C_6H_4)$_3$C• as obtained in Gaussian-09 in the quintet spin state. All calculations were done with a continuum polarized conductor model (CPCM) included with the solvent THF that has a dielectric constant of 7.6.

	E [au]	ZPE [au]	G [au]	E_{solv} [au]
	B3LYP/BS1	B3LYP/BS1	B3LYP/BS1	B3LYP/BS2
[Fe^{III}(nosterics)(OH)(Cl)]	-2014.683145	0.500205	-2014.245673	-2010.220686
(*p*-Cl-C_6H_4)$_3$-C•	-2111.594199	0.251930	-2111.391251	-2112.063601
^{5}**Re**$_{1Cl,NS}$	-4126.290939	0.752327	-4125.633914	-4122.340524
^{5}**TS**$_{Cl,1Cl,NS}$	-4126.265020	0.750943	-4125.610670	-4122.324083
^{5}**Pr**$_{Cl,1Cl,NS}$	-4126.271820	0.752098	-4125.615676	-4122.333430
^{5}**TS**$_{OH,1Cl,NS}$	-4126.258958	0.752482	-4125.598453	-4122.318799
^{5}**Pr**$_{OH,1Cl,NS}$	-4126.308904	0.754557	-4125.649164	-4122.378707

Table S19. Relative energies, zero-point energies and free energies (in kcal mol^{-1}) of optimized geometries for the reaction of [Fe^{III}(nosterics)(OH)(Cl)] (**1**$_{Cl,nosterics}$) with the radical substrate (*p*-Cl-C_6H_4)$_3$C• as obtained in Gaussian-09 in the quintet spin state.

	ΔE	ΔE+ZPE	ΔG	ΔE	ΔE+ZPE	ΔG
	BS1	BS1	BS1	BS2	BS2	BS2
^{5}**Re**$_{1Cl,NS}$	0.00	0.00	0.00	0.00	0.00	0.00
^{5}**TS**$_{Cl,1Cl,NS}$	16.26	15.40	14.59	10.32	9.45	8.64
^{5}**Pr**$_{Cl,1Cl,NS}$	12.00	11.85	11.44	4.45	4.31	3.90
^{5}**TS**$_{OH,1Cl,NS}$	20.07	20.17	22.25	13.63	13.73	15.82
^{5}**Pr**$_{OH,1Cl,NS}$	-11.27	-9.87	-9.57	-23.96	-22.56	-22.26

Data with phenylmethylmethyl as substrate

Table S20. Absolute energies, zero-point energies and free energies (in a.u.) of optimized geometries for the reaction of $[Fe^{III}(BNPA^{Ph2}O)(OH)(Cl)]$ (**1**$_{Cl}$) with the radical substrate phenylmethylmethyl radical• as obtained in Gaussian-09 in the quintet spin state. All calculations were done with a continuum polarized conductor model (CPCM) included with the solvent THF that has a dielectric constant of 7.6.

	E [au]	ZPE [au]	G [au]	E_{solv} [au]
	B3LYP/BS1	B3LYP/BS1	B3LYP/BS1	B3LYP/BS2
$[Fe^{III}(BNPA^{Ph2}O)(OH)(Cl)]$	-2407.708188	0.78465	-2407.003296	-2403.445439
phenylmethylmethyl•	-310.1676298	0.144248	-310.054707	-310.3039377
^{5}**Re**$_{1Cl,PM}$	-2717.877710	0.930014	-2717.040984	-2713.780381
^{5}**TS**$_{Cl,1Cl,PM}$	-2717.865782	0.928893	-2717.032417	-2713.770391
^{5}**Pr**$_{Cl,1Cl,PM}$	-2717.890568	0.931434	-2717.054175	-2713.805723
^{5}**TS**$_{OH,1Cl,PM}$	-2717.857923	0.930815	-2717.018755	-2713.761814
^{5}**Pr**$_{OH,1Cl,PM}$	-2717.914704	0.933849	-2717.078130	-2713.843038

Table S21. Relative energies, zero-point energies and free energies (in kcal mol^{-1}) of optimized geometries for the reaction of $[Fe^{III}(BNPA^{Ph2}O)(OH)(Cl)]$ (**1**$_{Cl}$) with the radical substrate phenylmethylmethyl• as obtained in Gaussian-09 in the quintet spin state.

	ΔE	ΔE+ZPE	ΔG	ΔE	ΔE+ZPE	ΔG
	BS1	BS1	BS1	BS2	BS2	BS2
^{5}**Re**$_{1Cl,PM}$	0.00	0.00	0.00	0.00	0.00	0.00
^{5}**TS**$_{Cl,1Cl,PM}$	7.48	6.78	5.38	6.27	5.57	4.16
^{5}**Pr**$_{Cl,1Cl,PM}$	-8.07	-7.18	-8.28	-15.90	-15.01	-16.11
^{5}**TS**$_{OH,1Cl,PM}$	12.42	12.92	13.95	11.65	12.15	13.18
^{5}**Pr**$_{OH,1Cl,PM}$	-23.21	-20.81	-23.31	-39.32	-36.91	-39.41

Table S22. Group spin densities of UB3LYP/BS1+solvent optimized geometries for the reaction pathway of $[Fe^{III}(BNPA^{Ph2}O)(OH)(Cl)]$ (**1**$_{Cl}$) with the radical substrate phenylmethylmethyl• as obtained in Gaussian-09 in the quintet spin state.

	ρ_{Fe}	ρ_{ligand}	ρ_{OH}	ρ_{Cl}	ρ_{Sub}
^{5}**Re**$_{1Cl,PM}$	4.03	0.64	0.20	0.12	-1.00
^{5}**TS**$_{Cl,1Cl,PM}$	3.89	0.39	0.12	0.12	-0.52
^{5}**Pr**$_{Cl,1Cl,PM}$	3.71	0.22	0.07	0.00	0.00
^{5}**TS**$_{OH,1Cl,PM}$	3.87	0.35	0.20	0.05	-0.47
^{5}**Pr**$_{OH,1Cl,PM}$	3.69	0.28	0.00	0.03	0.00

Table S23. Absolute energies, zero-point energies and free energies (in a.u.) of optimized geometries for the reaction of [Fe^{III}($BNPA^{Ph2}O$)(OH)(Br)] ($\mathbf{1}_{Br}$) with the radical substrate phenylmethylmethyl radical• as obtained in Gaussian-09 in the quintet spin state. All calculations were done with a continuum polarized conductor model (CPCM) included with the solvent THF that has a dielectric constant of 7.6.

	E [au]	ZPE [au]	G [au]	E_{solv} [au]
	B3LYP/BS1	B3LYP/BS1	B3LYP/BS1	B3LYP/BS2
[Fe^{III}($BNPA^{Ph2}O$)(OH)(Br)]	-4518.908211	0.784449	-4518.203936	-4517.354653
phenylmethylmethyl•	-310.1676298	0.144248	-310.0547070	-310.3039377
^{5}**Re**$_{1Br,PM}$	-4829.077928	0.929624	-4828.245953	-4827.701023
^{5}**TS**$_{Br,1Br,PM}$	-4829.068642	0.929155	-4828.235734	-4827.688840
^{5}**Pr**$_{Br,1Br,PM}$	-4829.093700	0.930908	-4828.257705	-4827.727647
^{5}**TS**$_{OH,1Br,PM}$	-4829.058735	0.930856	-4828.219856	-4827.683637
^{5}**Pr**$_{OH,1Br,PM}$	-4829.122312	0.935092	-4828.279630	-4827.765137

Table S24. Relative energies, zero-point energies and free energies (in kcal mol^{-1}) of optimized geometries for the reaction of [Fe^{III}($BNPA^{Ph2}O$)(OH)(Br)] ($\mathbf{1}_{Br}$) with the radical substrate phenylmethylmethyl• as obtained in Gaussian-09 in the quintet spin state.

	ΔE	ΔE+ZPE	ΔG	ΔE	ΔE+ZPE	ΔG
	BS1	BS1	BS1	BS2	BS2	BS2
^{5}**Re**$_{1Br,PM}$	0.00	0.00	0.00	0.00	0.00	0.00
^{5}**TS**$_{Br,1Br,PM}$	5.83	5.53	6.41	7.64	7.35	8.23
^{5}**Pr**$_{Br,1Br,PM}$	-9.90	-9.09	-7.37	-16.71	-15.90	-14.18
^{5}**TS**$_{OH,1Br,PM}$	12.04	12.82	16.38	10.91	11.68	15.24
^{5}**Pr**$_{OH,1Br,PM}$	-27.85	-24.42	-21.13	-40.23	-36.80	-33.51

Table S25. Group spin densities of UB3LYP/BS1+solvent optimized geometries for the reaction pathway of [Fe^{III}($BNPA^{Ph2}O$)(OH)(Br)] ($\mathbf{1}_{Br}$) with the radical substrate phenylmethylmethyl• as obtained in Gaussian-09 in the quintet spin state.

	ρ_{Fe}	ρ_{ligand}	ρ_{OH}	ρ_{Br}	ρ_{Sub}
^{5}**Re**$_{1Br,PM}$	4.00	0.65	0.19	0.15	-1.00
^{5}**TS**$_{Br,1Br,PM}$	3.88	0.43	0.14	0.17	-0.61
^{5}**Pr**$_{Br,1Br,PM}$	3.71	0.21	0.07	0.00	0.00
^{5}**TS**$_{OH,1Br,PM}$	3.85	0.35	0.20	0.06	-0.46
^{5}**Pr**$_{OH,1Br,PM}$	3.70	0.25	0.00	0.05	0.00

Data with diphenylmethyl radical

Table S26. Absolute energies, zero-point energies and free energies (in a.u.) of optimized geometries for the reaction of [Fe^{III}($BNPA^{Ph2}O$)(OH)(Cl)] (**1**$_{Cl}$) with the radical substrate diphenylmethyl radical• as obtained in Gaussian-09 in the quintet spin state. All calculations were done with a continuum polarized conductor model (CPCM) included with the solvent THF that has a dielectric constant of 7.6.

	E [au]	ZPE [au]	G [au]	E_{solv} [au]
	B3LYP/BS1	B3LYP/BS1	B3LYP/BS1	B3LYP/BS2
[Fe^{III}($BNPA^{Ph2}O$)(OH)(Cl)]	-2407.708188	0.78465	-2407.003296	-2403.445439
diphenylmethyl•	-541.1744063	0.227567	-540.985552	-541.4094898
^{5}**Re**$_{1Cl,PP}$	-2948.883370	1.012475	-2947.972891	-2944.885173
^{5}**TS**$_{Cl,1Cl,PP}$	-2948.868477	1.011967	-2947.954991	-2944.876594
^{5}**Pr**$_{Cl,1Cl,PP}$	-2948.882263	1.013182	-2947.969674	-2944.894891
^{5}**TS**$_{OH,1Cl,PP}$	-2948.858663	1.013377	-2947.940965	-2944.873106
^{5}**Pr**$_{OH,1Cl,PP}$	-2948.914165	1.015683	-2947.999095	-2944.938452

Table S27. Relative energies, zero-point energies and free energies (in kcal mol^{-1}) of optimized geometries for the reaction of [Fe^{III}($BNPA^{Ph2}O$)(OH)(Cl)] (**1**$_{Cl}$) with the radical substrate diphenylmethyl radical• as obtained in Gaussian-09 in the quintet spin state.

	ΔE	ΔE+ZPE	ΔG	ΔE	ΔE+ZPE	ΔG
	BS1	BS1	BS1	BS2	BS2	BS2
^{5}**Re**$_{1Cl,PP}$	0.00	0.00	0.00	0.00	0.00	0.00
^{5}**TS**$_{Cl,1Cl,PP}$	9.35	9.03	11.23	5.38	5.06	7.27
^{5}**Pr**$_{Cl,1Cl,PP}$	0.69	1.14	2.02	-6.10	-5.65	-4.77
^{5}**TS**$_{OH,1Cl,PP}$	15.50	16.07	20.03	7.57	8.14	12.10
^{5}**Pr**$_{OH,1Cl,PP}$	-19.32	-17.31	-16.44	-33.43	-31.42	-30.55

Table S28. Group spin densities of UB3LYP/BS1+solvent optimized geometries for the reaction pathway of [Fe^{III}($BNPA^{Ph2}O$)(OH)(Cl)] (**1**$_{Cl}$) with the radical substrate diphenylmethyl radical • as obtained in Gaussian-09 in the quintet spin state.

	ρ_{Fe}	ρ_{ligand}	ρ_{OH}	ρ_{Cl}	ρ_{Sub}
^{5}**Re**$_{1Cl,PP}$	4.02	0.66	0.19	0.12	-1.00
^{5}**TS**$_{Cl,1Cl,PP}$	3.81	0.30	0.09	0.08	-0.28
^{5}**Pr**$_{Cl,1Cl,PP}$	3.70	0.23	0.07	0.00	0.00
^{5}**TS**$_{OH,1Cl,PP}$	3.83	0.32	0.16	0.05	-0.36
^{5}**Pr**$_{OH,1Cl,PP}$	3.67	0.29	0.00	0.04	0.00

Table S29. Absolute energies, zero-point energies and free energies (in a.u.) of optimized geometries for the reaction of $[Fe^{III}(BNPA^{Ph2}O)(OH)(Br)]$ ($\mathbf{1}_{Br}$) with the radical substrate diphenylmethyl radical • as obtained in Gaussian-09 in the quintet spin state. All calculations were done with a continuum polarized conductor model (CPCM) included with the solvent THF that has a dielectric constant of 7.6.

	E [au]	ZPE [au]	G [au]	E_{solv} [au]
	B3LYP/BS1	B3LYP/BS1	B3LYP/BS1	B3LYP/BS2
$[Fe^{III}(BNPA^{Ph2}O)(OH)(Br)]$	-4518.908211	0.784449	-4518.203936	-4517.354653
diphenylmethyl•	-541.1744063	0.227567	-540.985552	-541.4094898
$^{5}\mathbf{Re}_{1Br,PP}$	-5060.084622	1.012372	-5059.176127	-5058.805782
$^{5}\mathbf{TS}_{Br,1Br,PP}$	-5060.071323	1.011613	-5059.159232	-5058.798028
$^{5}\mathbf{Pr}_{Br,1Br,PP}$	-5060.083678	1.012471	-5059.174344	-5058.815652
$^{5}\mathbf{TS}_{OH,1Br,PP}$	-5060.060239	1.012929	-5059.142741	-5058.786353
$^{5}\mathbf{Pr}_{OH,1Br,PP}$	-5060.114856	1.015434	-5059.201400	-5058.858336

Table S30. Relative energies, zero-point energies and free energies (in kcal mol^{-1}) of optimized geometries for the reaction of $[Fe^{III}(BNPA^{Ph2}O)(OH)(Br)]$ ($\mathbf{1}_{Br}$) with the radical substrate diphenyl methyl radical • as obtained in Gaussian-09 in the quintet spin state.

	ΔE	ΔE+ZPE	ΔG	ΔE	ΔE+ZPE	ΔG
	BS1	BS1	BS1	BS2	BS2	BS2
$^{5}\mathbf{Re}_{1Br,PP}$	0.00	0.00	0.00	0.00	0.00	0.00
$^{5}\mathbf{TS}_{Br,1Br,PP}$	8.35	7.87	10.60	4.87	4.39	7.12
$^{5}\mathbf{Pr}_{Br,1Br,PP}$	0.59	0.65	1.12	-6.19	-6.13	-5.67
$^{5}\mathbf{TS}_{OH,1Br,PP}$	15.30	15.65	20.95	12.19	12.54	17.84
$^{5}\mathbf{Pr}_{OH,1Br,PP}$	-18.97	-17.05	-15.86	-32.98	-31.06	-29.87

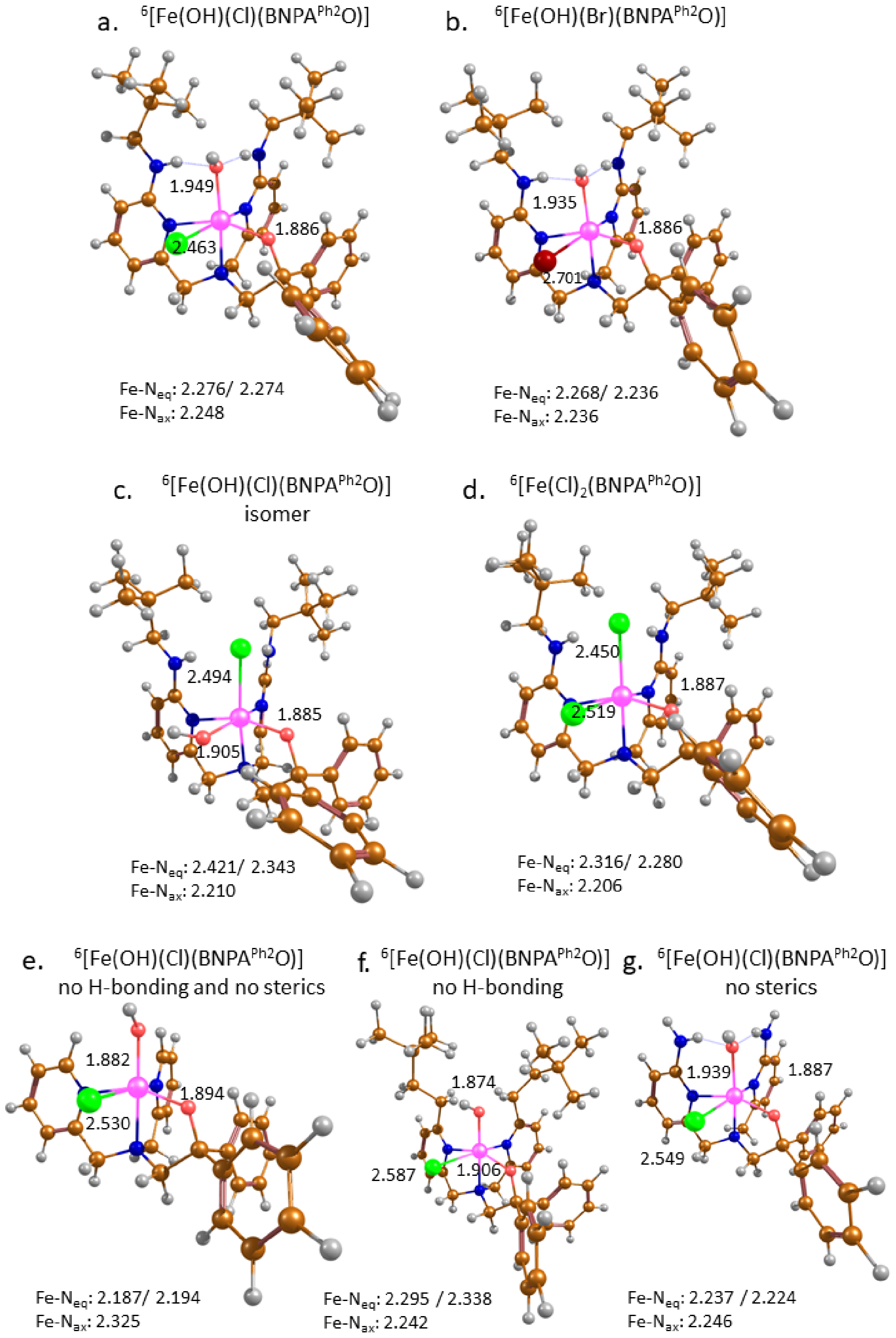

Figure S1. Optimized geometries of (a) ^{6}[Fe(OH)(Cl)(BNPA^{Ph2}O)]; (b) ^{6}[Fe(OH)(Br)(BNPA^{Ph2}O)]; (c) ^{6}[Fe(OH)(Cl)(BNPA^{Ph2}O)] isomer; (d) ^{6}[Fe(Cl)$_2$(BNPA^{Ph2}O)]; (e) ^{6}[Fe(OH)(Cl)(BNPA^{Ph2}O)] no H-bonding and no sterics; (f) ^{6}[Fe(OH)(Cl)(BNPA^{Ph2}O)] no H-bonding; (g) ^{6}[Fe(OH)(Cl)(BNPA^{Ph2}O)] no sterics, as obtained at UB3LYP/BS1 in Gaussian-09. Bond lengths are in angstroms.

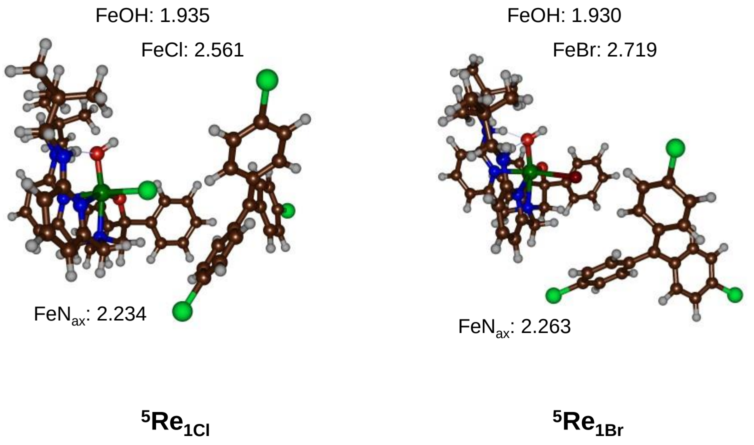

Figure S2. Optimized geometries of ^{5}**Re**$_{1Cl}$ and ^{5}**Re**$_{1Br}$ as obtained at UB3LYP/BS1 in Gaussian-09. Bond lengths are in angstroms.

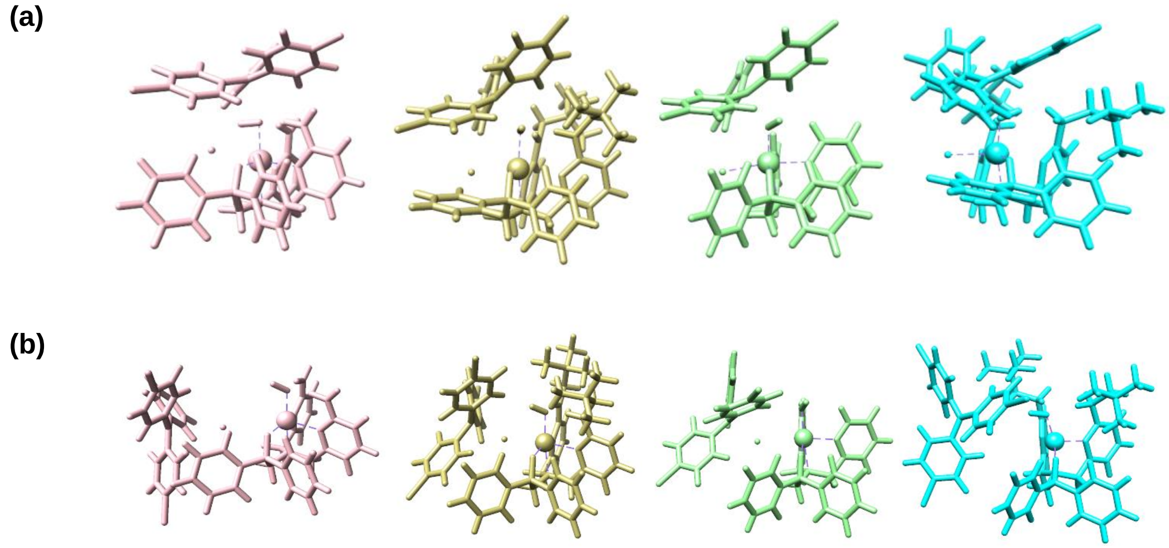

Figure S3. OH (part a) and Cl (part b) rebound barriers from ^{5}**Re**$_{1Cl}$ and truncated models with steric or hydrogen bonding interactions (or both) removed. Color coding: full system (khaki), without sterics (pink), without H-bond (cyan) and without sterics/H-bond (light green).

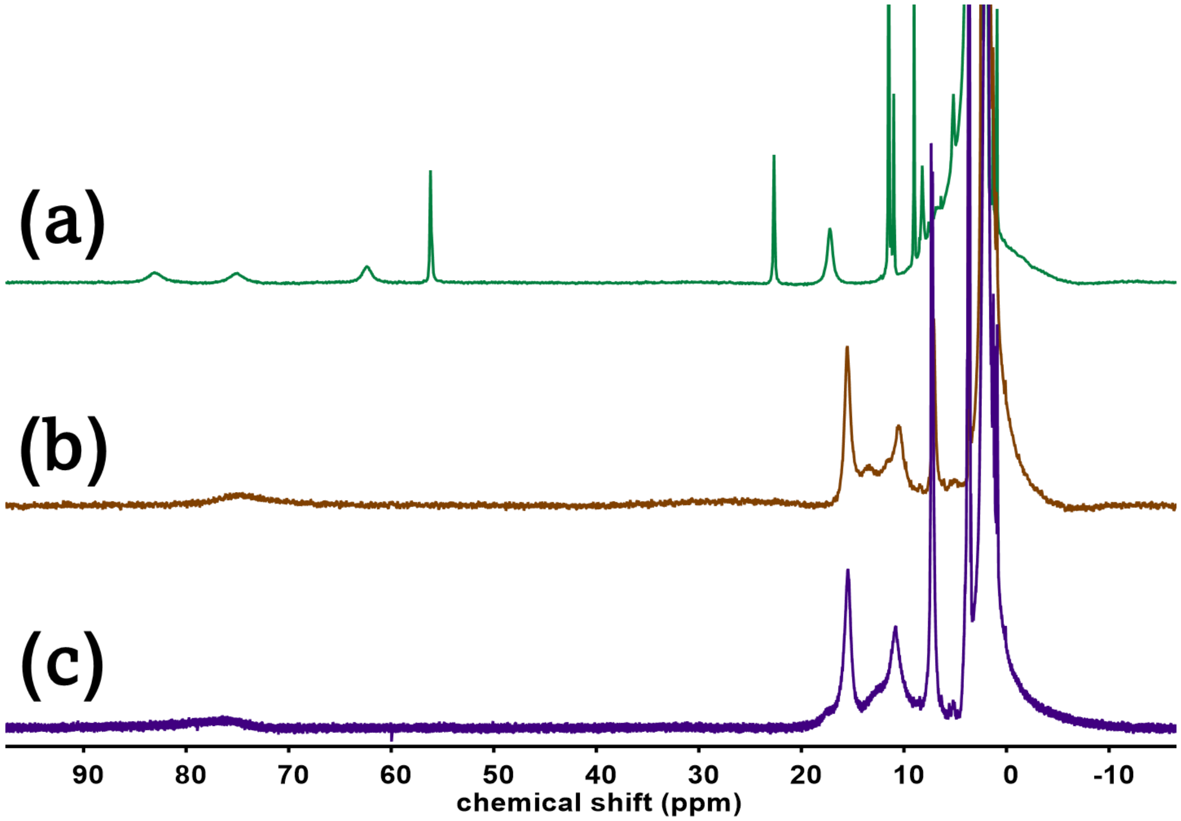

Figure S4. ^{1}H NMR spectra (CD_3CN) of (a) pure Fe^{II}(BNPA^{Ph2}O)(OH), (b) Fe^{II}(BNPA^{Ph2}O)(OH) + 10 equiv of (*p*-Cl-C_6H_4)$_3$C-Br in THF at 23 °C and (c) pure Fe^{III}(BNPA^{Ph2}O)(OH)(Br).

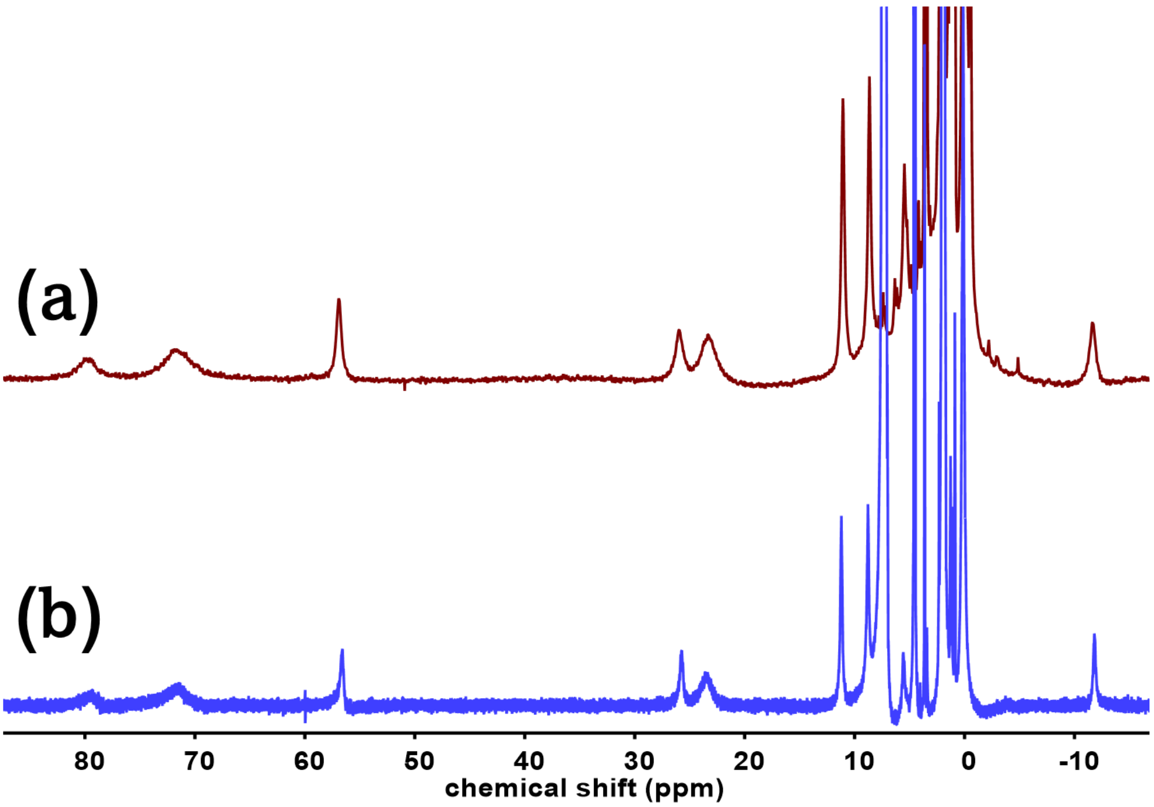

Figure S5. ^{1}H NMR spectra (CD_3CN) of (a) pure $Fe^{II}(BNPA^{Ph2}O)(Br)$ and (b) $Fe^{II}(BNPA^{Ph2}O)(Br)$ + 10 equiv of (p-Cl-C_6H_4)$_3$C-OH in THF at 23 °C.

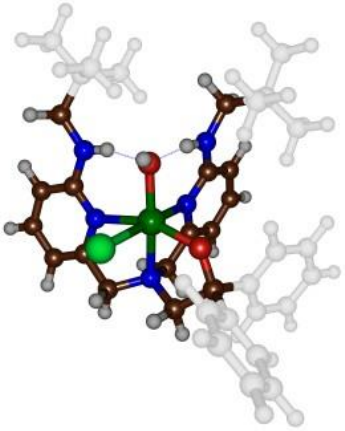

^{6}**1**$_{Cl}$

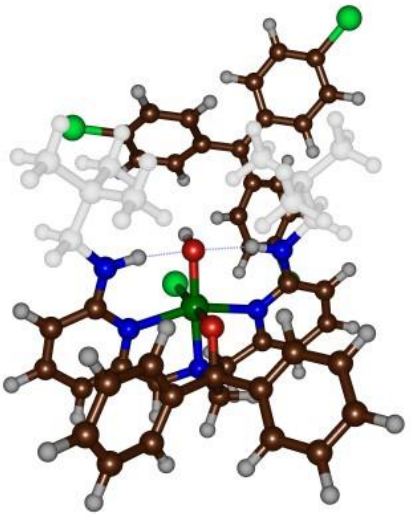

^{5}**Re'**$_{1Cl}$

Figure S6. Optimized geometry of ^{6}**1**$_{Cl}$ and the isomeric reactant complex with the equatorial ligand positions swapped.

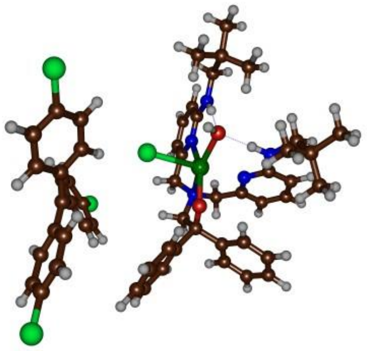

^{5}**Re**$_{1Cl}$

Figure S7. UB3LYP/BS2 optimized geometry of ^{5}**Re**$_{1Cl}$.

Cartesian coordinates of optimized geometries.

(*p*-Cl-C_6H_4)$_3$CCl:

17	8.028529000	0.313856000	-1.115958000
6	7.498180000	0.081923000	0.861755000
6	7.019132000	-1.358658000	0.950500000
6	8.786020000	0.360207000	1.620170000
6	6.406289000	1.118855000	1.071580000
6	6.619632000	2.465106000	0.710746000
6	5.190895000	0.774569000	1.687727000
6	4.206923000	1.741752000	1.934024000
6	4.452377000	3.055377000	1.554508000
6	5.647221000	3.437704000	0.944963000
1	5.817418000	4.466963000	0.655516000
17	3.170953000	4.325392000	1.863021000
1	3.275312000	1.465265000	2.411300000
1	5.001115000	-0.245816000	1.993336000
1	7.545654000	2.752429000	0.228056000
6	6.060337000	-1.852677000	0.042828000
6	7.464791000	-2.204505000	1.980992000
6	6.976883000	-3.512604000	2.103274000
6	6.040510000	-3.964684000	1.181913000
6	5.569534000	-3.154455000	0.149034000
1	4.840104000	-3.526935000	-0.559044000
1	5.706781000	-1.221127000	-0.762821000
1	8.186392000	-1.852109000	2.706251000
1	7.326186000	-4.154288000	2.902263000
17	5.402882000	-5.674105000	1.326647000
6	8.809450000	1.248916000	2.708656000
6	9.984746000	1.460843000	3.442427000
6	11.134974000	0.775015000	3.073251000
6	11.150706000	-0.119779000	2.003280000
6	9.972340000	-0.324438000	1.285279000
1	7.912779000	1.777046000	3.004822000
1	9.990429000	2.146737000	4.280063000
17	12.677912000	1.050813000	4.018078000
1	12.058708000	-0.643233000	1.731756000
1	9.979301000	-1.009358000	0.446254000

(*p*-Cl-C_6H_4)$_3$CBr:

35	8.061607000	0.338955000	-1.225673000
6	7.516332000	0.092166000	0.798661000
6	7.036876000	-1.352516000	0.911255000
6	8.795170000	0.370476000	1.582162000
6	6.412993000	1.119180000	1.036811000
6	6.574601000	2.452683000	0.613675000
6	5.255102000	0.778630000	1.754778000
6	4.277046000	1.740048000	2.045625000
6	4.474468000	3.044655000	1.611010000
6	5.611910000	3.421753000	0.897587000
1	5.743614000	4.443156000	0.563328000
17	3.201840000	4.309125000	1.980661000
1	3.387056000	1.466184000	2.598122000
1	5.104127000	-0.235921000	2.099923000
1	7.450425000	2.725588000	0.038564000
6	6.145845000	-1.888209000	-0.038125000
6	7.397536000	-2.143887000	2.014337000
6	6.886949000	-3.440054000	2.170810000
6	6.010178000	-3.931698000	1.211760000
6	5.626881000	-3.175617000	0.104587000
1	4.946330000	-3.579496000	-0.634422000
1	5.875819000	-1.298912000	-0.905220000
1	8.074292000	-1.759057000	2.766376000
1	7.173099000	-4.042093000	3.024025000
17	5.339120000	-5.625224000	1.403312000
6	8.783017000	1.191958000	2.720700000
6	9.942110000	1.380797000	3.486855000
6	11.109620000	0.735344000	3.099844000
6	11.160308000	-0.090485000	1.976858000
6	9.999411000	-0.266190000	1.223776000
1	7.871714000	1.688842000	3.027118000
1	9.922279000	2.017810000	4.362111000
17	12.631387000	0.976832000	4.090009000
1	12.082351000	-0.580256000	1.689925000
1	10.029982000	-0.884572000	0.335320000

Data for 1_{Cl}:

$^{5}Re_{1Cl}$:

26	6.287278000	12.868423000	3.594073000
17	7.688032000	12.306832000	1.524618000
8	5.163954000	11.319976000	3.302897000
1	5.581186000	10.523675000	2.924422000
6	4.251471000	8.741021000	5.999978000
1	5.098436000	9.335780000	5.639701000
1	3.698361000	8.371314000	5.127013000
1	4.650513000	7.873782000	6.539452000
6	2.142640000	8.709337000	7.390187000
1	2.498597000	7.833068000	7.944459000
1	1.555091000	8.351774000	6.535114000
1	1.474431000	9.277837000	8.049393000
6	4.135963000	10.062621000	8.149857000
1	4.959065000	10.719306000	7.847842000
1	4.563999000	9.208326000	8.688552000
1	3.495710000	10.613901000	8.849683000
6	3.334988000	9.576165000	6.922986000
6	2.748840000	10.785763000	6.130204000
1	2.153816000	10.387854000	5.297957000
1	2.056080000	11.338397000	6.771951000
6	4.089341000	12.919586000	6.035417000
6	3.525138000	13.484669000	7.212345000
1	2.782781000	12.935770000	7.773203000
6	3.950340000	14.726295000	7.649280000
1	3.525873000	15.154800000	8.551010000
6	4.939909000	15.419968000	6.937069000
1	5.305739000	16.382496000	7.272681000
6	5.455210000	14.837648000	5.782907000
6	6.466017000	15.623032000	4.975866000
1	5.919689000	16.398370000	4.426886000
1	7.144490000	16.143388000	5.660336000
6	7.260227000	15.542143000	2.655771000
1	7.967834000	15.018810000	2.006542000
1	7.585406000	16.585155000	2.757278000
6	5.885322000	15.463903000	2.041668000
6	5.352757000	16.510326000	1.300752000
1	5.898894000	17.439050000	1.193248000
6	4.089131000	16.327435000	0.709515000
1	3.631303000	17.132499000	0.144837000
6	3.425125000	15.121862000	0.847825000
1	2.451323000	14.978497000	0.401630000
6	4.028532000	14.065677000	1.591693000
6	2.246180000	12.451875000	0.976067000
1	2.284057000	12.868369000	-0.039657000
1	1.361970000	12.883721000	1.470371000
6	2.044622000	10.918049000	0.847471000
6	0.754617000	10.702928000	0.019487000
1	0.554150000	9.632026000	-0.099544000
1	0.847384000	11.143401000	-0.980928000
1	-0.113270000	11.156511000	0.514128000
6	1.867738000	10.262005000	2.236524000
1	2.775235000	10.350990000	2.841425000
1	1.643968000	9.194407000	2.123060000
1	1.037743000	10.725278000	2.784690000
6	3.240790000	10.276365000	0.106543000
1	4.174296000	10.400636000	0.665563000
1	3.375086000	10.727887000	-0.884136000
1	3.070015000	9.201976000	-0.030517000
6	8.645446000	14.474955000	4.420957000
1	9.138874000	15.334609000	4.888367000
1	9.199682000	14.216818000	3.517449000
6	8.647098000	13.204165000	5.329230000
6	8.346702000	13.471773000	6.822778000
6	7.672028000	12.485783000	7.563856000
1	7.321776000	11.599293000	7.049609000
6	7.447205000	12.646595000	8.934854000
1	6.922676000	11.872970000	9.487264000
6	7.894879000	13.798985000	9.594231000
1	7.720069000	13.925419000	10.657903000
6	8.572820000	14.784089000	8.868410000
1	8.928285000	15.680999000	9.366186000
6	8.800352000	14.619904000	7.495258000

1	9.339064000	15.398074000	6.965580000
6	10.036131000	12.527073000	5.229031000
6	11.211995000	13.235394000	5.537350000
1	11.162287000	14.269415000	5.862566000
6	12.463426000	12.614792000	5.445373000
1	13.360294000	13.176517000	5.687761000
6	12.558521000	11.273373000	5.050503000
1	13.528070000	10.790403000	4.982691000
6	11.391349000	10.562169000	4.746882000
1	11.453601000	9.523117000	4.438541000
6	10.139802000	11.185309000	4.833291000
1	9.233786000	10.644615000	4.591368000
7	3.741874000	11.696281000	5.559670000
1	4.226972000	11.390696000	4.701224000
7	5.038433000	13.628494000	5.319360000
7	7.262315000	14.840991000	3.978408000
7	5.233062000	14.280178000	2.223294000
7	3.468842000	12.834090000	1.690818000
1	3.987614000	12.135261000	2.241356000
8	7.645270000	12.327284000	4.789017000
6	12.294477000	10.855067000	0.131243000
6	13.416582000	10.281483000	0.879421000
6	14.609789000	11.017362000	1.102550000
6	15.680595000	10.474673000	1.814084000
6	15.564023000	9.180472000	2.319129000
6	14.413545000	8.415993000	2.130270000
6	13.351630000	8.966756000	1.411624000
1	14.340110000	7.416380000	2.540579000
17	16.966030000	8.461351000	3.256487000
1	16.588813000	11.046123000	1.961290000
1	14.706298000	12.016032000	0.693444000
1	12.449611000	8.381143000	1.279836000
6	12.011078000	12.290802000	0.216501000
6	12.240262000	13.017763000	1.413967000
1	12.607044000	12.502246000	2.293580000
6	11.966017000	14.383469000	1.501674000
1	12.132796000	14.916973000	2.429377000
6	11.460902000	15.039805000	0.380338000
17	11.099737000	16.835510000	0.488494000
6	11.220514000	14.373176000	-0.819929000
1	10.837592000	14.905379000	-1.682009000
6	11.492499000	13.006435000	-0.894090000
1	11.321424000	12.489219000	-1.830658000
6	11.450750000	9.990936000	-0.701580000
6	11.995510000	8.882037000	-1.399972000
6	11.198654000	8.058679000	-2.196613000
1	11.633466000	7.225295000	-2.734619000
1	13.057963000	8.678543000	-1.335465000
6	10.058669000	10.232642000	-0.836850000
1	9.596680000	11.048845000	-0.292508000
6	9.251669000	9.410827000	-1.625522000
1	8.188064000	9.599990000	-1.703807000
6	9.836441000	8.337551000	-2.295358000
17	8.782165000	7.256677000	-3.336965000

$^{5}TS_{OH,1Cl}$:

26	7.110825000	11.835634000	3.170699000
17	9.057717000	11.872726000	1.229596000
8	6.201415000	10.001740000	2.670947000
1	6.451887000	9.468314000	3.456701000
6	4.359910000	8.271272000	6.353892000
1	5.230923000	8.922812000	6.220483000
1	4.359620000	7.528607000	5.547352000
1	4.480467000	7.737225000	7.304138000
6	1.842386000	8.145082000	6.542170000
1	1.912518000	7.613824000	7.498507000
1	1.800416000	7.394918000	5.742899000
1	0.897632000	8.703531000	6.535694000
6	3.085969000	10.101377000	7.530267000
1	3.926195000	10.797344000	7.437250000
1	3.196532000	9.567778000	8.482645000
1	2.159546000	10.686341000	7.580048000
6	3.052156000	9.092655000	6.360509000
6	2.862797000	9.816881000	4.988196000
1	2.755700000	9.049171000	4.215835000

1	1.917265000	10.371049000	5.006213000
6	4.088918000	12.023738000	4.873097000
6	3.031615000	12.794330000	5.421281000
1	2.061239000	12.349573000	5.590145000
6	3.257019000	14.126356000	5.732831000
1	2.450693000	14.730894000	6.134783000
6	4.529640000	14.681768000	5.541404000
1	4.743470000	15.707071000	5.818870000
6	5.534642000	13.872629000	5.009453000
6	6.955248000	14.392930000	5.007375000
1	6.941975000	15.483483000	4.877931000
1	7.354849000	14.203291000	6.007516000
6	8.033809000	14.634403000	2.813717000
1	8.874161000	14.240601000	2.234876000
1	8.254112000	15.675893000	3.089395000
6	6.804004000	14.584668000	1.937777000
6	6.409909000	15.698581000	1.204206000
1	6.940432000	16.636802000	1.309570000
6	5.322150000	15.567241000	0.327316000
1	4.994789000	16.413239000	-0.267489000
6	4.652086000	14.358875000	0.228571000
1	3.800520000	14.264124000	-0.427700000
6	5.075324000	13.258052000	1.020399000
6	3.417603000	11.652522000	0.020427000
1	3.634256000	10.621603000	-0.280914000
1	3.531309000	12.264802000	-0.880461000
6	1.931540000	11.707749000	0.493105000
6	1.055397000	11.343147000	-0.728913000
1	-0.004585000	11.327637000	-0.449676000
1	1.315567000	10.351820000	-1.121017000
1	1.180980000	12.073109000	-1.538457000
6	1.543410000	13.111251000	1.006577000
1	2.180080000	13.418505000	1.843618000
1	0.504267000	13.107857000	1.357891000
1	1.622244000	13.865810000	0.215309000
6	1.704947000	10.673422000	1.615856000
1	2.352777000	10.881833000	2.473046000
1	1.915537000	9.656544000	1.259084000
1	0.663692000	10.697663000	1.959307000
6	9.194955000	13.359398000	4.611415000
1	9.598545000	14.141417000	5.268904000
1	9.878953000	13.236699000	3.773382000
6	9.093325000	11.973532000	5.348797000
6	8.497599000	12.119598000	6.767336000
6	7.487188000	11.245294000	7.194060000
1	7.126505000	10.506546000	6.490573000
6	6.955867000	11.331973000	8.487464000
1	6.175448000	10.642595000	8.795360000
6	7.426447000	12.301261000	9.381225000
1	7.013727000	12.372200000	10.382745000
6	8.441196000	13.176255000	8.971877000
1	8.821903000	13.927881000	9.656828000
6	8.974073000	13.081892000	7.680399000
1	9.775992000	13.755702000	7.394298000
6	10.520616000	11.354236000	5.420638000
6	11.134188000	10.964060000	6.623785000
1	10.624035000	11.112309000	7.567035000
6	12.409756000	10.380715000	6.631514000
1	12.858365000	10.089951000	7.576897000
6	13.099665000	10.174589000	5.433655000
1	14.089088000	9.727668000	5.438287000
6	12.495151000	10.547130000	4.225101000
1	13.013066000	10.381634000	3.285335000
6	11.220409000	11.121973000	4.218551000
1	10.755777000	11.375223000	3.271404000
7	3.953216000	10.696269000	4.563379000
1	4.685551000	10.307726000	3.973013000
7	5.317073000	12.586227000	4.631351000
7	7.883935000	13.777370000	4.021376000
7	6.159592000	13.385092000	1.849361000
7	4.445148000	12.042846000	0.994659000
1	4.852804000	11.307031000	1.565037000
8	8.262179000	11.120505000	4.540961000
6	6.613243000	8.169537000	1.370314000
6	8.072685000	8.021560000	1.570600000

6	8.936794000	7.896775000	0.457610000
6	10.300217000	7.646586000	0.621465000
6	10.809026000	7.532952000	1.912038000
6	10.000200000	7.665868000	3.040116000
6	8.637305000	7.901223000	2.862601000
1	10.420642000	7.593278000	4.034553000
17	12.588789000	7.185981000	2.131878000
1	10.944579000	7.533308000	-0.240976000
1	8.539043000	7.959073000	-0.546383000
1	8.016478000	8.009048000	3.743105000
6	6.148820000	8.693154000	0.065083000
6	6.724641000	9.862057000	-0.482793000
1	7.443306000	10.431152000	0.098657000
6	6.367632000	10.309788000	-1.755796000
1	6.808726000	11.211762000	-2.161636000
6	5.436003000	9.577680000	-2.490262000
17	4.974560000	10.153116000	-4.164496000
6	4.846521000	8.418768000	-1.991206000
1	4.136262000	7.856837000	-2.584473000
6	5.202349000	7.983624000	-0.711463000
1	4.768661000	7.064379000	-0.339145000
6	5.711385000	7.255176000	2.106603000
6	6.179545000	6.037268000	2.652888000
6	5.304482000	5.127048000	3.251972000
1	5.676211000	4.192686000	3.652910000
1	7.226111000	5.773748000	2.577767000
6	4.325665000	7.530239000	2.193023000
1	3.946440000	8.465946000	1.805319000
6	3.441687000	6.626112000	2.780305000
1	2.382274000	6.843886000	2.829921000
6	3.948143000	5.434884000	3.301847000
17	2.794356000	4.243795000	4.066458000

$^{5}P_{OH,1Cl}$:

26	7.220714000	12.017855000	3.389223000
17	6.836143000	9.929388000	1.857464000
8	4.775856000	7.706456000	0.556717000
1	5.478977000	8.253214000	0.982320000
6	4.160677000	7.734054000	4.524473000
1	5.047793000	8.292561000	4.845429000
1	4.331115000	7.381695000	3.500300000
1	4.062035000	6.857723000	5.176097000
6	1.656800000	7.770122000	4.197619000
1	1.533566000	6.918707000	4.876946000
1	1.764534000	7.376065000	3.179244000
1	0.738163000	8.369298000	4.236807000
6	2.707676000	9.133652000	6.045517000
1	3.555531000	9.755476000	6.353642000
1	2.636416000	8.293111000	6.746752000
1	1.790611000	9.728156000	6.141623000
6	2.890493000	8.610204000	4.604591000
6	2.999610000	9.786747000	3.586605000
1	3.083054000	9.356979000	2.581575000
1	2.077015000	10.375456000	3.603318000
6	4.176978000	11.876708000	4.405958000
6	3.019909000	12.493607000	4.950035000
1	2.055090000	12.014327000	4.868207000
6	3.143511000	13.715425000	5.590931000
1	2.262038000	14.199897000	5.997238000
6	4.404795000	14.317118000	5.732026000
1	4.523934000	15.254036000	6.262357000
6	5.512421000	13.665243000	5.197254000
6	6.901644000	14.195854000	5.507228000
1	6.870389000	15.292563000	5.544462000
1	7.146616000	13.853155000	6.517827000
6	8.361496000	14.772238000	3.587756000
1	9.327370000	14.478550000	3.162251000
1	8.496835000	15.757948000	4.054819000
6	7.374472000	14.881960000	2.449744000
6	7.096750000	16.122543000	1.892191000
1	7.506636000	17.022429000	2.333180000
6	6.290007000	16.173847000	0.742342000
1	6.055537000	17.127322000	0.281631000
6	5.792030000	15.005732000	0.197352000
1	5.163510000	15.038383000	-0.679705000

6	6.088410000	13.757951000	0.814389000
6	4.970552000	12.367944000	-0.974788000
1	5.400247000	11.453183000	-1.402010000
1	5.233149000	13.184109000	-1.656740000
6	3.421934000	12.196387000	-0.936457000
6	2.964029000	11.873310000	-2.378094000
1	1.876060000	11.745041000	-2.414002000
1	3.425270000	10.946537000	-2.741715000
1	3.231585000	12.681615000	-3.070571000
6	2.727180000	13.488384000	-0.452929000
1	3.064943000	13.770615000	0.550698000
1	1.641137000	13.340760000	-0.411736000
1	2.922924000	14.326576000	-1.133315000
6	3.049136000	11.030714000	0.003998000
1	3.352639000	11.252306000	1.032620000
1	3.536778000	10.095617000	-0.297871000
1	1.964680000	10.866681000	-0.003381000
6	9.195662000	13.202656000	5.313883000
1	9.418399000	13.804422000	6.203738000
1	10.041092000	13.301067000	4.630631000
6	9.065867000	11.673768000	5.627953000
6	8.232043000	11.363488000	6.899787000
6	7.382849000	10.243685000	6.897131000
1	7.304639000	9.662792000	5.986721000
6	6.650171000	9.890002000	8.036050000
1	6.001732000	9.019311000	8.008467000
6	6.751555000	10.652133000	9.206909000
1	6.182226000	10.381005000	10.090406000
6	7.598152000	11.766443000	9.226404000
1	7.689675000	12.365951000	10.127097000
6	8.334365000	12.115049000	8.085781000
1	8.988542000	12.979325000	8.138864000
6	10.494597000	11.102869000	5.834772000
6	11.401767000	11.668578000	6.749834000
1	11.117314000	12.527341000	7.348860000
6	12.685087000	11.131778000	6.910481000
1	13.371756000	11.584908000	7.619267000
6	13.081512000	10.014841000	6.164052000
1	14.075158000	9.596515000	6.291287000
6	12.184021000	9.444334000	5.253303000
1	12.480052000	8.578101000	4.668781000
6	10.903552000	9.986871000	5.088358000
1	10.202323000	9.563111000	4.380693000
7	4.153446000	10.672024000	3.769286000
1	5.031772000	10.353540000	3.369412000
7	5.402761000	12.494409000	4.508925000
7	7.993648000	13.743789000	4.598323000
7	6.873069000	13.711987000	1.946387000
7	5.633805000	12.574891000	0.318420000
1	5.935129000	11.732238000	0.802487000
8	8.471096000	11.063472000	4.487058000
6	5.326122000	6.444996000	0.034091000
6	6.476534000	6.758918000	-0.944580000
6	7.578872000	5.903922000	-1.103570000
6	8.580410000	6.185388000	-2.042660000
6	8.462578000	7.334133000	-2.817131000
6	7.382901000	8.204995000	-2.690324000
6	6.390141000	7.906094000	-1.752057000
1	7.311949000	9.093549000	-3.305407000
17	9.771468000	7.715496000	-4.046083000
1	9.428839000	5.522395000	-2.157618000
1	7.673360000	5.013138000	-0.493923000
1	5.542644000	8.571160000	-1.640915000
6	4.161433000	5.789462000	-0.730737000
6	2.840825000	6.211183000	-0.512155000
1	2.651025000	7.015719000	0.184462000
6	1.772050000	5.610744000	-1.190203000
1	0.755756000	5.944446000	-1.020267000
6	2.043234000	4.580447000	-2.084235000
17	0.649239000	3.788293000	-2.980071000
6	3.339121000	4.132166000	-2.324389000
1	3.528707000	3.330073000	-3.026800000
6	4.395736000	4.746435000	-1.642901000
1	5.406525000	4.406640000	-1.836906000
6	5.793305000	5.598175000	1.237323000

6	6.873082000	6.056871000	2.018027000
6	7.289861000	5.362266000	3.157112000
1	8.121107000	5.723675000	3.749666000
1	7.397800000	6.966268000	1.744979000
6	5.135431000	4.423983000	1.632820000
1	4.298987000	4.047303000	1.058557000
6	5.541094000	3.713119000	2.771073000
1	5.026964000	2.806407000	3.064801000
6	6.612447000	4.197478000	3.511654000
17	7.154054000	3.274241000	5.002397000

^{5}TS$_{Cl,1Cl}$:

26	6.874989000	12.733024000	3.626713000
17	8.684183000	12.281002000	1.794956000
8	6.041524000	11.004185000	3.337679000
1	6.600528000	10.223045000	3.172630000
6	5.062955000	8.602841000	6.188630000
1	5.865114000	9.273492000	5.860514000
1	4.695364000	8.055954000	5.310775000
1	5.494024000	7.873576000	6.884972000
6	2.792101000	8.411952000	7.277631000
1	3.172571000	7.656583000	7.975256000
1	2.384489000	7.888694000	6.403393000
1	1.968431000	8.943191000	7.771399000
6	4.457838000	10.112058000	8.122142000
1	5.229801000	10.841690000	7.854202000
1	4.901941000	9.389306000	8.817451000
1	3.655516000	10.639479000	8.652872000
6	3.918873000	9.389222000	6.868772000
6	3.308490000	10.409995000	5.858782000
1	2.918790000	9.843715000	5.002992000
1	2.448908000	10.906556000	6.319831000
6	4.336453000	12.707654000	5.721525000
6	3.528454000	13.281691000	6.740568000
1	2.791823000	12.679952000	7.252772000
6	3.710292000	14.609069000	7.090284000
1	3.099655000	15.048322000	7.872182000
6	4.692708000	15.376979000	6.448400000
1	4.868979000	16.410121000	6.722358000
6	5.451619000	14.775496000	5.446768000
6	6.466504000	15.622617000	4.708994000
1	5.915298000	16.278055000	4.024627000
1	6.972602000	16.277077000	5.427261000
6	7.556968000	15.444571000	2.518717000
1	8.418519000	14.988228000	2.023423000
1	7.701782000	16.532690000	2.537914000
6	6.312080000	15.083707000	1.746378000
6	5.731409000	15.966021000	0.843372000
1	6.130967000	16.964800000	0.719381000
6	4.615407000	15.524755000	0.109755000
1	4.123167000	16.194239000	-0.587632000
6	4.140097000	14.235709000	0.276877000
1	3.278711000	13.892435000	-0.278429000
6	4.787094000	13.360606000	1.197841000
6	3.391657000	11.426747000	0.517654000
1	3.539304000	11.736456000	-0.526485000
1	2.387070000	11.766645000	0.815052000
6	3.422919000	9.875582000	0.559565000
6	2.336125000	9.375578000	-0.422511000
1	2.303194000	8.280070000	-0.426226000
1	2.542596000	9.711787000	-1.445990000
1	1.343150000	9.742911000	-0.135000000
6	3.099471000	9.353113000	1.978691000
1	3.868659000	9.646869000	2.699830000
1	3.044711000	8.257732000	1.973890000
1	2.132495000	9.738406000	2.325995000
6	4.803596000	9.348593000	0.103684000
1	5.600501000	9.670713000	0.782356000
1	5.046822000	9.708817000	-0.903330000
1	4.801718000	8.252308000	0.080992000
6	8.836951000	14.780470000	4.540365000
1	9.126057000	15.743936000	4.976559000
1	9.541221000	14.547594000	3.740516000
6	8.914146000	13.604833000	5.568777000
6	8.388665000	13.961540000	6.981792000

6	7.715771000	12.976846000	7.725034000
1	7.528298000	12.017259000	7.260190000
6	7.287310000	13.230119000	9.032388000
1	6.768130000	12.454305000	9.587101000
6	7.523553000	14.477436000	9.624713000
1	7.189647000	14.676174000	10.638178000
6	8.198145000	15.464186000	8.897179000
1	8.391712000	16.434717000	9.343704000
6	8.631047000	15.206458000	7.589476000
1	9.162455000	15.989231000	7.058659000
6	10.392781000	13.161557000	5.704524000
6	11.415648000	14.091884000	5.964081000
1	11.184320000	15.145606000	6.079680000
6	12.747013000	13.675407000	6.086414000
1	13.522749000	14.408326000	6.286207000
6	13.076494000	12.319655000	5.957723000
1	14.108007000	11.996134000	6.054720000
6	12.063047000	11.387054000	5.705197000
1	12.306256000	10.333885000	5.602210000
6	10.732006000	11.805362000	5.578424000
1	9.943812000	11.091741000	5.374870000
7	4.241778000	11.407294000	5.335922000
1	4.892792000	11.106397000	4.592667000
7	5.274924000	13.483875000	5.067536000
7	7.486443000	14.881503000	3.902249000
7	5.840438000	13.822195000	1.953733000
7	4.415526000	12.064079000	1.352058000
1	4.942785000	11.510602000	2.044294000
8	8.139924000	12.530211000	5.022993000
6	10.476664000	11.319407000	0.335244000
6	10.718693000	10.072336000	1.100889000
6	12.005959000	9.729654000	1.575349000
6	12.236892000	8.540711000	2.275470000
6	11.167989000	7.680322000	2.503824000
6	9.880666000	7.968797000	2.051041000
6	9.666079000	9.158126000	1.354403000
1	9.061029000	7.287019000	2.242473000
17	11.459370000	6.118904000	3.422962000
1	13.232660000	8.293052000	2.622081000
1	12.845158000	10.385484000	1.380875000
1	8.664297000	9.398999000	1.021225000
6	11.451855000	12.428236000	0.473168000
6	11.924227000	12.826941000	1.748484000
1	11.532228000	12.345614000	2.635583000
6	12.859969000	13.851553000	1.894617000
1	13.199375000	14.146049000	2.880168000
6	13.336936000	14.491457000	0.750977000
17	14.565309000	15.843576000	0.934522000
6	12.910423000	14.133799000	-0.523881000
1	13.302577000	14.633325000	-1.401251000
6	11.971359000	13.105096000	-0.654812000
1	11.658375000	12.815485000	-1.650150000
6	9.688971000	11.229318000	-0.918516000
6	9.564963000	10.014105000	-1.631404000
6	8.844154000	9.936193000	-2.827728000
1	8.769266000	8.997523000	-3.362903000
1	10.052616000	9.120344000	-1.262998000
6	9.058681000	12.373879000	-1.466835000
1	9.114801000	13.314725000	-0.934253000
6	8.335863000	12.311737000	-2.658224000
1	7.852140000	13.195730000	-3.055443000
6	8.238819000	11.087849000	-3.320003000
17	7.286747000	10.996799000	-4.886574000

$^5P_{Cl,1Cl}$:

26	7.099300000	12.664443000	3.797263000
17	9.604607000	9.946958000	1.754634000
8	6.187899000	10.900244000	3.453733000
1	6.684090000	10.068440000	3.564761000
6	4.691121000	8.972568000	6.340797000
1	5.594734000	9.525776000	6.061494000
1	4.352870000	8.409382000	5.461504000
1	4.959787000	8.251280000	7.121987000
6	2.314953000	9.111509000	7.183668000
1	2.531395000	8.368677000	7.960543000

1	1.940407000	8.577492000	6.301220000
1	1.512972000	9.762966000	7.553645000
6	4.073771000	10.672613000	8.104405000
1	4.948343000	11.292453000	7.879473000
1	4.356604000	9.956305000	8.885661000
1	3.288741000	11.321415000	8.512744000
6	3.584335000	9.927060000	6.844535000
6	3.205729000	10.937081000	5.716841000
1	2.839921000	10.359248000	4.857125000
1	2.368418000	11.557438000	6.053542000
6	4.482345000	13.100661000	5.541164000
6	3.656777000	13.838807000	6.432188000
1	2.829061000	13.359592000	6.935351000
6	3.941766000	15.174261000	6.672145000
1	3.320271000	15.739620000	7.359129000
6	5.034120000	15.792211000	6.043149000
1	5.277722000	16.831032000	6.230594000
6	5.807963000	15.030600000	5.168999000
6	6.938349000	15.698299000	4.403485000
1	6.484802000	16.242833000	3.565393000
1	7.406060000	16.453519000	5.048233000
6	8.266112000	15.110522000	2.413059000
1	9.168949000	14.558139000	2.132552000
1	8.481166000	16.180299000	2.274569000
6	7.150348000	14.688191000	1.485408000
6	6.775468000	15.486580000	0.409214000
1	7.228359000	16.459819000	0.265065000
6	5.801327000	14.992756000	-0.477086000
1	5.477770000	15.594061000	-1.320416000
6	5.248082000	13.739924000	-0.273149000
1	4.489385000	13.359190000	-0.942897000
6	5.666209000	12.960169000	0.847206000
6	4.265618000	11.032284000	0.177128000
1	4.629755000	11.144324000	-0.854990000
1	3.267373000	11.498590000	0.214593000
6	4.112748000	9.514763000	0.464485000
6	3.178239000	8.938386000	-0.626113000
1	3.024704000	7.864813000	-0.466892000
1	3.605790000	9.074585000	-1.627274000
1	2.196124000	9.427135000	-0.604208000
6	3.476946000	9.277689000	1.854626000
1	4.124623000	9.647288000	2.655866000
1	3.313261000	8.205051000	2.016466000
1	2.505268000	9.782390000	1.929607000
6	5.487004000	8.809718000	0.385407000
1	6.167272000	9.171990000	1.162932000
1	5.960527000	8.981911000	-0.589303000
1	5.366699000	7.727607000	0.517611000
6	9.229349000	14.676619000	4.648032000
1	9.496926000	15.650225000	5.079067000
1	10.025356000	14.396950000	3.955331000
6	9.174115000	13.534772000	5.721184000
6	8.496327000	13.961336000	7.050369000
6	7.742458000	13.008777000	7.757246000
1	7.610739000	12.030724000	7.311289000
6	7.168594000	13.315489000	8.995787000
1	6.593100000	12.560830000	9.523877000
6	7.333022000	14.589765000	9.555002000
1	6.886889000	14.830767000	10.514929000
6	8.081400000	15.548178000	8.862861000
1	8.218151000	16.540387000	9.282826000
6	8.660518000	15.234847000	7.625453000
1	9.241446000	15.999518000	7.120851000
6	10.639698000	13.128570000	6.046800000
6	11.605945000	14.066155000	6.455896000
1	11.340229000	15.111007000	6.577814000
6	12.923320000	13.669999000	6.719896000
1	13.654998000	14.409846000	7.030577000
6	13.295736000	12.325298000	6.589513000
1	14.315274000	12.017012000	6.799572000
6	12.337795000	11.383441000	6.191840000
1	12.613062000	10.337288000	6.093113000
6	11.023409000	11.784504000	5.919072000
1	10.272885000	11.073397000	5.597846000
7	4.297459000	11.785597000	5.241636000

1	4.976074000	11.356368000	4.580646000
7	5.542588000	13.724465000	4.917553000
7	7.977128000	14.788956000	3.840468000
7	6.602918000	13.464712000	1.725414000
7	5.186477000	11.715613000	1.088517000
1	5.523854000	11.248162000	1.958893000
8	8.494042000	12.433303000	5.137339000
6	10.995936000	10.438046000	0.323626000
6	11.937708000	9.244531000	0.307662000
6	13.329391000	9.417116000	0.399016000
6	14.198918000	8.319947000	0.332372000
6	13.659581000	7.049352000	0.175861000
6	12.284555000	6.836838000	0.077427000
6	11.431580000	7.938891000	0.140880000
1	11.885171000	5.837611000	-0.041891000
17	14.785328000	5.608313000	0.095057000
1	15.269547000	8.465257000	0.401863000
1	13.753058000	10.406176000	0.511839000
1	10.362018000	7.780515000	0.076952000
6	11.626066000	11.727041000	0.830818000
6	12.083646000	11.834948000	2.159167000
1	11.924718000	11.020541000	2.854520000
6	12.720450000	12.992016000	2.613030000
1	13.054241000	13.065209000	3.640698000
6	12.907489000	14.044541000	1.717782000
17	13.742169000	15.567008000	2.301065000
6	12.479761000	13.975259000	0.397226000
1	12.639218000	14.801736000	-0.283732000
6	11.835513000	12.810500000	-0.040984000
1	11.511178000	12.754651000	-1.071824000
6	10.172869000	10.610629000	-0.943944000
6	10.557176000	9.994841000	-2.147201000
6	9.833399000	10.205647000	-3.328955000
1	10.139789000	9.727550000	-4.250763000
1	11.429165000	9.355207000	-2.181728000
6	9.045404000	11.457137000	-0.951039000
1	8.727117000	11.939510000	-0.034764000
6	8.311851000	11.672060000	-2.118499000
1	7.442632000	12.318050000	-2.108616000
6	8.721369000	11.037395000	-3.291053000
17	7.764470000	11.313522000	-4.827458000

^{3}Re$_{1Cl}$:

26	6.152087000	12.617055000	3.452727000
17	7.548680000	11.444391000	1.342854000
8	5.049624000	11.117353000	3.315252000
1	5.537472000	10.386619000	2.881711000
6	4.023190000	8.926667000	6.513575000
1	4.904275000	9.446492000	6.120478000
1	3.518955000	8.428085000	5.675661000
1	4.369049000	8.151635000	7.207964000
6	1.829568000	9.137564000	7.747178000
1	2.130700000	8.361379000	8.460474000
1	1.294277000	8.649386000	6.922922000
1	1.128727000	9.810450000	8.257552000
6	3.795233000	10.571783000	8.418629000
1	4.649862000	11.162745000	8.074465000
1	4.167021000	9.809231000	9.113862000
1	3.121315000	11.234436000	8.975908000
6	3.067604000	9.907392000	7.230880000
6	2.558425000	10.982206000	6.221945000
1	1.987854000	10.464514000	5.441392000
1	1.854962000	11.651123000	6.728749000
6	3.979994000	13.038056000	5.854970000
6	3.438869000	13.801437000	6.924715000
1	2.659966000	13.388882000	7.549934000
6	3.943262000	15.067151000	7.179547000
1	3.539485000	15.649168000	8.001239000
6	4.987486000	15.588598000	6.397777000
1	5.417391000	16.560282000	6.606948000
6	5.463898000	14.810044000	5.347090000
6	6.546714000	15.346551000	4.438089000
1	6.088517000	16.056821000	3.741975000
1	7.285196000	15.901860000	5.023472000
6	7.414768000	14.818432000	2.189137000

1	8.083342000	14.128193000	1.666641000
1	7.861145000	15.818410000	2.187437000
6	6.076546000	14.788701000	1.512935000
6	5.718840000	15.695240000	0.529044000
1	6.375475000	16.517752000	0.276359000
6	4.494857000	15.497221000	-0.137597000
1	4.185397000	16.180464000	-0.920799000
6	3.690719000	14.423871000	0.193843000
1	2.765128000	14.253253000	-0.336030000
6	4.086488000	13.528050000	1.230787000
6	2.125320000	12.062571000	0.857814000
1	2.390787000	11.700384000	-0.148301000
1	1.488641000	12.946373000	0.726876000
6	1.278334000	10.978402000	1.572730000
6	0.086801000	10.651188000	0.640595000
1	-0.567771000	9.907328000	1.108920000
1	0.433230000	10.242505000	-0.316503000
1	-0.512304000	11.546709000	0.434454000
6	0.742199000	11.529015000	2.912977000
1	1.560299000	11.833821000	3.573212000
1	0.147211000	10.766871000	3.430171000
1	0.099699000	12.402544000	2.744648000
6	2.101184000	9.689644000	1.807708000
1	2.928112000	9.848811000	2.508111000
1	2.518616000	9.314929000	0.864741000
1	1.458427000	8.906248000	2.226717000
6	8.609170000	13.867799000	4.143549000
1	9.171916000	14.735162000	4.500703000
1	9.143312000	13.437240000	3.296823000
6	8.438657000	12.740546000	5.191229000
6	8.127989000	13.221912000	6.619752000
6	7.375906000	12.387508000	7.465018000
1	6.973328000	11.466798000	7.060447000
6	7.142481000	12.739015000	8.798043000
1	6.560907000	12.079109000	9.434027000
6	7.656645000	13.935968000	9.314978000
1	7.475261000	14.208925000	10.349602000
6	8.409887000	14.772547000	8.485759000
1	8.818838000	15.700737000	8.872624000
6	8.647033000	14.416100000	7.150528000
1	9.253766000	15.077624000	6.541135000
6	9.729563000	11.879498000	5.192106000
6	10.831842000	12.188224000	6.007942000
1	10.782526000	13.021213000	6.699728000
6	12.000069000	11.417001000	5.951253000
1	12.840007000	11.669106000	6.591360000
6	12.081096000	10.321444000	5.083283000
1	12.982884000	9.718220000	5.046103000
6	10.986429000	10.008991000	4.266755000
1	11.038499000	9.162544000	3.588631000
6	9.821077000	10.782925000	4.317013000
1	8.978843000	10.556316000	3.675553000
7	3.596223000	11.767887000	5.552346000
1	4.087622000	11.335458000	4.758382000
7	4.957121000	13.587559000	5.055375000
7	7.265149000	14.306772000	3.601437000
7	5.276673000	13.743654000	1.891716000
7	3.340494000	12.453647000	1.584356000
1	3.788271000	11.800226000	2.241354000
8	7.343367000	11.923859000	4.687995000
6	12.956300000	11.314391000	0.139959000
6	14.234270000	10.721959000	0.538698000
6	15.347289000	11.531359000	0.889905000
6	16.568564000	10.971544000	1.265997000
6	16.688692000	9.583031000	1.299956000
6	15.626967000	8.742928000	0.967575000
6	14.411764000	9.313561000	0.587840000
1	15.738461000	7.666555000	1.012313000
17	18.289705000	8.840564000	1.795907000
1	17.410081000	11.605640000	1.516560000
1	15.259819000	12.610207000	0.842658000
1	13.580135000	8.661467000	0.349137000
6	12.528958000	12.596699000	0.710417000
6	12.823960000	12.942936000	2.054378000
1	13.344533000	12.234931000	2.687892000

6	12.421120000	14.163543000	2.598999000
1	12.638398000	14.401251000	3.632947000
6	11.720539000	15.058223000	1.791806000
17	11.195176000	16.669679000	2.497334000
6	11.402702000	14.765907000	0.466322000
1	10.865345000	15.480210000	-0.145316000
6	11.802850000	13.538351000	-0.063887000
1	11.570855000	13.313952000	-1.098230000
6	12.097070000	10.631530000	-0.834026000
6	12.645957000	9.898224000	-1.917988000
6	11.829939000	9.253261000	-2.848664000
1	12.264666000	8.710616000	-3.679165000
1	13.721329000	9.854239000	-2.045389000
6	10.683482000	10.682222000	-0.724563000
1	10.216019000	11.203353000	0.103163000
6	9.858309000	10.033741000	-1.644700000
1	8.782386000	10.071356000	-1.527126000
6	10.446430000	9.331688000	-2.694818000
17	9.369346000	8.481530000	-3.912331000

Data for 2_{OH}:

$^{5}Re_{2OH}$:

26	7.099021000	12.650276000	4.094380000
17	6.139646000	10.427553000	3.483842000
6	3.526361000	8.891587000	6.699143000
1	4.494158000	9.394986000	6.805272000
1	3.557211000	8.279948000	5.788643000
1	3.402246000	8.217605000	7.555210000
6	1.022487000	9.162648000	6.502944000
1	0.860003000	8.501271000	7.361952000
1	1.006558000	8.546645000	5.595115000
1	0.180484000	9.864816000	6.453634000
6	2.357357000	10.756099000	7.937878000
1	3.288802000	11.322163000	8.049668000
1	2.251744000	10.105434000	8.814541000
1	1.520502000	11.465415000	7.944094000
6	2.367560000	9.912889000	6.644387000
6	2.522409000	10.823155000	5.386980000
1	2.474887000	10.184568000	4.496595000
1	1.669903000	11.506682000	5.325635000
6	3.969838000	12.887852000	5.621661000
6	2.917753000	13.724635000	6.088306000
1	1.915108000	13.333955000	6.183092000
6	3.189548000	15.036513000	6.428624000
1	2.389409000	15.682543000	6.773751000
6	4.505089000	15.521081000	6.345227000
1	4.751949000	16.532701000	6.642461000
6	5.498465000	14.662443000	5.887414000
6	6.940037000	15.101802000	5.966670000
1	6.995274000	16.197251000	6.000376000
1	7.340752000	14.732187000	6.913534000
6	7.838872000	15.495489000	3.695183000
1	8.644422000	15.148096000	3.042199000
1	8.057622000	16.526657000	4.000710000
6	6.540697000	15.440679000	2.925456000
6	5.972131000	16.593701000	2.398159000
1	6.416510000	17.561293000	2.594006000
6	4.813119000	16.465290000	1.614161000
1	4.333162000	17.346202000	1.201864000
6	4.278642000	15.212604000	1.368578000
1	3.382361000	15.107852000	0.774204000
6	4.908368000	14.060367000	1.920055000
6	3.364163000	12.543023000	0.699278000
1	3.540467000	13.150149000	-0.198982000
1	2.398652000	12.860855000	1.122010000
6	3.244865000	11.061335000	0.254183000
6	2.131792000	11.009036000	-0.821046000
1	1.986331000	9.978453000	-1.164127000
1	2.392053000	11.622540000	-1.692335000
1	1.176353000	11.369918000	-0.420453000
6	2.837190000	10.157008000	1.440476000
1	3.600889000	10.142723000	2.224362000
1	2.696348000	9.125562000	1.095960000
1	1.892295000	10.498462000	1.881237000
6	4.572953000	10.567552000	-0.365874000
1	5.386881000	10.555913000	0.366602000
1	4.874469000	11.208465000	-1.203848000
1	4.453372000	9.546512000	-0.747004000
6	9.200799000	14.244749000	5.354889000
1	9.619144000	15.065634000	5.949213000
1	9.808553000	14.120759000	4.460289000
6	9.209109000	12.881466000	6.135292000
6	8.874142000	13.036321000	7.631396000
6	7.990157000	12.131811000	8.242744000
1	7.536475000	11.361141000	7.632378000
6	7.692220000	12.230932000	9.607201000
1	7.004046000	11.523182000	10.059203000
6	8.276366000	13.236454000	10.386952000
1	8.043929000	13.315650000	11.444161000
6	9.166254000	14.138831000	9.791096000
1	9.630115000	14.920011000	10.385260000
6	9.465013000	14.037488000	8.426732000
1	10.169378000	14.739543000	7.991408000
6	10.587755000	12.199539000	5.927444000
6	11.491071000	11.944043000	6.970892000

1	11.249208000	12.234585000	7.985616000
6	12.715723000	11.307811000	6.719407000
1	13.399381000	11.120751000	7.541951000
6	13.053152000	10.913117000	5.421692000
1	14.000784000	10.420193000	5.227583000
6	12.154132000	11.157566000	4.373708000
1	12.403653000	10.850708000	3.362057000
6	10.933280000	11.791903000	4.621948000
1	10.234060000	11.969793000	3.809149000
7	3.774330000	11.577813000	5.309725000
1	4.572719000	11.084269000	4.922636000
7	5.248384000	13.383496000	5.480342000
7	7.818981000	14.585709000	4.877863000
7	6.016526000	14.196804000	2.718641000
7	4.447968000	12.805527000	1.656264000
1	4.904770000	12.023756000	2.114541000
8	8.203419000	12.058302000	5.502393000
6	11.429894000	10.930424000	-0.244718000
6	12.867961000	10.683530000	-0.383100000
6	13.693534000	11.524219000	-1.174985000
6	15.062132000	11.287414000	-1.309171000
6	15.624011000	10.199957000	-0.641860000
6	14.858549000	9.344011000	0.148413000
6	13.489762000	9.586549000	0.270150000
1	15.317538000	8.510869000	0.666338000
17	17.422015000	9.885690000	-0.810138000
1	15.673360000	11.931629000	-1.929255000
1	13.252373000	12.356414000	-1.710599000
1	12.895747000	8.932252000	0.897046000
6	10.909815000	12.299216000	-0.254466000
6	11.663607000	13.383411000	0.267772000
1	12.635412000	13.196004000	0.708473000
6	11.171690000	14.689400000	0.256666000
1	11.758432000	15.500959000	0.669259000
6	9.909828000	14.926750000	-0.286046000
17	9.262169000	16.641800000	-0.317645000
6	9.127265000	13.898077000	-0.810684000
1	8.155680000	14.107021000	-1.241691000
6	9.627786000	12.595141000	-0.789522000
1	9.029754000	11.798415000	-1.215384000
6	10.506922000	9.797212000	-0.098097000
6	10.725794000	8.580429000	-0.793856000
6	9.851057000	7.500479000	-0.665275000
1	10.023789000	6.583356000	-1.215010000
1	11.576436000	8.489049000	-1.459300000
6	9.367653000	9.880056000	0.743436000
1	9.179928000	10.780306000	1.320762000
6	8.490870000	8.801764000	0.886087000
1	7.638726000	8.882233000	1.550666000
6	8.746714000	7.631030000	0.175266000
17	7.596328000	6.212130000	0.351475000
8	8.424395000	12.633458000	2.718139000
1	8.157015000	12.932562000	1.825718000

$^5TS_{OH,2OH}$:

26	7.521133000	12.339044000	3.698806000
17	6.024886000	10.542354000	2.740747000
6	3.573955000	8.996437000	6.211229000
1	4.613974000	9.340972000	6.244542000
1	3.420372000	8.463093000	5.264475000
1	3.429761000	8.280610000	7.029486000
6	1.136483000	9.653894000	6.292404000
1	0.951560000	8.949105000	7.111501000
1	0.940676000	9.131719000	5.347405000
1	0.414235000	10.475063000	6.384082000
6	2.829129000	10.900529000	7.693087000
1	3.846075000	11.304488000	7.750345000
1	2.696620000	10.201315000	8.527769000
1	2.122465000	11.727858000	7.832849000
6	2.589701000	10.180062000	6.348392000
6	2.773425000	11.162404000	5.150063000
1	2.561595000	10.612973000	4.224832000
1	2.025753000	11.959236000	5.217474000
6	4.519503000	12.976758000	5.419070000
6	3.636837000	13.919435000	6.013311000

1	2.590594000	13.681496000	6.141061000
6	4.131723000	15.141729000	6.434035000
1	3.462128000	15.872764000	6.874935000
6	5.501450000	15.420459000	6.309897000
1	5.922062000	16.350308000	6.673039000
6	6.323574000	14.455098000	5.732520000
6	7.820944000	14.643747000	5.799672000
1	8.055425000	15.714051000	5.872366000
1	8.153725000	14.184228000	6.734383000
6	8.866305000	15.004740000	3.598369000
1	9.580103000	14.533986000	2.917165000
1	9.316301000	15.926281000	3.992928000
6	7.603928000	15.336279000	2.836671000
6	7.325244000	16.645855000	2.461761000
1	7.983686000	17.451424000	2.761982000
6	6.175524000	16.889376000	1.692101000
1	5.926033000	17.900197000	1.387676000
6	5.354445000	15.837541000	1.325479000
1	4.464354000	16.017323000	0.739856000
6	5.683617000	14.514030000	1.737036000
6	3.745023000	13.592807000	0.492767000
1	4.055615000	14.123994000	-0.418709000
1	2.978624000	14.212800000	0.982618000
6	3.088208000	12.253877000	0.067705000
6	1.930713000	12.614177000	-0.895577000
1	1.414277000	11.704561000	-1.222386000
1	2.304443000	13.130568000	-1.788172000
1	1.194527000	13.263795000	-0.406133000
6	2.509243000	11.510448000	1.293398000
1	3.294801000	11.208903000	1.992798000
1	1.984919000	10.603415000	0.969309000
1	1.791610000	12.143719000	1.829935000
6	4.102007000	11.353766000	-0.675977000
1	4.936083000	11.058080000	-0.031247000
1	4.509800000	11.869199000	-1.553746000
1	3.607227000	10.437959000	-1.021323000
6	9.858001000	13.366760000	5.172841000
1	10.393892000	14.023731000	5.870486000
1	10.481626000	13.212798000	4.292901000
6	9.557496000	11.956453000	5.790036000
6	9.125066000	12.021619000	7.281032000
6	7.961765000	11.355964000	7.698507000
1	7.373469000	10.836378000	6.953582000
6	7.570042000	11.371293000	9.043435000
1	6.666954000	10.849027000	9.345504000
6	8.335333000	12.055274000	9.995381000
1	8.031142000	12.069949000	11.037308000
6	9.501697000	12.718051000	9.591096000
1	10.107495000	13.248360000	10.319662000
6	9.895059000	12.695943000	8.247918000
1	10.811163000	13.203473000	7.962970000
6	10.833752000	11.081993000	5.714104000
6	12.122706000	11.608207000	5.921470000
1	12.263457000	12.666700000	6.114782000
6	13.250533000	10.776715000	5.884058000
1	14.236255000	11.202691000	6.045284000
6	13.108061000	9.405227000	5.643201000
1	13.981123000	8.760827000	5.616580000
6	11.828920000	8.873534000	5.434177000
1	11.706222000	7.812248000	5.241004000
6	10.703841000	9.705327000	5.467218000
1	9.713720000	9.304076000	5.290372000
7	4.110107000	11.740886000	5.014874000
1	4.793296000	11.191825000	4.501847000
7	5.851253000	13.277046000	5.240000000
7	8.605585000	14.027836000	4.692626000
7	6.804098000	14.281633000	2.497075000
7	4.908834000	13.452133000	1.379040000
1	5.169325000	12.532989000	1.724402000
8	8.521495000	11.366146000	5.006702000
6	10.178369000	11.311266000	0.560479000
6	11.360218000	12.179151000	0.761037000
6	11.943535000	12.914127000	-0.295563000
6	13.090293000	13.689528000	-0.094433000
6	13.656626000	13.728809000	1.175984000

6	13.122629000	13.011127000	2.246333000
6	11.981537000	12.238899000	2.031069000
1	13.580971000	13.050683000	3.226645000
17	15.155600000	14.745677000	1.449312000
1	13.532947000	14.238132000	-0.916403000
1	11.523134000	12.861447000	-1.292017000
1	11.550392000	11.687434000	2.856034000
6	9.256060000	11.593021000	-0.556964000
6	8.942480000	12.926305000	-0.931442000
1	9.365860000	13.757672000	-0.379224000
6	8.089803000	13.204692000	-1.999078000
1	7.856494000	14.228598000	-2.263203000
6	7.545308000	12.140724000	-2.717411000
17	6.437525000	12.499297000	-4.129683000
6	7.825180000	10.815728000	-2.393510000
1	7.401597000	10.003046000	-2.970326000
6	8.674149000	10.549470000	-1.316756000
1	8.904891000	9.518584000	-1.082495000
6	10.259281000	9.930458000	1.095416000
6	11.500382000	9.256233000	1.149506000
6	11.587653000	7.931045000	1.586447000
1	12.543084000	7.422046000	1.611413000
1	12.404150000	9.758112000	0.826026000
6	9.103064000	9.231958000	1.505588000
1	8.148529000	9.741727000	1.521767000
6	9.178092000	7.910972000	1.952479000
1	8.285870000	7.392632000	2.281482000
6	10.421373000	7.281817000	1.980656000
17	10.526425000	5.546581000	2.564587000
8	8.906219000	12.302875000	2.199720000
1	8.569559000	12.931995000	1.527965000

$^{5}P_{OH,2OH}$;

26	7.483384000	13.019809000	3.816641000
17	6.819892000	11.065878000	2.312741000
6	4.458648000	8.855221000	5.472981000
1	5.426537000	9.361015000	5.567584000
1	4.371907000	8.470734000	4.448883000
1	4.460459000	7.997945000	6.156697000
6	1.948441000	9.056576000	5.668591000
1	1.913432000	8.203864000	6.356582000
1	1.814507000	8.673189000	4.649310000
1	1.099748000	9.712438000	5.900579000
6	3.445657000	10.329450000	7.255729000
1	4.378083000	10.891750000	7.378895000
1	3.464558000	9.488835000	7.959957000
1	2.610510000	10.983016000	7.536257000
6	3.291397000	9.810936000	5.809321000
6	3.263887000	10.993445000	4.791896000
1	3.121052000	10.577475000	3.787851000
1	2.394741000	11.627004000	4.993875000
6	4.668583000	13.017861000	5.374213000
6	3.661048000	13.675567000	6.126840000
1	2.679152000	13.236768000	6.228958000
6	3.953040000	14.886213000	6.734776000
1	3.184440000	15.401168000	7.301239000
6	5.240566000	15.436662000	6.633747000
1	5.495092000	16.365628000	7.129366000
6	6.199446000	14.745881000	5.897149000
6	7.639156000	15.229370000	5.920947000
1	7.652634000	16.326858000	5.954241000
1	8.075688000	14.881438000	6.862890000
6	8.669376000	15.746781000	3.726680000
1	9.466633000	15.388837000	3.066163000
1	8.983498000	16.719827000	4.129804000
6	7.410268000	15.914247000	2.910927000
6	6.953302000	17.180988000	2.572263000
1	7.467822000	18.061279000	2.936424000
6	5.813876000	17.284675000	1.756138000
1	5.423846000	18.259094000	1.482593000
6	5.189315000	16.139385000	1.299167000
1	4.314295000	16.208751000	0.669543000
6	5.702574000	14.861373000	1.667105000
6	3.976452000	13.728627000	0.290015000
1	4.206206000	14.370082000	-0.573048000

1	3.108462000	14.171626000	0.800985000
6	3.568706000	12.333078000	-0.248943000
6	2.354057000	12.556207000	-1.183006000
1	2.019541000	11.600617000	-1.602345000
1	2.611234000	13.220238000	-2.017343000
1	1.511811000	13.000284000	-0.638028000
6	3.148451000	11.395126000	0.905693000
1	3.984351000	11.182356000	1.579593000
1	2.793432000	10.438903000	0.503146000
1	2.333932000	11.840043000	1.490671000
6	4.722573000	11.700782000	-1.061501000
1	5.601481000	11.503143000	-0.439406000
1	5.026656000	12.358840000	-1.884670000
1	4.398637000	10.746349000	-1.493662000
6	9.818139000	14.185304000	5.271591000
1	10.250481000	14.824481000	6.052271000
1	10.490158000	14.205848000	4.411177000
6	9.695743000	12.687803000	5.717799000
6	9.188735000	12.544000000	7.179601000
6	8.110745000	11.690495000	7.460608000
1	7.640398000	11.174245000	6.633890000
6	7.654457000	11.516392000	8.773664000
1	6.819512000	10.849517000	8.968385000
6	8.269348000	12.196390000	9.831554000
1	7.915656000	12.064415000	10.849345000
6	9.350802000	13.046263000	9.564590000
1	9.841164000	13.575345000	10.376232000
6	9.808763000	13.213374000	8.252158000
1	10.660541000	13.862423000	8.075098000
6	11.088795000	12.013777000	5.628275000
6	12.282265000	12.686888000	5.945917000
1	12.266464000	13.728044000	6.250926000
6	13.518417000	12.030597000	5.869420000
1	14.428049000	12.570210000	6.115290000
6	13.581940000	10.687984000	5.478612000
1	14.538664000	10.178027000	5.423849000
6	12.397466000	10.008968000	5.160039000
1	12.433837000	8.967935000	4.853142000
6	11.164776000	10.667618000	5.232326000
1	10.246253000	10.156528000	4.971883000
7	4.473981000	11.818096000	4.752794000
1	5.245405000	11.464503000	4.196080000
7	5.914932000	13.583901000	5.246292000
7	8.506488000	14.739049000	4.811334000
7	6.796787000	14.763468000	2.496172000
7	5.128977000	13.718394000	1.202344000
1	5.558929000	12.833320000	1.455268000
8	8.801579000	12.045721000	4.815741000
6	10.472422000	10.837164000	0.035693000
6	11.966398000	11.210773000	0.088318000
6	12.713856000	11.444409000	-1.076601000
6	14.086283000	11.718730000	-1.008887000
6	14.695208000	11.757752000	0.240993000
6	13.986180000	11.531568000	1.417686000
6	12.617452000	11.254899000	1.332250000
1	14.478389000	11.566426000	2.381929000
17	16.493231000	12.122825000	0.341796000
1	14.656287000	11.901419000	-1.911380000
1	12.234594000	11.428685000	-2.048759000
1	12.051208000	11.077126000	2.237186000
6	9.727858000	11.528166000	-1.123038000
6	9.858589000	12.924709000	-1.257747000
1	10.520247000	13.467440000	-0.592611000
6	9.148932000	13.629072000	-2.233056000
1	9.260858000	14.701645000	-2.331998000
6	8.294032000	12.919016000	-3.074609000
17	7.358977000	13.832390000	-4.363365000
6	8.125450000	11.544423000	-2.966158000
1	7.450392000	11.013220000	-3.625370000
6	8.845682000	10.853968000	-1.979907000
1	8.706193000	9.784166000	-1.892633000
6	10.365647000	9.294990000	0.009098000
6	10.885078000	8.558246000	-1.071458000
6	10.823506000	7.161593000	-1.090544000
1	11.223746000	6.603126000	-1.927610000

1	11.347066000	9.067896000	-1.909461000
6	9.796775000	8.598190000	1.084566000
1	9.410802000	9.143630000	1.935633000
6	9.727560000	7.198062000	1.082900000
1	9.284576000	6.669561000	1.917989000
6	10.241135000	6.506596000	-0.007623000
17	10.156515000	4.672563000	-0.021965000
8	9.913161000	11.360871000	1.284195000
1	8.937745000	11.216760000	1.340110000

$^5TS_{Cl,2OH}$:

26	7.561885000	13.703107000	3.818111000
17	7.379648000	11.034907000	1.961217000
6	5.777792000	8.313586000	5.036806000
1	6.542280000	9.044384000	5.325794000
1	5.907867000	8.081409000	3.971885000
1	5.959410000	7.395487000	5.608164000
6	3.311585000	7.783164000	4.915439000
1	3.455740000	6.866512000	5.499161000
1	3.398450000	7.523524000	3.852841000
1	2.290078000	8.141138000	5.096256000
6	4.212553000	9.177482000	6.819472000
1	4.915700000	9.960410000	7.124303000
1	4.418943000	8.285519000	7.423533000
1	3.196672000	9.515596000	7.058025000
6	4.355785000	8.851098000	5.317046000
6	4.084420000	10.113348000	4.441333000
1	4.149892000	9.815824000	3.388021000
1	3.057419000	10.455168000	4.604316000
6	4.795094000	12.351653000	5.402583000
6	3.595855000	12.584033000	6.124571000
1	2.780247000	11.876127000	6.083537000
6	3.490169000	13.730785000	6.897865000
1	2.575195000	13.925134000	7.447768000
6	4.567467000	14.626269000	6.983493000
1	4.513544000	15.511750000	7.605151000
6	5.722528000	14.345167000	6.254457000
6	6.960941000	15.202863000	6.440273000
1	6.668036000	16.183009000	6.843627000
1	7.576578000	14.716973000	7.202526000
6	7.404175000	16.541246000	4.407248000
1	8.155758000	16.633321000	3.617494000
1	7.388863000	17.466119000	5.002719000
6	6.057349000	16.336493000	3.745630000
6	5.116912000	17.363936000	3.724264000
1	5.317278000	18.296295000	4.237760000
6	3.912929000	17.154886000	3.034702000
1	3.151188000	17.927194000	3.017005000
6	3.693368000	15.955024000	2.376752000
1	2.764528000	15.780470000	1.852394000
6	4.698641000	14.949837000	2.414521000
6	3.427969000	13.523064000	0.818185000
1	3.263794000	14.424339000	0.211992000
1	2.505193000	13.347324000	1.392871000
6	3.636378000	12.332331000	-0.153679000
6	2.397561000	12.293461000	-1.081872000
1	2.477163000	11.457581000	-1.786182000
1	2.309425000	13.219430000	-1.663089000
1	1.474422000	12.162106000	-0.503909000
6	3.723242000	10.997125000	0.619826000
1	4.596608000	10.961057000	1.278556000
1	3.807344000	10.158282000	-0.081565000
1	2.823549000	10.841565000	1.227788000
6	4.905819000	12.543085000	-1.010992000
1	5.814216000	12.557158000	-0.399633000
1	4.851475000	13.489709000	-1.562700000
1	5.005914000	11.731996000	-1.742436000
6	9.271630000	15.292384000	5.487266000
1	9.563652000	15.955317000	6.313888000
1	9.747754000	15.652886000	4.577319000
6	9.768967000	13.805999000	5.735376000
6	9.661237000	13.419206000	7.232384000
6	9.129487000	12.167103000	7.582043000
1	8.791537000	11.525900000	6.777851000
6	9.030303000	11.770266000	8.921308000

1	8.613137000	10.797354000	9.165571000
6	9.465881000	12.621728000	9.944251000
1	9.387488000	12.318053000	10.983709000
6	10.008390000	13.869747000	9.611834000
1	10.356678000	14.537863000	10.394103000
6	10.108194000	14.261232000	8.270559000
1	10.549392000	15.226107000	8.039720000
6	11.252427000	13.743276000	5.256421000
6	12.331707000	13.384755000	6.080407000
1	12.165119000	13.162362000	7.127417000
6	13.637953000	13.308893000	5.571004000
1	14.454253000	13.030256000	6.231388000
6	13.888899000	13.590673000	4.225107000
1	14.900035000	13.539899000	3.831896000
6	12.817192000	13.939418000	3.388882000
1	12.997243000	14.154013000	2.338752000
6	11.515565000	14.008064000	3.894456000
1	10.685184000	14.255703000	3.236291000
7	5.004704000	11.233882000	4.638415000
1	5.904533000	11.170927000	4.181006000
7	5.821793000	13.254848000	5.447380000
7	7.805291000	15.372080000	5.228608000
7	5.853645000	15.137591000	3.125019000
7	4.552515000	13.776072000	1.727043000
1	5.277937000	13.079837000	1.833918000
8	8.997501000	12.902828000	4.938218000
6	9.123356000	10.042731000	0.988169000
6	10.027994000	9.835791000	2.165326000
6	11.335901000	10.355890000	2.174949000
6	12.203652000	10.104893000	3.244569000
6	11.747680000	9.335615000	4.307989000
6	10.453824000	8.812535000	4.344121000
6	9.602074000	9.065310000	3.271143000
1	10.116261000	8.227922000	5.190392000
17	12.873032000	8.997585000	5.707872000
1	13.204951000	10.514947000	3.243322000
1	11.694898000	10.946787000	1.343166000
1	8.588922000	8.683756000	3.301365000
6	9.560689000	11.065979000	-0.029189000
6	9.682853000	12.435234000	0.288300000
1	9.356947000	12.840549000	1.244967000
6	10.172646000	13.341465000	-0.655746000
1	10.244809000	14.392156000	-0.403742000
6	10.549907000	12.874218000	-1.912979000
17	11.192180000	14.064016000	-3.148392000
6	10.448342000	11.531948000	-2.261164000
1	10.749161000	11.185702000	-3.241809000
6	9.946126000	10.631523000	-1.314672000
1	9.879337000	9.584392000	-1.581216000
6	8.473540000	8.813815000	0.411918000
6	8.988491000	7.527213000	0.660589000
6	8.419680000	6.394958000	0.063452000
1	8.828751000	5.411399000	0.256245000
1	9.850615000	7.395840000	1.300753000
6	7.374912000	8.939434000	-0.466413000
1	6.959184000	9.919264000	-0.665671000
6	6.793611000	7.820397000	-1.059402000
1	5.942978000	7.926356000	-1.720667000
6	7.328713000	6.561633000	-0.780831000
17	6.572607000	5.089404000	-1.557242000
8	8.655839000	14.564975000	2.408265000
1	8.154922000	14.889273000	1.631778000

$^5P_{Cl,2OH}$:

26	6.669633000	12.736805000	3.839816000
17	7.756123000	4.822434000	4.688417000
6	2.591359000	9.509128000	2.853492000
1	3.629530000	9.588403000	3.195378000
1	2.550270000	9.840787000	1.808088000
1	2.303096000	8.451394000	2.884317000
6	0.200290000	10.241859000	3.209543000
1	-0.138298000	9.199262000	3.230758000
1	0.129862000	10.598208000	2.174117000
1	-0.492307000	10.833874000	3.821322000
6	1.707549000	9.842181000	5.196047000

1	2.717770000	9.940277000	5.608687000
1	1.426500000	8.782723000	5.240062000
1	1.016681000	10.399246000	5.841268000
6	1.648304000	10.353533000	3.740191000
6	2.059550000	11.856857000	3.665631000
1	1.960127000	12.183267000	2.622210000
1	1.349563000	12.453412000	4.248331000
6	3.808694000	12.697793000	5.271185000
6	2.904697000	13.060105000	6.309064000
1	1.844754000	12.880702000	6.197946000
6	3.395786000	13.645742000	7.464815000
1	2.707829000	13.936060000	8.252232000
6	4.776629000	13.856492000	7.624907000
1	5.178740000	14.299329000	8.527954000
6	5.623977000	13.455083000	6.595728000
6	7.126490000	13.490453000	6.776915000
1	7.393226000	14.248174000	7.527869000
1	7.426830000	12.520247000	7.181639000
6	8.063508000	15.147256000	5.192738000
1	8.993223000	15.252507000	4.621442000
1	8.187277000	15.743477000	6.107983000
6	6.957272000	15.744196000	4.347166000
6	6.652637000	17.098182000	4.467373000
1	7.115988000	17.696606000	5.242534000
6	5.750789000	17.661933000	3.549246000
1	5.489249000	18.712918000	3.617738000
6	5.192686000	16.876784000	2.554315000
1	4.492234000	17.300072000	1.847652000
6	5.523582000	15.492411000	2.485486000
6	4.193218000	15.157393000	0.424900000
1	4.668952000	16.049801000	-0.007947000
1	3.198499000	15.470181000	0.784216000
6	3.999234000	14.119724000	-0.713711000
6	3.224793000	14.830145000	-1.849560000
1	3.035953000	14.133759000	-2.674693000
1	3.794370000	15.679713000	-2.246128000
1	2.256283000	15.203588000	-1.493936000
6	3.173237000	12.908449000	-0.219547000
1	3.691735000	12.370408000	0.580493000
1	2.998935000	12.206964000	-1.044827000
1	2.194909000	13.233618000	0.157415000
6	5.368622000	13.640401000	-1.249191000
1	5.932865000	13.104030000	-0.478989000
1	5.975246000	14.488951000	-1.589589000
1	5.226928000	12.962634000	-2.099794000
6	9.206864000	12.967865000	5.500742000
1	9.757061000	13.102664000	6.442418000
1	9.800953000	13.419652000	4.704420000
6	9.046647000	11.449463000	5.122281000
6	8.667306000	10.554954000	6.329134000
6	7.733752000	9.520253000	6.142775000
1	7.272668000	9.416975000	5.169271000
6	7.388219000	8.657457000	7.189820000
1	6.667389000	7.864087000	7.016291000
6	7.964885000	8.816337000	8.456054000
1	7.696912000	8.150022000	9.269854000
6	8.886555000	9.849023000	8.662346000
1	9.335626000	9.991752000	9.640541000
6	9.233805000	10.707834000	7.610611000
1	9.945146000	11.503188000	7.807798000
6	10.417276000	11.004833000	4.525384000
6	11.506038000	10.587015000	5.310200000
1	11.404927000	10.499440000	6.384984000
6	12.734996000	10.258735000	4.720542000
1	13.561009000	9.938519000	5.348617000
6	12.898111000	10.338726000	3.332728000
1	13.850319000	10.085933000	2.876361000
6	11.818220000	10.749888000	2.539830000
1	11.928378000	10.812495000	1.461107000
6	10.594480000	11.081192000	3.131677000
1	9.752690000	11.390666000	2.525497000
7	3.429243000	12.150274000	4.084203000
1	4.175057000	12.017645000	3.373014000
7	5.158954000	12.907320000	5.442130000
7	7.902430000	13.704172000	5.520999000

7	6.395692000	14.943549000	3.403014000
7	5.017760000	14.669842000	1.531395000
1	5.196270000	13.650325000	1.654453000
8	8.042811000	11.368223000	4.112406000
6	9.079577000	4.965196000	3.102725000
6	8.592021000	6.181405000	2.335824000
6	8.338536000	6.115593000	0.954631000
6	7.937840000	7.253622000	0.242025000
6	7.797485000	8.452717000	0.929019000
6	8.044025000	8.568920000	2.296088000
6	8.440376000	7.422199000	2.988548000
1	7.952996000	9.520110000	2.817855000
17	7.272298000	9.943071000	-0.007731000
1	7.745974000	7.193889000	-0.821891000
1	8.460929000	5.185156000	0.416348000
1	8.623006000	7.495451000	4.053985000
6	8.915990000	3.646389000	2.362943000
6	7.636186000	3.159841000	2.028635000
1	6.754826000	3.695851000	2.357906000
6	7.480478000	1.982402000	1.295876000
1	6.492450000	1.611895000	1.053468000
6	8.621642000	1.292968000	0.887319000
17	8.425453000	-0.254486000	-0.071041000
6	9.901079000	1.745207000	1.185273000
1	10.775560000	1.199843000	0.853633000
6	10.041773000	2.925862000	1.926865000
1	11.039206000	3.281453000	2.149533000
6	10.428697000	5.133756000	3.781467000
6	11.298591000	6.180135000	3.430474000
6	12.561012000	6.301108000	4.027631000
1	13.216195000	7.118372000	3.754576000
1	11.009336000	6.909290000	2.685662000
6	10.858916000	4.196308000	4.743532000
1	10.201978000	3.386064000	5.035443000
6	12.111524000	4.304486000	5.347321000
1	12.428267000	3.584484000	6.091268000
6	12.942511000	5.362072000	4.976969000
17	14.585888000	5.514527000	5.770107000
8	5.527835000	12.144794000	2.308915000
1	5.831400000	11.411333000	1.742370000

Data for $1_{Cl,NS}$:

$^{5}Re_{1Cl,NS}$:

26	7.064320000	13.530199000	4.337821000
17	9.202496000	13.926783000	2.993466000
8	7.063152000	11.662166000	3.819830000
1	7.925031000	11.269891000	3.584463000
6	4.415721000	12.245409000	5.696136000
6	3.218300000	12.307893000	6.458505000
1	2.707360000	11.386338000	6.712060000
6	2.737998000	13.536381000	6.872341000
1	1.828962000	13.594039000	7.461295000
6	3.437206000	14.708361000	6.536908000
1	3.091449000	15.680713000	6.865833000
6	4.594538000	14.599070000	5.772770000
6	5.310130000	15.865709000	5.351953000
1	4.717533000	16.333921000	4.557937000
1	5.316440000	16.568523000	6.191899000
6	6.872437000	16.419502000	3.534721000
1	7.933515000	16.381715000	3.272172000
1	6.575168000	17.471665000	3.626167000
6	6.074355000	15.725730000	2.458430000
6	5.454660000	16.416503000	1.427398000
1	5.492461000	17.497966000	1.391669000
6	4.781006000	15.673152000	0.436692000
1	4.285742000	16.183890000	-0.381950000
6	4.751240000	14.292875000	0.506816000
1	4.245971000	13.700637000	-0.247097000
6	5.401925000	13.632754000	1.586510000
6	7.789094000	16.014081000	5.813238000
1	7.570625000	16.951932000	6.337081000
1	8.699929000	16.158288000	5.229995000
6	8.064635000	14.825486000	6.790049000
6	7.107255000	14.744177000	8.001622000
6	6.765938000	13.477986000	8.508649000
1	7.130727000	12.597641000	7.994298000
6	5.962164000	13.352929000	9.646313000
1	5.709926000	12.365095000	10.019734000
6	5.483196000	14.494484000	10.302402000
1	4.858146000	14.398825000	11.184536000
6	5.820271000	15.759900000	9.810267000
1	5.457737000	16.653526000	10.308881000
6	6.627866000	15.883666000	8.671542000
1	6.875930000	16.879922000	8.321382000
6	9.516896000	14.953382000	7.313761000
6	9.935704000	16.094234000	8.022296000
1	9.237163000	16.896888000	8.233902000
6	11.254553000	16.206779000	8.478001000
1	11.559981000	17.094010000	9.024234000
6	12.174079000	15.176987000	8.238126000
1	13.196514000	15.263662000	8.591336000
6	11.761904000	14.037015000	7.538124000
1	12.465288000	13.232181000	7.347258000
6	10.443614000	13.927039000	7.077676000
1	10.118801000	13.052783000	6.528632000
7	4.937372000	11.058348000	5.306206000
1	5.803811000	11.040476000	4.747592000
7	5.074512000	13.399863000	5.341016000
7	6.704212000	15.690451000	4.832771000
7	6.036860000	14.364708000	2.554475000
7	5.423135000	12.282869000	1.694868000
1	5.995230000	11.847378000	2.432031000
8	7.943092000	13.628723000	6.005154000
1	4.965738000	11.713045000	1.001970000
1	4.467656000	10.202268000	5.551998000
6	13.909271000	16.276825000	4.074278000
6	14.814161000	16.682390000	5.152837000
6	15.479776000	15.722131000	5.959952000
6	15.061616000	18.052424000	5.432050000
6	15.926076000	18.445726000	6.454181000
6	16.553053000	17.462785000	7.218357000
6	16.342347000	16.103924000	6.988450000
1	16.831275000	15.356971000	7.601729000
17	17.687777000	17.972005000	8.565050000
1	16.113201000	19.495816000	6.642167000
1	14.589617000	18.815363000	4.824609000

1	15.299515000	14.666925000	5.793195000
1	16.327731000	15.198213000	3.389646000
6	15.486480000	14.604936000	3.051143000
6	14.164258000	15.045511000	3.320243000
6	15.733451000	13.435952000	2.330239000
1	16.748883000	13.122275000	2.121521000
6	14.648741000	12.688531000	1.873956000
17	14.966527000	11.148350000	0.930406000
6	13.331664000	13.076295000	2.113536000
1	12.502300000	12.475471000	1.761038000
6	13.095239000	14.250796000	2.829705000
1	12.068661000	14.534992000	3.032500000
6	12.748444000	17.109912000	3.740935000
6	12.031555000	17.810284000	4.745443000
1	12.319845000	17.699735000	5.784079000
6	12.303504000	17.249733000	2.401053000
1	12.834385000	16.740427000	1.605606000
6	11.211701000	18.055579000	2.074688000
1	10.896340000	18.163402000	1.044248000
6	10.545644000	18.727733000	3.098079000
6	10.934145000	18.614062000	4.432125000
1	10.391764000	19.132472000	5.213194000
17	9.114985000	19.796831000	2.677145000

$^{5}TS_{OH,1Cl,NS}$:

26	8.188437000	15.434840000	4.925572000
17	9.123631000	18.313541000	4.396644000
8	10.063809000	15.290111000	3.904165000
1	10.201507000	16.261343000	4.014915000
6	8.321350000	12.271480000	4.888034000
6	7.838782000	10.960152000	5.121377000
1	8.491158000	10.109770000	4.960188000
6	6.536190000	10.798306000	5.570382000
1	6.151051000	9.802168000	5.761428000
6	5.721784000	11.921206000	5.792933000
1	4.711836000	11.814167000	6.170243000
6	6.245582000	13.186254000	5.528081000
6	5.377407000	14.418363000	5.714993000
1	4.666737000	14.455777000	4.880089000
1	4.779235000	14.294621000	6.626250000
6	5.495086000	16.716591000	4.824521000
1	5.995516000	17.673898000	5.003787000
1	4.420857000	16.851962000	5.015965000
6	5.725372000	16.322083000	3.385034000
6	4.758076000	16.502632000	2.403986000
1	3.780482000	16.890004000	2.663150000
6	5.084208000	16.169800000	1.074202000
1	4.349506000	16.297493000	0.286337000
6	6.343123000	15.681825000	0.771545000
1	6.619732000	15.427130000	-0.245107000
6	7.293251000	15.515474000	1.816332000
6	6.340949000	16.282436000	7.118817000
1	5.445170000	16.172321000	7.744618000
1	6.519520000	17.351500000	6.986207000
6	7.629817000	15.707389000	7.803552000
6	7.414107000	14.338144000	8.500229000
6	8.476291000	13.417245000	8.508393000
1	9.377903000	13.677113000	7.968111000
6	8.368539000	12.194801000	9.179395000
1	9.204012000	11.500618000	9.171236000
6	7.188824000	11.862975000	9.859299000
1	7.102412000	10.914207000	10.379882000
6	6.122631000	12.768770000	9.859259000
1	5.200774000	12.525907000	10.379518000
6	6.236088000	13.994904000	9.188862000
1	5.390599000	14.674065000	9.214144000
6	8.072086000	16.733463000	8.886833000
6	7.245492000	17.083249000	9.970624000
1	6.273605000	16.616897000	10.092409000
6	7.665500000	18.026413000	10.916606000
1	7.011597000	18.284754000	11.744397000
6	8.923728000	18.631400000	10.800072000
1	9.250707000	19.359953000	11.535675000
6	9.753817000	18.286557000	9.726596000
1	10.731695000	18.748519000	9.624596000

6	9.328435000	17.348635000	8.777331000
1	9.951743000	17.078604000	7.934793000
7	9.599439000	12.516655000	4.492743000
1	9.914379000	13.474394000	4.334914000
7	7.508726000	13.355622000	5.062488000
7	6.104952000	15.720848000	5.754606000
7	6.962376000	15.820084000	3.107999000
7	8.551691000	15.061817000	1.587720000
1	9.235806000	15.088573000	2.362779000
8	8.637921000	15.605047000	6.804611000
1	8.851740000	14.865690000	0.646622000
1	10.243915000	11.756426000	4.356766000
6	12.414189000	15.382242000	3.218388000
6	12.764882000	16.214120000	4.377581000
6	12.477112000	15.779276000	5.695900000
6	13.444191000	17.444052000	4.206660000
6	13.833221000	18.208307000	5.308033000
6	13.527345000	17.744781000	6.585289000
6	12.851019000	16.541369000	6.799047000
1	12.608433000	16.214298000	7.801880000
17	14.024266000	18.741438000	8.026979000
1	14.366988000	19.139519000	5.169087000
1	13.705013000	17.787586000	3.213994000
1	11.909720000	14.869784000	5.840941000
1	14.274342000	14.093056000	4.742252000
6	13.708920000	13.425726000	4.105175000
6	12.691912000	13.939169000	3.264494000
6	14.039396000	12.069463000	4.091680000
1	14.830562000	11.691174000	4.726052000
6	13.341832000	11.218477000	3.237431000
17	13.766724000	9.447734000	3.212158000
6	12.325643000	11.680226000	2.397323000
1	11.789316000	10.996777000	1.751431000
6	12.008292000	13.036848000	2.412961000
1	11.198611000	13.396380000	1.791414000
6	12.216341000	16.023428000	1.910331000
6	11.518849000	17.253621000	1.796806000
1	11.051097000	17.705800000	2.665790000
6	12.768940000	15.455441000	0.734378000
1	13.348601000	14.543576000	0.791116000
6	12.629812000	16.082865000	-0.503631000
1	13.072461000	15.650106000	-1.391517000
6	11.922461000	17.281688000	-0.570495000
6	11.363359000	17.877390000	0.560792000
1	10.810332000	18.804702000	0.482642000
17	11.727724000	18.100173000	-2.186565000

$^{5}P_{OH,1Cl,NS}$:

26	8.172839000	15.662865000	4.445825000
17	9.962293000	17.017842000	3.169616000
8	11.816610000	14.601388000	3.895978000
1	11.456825000	15.516152000	3.773011000
6	8.355062000	12.846883000	2.892945000
6	7.645717000	11.791208000	2.258771000
1	8.097270000	11.273310000	1.420565000
6	6.397387000	11.435018000	2.735900000
1	5.843878000	10.633084000	2.259403000
6	5.855872000	12.100335000	3.852875000
1	4.893458000	11.816488000	4.260528000
6	6.584671000	13.134139000	4.427413000
6	6.111752000	13.798880000	5.704537000
1	5.036205000	13.621184000	5.838858000
1	6.625878000	13.307536000	6.535688000
6	5.313064000	16.104398000	5.255417000
1	5.510596000	17.134344000	5.573035000
1	4.341669000	15.809650000	5.677583000
6	5.242926000	16.077916000	3.747046000
6	4.021562000	16.034332000	3.090224000
1	3.101224000	15.960074000	3.655832000
6	4.010435000	16.088852000	1.682834000
1	3.070433000	16.057947000	1.142818000
6	5.202960000	16.182795000	0.992458000
1	5.226303000	16.231381000	-0.089919000
6	6.427996000	16.214245000	1.714538000
6	6.865409000	15.704460000	7.148490000

1	6.245015000	15.260611000	7.938119000
1	6.718112000	16.785659000	7.184581000
6	8.400417000	15.457186000	7.375697000
6	8.737956000	14.017819000	7.844691000
6	9.901153000	13.400011000	7.352807000
1	10.488258000	13.926167000	6.610897000
6	10.279887000	12.124109000	7.785615000
1	11.182511000	11.669436000	7.388620000
6	9.498322000	11.432348000	8.719556000
1	9.789800000	10.441810000	9.054478000
6	8.332776000	12.029969000	9.211797000
1	7.710910000	11.503592000	9.929593000
6	7.957548000	13.309309000	8.779445000
1	7.043792000	13.738640000	9.176815000
6	8.870990000	16.472776000	8.456498000
6	8.429612000	16.413550000	9.790797000
1	7.770821000	15.616977000	10.117357000
6	8.843575000	17.370563000	10.726069000
1	8.491105000	17.305720000	11.751168000
6	9.709332000	18.403206000	10.345311000
1	10.032038000	19.142891000	11.071377000
6	10.154065000	18.470448000	9.019238000
1	10.823921000	19.267833000	8.710305000
6	9.735414000	17.515094000	8.084048000
1	10.068262000	17.555394000	7.054780000
7	9.607680000	13.186862000	2.482732000
1	10.224845000	13.781981000	3.028335000
7	7.794652000	13.537068000	3.931460000
7	6.414413000	15.257847000	5.792717000
7	6.438248000	16.155365000	3.082791000
7	7.612715000	16.298793000	1.057009000
1	8.479496000	16.445674000	1.564343000
8	9.058104000	15.740011000	6.146181000
1	7.629958000	16.348173000	0.051346000
1	10.016093000	12.713043000	1.692620000
6	13.287604000	14.582206000	3.936163000
6	13.761330000	15.821405000	4.723243000
6	13.051713000	16.197846000	5.879484000
6	14.881451000	16.576525000	4.344320000
6	15.295070000	17.682203000	5.100706000
6	14.569220000	18.021636000	6.236574000
6	13.448995000	17.299906000	6.642106000
1	12.892590000	17.584027000	7.526796000
17	15.100121000	19.469528000	7.231262000
1	16.159996000	18.260340000	4.800303000
1	15.443731000	16.315871000	3.456224000
1	12.172989000	15.641065000	6.182335000
1	15.370437000	14.065055000	5.713090000
6	14.762963000	13.189489000	5.517693000
6	13.653062000	13.275411000	4.663425000
6	15.104721000	11.980240000	6.138911000
1	15.962271000	11.923505000	6.797650000
6	14.315996000	10.861903000	5.895227000
17	14.750789000	9.272823000	6.705281000
6	13.204537000	10.908822000	5.056311000
1	12.604016000	10.024479000	4.881987000
6	12.880659000	12.122275000	4.440075000
1	12.016260000	12.171665000	3.790514000
6	13.825450000	14.572105000	2.487214000
6	13.112958000	15.231797000	1.471219000
1	12.175092000	15.726687000	1.693915000
6	15.033745000	13.936395000	2.155902000
1	15.601323000	13.412412000	2.915701000
6	15.527932000	13.957531000	0.845305000
1	16.459447000	13.462527000	0.600425000
6	14.793330000	14.620977000	-0.131571000
6	13.590938000	15.260475000	0.156149000
1	13.033962000	15.770131000	-0.620407000
17	15.426799000	14.650814000	-1.854170000

$^{5}TS_{Cl,1Cl,NS}$:

26	7.326135000	13.122023000	5.249101000
17	10.667954000	15.379059000	3.308926000
8	7.922644000	11.196371000	5.230050000
1	8.820828000	10.980961000	5.543949000

6	4.757290000	11.759353000	6.445694000
6	3.448775000	11.747458000	6.996884000
1	3.060563000	10.833381000	7.431458000
6	2.699637000	12.912942000	6.979949000
1	1.701925000	12.919408000	7.406624000
6	3.231160000	14.087123000	6.417959000
1	2.663420000	15.009757000	6.406560000
6	4.513042000	14.041276000	5.873596000
6	5.082679000	15.270477000	5.183147000
1	4.593168000	15.350628000	4.204191000
1	4.789926000	16.162747000	5.750709000
6	6.897708000	15.593935000	3.537292000
1	7.979789000	15.769019000	3.492935000
1	6.390089000	16.509322000	3.199966000
6	6.582244000	14.452488000	2.601182000
6	6.062778000	14.672099000	1.330937000
1	5.802456000	15.673954000	1.012183000
6	5.895270000	13.565135000	0.474964000
1	5.494134000	13.705470000	-0.523388000
6	6.243711000	12.298745000	0.906962000
1	6.129154000	11.432439000	0.265268000
6	6.758471000	12.126194000	2.223364000
6	7.353038000	16.085854000	5.921487000
1	6.831365000	17.021702000	6.163588000
1	8.283181000	16.346523000	5.413450000
6	7.773310000	15.284704000	7.202511000
6	6.665109000	15.205391000	8.284057000
6	6.564495000	14.035050000	9.056399000
1	7.226138000	13.211491000	8.818896000
6	5.627442000	13.928436000	10.089294000
1	5.569746000	13.013242000	10.671482000
6	4.764259000	14.995324000	10.373305000
1	4.035469000	14.914690000	11.173949000
6	4.850423000	16.165586000	9.611397000
1	4.185359000	16.999459000	9.815920000
6	5.792461000	16.269320000	8.578255000
1	5.830441000	17.190973000	8.006976000
6	9.006639000	16.009146000	7.813511000
6	8.932912000	17.319109000	8.321303000
1	7.988419000	17.851671000	8.328049000
6	10.067872000	17.951344000	8.844945000
1	9.987324000	18.960537000	9.238262000
6	11.298980000	17.282140000	8.873121000
1	12.176828000	17.768032000	9.288017000
6	11.381932000	15.977466000	8.368716000
1	12.329540000	15.446838000	8.387310000
6	10.245838000	15.349911000	7.841157000
1	10.291700000	14.343784000	7.444357000
7	5.558007000	10.663751000	6.452023000
1	6.514107000	10.711436000	6.044393000
7	5.257869000	12.905792000	5.881289000
7	6.556576000	15.269460000	4.955146000
7	6.909239000	13.207806000	3.054397000
7	7.117399000	10.911203000	2.702386000
1	7.502746000	10.841512000	3.669747000
8	8.149184000	13.979734000	6.779239000
1	7.008383000	10.093275000	2.124654000
1	5.221457000	9.804476000	6.856063000
6	13.627094000	16.418298000	3.374514000
6	14.046954000	15.395372000	4.324914000
6	13.862855000	14.018085000	4.035005000
6	14.677771000	15.750670000	5.544623000
6	15.120363000	14.772202000	6.433952000
6	14.914269000	13.431361000	6.110353000
6	14.286544000	13.037038000	4.925787000
1	14.120616000	11.989220000	4.711598000
17	15.480505000	12.150303000	7.271435000
1	15.622793000	15.049029000	7.351715000
1	14.863913000	16.792134000	5.774321000
1	13.323823000	13.733097000	3.141094000
1	15.570731000	14.944432000	2.165238000
6	14.870428000	15.385074000	1.467071000
6	13.804610000	16.189666000	1.945151000
6	15.069557000	15.197116000	0.099991000
1	15.897780000	14.599786000	-0.258615000

6	14.187067000	15.802571000	-0.793781000
17	14.433937000	15.550750000	-2.579008000
6	13.116915000	16.593140000	-0.366620000
1	12.434349000	17.031334000	-1.083168000
6	12.933664000	16.789524000	0.998313000
1	12.075808000	17.348386000	1.348292000
6	13.217917000	17.730234000	3.855770000
6	12.495168000	17.863164000	5.070935000
1	12.175241000	16.976653000	5.603206000
6	13.554801000	18.906993000	3.137671000
1	14.140306000	18.837778000	2.229778000
6	13.193523000	20.165684000	3.614862000
1	13.471344000	21.060728000	3.073497000
6	12.477585000	20.247636000	4.809196000
6	12.118248000	19.115653000	5.544877000
1	11.545913000	19.203825000	6.459408000
17	11.992803000	21.887995000	5.430547000

$^5P_{Cl,1Cl,NS}$:

26	8.645261000	13.197236000	5.409418000
17	11.370210000	17.398711000	2.878600000
8	9.711708000	11.489342000	5.567106000
1	10.447966000	11.449429000	6.205756000
6	6.258025000	11.133332000	5.592468000
6	4.912247000	10.713764000	5.415167000
1	4.663990000	9.662471000	5.506011000
6	3.944759000	11.659375000	5.116877000
1	2.915510000	11.349811000	4.967963000
6	4.293457000	13.018615000	5.006112000
1	3.548915000	13.772375000	4.779976000
6	5.622862000	13.378919000	5.210181000
6	6.031917000	14.838004000	5.255839000
1	5.352367000	15.431770000	4.627370000
1	5.893949000	15.182035000	6.285415000
6	7.631047000	15.346596000	3.427210000
1	8.555788000	15.921828000	3.302226000
1	6.812852000	15.962099000	3.025663000
6	7.773029000	14.088300000	2.598999000
6	7.274618000	14.035803000	1.301817000
1	6.688047000	14.856729000	0.907562000
6	7.567858000	12.904322000	0.516052000
1	7.198468000	12.837679000	-0.501954000
6	8.330433000	11.878368000	1.043779000
1	8.574826000	10.998787000	0.458949000
6	8.791476000	11.970747000	2.387259000
6	8.081647000	16.178930000	5.718331000
1	7.403277000	17.037330000	5.822241000
1	8.967165000	16.515333000	5.175954000
6	8.578034000	15.643310000	7.108061000
6	7.448986000	15.628323000	8.178441000
6	7.216341000	14.462617000	8.925268000
1	7.822592000	13.591559000	8.710217000
6	6.226998000	14.428201000	9.916646000
1	6.064137000	13.515361000	10.482365000
6	5.451642000	15.563474000	10.180560000
1	4.683310000	15.538350000	10.947077000
6	5.680037000	16.735937000	9.447876000
1	5.089459000	17.625503000	9.645901000
6	6.672128000	16.768472000	8.460837000
1	6.840295000	17.693734000	7.918548000
6	9.704619000	16.571373000	7.635528000
6	9.715418000	17.961003000	7.416956000
1	8.928534000	18.435607000	6.840086000
6	10.743206000	18.761900000	7.935470000
1	10.731629000	19.832754000	7.754925000
6	11.775766000	18.186548000	8.685680000
1	12.569308000	18.806502000	9.091292000
6	11.773237000	16.802728000	8.908481000
1	12.570150000	16.344569000	9.486922000
6	10.748859000	16.005551000	8.386796000
1	10.747460000	14.933605000	8.540775000
7	7.261938000	10.261309000	5.871567000
1	8.245793000	10.588355000	5.872932000
7	6.586124000	12.458749000	5.485116000
7	7.452286000	15.106759000	4.886042000

7	8.502778000	13.076182000	3.147906000
7	9.527624000	10.985792000	2.959367000
1	9.760476000	11.047311000	3.972671000
8	9.093610000	14.336149000	6.911309000
1	9.726724000	10.147795000	2.436660000
1	7.062449000	9.275329000	5.926455000
6	13.351144000	16.841826000	3.129895000
6	13.295090000	15.667468000	4.093255000
6	12.384390000	14.610569000	3.897049000
6	14.207944000	15.578086000	5.159647000
6	14.207671000	14.471354000	6.019359000
6	13.279013000	13.461929000	5.801311000
6	12.363303000	13.507020000	4.751661000
1	11.628651000	12.721154000	4.621346000
17	13.242316000	12.024446000	6.939304000
1	14.914101000	14.413365000	6.837710000
1	14.933407000	16.363315000	5.327329000
1	11.665787000	14.659561000	3.088572000
1	14.616796000	14.549325000	2.265454000
6	14.451630000	15.261069000	1.467575000
6	13.800685000	16.479913000	1.725408000
6	14.911959000	14.946224000	0.181963000
1	15.413762000	14.005097000	-0.004269000
6	14.711060000	15.863510000	-0.841900000
17	15.301193000	15.460084000	-2.526806000
6	14.072444000	17.084705000	-0.626199000
1	13.923954000	17.783893000	-1.439391000
6	13.623287000	17.386379000	0.659698000
1	13.114455000	18.326803000	0.832488000
6	13.995450000	18.087615000	3.711136000
6	13.466230000	18.676187000	4.878839000
1	12.567514000	18.272621000	5.329764000
6	15.160611000	18.638704000	3.150979000
1	15.607177000	18.199678000	2.268581000
6	15.780756000	19.755001000	3.729295000
1	16.679485000	20.169549000	3.290478000
6	15.221072000	20.312366000	4.871935000
6	14.070235000	19.789643000	5.462056000
1	13.650789000	20.236259000	6.354625000
17	16.018112000	21.775117000	5.630668000

Data for 1$_{Cl,NH}$:
^{5}Re$_{1Cl,NH}$:

6	12.924816000	9.438806000	0.851444000
6	14.273948000	9.240269000	1.386756000
6	15.226933000	10.292693000	1.403732000
6	16.511855000	10.107012000	1.915039000
6	16.857809000	8.855858000	2.423453000
6	15.960205000	7.789247000	2.431084000
6	14.679916000	7.984862000	1.911900000
1	16.246222000	6.828511000	2.841128000
17	18.543524000	8.605237000	3.099712000
1	17.229551000	10.918104000	1.906043000
1	14.963925000	11.259021000	0.990365000
1	13.975021000	7.162441000	1.935063000
6	12.264445000	10.740970000	0.977512000
6	12.486589000	11.576153000	2.103478000
1	13.133624000	11.238824000	2.904372000
6	11.859107000	12.816647000	2.226783000
1	12.030647000	13.430447000	3.102293000
6	10.999088000	13.237008000	1.213644000
17	10.172658000	14.868453000	1.368388000
6	10.745994000	12.455330000	0.087726000
1	10.081047000	12.805937000	-0.691998000
6	11.375247000	11.214668000	-0.022587000
1	11.193530000	10.612742000	-0.904964000
6	12.230356000	8.329439000	0.186290000
6	12.935707000	7.397051000	-0.617255000
6	12.280836000	6.344457000	-1.258572000
1	12.832204000	5.650391000	-1.880882000
1	14.003867000	7.513184000	-0.759032000
6	10.829320000	8.151395000	0.324108000
1	10.260774000	8.820847000	0.960462000
6	10.165869000	7.097539000	-0.307203000
1	9.098554000	6.965745000	-0.178300000
6	10.903346000	6.212545000	-1.091642000
17	10.036274000	4.829355000	-1.928174000
26	7.598730000	9.899387000	5.650715000
17	8.758215000	9.588115000	3.356778000
8	7.442373000	8.040899000	5.795877000
1	7.824795000	7.444938000	5.125208000
6	7.356085000	5.828302000	8.661351000
1	7.608503000	6.422034000	7.776242000
1	6.683530000	5.017857000	8.350279000
1	8.279085000	5.370923000	9.040205000
6	6.368570000	5.796450000	10.978785000
1	7.277983000	5.320025000	11.365198000
1	5.661984000	5.003317000	10.702986000
1	5.920941000	6.382081000	11.792084000
6	7.683683000	7.798776000	10.203925000
1	7.961872000	8.445490000	9.365237000
1	8.603489000	7.349148000	10.599106000
1	7.250658000	8.428912000	10.990497000
6	6.696451000	6.695327000	9.761091000
6	5.354414000	7.299288000	9.243058000
1	4.734299000	6.457771000	8.903288000
1	4.815168000	7.731988000	10.096072000
6	5.689634000	9.764996000	8.395660000
6	5.221046000	10.343699000	9.584387000
1	4.723254000	9.725836000	10.318889000
6	5.389693000	11.706656000	9.823694000
1	5.038816000	12.150295000	10.748823000
6	6.003098000	12.489988000	8.849465000
1	6.141806000	13.555276000	8.991004000
6	6.437425000	11.883757000	7.666937000
6	6.951682000	12.783861000	6.565116000
1	6.072437000	13.235993000	6.090351000
1	7.520089000	13.606521000	7.009920000
6	7.234573000	12.504569000	4.143924000
1	7.951059000	12.144770000	3.400410000
1	7.142290000	13.593304000	4.040199000
6	5.908107000	11.831102000	3.902365000
6	4.873815000	12.483889000	3.233423000
1	4.996156000	13.515786000	2.926290000
6	3.688843000	11.786729000	2.975972000
1	2.861501000	12.273938000	2.472348000

6	3.593786000	10.455608000	3.371579000
1	2.696255000	9.882415000	3.170903000
6	4.666954000	9.832627000	4.035323000
6	4.613452000	7.499176000	3.074442000
1	5.531787000	7.731432000	2.515914000
1	3.772236000	7.797742000	2.432289000
6	4.541299000	5.955975000	3.276615000
6	4.464799000	5.304044000	1.873488000
1	4.435598000	4.210638000	1.955987000
1	5.338072000	5.574866000	1.266865000
1	3.564327000	5.628359000	1.336851000
6	3.280995000	5.560306000	4.081510000
1	3.324147000	5.930347000	5.112234000
1	3.184804000	4.468463000	4.125871000
1	2.372534000	5.959980000	3.612774000
6	5.805854000	5.433615000	3.998252000
1	5.911249000	5.862301000	5.000162000
1	6.708792000	5.681642000	3.425049000
1	5.763401000	4.342170000	4.101287000
6	9.235640000	12.381742000	5.601873000
1	9.434836000	13.449329000	5.751985000
1	9.670293000	12.083019000	4.646960000
6	9.880986000	11.479919000	6.698599000
6	9.782060000	12.034231000	8.140624000
6	9.676113000	11.122314000	9.204843000
1	9.601061000	10.067171000	8.975299000
6	9.658392000	11.563836000	10.531646000
1	9.575298000	10.840710000	11.337275000
6	9.746254000	12.931578000	10.822001000
1	9.730699000	13.276575000	11.850958000
6	9.858301000	13.849240000	9.771589000
1	9.931376000	14.911960000	9.981281000
6	9.881376000	13.404165000	8.442583000
1	9.981421000	14.141788000	7.653356000
6	11.381938000	11.290391000	6.362745000
6	12.232308000	12.398091000	6.190232000
1	11.849938000	13.407459000	6.302710000
6	13.587216000	12.217536000	5.886838000
1	14.228805000	13.083774000	5.757539000
6	14.115164000	10.925505000	5.758711000
1	15.166048000	10.784516000	5.526785000
6	13.275504000	9.819346000	5.937048000
1	13.674281000	8.814027000	5.841849000
6	11.918773000	10.000828000	6.235321000
1	11.262333000	9.150595000	6.368814000
1	4.458102000	8.284636000	7.551141000
7	6.309024000	10.547320000	7.449084000
7	7.763028000	12.132756000	5.491046000
7	5.798306000	10.537798000	4.326050000
1	5.436652000	8.095889000	5.004986000
8	9.207833000	10.215093000	6.624924000
6	5.430539000	8.307830000	8.069607000
1	6.163670000	7.975723000	7.331519000
1	3.664483000	8.168481000	4.917979000
6	4.593193000	8.361859000	4.366645000

$^{5}TS_{OH,1Cl,NH}$:

26	8.366219000	10.991397000	4.649471000
17	8.378521000	12.222477000	2.392213000
8	9.166709000	9.305198000	4.025931000
1	9.377597000	8.625576000	4.693844000
6	10.607056000	7.324035000	8.768919000
1	10.582071000	8.238821000	8.166044000
1	11.104610000	6.542009000	8.182843000
1	11.224940000	7.526911000	9.652560000
6	9.312960000	5.619075000	10.091573000
1	9.922829000	5.831444000	10.978104000
1	9.785921000	4.793788000	9.544712000
1	8.326391000	5.279984000	10.432387000
6	8.525795000	8.007684000	10.023539000
1	8.488095000	8.949906000	9.467041000
1	9.098710000	8.188138000	10.942007000
1	7.502361000	7.739044000	10.311921000
6	9.188905000	6.879625000	9.198487000
6	8.334647000	6.480803000	7.955689000

1	8.853751000	5.649053000	7.459763000
1	7.379687000	6.069613000	8.309911000
6	6.999287000	8.585375000	7.195240000
6	5.756465000	8.218770000	7.741706000
1	5.524953000	7.174521000	7.912937000
6	4.831917000	9.208724000	8.072771000
1	3.867590000	8.941905000	8.491382000
6	5.171790000	10.551565000	7.875990000
1	4.490303000	11.347056000	8.154338000
6	6.410138000	10.855482000	7.304624000
6	6.879461000	12.283820000	7.169614000
1	6.077023000	12.970189000	7.473950000
1	7.706839000	12.420646000	7.867941000
6	6.268068000	13.114941000	4.926728000
1	6.719588000	13.554582000	4.031887000
1	5.654407000	13.882874000	5.419336000
6	5.402466000	11.954361000	4.507524000
6	4.023919000	12.112705000	4.370497000
1	3.565942000	13.068587000	4.596266000
6	3.255463000	11.021477000	3.957351000
1	2.179855000	11.110997000	3.853061000
6	3.894321000	9.812393000	3.700069000
1	3.321325000	8.944610000	3.397803000
6	5.289934000	9.699692000	3.844741000
6	5.607566000	7.705334000	2.245425000
1	6.427191000	7.896766000	1.540624000
1	4.721183000	8.190128000	1.815704000
6	5.328911000	6.171108000	2.272333000
6	5.069705000	5.709424000	0.817358000
1	4.859947000	4.633243000	0.783255000
1	5.940854000	5.906016000	0.181140000
1	4.208856000	6.235937000	0.386456000
6	4.073844000	5.853950000	3.120243000
1	4.195897000	6.152606000	4.168099000
1	3.868008000	4.776492000	3.107511000
1	3.190983000	6.368277000	2.719420000
6	6.544241000	5.398816000	2.833580000
1	6.766932000	5.691960000	3.866651000
1	7.435348000	5.582475000	2.221091000
1	6.350420000	4.319009000	2.830218000
6	8.468153000	13.698892000	5.890869000
1	8.208623000	14.487113000	6.609862000
1	8.527336000	14.154917000	4.903886000
6	9.860780000	13.047754000	6.204370000
6	10.079485000	12.824175000	7.717633000
6	10.619278000	11.611152000	8.173128000
1	10.831098000	10.840463000	7.442728000
6	10.870092000	11.399741000	9.535046000
1	11.288291000	10.453202000	9.863567000
6	10.584061000	12.401600000	10.470097000
1	10.775261000	12.238366000	11.526071000
6	10.052721000	13.620303000	10.028876000
1	9.832782000	14.409068000	10.741870000
6	9.808893000	13.830525000	8.666261000
1	9.418795000	14.792004000	8.346827000
6	10.967754000	13.982609000	5.631598000
6	12.013470000	14.506894000	6.410943000
1	12.070321000	14.280423000	7.468014000
6	12.995283000	15.333231000	5.844035000
1	13.790104000	15.725633000	6.471393000
6	12.951397000	15.652738000	4.484189000
1	13.707125000	16.297065000	4.045894000
6	11.923586000	15.123918000	3.691363000
1	11.883781000	15.351243000	2.630263000
6	10.950758000	14.294004000	4.256254000
1	10.183573000	13.866827000	3.619076000
1	7.772447000	7.034098000	5.952963000
7	7.290910000	9.887585000	6.936627000
7	7.381450000	12.671334000	5.812526000
7	6.032386000	10.771885000	4.244021000
1	7.046352000	8.496243000	3.702228000
8	9.903219000	11.790409000	5.505991000
6	8.064477000	7.558451000	6.874271000
1	8.988742000	8.098390000	6.649579000
1	5.653959000	7.703195000	4.430205000

6	5.965198000	8.366477000	3.610540000
6	10.861310000	8.408704000	2.633053000
6	11.194952000	7.354779000	3.604383000
6	12.469307000	7.285491000	4.218788000
6	12.797496000	6.248460000	5.094308000
6	11.843547000	5.270171000	5.365610000
6	10.571008000	5.301365000	4.794934000
6	10.254183000	6.341063000	3.921551000
1	9.842070000	4.534266000	5.024174000
17	12.271254000	3.901818000	6.499964000
1	13.781843000	6.200806000	5.542803000
1	13.221566000	8.028076000	3.987809000
1	9.264371000	6.376990000	3.485266000
6	11.764532000	9.574351000	2.502405000
6	12.129323000	10.374668000	3.607427000
1	11.611949000	10.259538000	4.550530000
6	13.083572000	11.386655000	3.474721000
1	13.336354000	12.012773000	4.320440000
6	13.679212000	11.591152000	2.231871000
17	14.961006000	12.888259000	2.058989000
6	13.329467000	10.844206000	1.110045000
1	13.800764000	11.025020000	0.152171000
6	12.364940000	9.844217000	1.249650000
1	12.106771000	9.244069000	0.385975000
6	10.039799000	8.070438000	1.451720000
6	10.067573000	6.778852000	0.877071000
6	9.376760000	6.499694000	-0.306538000
1	9.423278000	5.513179000	-0.750413000
1	10.664185000	5.995049000	1.327328000
6	9.287254000	9.077353000	0.803939000
1	9.226248000	10.065701000	1.246532000
6	8.584321000	8.805828000	-0.370781000
1	8.001102000	9.580622000	-0.852769000
6	8.646199000	7.520391000	-0.908994000
17	7.742374000	7.164602000	-2.461681000

$^5P_{OH,1Cl,NH}$:

26	8.199113000	10.889811000	5.007380000
17	9.157806000	9.878712000	2.934345000
8	11.024201000	7.209728000	3.119272000
1	10.545534000	8.051904000	2.921250000
6	11.201229000	7.867759000	7.080288000
1	10.812655000	8.800572000	6.656080000
1	11.395547000	7.175207000	6.251939000
1	12.158412000	8.097134000	7.564691000
6	10.883106000	6.007642000	8.747511000
1	11.801773000	6.290777000	9.275449000
1	11.145683000	5.262573000	7.986201000
1	10.209026000	5.532020000	9.471181000
6	9.913518000	8.280292000	9.215327000
1	9.491627000	9.205661000	8.810286000
1	10.836545000	8.542388000	9.748009000
1	9.206273000	7.873420000	9.948873000
6	10.218113000	7.250228000	8.102704000
6	8.919246000	6.759633000	7.393429000
1	9.207614000	5.943493000	6.716410000
1	8.261132000	6.306585000	8.147863000
6	7.269909000	8.785714000	7.283584000
6	6.401120000	8.393270000	8.314074000
1	6.340914000	7.349893000	8.597058000
6	5.615965000	9.346635000	8.960995000
1	4.933314000	9.051540000	9.750113000
6	5.721015000	10.687888000	8.582698000
1	5.135371000	11.454710000	9.076361000
6	6.595905000	11.034132000	7.550179000
6	6.816376000	12.494332000	7.206307000
1	5.907355000	13.066530000	7.436036000
1	7.600545000	12.868614000	7.871840000
6	6.118493000	13.073604000	4.896505000
1	6.547293000	13.461748000	3.965666000
1	5.479617000	13.862594000	5.318473000
6	5.284877000	11.860788000	4.564876000
6	3.895639000	11.951856000	4.495505000
1	3.402654000	12.884420000	4.743461000
6	3.161217000	10.826995000	4.105954000

1	2.079144000	10.866444000	4.050205000
6	3.846102000	9.656343000	3.797407000
1	3.309058000	8.765811000	3.492040000
6	5.250529000	9.608813000	3.889197000
6	5.901721000	7.996437000	2.024368000
1	6.633287000	8.638209000	1.516429000
1	4.912296000	8.264123000	1.627139000
6	6.179517000	6.509373000	1.646216000
6	6.309863000	6.431292000	0.105483000
1	6.457277000	5.394046000	-0.219619000
1	7.165038000	7.021544000	-0.245539000
1	5.406068000	6.814342000	-0.385451000
6	5.000097000	5.610943000	2.091137000
1	4.857650000	5.630017000	3.178380000
1	5.182683000	4.568792000	1.801289000
1	4.063162000	5.933700000	1.619446000
6	7.488997000	5.998549000	2.291239000
1	7.413026000	5.964826000	3.384736000
1	8.341410000	6.636671000	2.033895000
1	7.709629000	4.981037000	1.944734000
6	8.338959000	13.785046000	5.707385000
1	8.085842000	14.668356000	6.307934000
1	8.380471000	14.093645000	4.660882000
6	9.743350000	13.187215000	6.066530000
6	10.058160000	13.235241000	7.587653000
6	10.658998000	12.121968000	8.197422000
1	10.842870000	11.245146000	7.589957000
6	11.007507000	12.143237000	9.553480000
1	11.471504000	11.270133000	10.002420000
6	10.759224000	13.281872000	10.329403000
1	11.026457000	13.299354000	11.381322000
6	10.164512000	14.400639000	9.732959000
1	9.968582000	15.292199000	10.320997000
6	9.822496000	14.378856000	8.374973000
1	9.376249000	15.266717000	7.939158000
6	10.834216000	14.015636000	5.336286000
6	10.809372000	15.422017000	5.300446000
1	10.005993000	15.972108000	5.779642000
6	11.818102000	16.139677000	4.645356000
1	11.777972000	17.224669000	4.624410000
6	12.872792000	15.463072000	4.020498000
1	13.655961000	16.019044000	3.514484000
6	12.905785000	14.063230000	4.052019000
1	13.717648000	13.527562000	3.569198000
6	11.893078000	13.347385000	4.701904000
1	11.899120000	12.264705000	4.717932000
1	7.438007000	7.185183000	5.902744000
7	7.335486000	10.094029000	6.898581000
7	7.254354000	12.757039000	5.804088000
7	5.958265000	10.707367000	4.279267000
1	7.003559000	8.372551000	3.849493000
8	9.766207000	11.843978000	5.597821000
6	8.114140000	7.773210000	6.539381000
1	8.785732000	8.301398000	5.856651000
1	5.480155000	7.511403000	4.106736000
6	5.959804000	8.321234000	3.541685000
6	12.161714000	6.998423000	2.213942000
6	12.837035000	5.716476000	2.736121000
6	14.187688000	5.439094000	2.469344000
6	14.784923000	4.255796000	2.921105000
6	14.011406000	3.355598000	3.647088000
6	12.670637000	3.595214000	3.932081000
6	12.088061000	4.781260000	3.469298000
1	12.087946000	2.880405000	4.500102000
17	14.786121000	1.803219000	4.250655000
1	15.827812000	4.051267000	2.713121000
1	14.791528000	6.145811000	1.912577000
1	11.048532000	4.988718000	3.682507000
6	13.130975000	8.193667000	2.319511000
6	13.278625000	8.848638000	3.553946000
1	12.675079000	8.536548000	4.397284000
6	14.187421000	9.901088000	3.707730000
1	14.294314000	10.403048000	4.661436000
6	14.952422000	10.285589000	2.609076000
17	16.154908000	11.660543000	2.799746000

6	14.836555000	9.661845000	1.371779000
1	15.439559000	9.979431000	0.530220000
6	13.917644000	8.612830000	1.234356000
1	13.820328000	8.129140000	0.269526000
6	11.615567000	6.828794000	0.778430000
6	11.733267000	5.630682000	0.058664000
6	11.184021000	5.498458000	-1.224989000
1	11.283315000	4.568847000	-1.771622000
1	12.255911000	4.785041000	0.486717000
6	10.927167000	7.903995000	0.182073000
1	10.818143000	8.844342000	0.711986000
6	10.370333000	7.789247000	-1.094875000
1	9.844279000	8.622544000	-1.544004000
6	10.511919000	6.582000000	-1.776562000
17	9.790584000	6.421543000	-3.457076000

$^{5}TS_{Cl,1Cl,NH}$:

26	7.691246000	10.193372000	6.161870000
17	9.714160000	9.308938000	2.493125000
8	8.069330000	8.453760000	5.442878000
1	8.546386000	8.448233000	4.584312000
6	8.613856000	6.136800000	8.347663000
1	8.643456000	6.848659000	7.515532000
1	8.373894000	5.145672000	7.939930000
1	9.618136000	6.083618000	8.787596000
6	7.565310000	5.476881000	10.540664000
1	8.560201000	5.375207000	10.991868000
1	7.272833000	4.496621000	10.142886000
1	6.858333000	5.745359000	11.336228000
6	7.981393000	7.908462000	10.036095000
1	8.059085000	8.686260000	9.269847000
1	8.957349000	7.826758000	10.531628000
1	7.249456000	8.237781000	10.784861000
6	7.578576000	6.548129000	9.422243000
6	6.148988000	6.604467000	8.804207000
1	5.947304000	5.619934000	8.359445000
1	5.422870000	6.721497000	9.621213000
6	5.466213000	9.043033000	8.216317000
6	4.368432000	9.183482000	9.082080000
1	3.832926000	8.300560000	9.409581000
6	3.964265000	10.450529000	9.499681000
1	3.110149000	10.566820000	10.157811000
6	4.671288000	11.570068000	9.052163000
1	4.386932000	12.570556000	9.357313000
6	5.758994000	11.385202000	8.195713000
6	6.615396000	12.563291000	7.787775000
1	6.024476000	13.489003000	7.847333000
1	7.421643000	12.654722000	8.521291000
6	6.359010000	12.905340000	5.349942000
1	6.985220000	13.043527000	4.461882000
1	5.916584000	13.882130000	5.595733000
6	5.272482000	11.918852000	4.999127000
6	3.952096000	12.329455000	4.815777000
1	3.674031000	13.361340000	4.997285000
6	3.004523000	11.388174000	4.395942000
1	1.967996000	11.675214000	4.255745000
6	3.418222000	10.080791000	4.154904000
1	2.714318000	9.330881000	3.811642000
6	4.764674000	9.717008000	4.355262000
6	5.397574000	8.127454000	2.493500000
1	6.231267000	8.760427000	2.157519000
1	4.496186000	8.494311000	1.979364000
6	5.660966000	6.668441000	2.012859000
6	5.897868000	6.713789000	0.482783000
1	6.069859000	5.705146000	0.086536000
1	6.774320000	7.327556000	0.240346000
1	5.030025000	7.138590000	-0.037907000
6	4.435926000	5.767379000	2.298936000
1	4.249669000	5.662320000	3.373855000
1	4.598414000	4.761225000	1.892402000
1	3.529780000	6.177034000	1.834184000
6	6.914038000	6.074696000	2.697245000
1	6.776129000	5.981865000	3.779860000
1	7.791161000	6.709618000	2.524663000
1	7.128462000	5.075237000	2.297408000

6	8.589899000	13.099190000	6.380324000
1	8.531155000	14.120022000	6.783780000
1	8.848505000	13.169932000	5.321759000
6	9.703676000	12.239645000	7.072738000
6	9.823310000	12.522904000	8.599317000
6	9.899263000	11.439872000	9.489758000
1	9.835770000	10.441102000	9.076986000
6	10.047407000	11.648400000	10.866736000
1	10.103693000	10.795371000	11.536776000
6	10.121866000	12.949074000	11.379834000
1	10.234101000	13.113491000	12.447040000
6	10.052573000	14.038270000	10.501165000
1	10.112178000	15.052285000	10.885404000
6	9.909846000	13.826429000	9.124470000
1	9.872537000	14.687802000	8.465094000
6	11.077263000	12.589687000	6.437220000
6	11.445837000	13.903149000	6.094103000
1	10.755412000	14.727214000	6.242738000
6	12.709562000	14.175887000	5.552639000
1	12.973972000	15.197001000	5.294258000
6	13.628924000	13.139077000	5.349143000
1	14.611643000	13.351202000	4.939162000
6	13.268446000	11.826121000	5.684818000
1	13.971515000	11.012703000	5.530199000
6	12.003519000	11.556453000	6.220040000
1	11.705796000	10.545601000	6.469620000
1	5.064734000	7.307482000	7.079650000
7	6.141106000	10.146097000	7.778232000
7	7.251207000	12.432871000	6.444768000
7	5.671443000	10.631555000	4.799860000
1	6.185596000	8.139366000	4.531830000
8	9.402549000	10.875938000	6.843722000
6	5.885495000	7.678434000	7.711769000
1	6.748576000	7.781464000	7.048506000
1	4.487855000	7.606003000	4.403101000
6	5.231170000	8.320056000	4.025837000
6	12.179989000	8.738072000	1.143073000
6	12.812727000	8.102325000	2.300607000
6	14.067193000	8.552808000	2.780557000
6	14.697267000	7.915707000	3.850620000
6	14.060688000	6.832221000	4.452534000
6	12.820438000	6.359223000	4.015395000
6	12.206510000	6.990047000	2.938089000
1	12.339906000	5.527120000	4.513516000
17	14.871567000	6.003290000	5.855752000
1	15.665388000	8.252641000	4.198035000
1	14.573807000	9.373631000	2.289468000
1	11.222807000	6.669827000	2.620660000
6	12.464048000	10.143009000	0.849833000
6	12.589193000	11.091020000	1.897765000
1	12.387018000	10.789780000	2.917563000
6	12.895490000	12.420963000	1.628477000
1	12.969106000	13.138961000	2.435287000
6	13.091163000	12.808368000	0.300420000
17	13.497163000	14.547493000	-0.056429000
6	12.986644000	11.910492000	-0.760066000
1	13.158500000	12.233510000	-1.778626000
6	12.666870000	10.581054000	-0.482514000
1	12.618494000	9.873476000	-1.300074000
6	11.552740000	7.898551000	0.118838000
6	12.015350000	6.580674000	-0.115768000
6	11.464052000	5.796179000	-1.130142000
1	11.836028000	4.796913000	-1.315782000
1	12.837224000	6.182645000	0.465389000
6	10.505594000	8.401026000	-0.694724000
1	10.099584000	9.382302000	-0.486558000
6	9.941006000	7.620594000	-1.698560000
1	9.126815000	8.001846000	-2.301186000
6	10.434747000	6.329048000	-1.902639000
17	9.704358000	5.308486000	-3.221989000

$^{5}P_{Cl,1Cl,NH}$:

26	8.576411000	9.309028000	5.272914000
17	10.837811000	8.454885000	1.355052000
8	8.609106000	7.370807000	5.419809000

1	9.398137000	6.969061000	5.830943000
6	7.924457000	6.126831000	8.782840000
1	8.276124000	6.518070000	7.822137000
1	7.333158000	5.223108000	8.584165000
1	8.800567000	5.830644000	9.373946000
6	6.587343000	6.532459000	10.877116000
1	7.435087000	6.200721000	11.489331000
1	5.952210000	5.659629000	10.678850000
1	6.003533000	7.250271000	11.467468000
6	7.958792000	8.398335000	9.889416000
1	8.354597000	8.867484000	8.982962000
1	8.811782000	8.099134000	10.511814000
1	7.387587000	9.155870000	10.440211000
6	7.082074000	7.170440000	9.555687000
6	5.826471000	7.575747000	8.724157000
1	5.280753000	6.650385000	8.492166000
1	5.155208000	8.158342000	9.369561000
6	6.234439000	9.823984000	7.425795000
6	5.559837000	10.640125000	8.344196000
1	4.938546000	10.194177000	9.109658000
6	5.689738000	12.028686000	8.270997000
1	5.182454000	12.664636000	8.988327000
6	6.468235000	12.587727000	7.257718000
1	6.579816000	13.661889000	7.165558000
6	7.104752000	11.737624000	6.346673000
6	7.826773000	12.342456000	5.156206000
1	7.056277000	12.609347000	4.420997000
1	8.297157000	13.282620000	5.464419000
6	8.574663000	11.372516000	3.012532000
1	9.427459000	10.846069000	2.571442000
1	8.521136000	12.368492000	2.548696000
6	7.324583000	10.588938000	2.700287000
6	6.398769000	11.041826000	1.760697000
1	6.542861000	12.001126000	1.277171000
6	5.291734000	10.239061000	1.461963000
1	4.548813000	10.570116000	0.744541000
6	5.162942000	9.007873000	2.099404000
1	4.321552000	8.360857000	1.881532000
6	6.126613000	8.591766000	3.038267000
6	5.945165000	6.074541000	2.694253000
1	6.779062000	6.166833000	1.982652000
1	5.021262000	6.161652000	2.103721000
6	5.991663000	4.642842000	3.309917000
6	5.710278000	3.627784000	2.174788000
1	5.775366000	2.598578000	2.549411000
1	6.439031000	3.738199000	1.361692000
1	4.707003000	3.772908000	1.754134000
6	4.913588000	4.477268000	4.407157000
1	5.118292000	5.115124000	5.274278000
1	4.884325000	3.438142000	4.758003000
1	3.916872000	4.731661000	4.023434000
6	7.390677000	4.353187000	3.904999000
1	7.671556000	5.091461000	4.663349000
1	8.155085000	4.376439000	3.116877000
1	7.411900000	3.355507000	4.362394000
6	10.251354000	11.811802000	4.766945000
1	10.406175000	12.898441000	4.720154000
1	10.845270000	11.362430000	3.968241000
6	10.773871000	11.177112000	6.098555000
6	10.419732000	11.988574000	7.371338000
6	10.249486000	11.286618000	8.577805000
1	10.318857000	10.206407000	8.552819000
6	9.986412000	11.958486000	9.775741000
1	9.859317000	11.392214000	10.693756000
6	9.885121000	13.356347000	9.794375000
1	9.677756000	13.880177000	10.722329000
6	10.057818000	14.069634000	8.603492000
1	9.985264000	15.153233000	8.601503000
6	10.327566000	13.392204000	7.405423000
1	10.464340000	13.978958000	6.503202000
6	12.324840000	11.109390000	5.984914000
6	13.123422000	12.266605000	5.936560000
1	12.669030000	13.248305000	6.018921000
6	14.514803000	12.172996000	5.804406000
1	15.114184000	13.078111000	5.773928000

6	15.133077000	10.917660000	5.722294000
1	16.212176000	10.844277000	5.628204000
6	14.345416000	9.760109000	5.771648000
1	14.813898000	8.781736000	5.715260000
6	12.953512000	9.857199000	5.899170000
1	12.328857000	8.973773000	5.942524000
1	5.190471000	8.127368000	6.743306000
7	7.009769000	10.387160000	6.446611000
7	8.825182000	11.465752000	4.480404000
7	7.183906000	9.396728000	3.346559000
1	6.884426000	7.116906000	4.370534000
8	10.234431000	9.867063000	6.171243000
6	6.065538000	8.317330000	7.385861000
1	6.917058000	7.882231000	6.849841000
1	5.115197000	7.232186000	4.333695000
6	6.021190000	7.243066000	3.711222000
6	12.427682000	8.456478000	0.038294000
6	13.222698000	7.220347000	0.425912000
6	14.612641000	7.286373000	0.624003000
6	15.354311000	6.135886000	0.924749000
6	14.689122000	4.920566000	1.027273000
6	13.312229000	4.812794000	0.832534000
6	12.587627000	5.965544000	0.528385000
1	12.813159000	3.855889000	0.918795000
17	15.646597000	3.413209000	1.428529000
1	16.424855000	6.198594000	1.073449000
1	15.135004000	8.229785000	0.535676000
1	11.516457000	5.891237000	0.386328000
6	13.132746000	9.772688000	0.320724000
6	13.505366000	10.115126000	1.636944000
1	13.241737000	9.463394000	2.461058000
6	14.196903000	11.296666000	1.907656000
1	14.470262000	11.550564000	2.924308000
6	14.523084000	12.136986000	0.843505000
17	15.426378000	13.691912000	1.189591000
6	14.185948000	11.830818000	-0.469014000
1	14.455610000	12.492799000	-1.282227000
6	13.487190000	10.643087000	-0.724635000
1	13.234827000	10.401865000	-1.748753000
6	11.770328000	8.375739000	-1.329985000
6	12.214647000	7.451164000	-2.290989000
6	11.649954000	7.414677000	-3.573158000
1	12.001480000	6.699151000	-4.305698000
1	13.011898000	6.758206000	-2.056817000
6	10.745821000	9.276166000	-1.686715000
1	10.383839000	9.993588000	-0.960568000
6	10.170404000	9.247862000	-2.957366000
1	9.377460000	9.937421000	-3.217959000
6	10.634897000	8.311742000	-3.881055000
17	9.883211000	8.267530000	-5.549299000

Data for $1_{Cl,NE}$:

$^{5}Re_{1Cl,NE}$:

26	6.111923000	13.179198000	4.086169000
17	7.940986000	13.315341000	2.331739000
8	5.316759000	11.589319000	3.466321000
1	5.790913000	11.010950000	2.839794000
6	3.365275000	12.626808000	5.433360000
6	2.247790000	12.844403000	6.235092000
1	1.455606000	12.106880000	6.273772000
6	2.173888000	14.030325000	6.979038000
1	1.315487000	14.231223000	7.610150000
6	3.217333000	14.954361000	6.898078000
1	3.185197000	15.879690000	7.462152000
6	4.319482000	14.675047000	6.077725000
6	5.481768000	15.642649000	5.989790000
1	5.093065000	16.643684000	5.764612000
1	5.948653000	15.707864000	6.977806000
6	6.528887000	16.209470000	3.815144000
1	7.464683000	16.052884000	3.269308000
1	6.494961000	17.259468000	4.132968000
6	5.384755000	15.894741000	2.880892000
6	4.731233000	16.862869000	2.112658000
1	5.001276000	17.907330000	2.215723000
6	3.737093000	16.459933000	1.214336000
1	3.219484000	17.195121000	0.608661000
6	3.415354000	15.101047000	1.108967000
1	2.650255000	14.756940000	0.424092000
6	4.096178000	14.185902000	1.911755000
6	7.890842000	15.089462000	5.576321000
1	8.139513000	15.920294000	6.248491000
1	8.595628000	15.095343000	4.743896000
6	8.018000000	13.700065000	6.289934000
6	7.478503000	13.721798000	7.744125000
6	6.520617000	12.782615000	8.155933000
1	6.152383000	12.068883000	7.430564000
6	6.047516000	12.774230000	9.474855000
1	5.306313000	12.039215000	9.774071000
6	6.525178000	13.705864000	10.403451000
1	6.158266000	13.700721000	11.424956000
6	7.486531000	14.643794000	10.003739000
1	7.870278000	15.368068000	10.715698000
6	7.961959000	14.648122000	8.687528000
1	8.721960000	15.370622000	8.407053000
6	9.501367000	13.264168000	6.332501000
6	10.552569000	14.187415000	6.470350000
1	10.351221000	15.252318000	6.519624000
6	11.882562000	13.751093000	6.540041000
1	12.681174000	14.479533000	6.640751000
6	12.180881000	12.385519000	6.474604000
1	13.210809000	12.047128000	6.528839000
6	11.138566000	11.459218000	6.335660000
1	11.358959000	10.397566000	6.279184000
6	9.811480000	11.895399000	6.265220000
1	9.003200000	11.184746000	6.142856000
7	4.374754000	13.529964000	5.365178000
7	6.530341000	15.280229000	4.984953000
7	5.058898000	14.583939000	2.774873000
8	7.274627000	12.749746000	5.516916000
1	3.499705000	11.745954000	4.818433000
1	3.897105000	13.122090000	1.890838000
6	12.901300000	15.940940000	2.775743000
6	14.063996000	16.365429000	3.563487000
6	11.739011000	16.825923000	2.657642000
6	12.906970000	14.636695000	2.106531000
6	14.829128000	15.431569000	4.309936000
6	15.933510000	15.830266000	5.064796000
6	16.289265000	17.178036000	5.073151000
6	15.574127000	18.134312000	4.354001000
6	14.467583000	17.725173000	3.608531000
1	13.922388000	18.464031000	3.033175000
1	15.876151000	19.174345000	4.364544000
17	17.740503000	17.709395000	6.058959000
1	16.497077000	15.105726000	5.639676000
1	14.538333000	14.387810000	4.317252000
6	14.106700000	14.081462000	1.588563000

6	11.715967000	13.880246000	1.952540000
6	11.718887000	12.636949000	1.318579000
6	12.922578000	12.138397000	0.824325000
6	14.120062000	12.841983000	0.947904000
1	15.042230000	12.440197000	0.546197000
17	12.934434000	10.507535000	-0.013943000
1	10.798263000	12.073936000	1.227067000
1	10.778376000	14.248031000	2.352418000
6	10.956202000	16.865062000	1.474060000
6	11.353862000	17.681246000	3.723661000
6	10.252091000	18.531105000	3.617596000
6	9.521832000	18.535962000	2.430097000
6	9.855623000	17.714941000	1.354014000
1	9.280176000	17.742773000	0.436948000
1	11.228669000	16.240697000	0.631709000
1	11.912845000	17.662589000	4.651654000
1	9.967349000	19.168961000	4.445343000
17	8.078601000	19.659270000	2.279033000
1	15.032621000	14.638738000	1.668264000

^{5}TS$_{OH,1Cl,NE}$:

26	6.926953000	12.526308000	4.436808000
17	9.422510000	12.434713000	3.666688000
8	6.302540000	10.685421000	4.017479000
1	5.762984000	10.325427000	4.749869000
6	3.680429000	12.369980000	4.364753000
6	2.365500000	12.825851000	4.416525000
1	1.548099000	12.156512000	4.177397000
6	2.133618000	14.160478000	4.774217000
1	1.123815000	14.552682000	4.819473000
6	3.222982000	14.982585000	5.064680000
1	3.076342000	16.021910000	5.337443000
6	4.522496000	14.456577000	5.002512000
6	5.707835000	15.328453000	5.359103000
1	5.579505000	16.312127000	4.887226000
1	5.684112000	15.501304000	6.439536000
6	7.611695000	15.407522000	3.779095000
1	8.668309000	15.126094000	3.728029000
1	7.547862000	16.503349000	3.830787000
6	6.937439000	14.898144000	2.526133000
6	6.708138000	15.702039000	1.405218000
1	6.970012000	16.753466000	1.432486000
6	6.140463000	15.132161000	0.260360000
1	5.956930000	15.739961000	-0.618454000
6	5.806017000	13.773170000	0.266333000
1	5.361826000	13.299959000	-0.600707000
6	6.049342000	13.028292000	1.421091000
6	7.998683000	14.722807000	6.144088000
1	8.012271000	15.685681000	6.672056000
1	8.986257000	14.545220000	5.716759000
6	7.690600000	13.526098000	7.112769000
6	6.579441000	13.867047000	8.144581000
6	5.460700000	13.029535000	8.271598000
1	5.390068000	12.168920000	7.619466000
6	4.460833000	13.303866000	9.214058000
1	3.603646000	12.641866000	9.296213000
6	4.562747000	14.423983000	10.046924000
1	3.788123000	14.638796000	10.776454000
6	5.678471000	15.264379000	9.933471000
1	5.773454000	16.133469000	10.577460000
6	6.678850000	14.985044000	8.995091000
1	7.543210000	15.639369000	8.939669000
6	8.971048000	13.163567000	7.905250000
6	9.920231000	14.124733000	8.296482000
1	9.797406000	15.167410000	8.022869000
6	11.048839000	13.755828000	9.041359000
1	11.772370000	14.512887000	9.328430000
6	11.245397000	12.420671000	9.410302000
1	12.119799000	12.134631000	9.986405000
6	10.304988000	11.456068000	9.024477000
1	10.450817000	10.415713000	9.299756000
6	9.180690000	11.825581000	8.278803000
1	8.459293000	11.083234000	7.960682000
7	4.738245000	13.166539000	4.658630000
7	7.040482000	14.765519000	4.998232000

7	6.608227000	13.581382000	2.521389000
8	7.288880000	12.411318000	6.316455000
6	6.844818000	8.630932000	3.329211000
6	8.051876000	8.408296000	4.156906000
6	9.277696000	8.019761000	3.569646000
6	8.007678000	8.534074000	5.566070000
6	9.130351000	8.283890000	6.354798000
6	10.316643000	7.901593000	5.729637000
6	10.409984000	7.764230000	4.348016000
1	11.338250000	7.457877000	3.882240000
17	11.792864000	7.566751000	6.759216000
1	9.081585000	8.391365000	7.430953000
1	7.093573000	8.853149000	6.051024000
1	9.346341000	7.892369000	2.496951000
1	8.470229000	10.518970000	2.229188000
6	7.946216000	9.931423000	1.483104000
6	7.026023000	8.939172000	1.892366000
6	8.160513000	10.201501000	0.129781000
1	8.858912000	10.972910000	-0.170622000
6	7.452532000	9.469472000	-0.821871000
17	7.731574000	9.818144000	-2.600366000
6	6.540232000	8.480401000	-0.464877000
1	6.010235000	7.914545000	-1.220919000
6	6.329991000	8.221764000	0.892953000
1	5.637962000	7.436161000	1.170262000
6	5.565999000	8.016784000	3.755841000
6	5.524177000	6.912423000	4.637754000
1	6.444447000	6.486051000	5.016217000
6	4.334246000	8.510952000	3.258141000
1	4.337665000	9.359095000	2.585443000
6	3.117212000	7.939599000	3.630116000
1	2.183796000	8.333297000	3.247409000
6	3.126938000	6.855440000	4.507291000
6	4.311301000	6.328403000	5.014534000
1	4.297465000	5.475958000	5.682151000
17	1.534151000	6.099798000	4.999473000
1	3.923920000	11.357927000	4.073145000
1	5.806034000	11.976147000	1.489431000

$^{5}P_{OH,1Cl,NE}$:

26	7.285149000	12.578445000	4.586658000
17	8.570239000	10.815134000	3.288614000
8	6.210033000	8.696735000	2.712630000
1	7.005976000	9.222870000	2.977995000
6	5.228040000	11.869046000	2.289789000
6	4.002619000	11.899685000	1.625221000
1	3.880240000	11.364212000	0.692070000
6	2.951007000	12.633863000	2.185773000
1	1.988305000	12.683215000	1.689747000
6	3.155892000	13.309032000	3.393079000
1	2.361058000	13.888535000	3.847995000
6	4.404396000	13.232741000	4.018742000
6	4.668719000	13.885064000	5.360642000
1	4.000259000	14.747132000	5.500091000
1	4.436444000	13.166552000	6.152162000
6	6.428125000	15.502746000	4.743731000
1	6.217924000	16.405721000	5.333577000
1	5.770675000	15.534745000	3.866327000
6	7.863742000	15.514493000	4.260976000
6	8.576828000	16.704609000	4.085167000
1	8.121701000	17.651152000	4.352928000
6	9.874028000	16.653938000	3.564485000
1	10.440523000	17.567253000	3.422330000
6	10.432811000	15.413261000	3.235663000
1	11.434998000	15.337510000	2.832481000
6	9.675594000	14.260875000	3.444533000
6	6.597619000	14.312771000	6.912689000
1	5.881337000	14.842614000	7.554800000
1	7.521142000	14.896729000	6.891081000
6	6.966301000	12.892336000	7.473569000
6	5.724135000	12.123706000	8.004588000
6	5.511560000	10.793658000	7.607228000
1	6.215690000	10.345255000	6.917873000
6	4.418694000	10.063823000	8.092710000
1	4.276492000	9.035605000	7.773663000

6	3.517088000	10.653065000	8.986662000
1	2.668813000	10.089800000	9.362729000
6	3.723161000	11.976935000	9.396894000
1	3.034490000	12.444239000	10.094346000
6	4.818754000	12.702564000	8.914663000
1	4.963492000	13.721009000	9.260748000
6	7.964217000	13.057668000	8.648077000
6	7.867711000	14.100065000	9.587821000
1	7.087351000	14.849770000	9.506444000
6	8.780403000	14.196561000	10.646884000
1	8.691713000	15.012023000	11.358424000
6	9.801526000	13.249823000	10.787284000
1	10.507706000	13.324068000	11.608362000
6	9.904665000	12.207875000	9.856312000
1	10.694611000	11.468938000	9.953044000
6	8.995695000	12.115617000	8.796629000
1	9.075195000	11.323417000	8.062639000
7	5.423919000	12.522900000	3.461862000
7	6.096363000	14.265964000	5.505726000
7	8.418155000	14.313333000	3.947960000
8	7.592893000	12.150040000	6.429463000
6	6.434872000	7.256494000	2.884508000
6	7.720463000	6.855063000	2.132209000
6	8.574893000	5.839823000	2.589414000
6	8.019713000	7.495108000	0.916856000
6	9.149294000	7.140123000	0.173699000
6	9.973969000	6.128023000	0.659925000
6	9.709297000	5.467786000	1.854520000
1	10.365892000	4.685470000	2.214453000
17	11.460177000	5.652627000	-0.307862000
1	9.377297000	7.641619000	-0.758715000
1	7.369142000	8.283563000	0.558624000
1	8.370115000	5.332373000	3.524653000
1	3.983601000	8.344871000	2.366580000
6	4.020033000	7.307214000	2.064381000
6	5.211553000	6.584559000	2.234900000
6	2.888888000	6.706494000	1.498092000
1	1.973904000	7.270812000	1.365669000
6	2.965600000	5.372786000	1.109389000
17	1.487283000	4.575676000	0.365109000
6	4.128258000	4.623640000	1.263278000
1	4.167962000	3.587909000	0.949732000
6	5.251496000	5.240861000	1.826702000
1	6.161780000	4.662861000	1.937204000
6	6.531339000	6.959865000	4.398007000
6	7.536805000	7.598486000	5.150978000
1	8.243128000	8.265558000	4.667733000
6	5.632953000	6.115098000	5.066370000
1	4.847001000	5.610468000	4.519476000
6	5.729467000	5.901049000	6.449399000
1	5.031617000	5.243658000	6.953080000
6	6.736536000	6.548039000	7.154686000
6	7.646729000	7.398735000	6.529914000
1	8.424064000	7.894297000	7.098191000
17	6.876870000	6.272621000	8.964015000
1	6.074375000	11.313204000	1.909591000
1	10.050204000	13.270657000	3.217475000

$^{5}TS_{Cl,1Cl,NE}$:

26	7.223899000	14.339812000	4.446303000
17	9.718955000	15.948683000	3.764422000
8	7.171546000	13.583547000	2.648990000
1	7.264987000	12.617563000	2.540960000
6	4.283760000	13.264648000	4.175297000
6	2.918935000	13.156104000	4.437004000
1	2.271748000	12.631178000	3.744775000
6	2.410497000	13.748670000	5.600648000
1	1.352090000	13.692369000	5.829704000
6	3.281995000	14.422286000	6.461009000
1	2.914284000	14.896845000	7.364104000
6	4.646811000	14.481786000	6.147256000
6	5.647029000	15.118303000	7.089304000
1	5.164797000	15.939579000	7.639114000
1	5.925088000	14.364409000	7.832133000
6	6.827848000	17.019477000	6.017475000

1	7.856599000	17.381085000	5.907986000
1	6.342273000	17.640071000	6.785108000
6	6.136739000	17.206100000	4.684821000
6	5.396346000	18.351747000	4.373469000
1	5.240285000	19.115896000	5.126734000
6	4.867617000	18.493770000	3.085772000
1	4.291994000	19.375210000	2.825771000
6	5.086493000	17.482822000	2.141620000
1	4.690695000	17.559352000	1.135980000
6	5.820473000	16.358009000	2.520555000
6	8.132356000	15.274922000	7.217080000
1	8.015237000	15.566150000	8.270541000
1	8.935191000	15.874013000	6.784855000
6	8.562794000	13.769446000	7.066177000
6	7.835148000	12.827841000	8.069958000
6	7.268378000	11.631443000	7.601329000
1	7.330649000	11.422616000	6.540845000
6	6.639005000	10.739244000	8.478510000
1	6.208473000	9.819619000	8.092827000
6	6.564215000	11.028074000	9.846414000
1	6.075292000	10.339037000	10.528070000
6	7.129955000	12.215734000	10.328003000
1	7.082131000	12.451477000	11.386990000
6	7.762480000	13.103632000	9.449127000
1	8.202154000	14.009971000	9.852278000
6	10.087517000	13.666874000	7.347693000
6	10.714222000	14.365877000	8.395786000
1	10.139238000	15.017566000	9.045499000
6	12.092149000	14.242553000	8.618069000
1	12.556456000	14.795507000	9.429250000
6	12.868063000	13.413804000	7.798685000
1	13.935359000	13.316380000	7.971962000
6	12.252503000	12.712159000	6.753260000
1	12.843361000	12.067902000	6.108683000
6	10.876039000	12.840393000	6.530820000
1	10.391736000	12.319910000	5.714250000
7	5.126575000	13.908620000	5.018443000
7	6.900111000	15.593044000	6.436217000
7	6.334527000	16.229627000	3.766834000
8	8.292900000	13.363723000	5.734769000
1	4.744698000	12.864687000	3.280546000
1	6.017590000	15.523616000	1.858793000
6	11.459915000	15.568924000	2.125442000
6	12.568670000	16.316905000	2.778656000
6	10.788772000	16.184485000	0.949028000
6	11.537401000	14.078775000	2.168684000
6	12.996668000	15.958478000	4.079006000
6	14.059084000	16.614068000	4.696665000
6	14.710161000	17.635348000	4.002190000
6	14.332354000	18.012461000	2.718307000
6	13.258697000	17.350921000	2.111260000
1	12.981838000	17.629705000	1.102796000
1	14.860120000	18.797818000	2.192100000
17	16.105916000	18.501268000	4.807632000
1	14.366042000	16.339800000	5.698062000
1	12.468866000	15.181287000	4.618134000
6	12.795659000	13.441579000	2.238172000
6	10.378742000	13.273968000	2.069483000
6	10.479989000	11.881692000	2.046909000
6	11.741843000	11.291653000	2.112490000
6	12.905565000	12.047195000	2.205088000
1	13.877329000	11.571666000	2.247041000
17	11.869581000	9.465275000	2.080877000
1	9.587346000	11.270731000	1.993777000
1	9.385540000	13.717582000	2.071004000
6	10.584193000	15.447252000	-0.236689000
6	10.373904000	17.536921000	0.973076000
6	9.778741000	18.130840000	-0.137839000
6	9.597979000	17.364613000	-1.290706000
6	9.991635000	16.032977000	-1.360839000
1	9.848879000	15.458033000	-2.267030000
1	10.907140000	14.416390000	-0.297808000
1	10.486158000	18.111565000	1.883808000
1	9.453856000	19.162978000	-0.102959000
17	8.815921000	18.138976000	-2.751852000

1	13.701907000	14.031389000	2.292148000

$^{5}P_{Cl,1Cl,NE}$:

26	7.706271000	13.471028000	4.221017000
17	11.852906000	16.512154000	3.678807000
8	7.592445000	12.146367000	2.790815000
1	7.874430000	11.229548000	2.975711000
6	4.646657000	12.978404000	4.153507000
6	3.291263000	13.244043000	4.343004000
1	2.544271000	12.653328000	3.826631000
6	2.925432000	14.289501000	5.200944000
1	1.880317000	14.528549000	5.363212000
6	3.924952000	15.027498000	5.842367000
1	3.671450000	15.845816000	6.507020000
6	5.269017000	14.702330000	5.619810000
6	6.391985000	15.397714000	6.359282000
1	6.071210000	16.408458000	6.653386000
1	6.572934000	14.841259000	7.283867000
6	7.766242000	16.622962000	4.698864000
1	8.827645000	16.866886000	4.574264000
1	7.287378000	17.507915000	5.143545000
6	7.198416000	16.375694000	3.317359000
6	6.659674000	17.410414000	2.543538000
1	6.570102000	18.406589000	2.962354000
6	6.245230000	17.142576000	1.234954000
1	5.826724000	17.932717000	0.621461000
6	6.372788000	15.841617000	0.731758000
1	6.060247000	15.596876000	-0.276357000
6	6.903313000	14.851181000	1.558131000
6	8.884888000	15.283013000	6.471368000
1	8.810840000	15.897144000	7.379887000
1	9.733363000	15.653267000	5.893089000
6	9.203991000	13.778830000	6.797751000
6	8.386640000	13.210810000	7.991852000
6	7.911767000	11.890111000	7.913806000
1	8.100520000	11.336101000	7.002754000
6	7.212046000	11.308459000	8.977360000
1	6.858025000	10.285132000	8.892450000
6	6.967823000	12.039946000	10.146548000
1	6.422646000	11.592405000	10.971691000
6	7.434872000	13.356358000	10.239583000
1	7.251692000	13.937197000	11.138666000
6	8.139825000	13.933966000	9.174977000
1	8.487129000	14.956456000	9.280853000
6	10.714357000	13.693566000	7.161316000
6	11.291232000	14.488422000	8.169392000
1	10.681225000	15.180176000	8.740225000
6	12.658592000	14.399845000	8.458254000
1	13.084648000	15.024943000	9.237430000
6	13.473839000	13.509036000	7.748275000
1	14.533192000	13.437423000	7.974960000
6	12.907838000	12.711300000	6.745967000
1	13.528847000	12.014269000	6.190589000
6	11.541051000	12.806579000	6.454317000
1	11.090683000	12.204526000	5.675426000
7	5.612467000	13.692302000	4.782769000
7	7.676325000	15.448625000	5.608329000
7	7.309443000	15.117402000	2.825354000
8	8.950613000	13.018446000	5.629247000
1	5.006002000	12.204011000	3.486479000
1	7.010669000	13.817195000	1.254219000
6	12.781193000	15.829749000	1.955499000
6	14.256700000	16.057789000	2.234791000
6	12.194520000	16.711260000	0.866170000
6	12.385041000	14.365771000	1.862467000
6	14.846745000	15.534730000	3.404290000
6	16.210651000	15.687847000	3.653497000
6	16.990551000	16.363729000	2.714954000
6	16.450626000	16.881803000	1.544619000
6	15.077588000	16.725859000	1.310155000
1	14.661785000	17.119983000	0.392313000
1	17.073825000	17.396057000	0.823988000
17	18.780782000	16.569942000	3.036483000
1	16.652626000	15.291289000	4.558792000
1	14.233763000	15.017413000	4.132516000

6	13.344324000	13.387189000	1.544184000
6	11.041637000	13.966505000	2.008507000
6	10.657237000	12.631157000	1.860250000
6	11.641505000	11.695873000	1.547204000
6	12.976705000	12.044942000	1.381377000
1	13.722226000	11.299645000	1.134047000
17	11.159203000	9.935832000	1.352199000
1	9.614667000	12.350140000	2.011419000
1	10.284694000	14.697210000	2.266511000
6	11.658118000	16.151080000	-0.305945000
6	12.228593000	18.116131000	0.985438000
6	11.733785000	18.938593000	-0.026969000
6	11.205419000	18.343915000	-1.172775000
6	11.161289000	16.964695000	-1.333366000
1	10.752492000	16.521708000	-2.232727000
1	11.630943000	15.077429000	-0.436299000
1	12.628216000	18.569916000	1.884081000
1	11.756896000	20.015927000	0.078197000
17	10.550298000	19.416799000	-2.503640000
1	14.382554000	13.660309000	1.407618000

Data for 1_{Br}:
$^{5}Re_{1Br}$:

26	6.143256000	12.473297000	3.458852000
8	4.827007000	11.083811000	3.210732000
1	5.139164000	10.238326000	2.837116000
6	3.732212000	8.852996000	6.299167000
1	4.650622000	9.339046000	5.950775000
1	3.223876000	8.419657000	5.428195000
1	4.016275000	8.031585000	6.967759000
6	1.531106000	9.128608000	7.506412000
1	1.782653000	8.315639000	8.197504000
1	0.987416000	8.694156000	6.658025000
1	0.854549000	9.817476000	8.028005000
6	3.552733000	10.442352000	8.255451000
1	4.439056000	11.005337000	7.944774000
1	3.879086000	9.638772000	8.927019000
1	2.902294000	11.114716000	8.828735000
6	2.812776000	9.854499000	7.034962000
6	2.371500000	10.984652000	6.054510000
1	1.797460000	10.519973000	5.243275000
1	1.684725000	11.664496000	6.569165000
6	3.905541000	12.972349000	5.794091000
6	3.357674000	13.710265000	6.879059000
1	2.537749000	13.300964000	7.451025000
6	3.897720000	14.939193000	7.214405000
1	3.485424000	15.499897000	8.046532000
6	4.988445000	15.448029000	6.493304000
1	5.447787000	16.392308000	6.758437000
6	5.479778000	14.704118000	5.424668000
6	6.613052000	15.283270000	4.604658000
1	6.189921000	16.054270000	3.951199000
1	7.316107000	15.791322000	5.274063000
6	7.540916000	14.893906000	2.366452000
1	8.216746000	14.230666000	1.818600000
1	7.995147000	15.891530000	2.410128000
6	6.221011000	14.935279000	1.641284000
6	5.936227000	15.933014000	0.717993000
1	6.632479000	16.747264000	0.560507000
6	4.733721000	15.842435000	-0.004430000
1	4.482205000	16.598216000	-0.740725000
6	3.870692000	14.784013000	0.215985000
1	2.955602000	14.699366000	-0.351867000
6	4.194696000	13.798478000	1.193551000
6	2.153137000	12.495495000	0.682365000
1	2.404317000	12.238082000	-0.359333000
1	1.562034000	13.420348000	0.651761000
6	1.247431000	11.380464000	1.264418000
6	0.032585000	11.246690000	0.313864000
1	-0.657960000	10.481660000	0.686726000
1	0.348929000	10.954355000	-0.695000000
1	-0.518595000	12.192382000	0.239047000
6	0.751125000	11.781190000	2.671676000
1	1.588223000	11.946123000	3.357071000
1	0.112749000	10.994350000	3.091079000
1	0.162403000	12.706085000	2.626869000
6	1.991336000	10.025189000	1.319741000
1	2.824261000	10.044385000	2.029655000
1	2.385575000	9.755391000	0.331929000
1	1.302360000	9.232600000	1.635547000
6	8.654804000	13.821738000	4.300743000
1	9.224127000	14.652049000	4.732753000
1	9.223312000	13.422514000	3.459810000
6	8.441670000	12.640545000	5.298113000
6	8.117427000	13.072795000	6.747493000
6	7.267717000	12.266963000	7.524359000
1	6.805004000	11.404142000	7.061600000
6	7.017195000	12.573394000	8.866032000
1	6.357755000	11.937153000	9.448231000
6	7.612537000	13.694642000	9.458436000
1	7.417713000	13.933886000	10.498989000
6	8.465409000	14.500399000	8.696449000
1	8.937777000	15.369950000	9.142960000
6	8.719574000	14.189217000	7.354049000
1	9.397872000	14.826417000	6.796960000
6	9.724213000	11.773872000	5.336448000

6	10.990504000	12.354771000	5.529491000
1	11.091118000	13.428443000	5.651379000
6	12.140775000	11.558351000	5.575313000
1	13.110556000	12.024170000	5.720922000
6	12.042101000	10.168102000	5.436715000
1	12.933830000	9.550451000	5.474229000
6	10.783991000	9.583231000	5.249943000
1	10.695534000	8.506533000	5.141131000
6	9.633808000	10.380463000	5.198393000
1	8.659015000	9.935645000	5.043813000
7	3.458865000	11.741308000	5.431281000
1	3.917094000	11.319591000	4.607209000
7	4.943808000	13.510067000	5.054379000
7	7.359180000	14.317725000	3.734631000
7	5.371144000	13.901223000	1.902142000
7	3.382056000	12.740071000	1.448132000
1	3.752187000	12.019113000	2.081912000
8	7.357672000	11.860141000	4.768230000
6	12.248738000	12.733616000	-1.971032000
6	13.676191000	12.650478000	-2.299992000
6	14.409387000	13.796093000	-2.705437000
6	15.766462000	13.720812000	-3.022104000
6	16.409423000	12.487514000	-2.929302000
6	15.736326000	11.332597000	-2.534152000
6	14.377977000	11.418943000	-2.226094000
1	16.258360000	10.386717000	-2.457564000
17	18.192873000	12.381130000	-3.339625000
1	16.306064000	14.603257000	-3.343427000
1	13.903730000	14.750118000	-2.796130000
1	13.857622000	10.526030000	-1.900568000
6	11.714345000	13.937217000	-1.328601000
6	12.498890000	14.702085000	-0.425199000
1	13.504599000	14.378944000	-0.184361000
6	11.996491000	15.849790000	0.189139000
1	12.606427000	16.412410000	0.885179000
6	10.692766000	16.248915000	-0.100243000
17	10.026410000	17.759085000	0.701604000
6	9.879775000	15.534958000	-0.979260000
1	8.872139000	15.867099000	-1.196942000
6	10.391954000	14.386579000	-1.584562000
1	9.767863000	13.842576000	-2.283246000
6	11.360192000	11.610693000	-2.288027000
6	11.574932000	10.806887000	-3.438626000
6	10.733467000	9.738357000	-3.750125000
1	10.904280000	9.144875000	-4.639944000
1	12.395949000	11.038006000	-4.106956000
6	10.254918000	11.283755000	-1.460466000
1	10.058993000	11.851970000	-0.558822000
6	9.409747000	10.213987000	-1.758714000
1	8.582122000	9.984766000	-1.098681000
6	9.662887000	9.458697000	-2.901565000
17	8.555402000	8.053419000	-3.304942000
35	7.615287000	11.462106000	1.408500000

$^{3}Re_{1Br}$:

26	5.957655000	12.631756000	3.225068000
8	4.707480000	11.248991000	3.122486000
1	5.093174000	10.484438000	2.646234000
6	3.733115000	9.000727000	6.224991000
1	4.618585000	9.454150000	5.765386000
1	3.083444000	8.631347000	5.420982000
1	4.063012000	8.137470000	6.815306000
6	1.738986000	9.333550000	7.738315000
1	2.034897000	8.476607000	8.354616000
1	1.059282000	8.969579000	6.957573000
1	1.182344000	10.031603000	8.376442000
6	3.916475000	10.504053000	8.248992000
1	4.781508000	11.035912000	7.840038000
1	4.286501000	9.654770000	8.836298000
1	3.389097000	11.181570000	8.932056000
6	2.984235000	10.012852000	7.121494000
6	2.478995000	11.210823000	6.259835000
1	1.760637000	10.819003000	5.529628000
1	1.925785000	11.907739000	6.897478000
6	4.061330000	13.134892000	5.843196000

6	3.716975000	13.877535000	7.005066000
1	2.960380000	13.510453000	7.683764000
6	4.383973000	15.061401000	7.278174000
1	4.130159000	15.625793000	8.169237000
6	5.399748000	15.520066000	6.423288000
1	5.955745000	16.423421000	6.641432000
6	5.678663000	14.768903000	5.285361000
6	6.715169000	15.249593000	4.295952000
1	6.261883000	16.032522000	3.679232000
1	7.557685000	15.705387000	4.824397000
6	7.317817000	14.769259000	1.953704000
1	7.865487000	14.050279000	1.336917000
1	7.853696000	15.723875000	1.958524000
6	5.929462000	14.898262000	1.403240000
6	5.575509000	15.889036000	0.503208000
1	6.283956000	16.662514000	0.235517000
6	4.285708000	15.839808000	-0.059235000
1	3.974222000	16.594885000	-0.772577000
6	3.417801000	14.822031000	0.285810000
1	2.436718000	14.765894000	-0.162563000
6	3.817601000	13.830997000	1.230678000
6	1.712705000	12.563876000	0.938275000
1	1.864071000	12.306382000	-0.122149000
1	1.123582000	13.489744000	0.965833000
6	0.868930000	11.449072000	1.607031000
6	-0.420946000	11.294081000	0.765522000
1	-1.074637000	10.535389000	1.210672000
1	-0.189655000	10.982058000	-0.260231000
1	-0.980012000	12.236870000	0.718836000
6	0.492978000	11.865350000	3.046372000
1	1.385709000	12.054833000	3.650524000
1	-0.092507000	11.076435000	3.533419000
1	-0.111522000	12.781044000	3.041157000
6	1.628201000	10.101059000	1.614984000
1	2.518771000	10.132379000	2.251537000
1	1.940768000	9.822479000	0.600899000
1	0.976220000	9.306539000	1.997076000
6	8.576693000	13.592054000	3.736572000
1	9.252867000	14.382704000	4.073622000
1	8.982426000	13.152024000	2.825411000
6	8.379030000	12.439651000	4.749930000
6	8.285798000	12.879937000	6.227566000
6	7.503568000	12.119638000	7.113559000
1	6.924777000	11.294300000	6.717573000
6	7.465069000	12.424558000	8.477893000
1	6.855373000	11.823814000	9.145581000
6	8.208117000	13.499279000	8.984054000
1	8.177340000	13.737108000	10.042516000
6	8.994192000	14.259717000	8.112273000
1	9.578407000	15.093051000	8.490093000
6	9.037213000	13.948777000	6.746446000
1	9.672288000	14.547628000	6.102357000
6	9.555521000	11.441791000	4.627558000
6	10.887691000	11.886110000	4.708565000
1	11.108001000	12.938275000	4.858162000
6	11.948445000	10.978624000	4.609260000
1	12.970789000	11.339053000	4.668145000
6	11.692967000	9.612034000	4.438570000
1	12.515375000	8.907618000	4.364561000
6	10.369072000	9.163171000	4.365411000
1	10.159825000	8.106030000	4.233391000
6	9.307250000	10.072010000	4.456436000
1	8.281706000	9.733197000	4.388390000
7	3.510240000	11.934505000	5.513575000
1	3.876507000	11.500551000	4.655903000
7	5.010499000	13.630571000	4.978154000
7	7.243324000	14.192510000	3.346838000
7	5.072736000	13.903569000	1.796760000
7	3.011458000	12.801585000	1.582244000
1	3.446248000	12.078353000	2.171445000
8	7.153151000	11.776473000	4.345090000
6	12.380487000	12.830534000	-1.542278000
6	13.847518000	12.857717000	-1.539399000
6	14.565321000	14.019646000	-1.925549000
6	15.960483000	14.049955000	-1.926600000

6	16.656480000	12.908570000	-1.531753000
6	16.000005000	11.742381000	-1.141710000
6	14.604613000	11.721899000	-1.150713000
1	16.560678000	10.870396000	-0.828014000
17	18.489271000	12.942632000	-1.525574000
1	16.491009000	14.941386000	-2.238025000
1	14.023057000	14.899507000	-2.251108000
1	14.092200000	10.822745000	-0.829804000
6	11.626773000	14.038568000	-1.195103000
6	12.124869000	14.972163000	-0.247995000
1	13.069695000	14.779968000	0.246136000
6	11.412009000	16.123570000	0.088785000
1	11.802666000	16.817243000	0.823177000
6	10.184256000	16.355741000	-0.528745000
17	9.241966000	17.869522000	-0.091224000
6	9.649844000	15.473619000	-1.466430000
1	8.698856000	15.679277000	-1.942195000
6	10.369954000	14.323062000	-1.791404000
1	9.964548000	13.646271000	-2.533773000
6	11.672190000	11.595079000	-1.891761000
6	12.201926000	10.687331000	-2.846832000
6	11.530674000	9.512152000	-3.186848000
1	11.942770000	8.838314000	-3.928004000
1	13.136624000	10.918233000	-3.344079000
6	10.431267000	11.259003000	-1.291343000
1	9.990294000	11.904450000	-0.540955000
6	9.753274000	10.084079000	-1.618827000
1	8.813708000	9.858900000	-1.128304000
6	10.315185000	9.229539000	-2.564329000
17	9.431604000	7.683868000	-3.006778000
35	7.159065000	11.368964000	0.857307000

^{5}TS$_{OH,1Br}$:

26	7.055181000	11.932347000	3.197104000
8	6.303596000	10.040820000	2.664753000
1	6.525716000	9.535131000	3.476613000
6	4.362048000	8.243940000	6.334727000
1	5.189621000	8.952910000	6.220191000
1	4.461175000	7.473712000	5.561056000
1	4.467360000	7.756792000	7.311626000
6	1.854035000	7.936408000	6.390053000
1	1.911917000	7.441932000	7.366735000
1	1.909150000	7.160350000	5.616705000
1	0.873049000	8.422728000	6.316029000
6	2.893330000	10.015250000	7.365038000
1	3.690444000	10.762629000	7.295280000
1	2.978940000	9.525001000	8.342926000
1	1.929135000	10.537176000	7.335484000
6	2.999791000	8.965152000	6.236904000
6	2.838763000	9.622314000	4.827521000
1	2.840971000	8.821506000	4.081932000
1	1.853174000	10.098023000	4.766766000
6	3.902357000	11.911841000	4.719796000
6	2.753067000	12.620962000	5.153968000
1	1.800834000	12.117296000	5.243093000
6	2.865683000	13.969417000	5.456239000
1	1.990400000	14.527362000	5.772079000
6	4.113457000	14.602462000	5.365500000
1	4.237663000	15.643224000	5.639870000
6	5.209520000	13.852084000	4.937898000
6	6.591492000	14.456051000	5.047254000
1	6.520918000	15.547262000	4.943990000
1	6.933083000	14.260348000	6.067148000
6	7.770876000	14.804123000	2.923192000
1	8.671706000	14.484714000	2.390221000
1	7.888985000	15.857390000	3.216437000
6	6.602808000	14.661653000	1.975447000
6	6.166603000	15.741704000	1.215814000
1	6.612975000	16.719201000	1.350231000
6	5.148034000	15.525398000	0.274701000
1	4.790747000	16.344020000	-0.340855000
6	4.583448000	14.267639000	0.138288000
1	3.783230000	14.106689000	-0.567915000
6	5.045488000	13.201879000	0.956026000
6	3.595584000	11.467264000	-0.145750000

1	3.919791000	10.460944000	-0.433319000
1	3.713705000	12.093109000	-1.036760000
6	2.083625000	11.392110000	0.233640000
6	1.316288000	10.967152000	-1.040769000
1	0.247115000	10.856666000	-0.825029000
1	1.684136000	10.006513000	-1.423095000
1	1.424669000	11.714235000	-1.837007000
6	1.548557000	12.752595000	0.730168000
1	2.106019000	13.101290000	1.606403000
1	0.493744000	12.659317000	1.016321000
1	1.612487000	13.520097000	-0.050002000
6	1.878131000	10.330006000	1.334719000
1	2.460601000	10.577187000	2.227860000
1	2.185453000	9.336896000	0.981476000
1	0.821088000	10.269497000	1.620631000
6	8.921627000	13.600728000	4.781405000
1	9.221427000	14.401907000	5.470575000
1	9.666263000	13.542712000	3.988473000
6	8.885256000	12.198141000	5.494593000
6	8.186576000	12.256187000	6.871863000
6	7.275512000	11.251645000	7.231875000
1	7.057660000	10.479677000	6.505415000
6	6.662537000	11.249702000	8.491337000
1	5.962893000	10.459890000	8.747543000
6	6.947953000	12.259772000	9.417798000
1	6.470934000	12.262121000	10.392905000
6	7.861471000	13.265060000	9.076002000
1	8.099308000	14.050351000	9.787138000
6	8.478508000	13.258488000	7.818690000
1	9.201445000	14.034455000	7.586844000
6	10.353791000	11.703064000	5.655935000
6	10.943792000	11.414040000	6.898699000
1	10.380297000	11.549198000	7.813117000
6	12.264191000	10.948089000	6.983510000
1	12.692935000	10.733007000	7.957907000
6	13.023365000	10.761259000	5.825097000
1	14.046656000	10.404161000	5.889115000
6	12.444706000	11.036698000	4.578344000
1	13.018185000	10.886964000	3.668614000
6	11.126069000	11.494493000	4.494737000
1	10.686280000	11.677381000	3.519986000
7	3.880600000	10.571803000	4.433630000
1	4.678118000	10.226726000	3.905606000
7	5.107783000	12.548160000	4.570883000
7	7.621330000	13.924637000	4.113847000
7	6.062839000	13.414942000	1.849628000
7	4.521420000	11.938573000	0.892631000
1	4.946773000	11.236713000	1.493091000
8	8.186739000	11.294201000	4.619069000
6	6.904161000	8.194813000	1.437599000
6	8.361471000	8.160486000	1.698431000
6	9.271310000	8.015898000	0.624925000
6	10.641031000	7.867362000	0.849121000
6	11.108601000	7.876850000	2.159971000
6	10.252662000	8.034165000	3.249302000
6	8.884706000	8.167654000	3.012805000
1	10.640771000	8.062944000	4.258834000
17	12.898386000	7.668270000	2.460386000
1	11.322219000	7.738000000	0.017788000
1	8.906523000	7.982551000	-0.392971000
1	8.230131000	8.309286000	3.863116000
6	6.458495000	8.631453000	0.095030000
6	6.994925000	9.800933000	-0.491372000
1	7.668046000	10.431907000	0.079851000
6	6.658422000	10.171999000	-1.793964000
1	7.069384000	11.075015000	-2.228182000
6	5.787070000	9.362371000	-2.521126000
17	5.352161000	9.838095000	-4.232656000
6	5.239948000	8.198700000	-1.986213000
1	4.578585000	7.574639000	-2.573857000
6	5.575043000	7.840243000	-0.677573000
1	5.176791000	6.916546000	-0.278160000
6	6.041296000	7.249629000	2.179955000
6	6.575982000	6.098560000	2.804778000
6	5.746207000	5.147544000	3.404726000

1	6.169760000	4.264013000	3.865163000
1	7.642410000	5.917390000	2.789884000
6	4.635756000	7.417838000	2.193186000
1	4.204569000	8.303851000	1.747188000
6	3.797333000	6.471045000	2.779787000
1	2.723074000	6.605652000	2.771177000
6	4.368973000	5.346689000	3.377076000
17	3.275592000	4.099377000	4.140120000
35	9.184650000	12.143335000	1.241349000

$^5P_{OH,1Br}$:

26	7.440763000	12.422106000	3.834212000
8	5.620734000	7.647435000	1.046026000
1	6.286727000	8.263437000	1.432842000
6	4.978634000	7.826764000	4.881953000
1	5.773708000	8.514888000	5.190932000
1	5.202824000	7.482525000	3.865553000
1	5.004548000	6.958589000	5.551477000
6	2.501641000	7.501826000	4.515676000
1	2.486266000	6.644393000	5.198516000
1	2.684628000	7.122029000	3.502621000
1	1.506573000	7.964440000	4.532105000
6	3.312628000	8.999331000	6.377219000
1	4.045174000	9.748647000	6.695476000
1	3.366046000	8.158080000	7.079309000
1	2.310532000	9.438567000	6.459712000
6	3.594841000	8.510990000	4.940199000
6	3.546860000	9.690611000	3.920934000
1	3.693870000	9.273173000	2.917770000
1	2.550328000	10.144862000	3.930887000
6	4.395814000	11.944579000	4.708659000
6	3.193279000	12.322784000	5.358240000
1	2.350647000	11.647699000	5.382218000
6	3.123978000	13.553666000	5.991353000
1	2.211092000	13.845626000	6.499402000
6	4.234215000	14.410623000	5.991518000
1	4.205694000	15.366785000	6.499789000
6	5.385025000	14.006807000	5.318449000
6	6.560680000	14.968080000	5.247535000
1	6.301408000	15.751087000	4.524270000
1	6.667010000	15.467099000	6.218432000
6	8.520208000	15.174576000	3.756071000
1	9.513085000	14.741951000	3.593500000
1	8.661351000	16.221061000	4.060642000
6	7.747835000	15.117874000	2.461513000
6	7.590771000	16.257580000	1.685157000
1	7.981356000	17.208004000	2.026212000
6	6.912560000	16.142139000	0.458866000
1	6.767758000	17.014027000	-0.169664000
6	6.422080000	14.914855000	0.056232000
1	5.888843000	14.821986000	-0.878045000
6	6.608959000	13.773377000	0.886435000
6	5.553876000	12.158409000	-0.748086000
1	5.995367000	11.197150000	-1.039087000
1	5.836183000	12.878150000	-1.523352000
6	4.004266000	11.993358000	-0.727961000
6	3.576423000	11.511204000	-2.133640000
1	2.489197000	11.378875000	-2.177943000
1	4.043012000	10.549793000	-2.382213000
1	3.860074000	12.237728000	-2.905634000
6	3.308366000	13.334566000	-0.407379000
1	3.627796000	13.724376000	0.565775000
1	2.220635000	13.198223000	-0.373633000
1	3.525962000	14.091014000	-1.171663000
6	3.606331000	10.940468000	0.329258000
1	3.872954000	11.280215000	1.335980000
1	4.109672000	9.981729000	0.150942000
1	2.523908000	10.765575000	0.305710000
6	8.816976000	14.064062000	5.942883000
1	8.736840000	14.814886000	6.737876000
1	9.821395000	14.146269000	5.523418000
6	8.691645000	12.595322000	6.474068000
6	7.604619000	12.397194000	7.557655000
6	7.016017000	11.125511000	7.684146000
1	7.296793000	10.356136000	6.975082000

6	6.087385000	10.856955000	8.694487000
1	5.652697000	9.865259000	8.775429000
6	5.718750000	11.860374000	9.601700000
1	4.996976000	11.653779000	10.385667000
6	6.294196000	13.129351000	9.487157000
1	6.020039000	13.917512000	10.181994000
6	7.231240000	13.393237000	8.477098000
1	7.664160000	14.386466000	8.426292000
6	10.069140000	12.220152000	7.088512000
6	10.557231000	12.845825000	8.250071000
1	9.950262000	13.579225000	8.770901000
6	11.819289000	12.519934000	8.761277000
1	12.179508000	13.013619000	9.658951000
6	12.611972000	11.557154000	8.122949000
1	13.588570000	11.300849000	8.521950000
6	12.130950000	10.926395000	6.969165000
1	12.735109000	10.175353000	6.468467000
6	10.870992000	11.258388000	6.454903000
1	10.485159000	10.779932000	5.563459000
7	4.559920000	10.737364000	4.094389000
1	5.464259000	10.562656000	3.666536000
7	5.466083000	12.810715000	4.673205000
7	7.862038000	14.373418000	4.830657000
7	7.262007000	13.892233000	2.093859000
7	6.171104000	12.534602000	0.528619000
1	6.457243000	11.762907000	1.124924000
8	8.429237000	11.762964000	5.346527000
6	6.271331000	6.463246000	0.455496000
6	7.319116000	6.932798000	-0.575033000
6	8.548290000	6.281067000	-0.756704000
6	9.454267000	6.711175000	-1.736909000
6	9.110599000	7.800100000	-2.529426000
6	7.897018000	8.469127000	-2.382916000
6	7.004706000	8.024828000	-1.403523000
1	7.649094000	9.312764000	-3.015127000
17	10.289890000	8.375140000	-3.812562000
1	10.402189000	6.204572000	-1.869011000
1	8.816425000	5.435066000	-0.135734000
1	6.054519000	8.529706000	-1.278127000
6	5.138670000	5.707299000	-0.260819000
6	3.799308000	5.935443000	0.091021000
1	3.569038000	6.667075000	0.853177000
6	2.761189000	5.238978000	-0.540750000
1	1.729294000	5.423066000	-0.268043000
6	3.083102000	4.308930000	-1.523872000
17	1.729188000	3.390834000	-2.358687000
6	4.399339000	4.053947000	-1.898109000
1	4.627458000	3.328550000	-2.669064000
6	5.424383000	4.762835000	-1.261140000
1	6.450089000	4.575743000	-1.557892000
6	6.900527000	5.636671000	1.597887000
6	7.884101000	6.236262000	2.409585000
6	8.458013000	5.547303000	3.481420000
1	9.211985000	6.019420000	4.099018000
1	8.206384000	7.254799000	2.220627000
6	6.502903000	4.323957000	1.892260000
1	5.747667000	3.833331000	1.292196000
6	7.070106000	3.616772000	2.962173000
1	6.755612000	2.603299000	3.178129000
6	8.038443000	4.243403000	3.736469000
17	8.788580000	3.327495000	5.138579000
35	7.659743000	10.113043000	2.422214000

$^5TS_{Br,1Br}$:

26	6.484407000	13.205792000	3.413240000
8	5.785447000	11.396864000	2.982305000
1	6.404319000	10.683046000	2.743872000
6	5.347606000	8.765631000	5.733200000
1	6.048338000	9.522717000	5.363566000
1	4.912870000	8.252718000	4.865543000
1	5.916540000	8.027635000	6.311626000
6	3.240615000	8.316500000	7.052637000
1	3.749081000	7.540495000	7.637003000
1	2.769080000	7.832915000	6.187777000
1	2.446600000	8.743859000	7.678396000

6	4.878393000	10.065663000	7.848695000
1	5.569551000	10.865408000	7.560935000
1	5.441153000	9.323948000	8.428684000
1	4.111610000	10.494416000	8.505689000
6	4.243578000	9.405296000	6.605759000
6	3.451052000	10.454831000	5.765491000
1	3.007192000	9.932629000	4.907589000
1	2.616520000	10.838127000	6.361489000
6	4.273963000	12.834632000	5.734441000
6	3.555604000	13.252726000	6.887053000
1	2.941619000	12.551075000	7.433441000
6	3.669319000	14.564550000	7.320561000
1	3.128515000	14.886148000	8.204788000
6	4.489986000	15.468641000	6.630720000
1	4.606921000	16.492417000	6.965409000
6	5.165509000	15.014371000	5.498178000
6	6.000781000	15.999947000	4.702571000
1	5.307484000	16.641666000	4.144713000
1	6.533530000	16.655140000	5.402094000
6	6.799950000	16.027685000	2.377179000
1	7.635275000	15.681535000	1.760002000
1	6.840576000	17.125152000	2.426488000
6	5.516180000	15.580067000	1.719975000
6	4.738920000	16.460288000	0.973072000
1	5.006754000	17.507725000	0.907708000
6	3.604872000	15.950250000	0.316536000
1	2.966120000	16.611374000	-0.260005000
6	3.297784000	14.602869000	0.405621000
1	2.423567000	14.205309000	-0.091124000
6	4.136991000	13.741123000	1.170806000
6	2.885175000	11.708674000	0.498715000
1	2.866155000	12.098395000	-0.529386000
1	1.892568000	11.909619000	0.933974000
6	3.087087000	10.171331000	0.417706000
6	1.951485000	9.610649000	-0.471643000
1	2.040392000	8.521819000	-0.561135000
1	1.990653000	10.037896000	-1.481339000
1	0.966616000	9.835172000	-0.043296000
6	2.996097000	9.526352000	1.820558000
1	3.808839000	9.866939000	2.469635000
1	3.062391000	8.434410000	1.738547000
1	2.039955000	9.771297000	2.300349000
6	4.453527000	9.840453000	-0.227144000
1	5.282736000	10.212950000	0.383679000
1	4.534493000	10.289828000	-1.224806000
1	4.570249000	8.755148000	-0.333681000
6	8.389033000	15.402695000	4.173579000
1	8.653062000	16.359018000	4.644108000
1	8.993197000	15.295645000	3.270930000
6	8.732064000	14.168930000	5.077070000
6	8.355011000	14.355002000	6.570157000
6	7.907420000	13.235594000	7.293110000
1	7.783770000	12.300954000	6.760394000
6	7.617089000	13.326818000	8.658468000
1	7.273712000	12.447417000	9.195442000
6	7.765441000	14.546272000	9.332517000
1	7.537964000	14.620220000	10.391430000
6	8.211246000	15.668564000	8.625680000
1	8.331073000	16.620700000	9.134068000
6	8.507406000	15.572115000	7.258879000
1	8.857050000	16.460409000	6.743482000
6	10.266947000	13.938422000	4.997084000
6	11.191700000	14.950079000	5.315434000
1	10.848141000	15.924522000	5.646357000
6	12.569482000	14.717379000	5.222122000
1	13.266518000	15.512094000	5.470560000
6	13.048367000	13.464128000	4.817303000
1	14.116563000	13.280245000	4.755037000
6	12.134549000	12.449370000	4.503569000
1	12.492790000	11.472017000	4.193324000
6	10.756533000	12.687842000	4.589189000
1	10.037166000	11.918433000	4.339622000
7	4.242400000	11.566277000	5.240774000
1	4.814852000	11.383673000	4.395816000
7	5.055286000	13.740846000	5.046749000

7	6.963774000	15.411092000	3.726232000
7	5.216941000	14.259069000	1.849295000
7	3.930239000	12.401866000	1.254656000
1	4.590878000	11.870699000	1.854377000
8	8.048092000	13.044888000	4.535190000
6	10.232111000	11.976063000	-0.847457000
6	10.358039000	10.650352000	-0.197069000
6	11.615692000	10.152163000	0.210726000
6	11.741926000	8.879359000	0.777015000
6	10.599331000	8.103112000	0.938243000
6	9.336460000	8.553546000	0.547276000
6	9.224443000	9.820489000	-0.019450000
1	8.459752000	7.934859000	0.691103000
17	10.751733000	6.441355000	1.683275000
1	12.713411000	8.506244000	1.075492000
1	12.509079000	10.744342000	0.061202000
1	8.245861000	10.195370000	-0.291769000
6	11.312239000	12.968059000	-0.634213000
6	11.830837000	13.210741000	0.660695000
1	11.375635000	12.729256000	1.517128000
6	12.881399000	14.102144000	0.864248000
1	13.254519000	14.287782000	1.863784000
6	13.427596000	14.757430000	-0.241054000
17	14.806272000	15.929634000	0.018787000
6	12.955375000	14.546969000	-1.532406000
1	13.401257000	15.056885000	-2.376980000
6	11.896253000	13.653730000	-1.723188000
1	11.547163000	13.470663000	-2.731137000
6	9.435228000	12.068695000	-2.094747000
6	9.227764000	10.934274000	-2.911013000
6	8.529945000	11.028848000	-4.119492000
1	8.388245000	10.153377000	-4.740358000
1	9.637284000	9.975048000	-2.622215000
6	8.915776000	13.309669000	-2.535760000
1	9.030547000	14.184837000	-1.908706000
6	8.214177000	13.415513000	-3.734227000
1	7.808148000	14.367340000	-4.052460000
6	8.032691000	12.267853000	-4.508873000
17	7.111396000	12.400330000	-6.081777000
35	8.420706000	13.034156000	0.848076000

$^{5}P_{Br,1Br}$:

26	6.578474000	12.892546000	3.292199000
8	5.737648000	11.082496000	3.012670000
1	6.299892000	10.290191000	2.933205000
6	5.144864000	8.811487000	5.988307000
1	5.915771000	9.470577000	5.573717000
1	4.655949000	8.293803000	5.153011000
1	5.639884000	8.056313000	6.610697000
6	3.023874000	8.649168000	7.347901000
1	3.467790000	7.872639000	7.982175000
1	2.502728000	8.151234000	6.520441000
1	2.277593000	9.187393000	7.945945000
6	4.824651000	10.290356000	8.012358000
1	5.573501000	11.011296000	7.666747000
1	5.334562000	9.542772000	8.632400000
1	4.106781000	10.821489000	8.649923000
6	4.116059000	9.609132000	6.821776000
6	3.414267000	10.670208000	5.918566000
1	2.892940000	10.134766000	5.113632000
1	2.642711000	11.185620000	6.500408000
6	4.465017000	12.944747000	5.666936000
6	3.837694000	13.529434000	6.800478000
1	3.198204000	12.936251000	7.438531000
6	4.078943000	14.862214000	7.096863000
1	3.609977000	15.311039000	7.966631000
6	4.933406000	15.626957000	6.287243000
1	5.141463000	16.666096000	6.512226000
6	5.516820000	15.012366000	5.180254000
6	6.366576000	15.840713000	4.230791000
1	5.677377000	16.401384000	3.586466000
1	6.925713000	16.586048000	4.811082000
6	7.183030000	15.527471000	1.926394000
1	8.003592000	15.055078000	1.376084000
1	7.303540000	16.616953000	1.834892000

6	5.885534000	15.096180000	1.283257000
6	5.189223000	15.946638000	0.430419000
1	5.528312000	16.963244000	0.273446000
6	4.044152000	15.447233000	-0.216541000
1	3.470708000	16.087206000	-0.879220000
6	3.644325000	14.138729000	-0.008101000
1	2.762446000	13.749456000	-0.497852000
6	4.395937000	13.306049000	0.873421000
6	2.999502000	11.320895000	0.382637000
1	3.068570000	11.551637000	-0.690910000
1	2.018642000	11.690144000	0.724379000
6	3.026605000	9.776981000	0.539412000
6	1.873697000	9.212650000	-0.325025000
1	1.838135000	8.119977000	-0.245754000
1	2.007777000	9.471417000	-1.382775000
1	0.903901000	9.607141000	0.003197000
6	2.802034000	9.368817000	2.014444000
1	3.615257000	9.727213000	2.653447000
1	2.756686000	8.276089000	2.101168000
1	1.855474000	9.776528000	2.391888000
6	4.371912000	9.205797000	0.032884000
1	5.211334000	9.568805000	0.634969000
1	4.549966000	9.491793000	-1.011381000
1	4.363461000	8.110458000	0.087631000
6	8.710450000	15.015272000	3.804641000
1	9.010507000	15.966736000	4.262938000
1	9.323668000	14.878444000	2.911613000
6	9.009787000	13.780880000	4.725871000
6	8.661134000	14.014321000	6.219290000
6	8.206275000	12.922076000	6.978593000
1	8.052137000	11.978225000	6.470375000
6	7.947461000	13.050673000	8.347236000
1	7.600400000	12.190715000	8.912605000
6	8.133736000	14.282454000	8.989285000
1	7.931961000	14.384998000	10.051054000
6	8.582731000	15.379211000	8.245910000
1	8.729194000	16.341322000	8.728006000
6	8.846285000	15.244907000	6.875469000
1	9.196230000	16.115863000	6.331570000
6	10.536463000	13.496011000	4.631093000
6	11.502548000	14.453092000	4.992480000
1	11.198564000	15.419360000	5.380775000
6	12.869657000	14.172725000	4.873552000
1	13.599130000	14.925191000	5.158179000
6	13.296236000	12.925364000	4.397688000
1	14.355918000	12.705094000	4.312981000
6	12.341457000	11.964911000	4.039665000
1	12.659033000	10.993228000	3.672429000
6	10.974435000	12.251335000	4.152095000
1	10.221805000	11.524298000	3.874459000
7	4.302800000	11.646578000	5.290061000
1	4.821895000	11.337286000	4.443694000
7	5.290260000	13.711246000	4.871366000
7	7.290631000	15.078074000	3.344993000
7	5.495587000	13.815265000	1.529959000
7	4.082276000	12.004810000	1.092857000
1	4.672543000	11.495176000	1.785774000
8	8.284603000	12.675251000	4.207121000
6	10.234031000	11.599885000	-1.237000000
6	10.593335000	10.225710000	-0.686567000
6	11.920860000	9.899773000	-0.359843000
6	12.262516000	8.615598000	0.086921000
6	11.261020000	7.660236000	0.201574000
6	9.933255000	7.940484000	-0.121218000
6	9.609511000	9.222383000	-0.566398000
1	9.167170000	7.181292000	-0.025886000
17	11.693543000	5.981871000	0.791370000
1	13.289337000	8.378194000	0.335042000
1	12.705975000	10.637359000	-0.458497000
1	8.578206000	9.448581000	-0.808940000
6	11.296890000	12.663373000	-0.995402000
6	11.681525000	13.005909000	0.317479000
1	11.178752000	12.555587000	1.164886000
6	12.697323000	13.932309000	0.556391000
1	12.980140000	14.183886000	1.571031000

6	13.335219000	14.519943000	-0.535734000
17	14.665920000	15.741636000	-0.232628000
6	12.993601000	14.204842000	-1.844678000
1	13.505161000	14.665847000	-2.680211000
6	11.971185000	13.272052000	-2.068024000
1	11.717233000	13.019958000	-3.088982000
6	9.701217000	11.571483000	-2.664059000
6	9.941580000	10.468830000	-3.502077000
6	9.513899000	10.466628000	-4.837001000
1	9.705978000	9.610445000	-5.471335000
1	10.473396000	9.603598000	-3.129190000
6	9.025726000	12.685678000	-3.202739000
1	8.823245000	13.545666000	-2.576071000
6	8.591928000	12.697945000	-4.528680000
1	8.067091000	13.556708000	-4.927816000
6	8.843563000	11.580937000	-5.324941000
17	8.272457000	11.586772000	-7.064573000
35	8.537079000	12.242088000	-0.029994000

Data for 2_{Cl}:

$^{5}Re_{2Cl}$:

26	6.504002000	12.405875000	4.223576000
17	8.011659000	11.279591000	2.554948000
17	5.192828000	10.347857000	4.433364000
6	2.817131000	10.562006000	8.138425000
1	3.863658000	10.838138000	7.965002000
1	2.584068000	9.692977000	7.510377000
1	2.720159000	10.256712000	9.187068000
6	0.404996000	11.311681000	8.094989000
1	0.274274000	11.021283000	9.143854000
1	0.125845000	10.454091000	7.470180000
1	-0.293304000	12.130999000	7.882112000
6	2.224522000	12.944281000	8.732138000
1	3.248204000	13.288466000	8.547013000
1	2.151363000	12.661445000	9.789224000
1	1.541959000	13.786099000	8.562493000
6	1.867143000	11.741975000	7.831644000
6	1.968568000	12.125978000	6.323157000
1	1.664419000	11.257124000	5.727809000
1	1.253274000	12.922713000	6.099069000
6	3.778364000	13.774085000	5.675442000
6	2.972704000	14.928526000	5.885452000
1	1.938869000	14.819585000	6.178072000
6	3.521357000	16.185562000	5.723078000
1	2.906527000	17.066761000	5.869921000
6	4.880677000	16.318011000	5.391492000
1	5.346745000	17.291435000	5.304916000
6	5.629544000	15.164487000	5.201005000
6	7.125908000	15.277628000	5.054259000
1	7.391205000	16.267636000	4.665485000
1	7.552894000	15.201965000	6.057529000
6	7.876974000	14.641852000	2.783993000
1	8.556129000	13.941687000	2.289018000
1	8.302609000	15.649965000	2.709300000
6	6.539111000	14.580085000	2.092277000
6	6.194199000	15.522864000	1.133668000
1	6.852364000	16.357752000	0.929143000
6	4.979204000	15.361393000	0.447218000
1	4.670770000	16.087917000	-0.296442000
6	4.174562000	14.270373000	0.718720000
1	3.239294000	14.138870000	0.194794000
6	4.577652000	13.320522000	1.701078000
6	2.628257000	11.879332000	1.163902000
1	2.866196000	11.969219000	0.095024000
1	1.831773000	12.605855000	1.383663000
6	2.074951000	10.451329000	1.409125000
6	0.864408000	10.278132000	0.458888000
1	0.418341000	9.286202000	0.592868000
1	1.167621000	10.374223000	-0.590803000
1	0.089894000	11.027896000	0.662513000
6	1.593620000	10.285802000	2.868897000
1	2.419789000	10.371477000	3.581603000
1	1.138941000	9.297332000	3.005117000
1	0.839817000	11.043016000	3.118108000
6	3.141228000	9.383590000	1.070532000
1	4.008420000	9.444976000	1.736176000
1	3.494063000	9.497317000	0.038402000
1	2.712812000	8.379601000	1.174129000
6	9.077419000	13.757935000	4.779127000
1	9.697688000	14.619315000	5.053300000
1	9.577172000	13.197559000	3.989801000
6	8.834613000	12.787606000	5.984557000
6	8.683427000	13.512136000	7.344251000
6	7.580315000	13.245359000	8.170891000
1	6.823802000	12.552382000	7.825649000
6	7.458077000	13.869819000	9.418998000
1	6.597699000	13.652791000	10.044566000
6	8.436998000	14.767574000	9.859490000
1	8.341527000	15.252374000	10.825744000
6	9.545386000	15.032563000	9.044322000
1	10.314632000	15.722136000	9.377485000
6	9.670133000	14.406383000	7.799414000
1	10.546429000	14.611783000	7.192626000

6	9.986552000	11.767634000	6.105046000
6	11.312250000	12.100204000	5.778743000
1	11.559180000	13.085073000	5.396079000
6	12.341266000	11.162661000	5.938168000
1	13.359130000	11.435307000	5.677556000
6	12.059135000	9.881829000	6.425496000
1	12.855974000	9.154979000	6.545887000
6	10.739462000	9.544715000	6.754089000
1	10.509010000	8.552708000	7.129869000
6	9.712162000	10.480850000	6.597403000
1	8.689959000	10.218311000	6.842208000
7	3.306430000	12.514070000	5.867400000
1	3.938275000	11.753221000	5.639704000
7	5.093050000	13.909197000	5.279854000
7	7.768401000	14.220616000	4.211783000
7	5.743392000	13.513115000	2.405867000
7	3.830608000	12.209175000	1.945019000
1	4.161176000	11.546022000	2.637191000
8	7.615469000	12.082783000	5.684336000
6	7.515002000	5.626620000	-0.928533000
6	8.662994000	5.357512000	-0.057219000
6	9.804655000	6.201149000	-0.058964000
6	10.901977000	5.947421000	0.764806000
6	10.864383000	4.839914000	1.610394000
6	9.766415000	3.982185000	1.653911000
6	8.677161000	4.242698000	0.821698000
1	9.752820000	3.134899000	2.328424000
17	12.300802000	4.501116000	2.697442000
1	11.769498000	6.595294000	0.739761000
1	9.840668000	7.050908000	-0.730144000
1	7.814652000	3.588640000	0.870309000
6	7.144605000	7.009413000	-1.247470000
6	7.314614000	8.054984000	-0.302956000
1	7.703823000	7.829351000	0.682813000
6	6.965487000	9.374061000	-0.598110000
1	7.096337000	10.150442000	0.147459000
6	6.442861000	9.658226000	-1.858300000
17	5.983549000	11.389351000	-2.261120000
6	6.256520000	8.670254000	-2.824318000
1	5.859601000	8.918312000	-3.801125000
6	6.604115000	7.354840000	-2.513890000
1	6.479101000	6.588258000	-3.269502000
6	6.738123000	4.513311000	-1.482634000
6	7.354930000	3.282332000	-1.828137000
6	6.620760000	2.220892000	-2.358772000
1	7.111281000	1.294041000	-2.629821000
1	8.424735000	3.166718000	-1.701090000
6	5.338883000	4.624292000	-1.694774000
1	4.828448000	5.540542000	-1.422760000
6	4.593375000	3.567494000	-2.218822000
1	3.524017000	3.665668000	-2.359937000
6	5.248444000	2.380883000	-2.544442000
17	4.276373000	0.989546000	-3.237286000

^{5}TS$_{Cl2,2Cl}$:

26	6.152469000	11.772070000	3.822159000
17	7.851529000	10.655463000	1.482320000
17	4.370340000	9.935232000	3.466772000
6	1.826508000	9.968900000	6.984784000
1	2.919709000	10.036509000	6.948515000
1	1.494763000	9.350922000	6.140892000
1	1.550347000	9.452605000	7.912102000
6	-0.360519000	11.232123000	6.989356000
1	-0.668404000	10.736553000	7.917596000
1	-0.734289000	10.634761000	6.148136000
1	-0.849191000	12.214179000	6.953288000
6	1.666906000	12.210902000	8.134819000
1	2.752579000	12.354323000	8.102102000
1	1.421713000	11.705864000	9.077202000
1	1.189885000	13.198693000	8.150113000
6	1.178766000	11.371274000	6.934410000
6	1.533768000	12.069651000	5.585177000
1	1.131886000	11.456089000	4.769627000
1	1.020586000	13.035450000	5.531390000
6	3.686195000	13.396068000	5.492135000

6	3.120576000	14.611275000	5.964563000
1	2.070113000	14.667027000	6.211856000
6	3.933427000	15.723454000	6.110172000
1	3.508251000	16.659061000	6.458412000
6	5.304381000	15.635760000	5.821048000
1	5.962213000	16.486428000	5.951639000
6	5.810605000	14.415865000	5.376544000
6	7.307381000	14.242926000	5.225030000
1	7.776271000	15.225064000	5.069531000
1	7.681927000	13.858661000	6.178049000
6	7.913848000	13.949764000	2.842521000
1	8.425657000	13.227304000	2.197159000
1	8.534561000	14.853726000	2.926571000
6	6.598172000	14.289168000	2.182652000
6	6.434869000	15.498816000	1.514658000
1	7.223305000	16.240969000	1.532665000
6	5.231903000	15.726023000	0.828020000
1	5.064620000	16.666434000	0.313501000
6	4.254551000	14.745913000	0.806950000
1	3.323612000	14.913416000	0.284683000
6	4.477893000	13.517897000	1.492728000
6	2.361158000	12.579718000	0.601301000
1	2.654221000	12.929315000	-0.398866000
1	1.657734000	13.322921000	1.008464000
6	1.607036000	11.234894000	0.429991000
6	0.448200000	11.500518000	-0.562698000
1	-0.138665000	10.587087000	-0.712103000
1	0.829063000	11.822926000	-1.539613000
1	-0.227467000	12.277998000	-0.184703000
6	1.013893000	10.755932000	1.775188000
1	1.796961000	10.525246000	2.504311000
1	0.421463000	9.845414000	1.622348000
1	0.352642000	11.520922000	2.201450000
6	2.541014000	10.152375000	-0.159624000
1	3.359146000	9.905487000	0.524649000
1	2.975301000	10.486492000	-1.110119000
1	1.975051000	9.232607000	-0.350849000
6	8.920666000	12.470557000	4.557392000
1	9.709815000	13.107239000	4.981348000
1	9.292692000	12.015210000	3.638998000
6	8.517033000	11.294854000	5.518850000
6	8.482427000	11.713095000	7.018383000
6	7.390284000	11.328933000	7.813463000
1	6.583202000	10.786802000	7.337052000
6	7.344604000	11.645612000	9.176925000
1	6.489445000	11.338072000	9.771929000
6	8.394157000	12.355136000	9.773179000
1	8.359634000	12.603915000	10.829360000
6	9.491993000	12.739646000	8.992424000
1	10.314421000	13.288312000	9.441892000
6	9.537296000	12.416730000	7.630693000
1	10.405731000	12.716174000	7.052819000
6	9.574327000	10.165005000	5.391972000
6	10.952225000	10.427848000	5.281739000
1	11.317661000	11.449069000	5.253888000
6	11.880001000	9.380741000	5.203408000
1	12.938840000	9.606792000	5.118459000
6	11.446205000	8.049691000	5.240324000
1	12.164981000	7.237292000	5.191735000
6	10.074587000	7.778009000	5.347427000
1	9.725723000	6.750014000	5.377766000
6	9.149523000	8.826707000	5.418022000
1	8.086765000	8.630024000	5.484269000
7	2.961464000	12.252572000	5.326627000
1	3.454840000	11.465233000	4.912137000
7	5.022592000	13.323240000	5.180398000
7	7.737267000	13.292586000	4.164274000
7	5.635753000	13.320901000	2.205124000
7	3.562825000	12.507946000	1.442557000
1	3.753984000	11.661639000	1.972670000
8	7.240706000	10.815417000	5.117094000
6	8.266675000	7.237203000	0.412264000
6	9.707866000	7.334586000	0.272930000
6	10.338058000	7.188237000	-0.992021000
6	11.721649000	7.276880000	-1.112033000

6	12.479053000	7.537082000	0.032834000
6	11.899699000	7.697648000	1.293604000
6	10.518475000	7.583137000	1.413699000
1	12.507348000	7.917932000	2.161546000
17	14.282386000	7.670120000	-0.125304000
1	12.202021000	7.139405000	-2.071903000
1	9.745184000	6.954863000	-1.867523000
1	10.057232000	7.744021000	2.380316000
6	7.391214000	7.667330000	-0.657743000
6	7.755536000	8.745000000	-1.510973000
1	8.658557000	9.304825000	-1.309742000
6	6.916660000	9.150487000	-2.543136000
1	7.184269000	9.988070000	-3.173765000
6	5.710988000	8.470835000	-2.735014000
17	4.621113000	8.991089000	-4.089748000
6	5.311503000	7.408714000	-1.920275000
1	4.376551000	6.894876000	-2.101094000
6	6.146372000	7.017020000	-0.878245000
1	5.861724000	6.173508000	-0.262310000
6	7.704874000	6.658077000	1.619646000
6	8.338986000	5.556783000	2.255814000
6	7.782961000	4.978321000	3.392716000
1	8.245871000	4.116863000	3.856234000
1	9.234361000	5.125555000	1.825224000
6	6.514197000	7.184375000	2.186317000
1	6.064560000	8.077571000	1.771123000
6	5.977922000	6.633484000	3.347102000
1	5.101229000	7.079374000	3.798304000
6	6.612109000	5.530341000	3.920730000
17	5.898464000	4.788254000	5.417672000

$^5P_{Cl2,2Cl}$:

26	5.714060000	11.122203000	3.946468000
17	9.595943000	9.241358000	0.816631000
17	3.835932000	9.403394000	3.988607000
6	1.576356000	10.666121000	7.519399000
1	2.662129000	10.552190000	7.422503000
1	1.095862000	9.948049000	6.843024000
1	1.300374000	10.399928000	8.546736000
6	-0.399340000	12.232207000	7.353318000
1	-0.702068000	11.996175000	8.380126000
1	-0.919895000	11.539473000	6.680273000
1	-0.741442000	13.250069000	7.126692000
6	1.829795000	13.093281000	8.169780000
1	2.919386000	13.054275000	8.061188000
1	1.585809000	12.837339000	9.208004000
1	1.502443000	14.125526000	7.993845000
6	1.135089000	12.112407000	7.200274000
6	1.478709000	12.456604000	5.718418000
1	0.925847000	11.765563000	5.070590000
1	1.116178000	13.462603000	5.484593000
6	3.788458000	13.369907000	5.233842000
6	3.457810000	14.729520000	5.479417000
1	2.458163000	15.002883000	5.784499000
6	4.433610000	15.700891000	5.329995000
1	4.185408000	16.742351000	5.505068000
6	5.741984000	15.340599000	4.966869000
1	6.523225000	16.084188000	4.868191000
6	6.021145000	13.994313000	4.752750000
6	7.452738000	13.551653000	4.522957000
1	8.018108000	14.369033000	4.054350000
1	7.899009000	13.381156000	5.507570000
6	7.762189000	12.543560000	2.278640000
1	8.152002000	11.623361000	1.829692000
1	8.494803000	13.340353000	2.087020000
6	6.458108000	12.877573000	1.593165000
6	6.409077000	13.885878000	0.638921000
1	7.285794000	14.488861000	0.438582000
6	5.202939000	14.097812000	-0.050118000
1	5.127377000	14.887388000	-0.790015000
6	4.108588000	13.296221000	0.216359000
1	3.176246000	13.453077000	-0.306300000
6	4.211015000	12.267267000	1.197390000
6	1.915063000	11.482531000	0.676460000
1	2.162799000	11.500160000	-0.394346000

1	1.374207000	12.415205000	0.898630000
6	0.956537000	10.288101000	0.922065000
6	-0.244720000	10.481662000	-0.036200000
1	-0.970740000	9.671918000	0.098936000
1	0.078681000	10.475166000	-1.084398000
1	-0.758131000	11.431643000	0.158057000
6	0.438389000	10.281058000	2.379150000
1	1.248039000	10.119748000	3.097542000
1	-0.292116000	9.474326000	2.514265000
1	-0.058352000	11.229763000	2.618752000
6	1.655518000	8.948144000	0.593629000
1	2.499668000	8.754522000	1.263493000
1	2.026687000	8.945092000	-0.438892000
1	0.947129000	8.118059000	0.700720000
6	8.680488000	11.392743000	4.270628000
1	9.578736000	11.969839000	4.525085000
1	8.943110000	10.706472000	3.463693000
6	8.168497000	10.511343000	5.461385000
6	8.240020000	11.226771000	6.838921000
6	7.171004000	11.085403000	7.739508000
1	6.309432000	10.511024000	7.423366000
6	7.216034000	11.672116000	9.010033000
1	6.377654000	11.547786000	9.689165000
6	8.334752000	12.415270000	9.406276000
1	8.370302000	12.873198000	10.389823000
6	9.410094000	12.559001000	8.520894000
1	10.285692000	13.129845000	8.815161000
6	9.364791000	11.966243000	7.252524000
1	10.218745000	12.086843000	6.593903000
6	9.052179000	9.237980000	5.543507000
6	10.455935000	9.289484000	5.462042000
1	10.967473000	10.237699000	5.332490000
6	11.221366000	8.119170000	5.542806000
1	12.303345000	8.179657000	5.472358000
6	10.596721000	6.877597000	5.714125000
1	11.190091000	5.970811000	5.779340000
6	9.199982000	6.817865000	5.799317000
1	8.703695000	5.860677000	5.929617000
6	8.436146000	7.987837000	5.711290000
1	7.355016000	7.954234000	5.757720000
7	2.893977000	12.348779000	5.356992000
1	3.230722000	11.419533000	5.117707000
7	5.064050000	13.027960000	4.850366000
7	7.610772000	12.294866000	3.737950000
7	5.381487000	12.090531000	1.900685000
7	3.165399000	11.433158000	1.447524000
1	3.280715000	10.726887000	2.170185000
8	6.826007000	10.140728000	5.169954000
6	8.880855000	7.604546000	-0.248519000
6	10.146089000	6.874705000	-0.663610000
6	10.347332000	6.471263000	-1.994923000
6	11.487171000	5.744036000	-2.364142000
6	12.422603000	5.428185000	-1.386898000
6	12.255869000	5.804501000	-0.054069000
6	11.113910000	6.522955000	0.300115000
1	12.997656000	5.547148000	0.691360000
17	13.919442000	4.486733000	-1.859181000
1	11.630667000	5.435974000	-3.392085000
1	9.614455000	6.705598000	-2.755541000
1	10.981228000	6.826040000	1.331540000
6	8.108147000	8.238810000	-1.391291000
6	8.719312000	9.204772000	-2.217547000
1	9.728134000	9.535121000	-2.002390000
6	8.038613000	9.764499000	-3.298789000
1	8.513699000	10.512126000	-3.921302000
6	6.733669000	9.344499000	-3.556754000
17	5.832499000	10.077176000	-4.971366000
6	6.099361000	8.388162000	-2.773832000
1	5.088049000	8.070480000	-2.993716000
6	6.794017000	7.837744000	-1.688046000
1	6.301357000	7.086065000	-1.085753000
6	8.025407000	6.853745000	0.756259000
6	8.156599000	5.462087000	0.910822000
6	7.325819000	4.752430000	1.787761000
1	7.434018000	3.680707000	1.896743000

1	8.896214000	4.914380000	0.341926000
6	7.030694000	7.521472000	1.499818000
1	6.913362000	8.594118000	1.403713000
6	6.199618000	6.829813000	2.382875000
1	5.448526000	7.362519000	2.955331000
6	6.366038000	5.451189000	2.509745000
17	5.284030000	4.530617000	3.665186000

$^{5}TS_{Cl3,2Cl}$:

26	7.135623000	12.097495000	3.269824000
17	8.804193000	11.710839000	1.517126000
17	5.907847000	9.445343000	2.352631000
6	4.498122000	8.127176000	5.987697000
1	5.408459000	8.733826000	5.919359000
1	4.377229000	7.583741000	5.042334000
1	4.644074000	7.389767000	6.786088000
6	2.003087000	8.113011000	6.376575000
1	2.107277000	7.382161000	7.186974000
1	1.842707000	7.560172000	5.442395000
1	1.106021000	8.712844000	6.576393000
6	3.467992000	9.745240000	7.627306000
1	4.327958000	10.421931000	7.581075000
1	3.649284000	9.023562000	8.433364000
1	2.582121000	10.332776000	7.898052000
6	3.262945000	9.006309000	6.286969000
6	3.028199000	10.013062000	5.117853000
1	2.818702000	9.432817000	4.210706000
1	2.130762000	10.606099000	5.322094000
6	4.306240000	12.206208000	5.211613000
6	3.381677000	12.846816000	6.071553000
1	2.500437000	12.324935000	6.415117000
6	3.638746000	14.142460000	6.495972000
1	2.941339000	14.641406000	7.160607000
6	4.807116000	14.788840000	6.081682000
1	5.048619000	15.788781000	6.422653000
6	5.669857000	14.118766000	5.209450000
6	6.913639000	14.857601000	4.762050000
1	6.590032000	15.731543000	4.184025000
1	7.415093000	15.252274000	5.653371000
6	8.156893000	14.791313000	2.656370000
1	8.965145000	14.253969000	2.152816000
1	8.481017000	15.827947000	2.826717000
6	6.938582000	14.774977000	1.760172000
6	6.637994000	15.872414000	0.958691000
1	7.241654000	16.769897000	1.014226000
6	5.540989000	15.782274000	0.087973000
1	5.276020000	16.621364000	-0.546325000
6	4.786177000	14.621833000	0.043753000
1	3.930898000	14.554407000	-0.612281000
6	5.136371000	13.530343000	0.882603000
6	3.404276000	11.957935000	-0.082989000
1	3.609993000	10.924020000	-0.388488000
1	3.490907000	12.571266000	-0.985802000
6	1.940846000	12.022133000	0.449991000
6	1.012565000	11.576897000	-0.704172000
1	-0.034332000	11.584286000	-0.379129000
1	1.254847000	10.559970000	-1.037887000
1	1.102995000	12.248693000	-1.567080000
6	1.570764000	13.455061000	0.890537000
1	2.240426000	13.811547000	1.681111000
1	0.546140000	13.478072000	1.281132000
1	1.622572000	14.158220000	0.050361000
6	1.779977000	11.057572000	1.645394000
1	2.450468000	11.333030000	2.466731000
1	2.004717000	10.024624000	1.348744000
1	0.751075000	11.082411000	2.023853000
6	9.135082000	13.685488000	4.657851000
1	9.540434000	14.527794000	5.234339000
1	9.861521000	13.429850000	3.884616000
6	8.952774000	12.392834000	5.531515000
6	8.313564000	12.651140000	6.921411000
6	7.504084000	11.647196000	7.481441000
1	7.309016000	10.762128000	6.888877000
6	6.954794000	11.789689000	8.759931000
1	6.336130000	10.997326000	9.171042000

6	7.198823000	12.948572000	9.509259000
1	6.770354000	13.063223000	10.500096000
6	8.004249000	13.955255000	8.966687000
1	8.204663000	14.858818000	9.534824000
6	8.560716000	13.804605000	7.688334000
1	9.188860000	14.601613000	7.304632000
6	10.372266000	11.799271000	5.762935000
6	11.390214000	12.531646000	6.401845000
1	11.194472000	13.530153000	6.778308000
6	12.666455000	11.982829000	6.576197000
1	13.438921000	12.565583000	7.069378000
6	12.946128000	10.687839000	6.120971000
1	13.934932000	10.261101000	6.258658000
6	11.938142000	9.950515000	5.488207000
1	12.142863000	8.945945000	5.129421000
6	10.664070000	10.504466000	5.308585000
1	9.877272000	9.951749000	4.811627000
7	4.143140000	10.909366000	4.803113000
1	4.864331000	10.526542000	4.208308000
7	5.422131000	12.868794000	4.748285000
7	7.884476000	14.087910000	3.941947000
7	6.201300000	13.626287000	1.740748000
7	4.452440000	12.344419000	0.866966000
1	4.810151000	11.608120000	1.459972000
8	8.158978000	11.471162000	4.787242000
6	6.451090000	7.622485000	1.232541000
6	7.845142000	7.363420000	1.728434000
6	8.904557000	7.160605000	0.823315000
6	10.189031000	6.839190000	1.278239000
6	10.405259000	6.731202000	2.646743000
6	9.383402000	6.929981000	3.576847000
6	8.107011000	7.240648000	3.110849000
1	9.577164000	6.848688000	4.638767000
17	12.078130000	6.311530000	3.249950000
1	10.994226000	6.676582000	0.573295000
1	8.734135000	7.227477000	-0.242768000
1	7.311942000	7.412823000	3.825834000
6	6.326555000	8.091617000	-0.190005000
6	7.023151000	9.238161000	-0.632653000
1	7.595678000	9.835743000	0.070608000
6	6.956418000	9.643794000	-1.965473000
1	7.483343000	10.530679000	-2.294425000
6	6.195778000	8.890833000	-2.860883000
17	6.104899000	9.421308000	-4.608385000
6	5.505674000	7.750429000	-2.466494000
1	4.929452000	7.174034000	-3.178944000
6	5.571428000	7.356184000	-1.124796000
1	5.048738000	6.458085000	-0.823378000
6	5.390426000	6.667774000	1.708673000
6	5.732244000	5.403398000	2.225989000
6	4.742774000	4.490652000	2.611905000
1	5.017769000	3.518671000	3.001004000
1	6.769485000	5.108817000	2.312186000
6	4.021886000	6.994015000	1.582095000
1	3.735712000	7.965654000	1.199135000
6	3.027454000	6.098013000	1.970780000
1	1.981739000	6.362625000	1.879625000
6	3.408243000	4.856088000	2.482027000
17	2.110016000	3.676211000	2.991650000

$^5P_{Cl3,2Cl}$:

26	6.430756000	12.509515000	3.158835000
17	5.093845000	11.227955000	1.379382000
17	5.706669000	4.244092000	0.766263000
6	2.426362000	9.109404000	3.795531000
1	3.497095000	9.301143000	3.927221000
1	2.202170000	9.153277000	2.722319000
1	2.219272000	8.090259000	4.143330000
6	0.073341000	9.829025000	4.366642000
1	-0.171968000	8.824314000	4.729895000
1	-0.190788000	9.877309000	3.302748000
1	-0.557017000	10.546431000	4.907417000
6	1.907791000	10.030042000	6.088342000
1	2.957148000	10.275933000	6.281657000
1	1.730110000	9.008704000	6.446989000

1	1.278793000	10.705601000	6.681369000
6	1.575335000	10.129781000	4.584049000
6	1.835589000	11.570051000	4.043348000
1	1.511359000	11.600127000	2.996047000
1	1.205693000	12.283435000	4.585214000
6	3.810897000	12.831394000	5.004420000
6	3.124711000	13.298308000	6.155036000
1	2.093714000	13.024406000	6.323851000
6	3.800358000	14.083095000	7.075580000
1	3.284690000	14.434563000	7.962918000
6	5.148897000	14.408514000	6.869603000
1	5.699101000	15.005175000	7.587020000
6	5.773182000	13.950052000	5.711802000
6	7.204060000	14.384204000	5.439227000
1	7.169242000	15.425233000	5.094659000
1	7.757588000	14.390782000	6.386427000
6	8.639067000	14.468683000	3.430141000
1	9.246691000	13.816011000	2.794605000
1	9.319026000	15.175257000	3.926406000
6	7.666463000	15.221270000	2.556509000
6	7.899719000	16.548986000	2.225202000
1	8.753971000	17.072181000	2.636043000
6	7.001548000	17.186689000	1.351200000
1	7.151949000	18.224748000	1.075231000
6	5.919150000	16.491335000	0.847769000
1	5.219405000	16.979996000	0.186297000
6	5.720006000	15.130704000	1.217863000
6	3.717970000	14.796292000	-0.287009000
1	3.588365000	13.940130000	-0.961010000
1	4.137477000	15.607538000	-0.890692000
6	2.311123000	15.212446000	0.239934000
6	1.446046000	15.563971000	-0.992817000
1	0.435840000	15.854756000	-0.682016000
1	1.356930000	14.705778000	-1.670540000
1	1.880175000	16.399940000	-1.555623000
6	2.409132000	16.439533000	1.172268000
1	3.053558000	16.232269000	2.034107000
1	1.415705000	16.707462000	1.551987000
1	2.810188000	17.312486000	0.642523000
6	1.670782000	14.032173000	1.003322000
1	2.273633000	13.754240000	1.874979000
1	1.575584000	13.150261000	0.357020000
1	0.668294000	14.302834000	1.356073000
6	8.858082000	12.536753000	4.954397000
1	9.293862000	12.854014000	5.909194000
1	9.680735000	12.446537000	4.242707000
6	8.199340000	11.114539000	5.030351000
6	7.408490000	10.840290000	6.330233000
6	6.380828000	9.879423000	6.287610000
1	6.142281000	9.424299000	5.333963000
6	5.676092000	9.523429000	7.441702000
1	4.892206000	8.774529000	7.383939000
6	5.979347000	10.128120000	8.670100000
1	5.432231000	9.854229000	9.566666000
6	6.996204000	11.085491000	8.727361000
1	7.243530000	11.562823000	9.670812000
6	7.707019000	11.434319000	7.568838000
1	8.497639000	12.171731000	7.654812000
6	9.367304000	10.093748000	4.927706000
6	10.232987000	9.839427000	6.006593000
1	10.056456000	10.309916000	6.968039000
6	11.319305000	8.966633000	5.862614000
1	11.975284000	8.781999000	6.708102000
6	11.556868000	8.331021000	4.637687000
1	12.397287000	7.652830000	4.526916000
6	10.698034000	8.576356000	3.558524000
1	10.870047000	8.088095000	2.603725000
6	9.613530000	9.451510000	3.702983000
1	8.939999000	9.647031000	2.877965000
7	3.228284000	12.028867000	4.067821000
1	3.806370000	11.750609000	3.278749000
7	5.124160000	13.189334000	4.787695000
7	7.941249000	13.591420000	4.416941000
7	6.595780000	14.512053000	2.084216000
7	4.686560000	14.387879000	0.735989000

1	4.687467000	13.399750000	0.980032000
8	7.344695000	10.983227000	3.894938000
6	7.561220000	4.716839000	1.545582000
6	7.927695000	6.011759000	0.840810000
6	9.162704000	6.158333000	0.185463000
6	9.519254000	7.371692000	-0.418999000
6	8.626270000	8.434658000	-0.358448000
6	7.393839000	8.333737000	0.286700000
6	7.054296000	7.118111000	0.882990000
1	6.715595000	9.178091000	0.336866000
17	9.084819000	10.022173000	-1.150216000
1	10.472839000	7.473208000	-0.921716000
1	9.864819000	5.336215000	0.146958000
1	6.094050000	7.027221000	1.376192000
6	8.434176000	3.528114000	1.173657000
6	8.462670000	3.039993000	-0.148337000
1	7.808635000	3.472609000	-0.895139000
6	9.307189000	1.990319000	-0.512211000
1	9.314374000	1.616909000	-1.528415000
6	10.136787000	1.432111000	0.459775000
17	11.246460000	0.053423000	-0.009419000
6	10.146854000	1.890919000	1.771070000
1	10.802373000	1.448527000	2.510454000
6	9.289223000	2.941839000	2.123143000
1	9.305672000	3.304222000	3.142575000
6	7.301625000	4.855599000	3.036842000
6	7.750830000	5.979031000	3.751544000
6	7.556417000	6.079934000	5.136413000
1	7.903797000	6.953985000	5.672805000
1	8.268983000	6.783599000	3.247571000
6	6.659953000	3.819564000	3.746423000
1	6.301272000	2.946137000	3.215681000
6	6.456576000	3.906972000	5.123694000
1	5.954225000	3.110267000	5.657687000
6	6.908566000	5.043100000	5.795310000
17	6.638002000	5.166356000	7.602377000

Data for 1_F:

$^5Re_{1F}$:

26	6.777498000	12.474344000	4.161191000
8	5.579254000	11.027270000	3.651209000
1	5.942244000	10.129721000	3.536298000
6	3.562938000	8.937285000	6.198259000
1	4.545614000	9.400831000	6.054672000
1	3.257872000	8.483299000	5.246566000
1	3.671452000	8.133248000	6.936110000
6	1.140188000	9.290865000	6.820368000
1	1.188739000	8.475710000	7.551872000
1	0.806560000	8.867278000	5.864648000
1	0.379423000	10.004921000	7.160689000
6	2.953839000	10.557761000	8.038198000
1	3.914172000	11.078401000	7.955815000
1	3.065796000	9.756038000	8.778280000
1	2.210207000	11.267594000	8.421182000
6	2.520164000	9.973626000	6.676254000
6	2.372980000	11.103788000	5.609981000
1	2.030115000	10.641394000	4.675222000
1	1.583420000	11.794683000	5.922164000
6	3.932175000	13.079244000	5.764331000
6	3.104087000	13.842388000	6.631642000
1	2.153681000	13.448026000	6.961090000
6	3.533108000	15.084121000	7.069172000
1	2.905522000	15.666931000	7.735324000
6	4.781537000	15.577381000	6.665424000
1	5.148634000	16.535272000	7.013786000
6	5.553573000	14.799740000	5.804196000
6	6.878170000	15.362514000	5.330114000
1	6.663028000	16.168612000	4.618741000
1	7.390441000	15.827248000	6.179820000
6	8.237878000	14.906297000	3.326055000
1	8.982883000	14.201583000	2.949286000
1	8.693044000	15.901031000	3.408761000
6	7.071117000	14.923673000	2.368918000
6	6.866582000	15.959013000	1.464705000
1	7.523457000	16.819610000	1.455606000
6	5.779695000	15.854225000	0.576109000
1	5.572437000	16.655978000	-0.124718000
6	4.968257000	14.732795000	0.594551000
1	4.126991000	14.654357000	-0.079635000
6	5.243128000	13.682774000	1.519239000
6	3.506137000	12.181408000	0.565421000
1	3.803045000	12.567774000	-0.418394000
1	2.555903000	12.670764000	0.831808000
6	3.255626000	10.656025000	0.414293000
6	2.202869000	10.480373000	-0.706715000
1	1.978042000	9.417708000	-0.853160000
1	2.566863000	10.884083000	-1.659509000
1	1.266077000	10.992114000	-0.453358000
6	2.699908000	10.051728000	1.724789000
1	3.427052000	10.117927000	2.540321000
1	2.456812000	8.992483000	1.577498000
1	1.782961000	10.569389000	2.033406000
6	4.560842000	9.932644000	0.009901000
1	5.326623000	10.021326000	0.787880000
1	4.968893000	10.349791000	-0.919272000
1	4.368709000	8.865571000	-0.153745000
6	8.948165000	13.927438000	5.496150000
1	9.424703000	14.772784000	6.006048000
1	9.668539000	13.501885000	4.799035000
6	8.540091000	12.788293000	6.494490000
6	7.953902000	13.320502000	7.815580000
6	6.865532000	12.655110000	8.403920000
1	6.439361000	11.807777000	7.882010000
6	6.336074000	13.083115000	9.626473000
1	5.493270000	12.554557000	10.061650000
6	6.886026000	14.189675000	10.286079000
1	6.473973000	14.525070000	11.232633000
6	7.975686000	14.856904000	9.714763000
1	8.416809000	15.711722000	10.218208000
6	8.507470000	14.422947000	8.493018000
1	9.366810000	14.945771000	8.085558000

6	9.787833000	11.897272000	6.734795000
6	10.459075000	11.808369000	7.963210000
1	10.116783000	12.385259000	8.813879000
6	11.575937000	10.971482000	8.108736000
1	12.081374000	10.916040000	9.068209000
6	12.033779000	10.210468000	7.029294000
1	12.897293000	9.562366000	7.143002000
6	11.366629000	10.291204000	5.797963000
1	11.712582000	9.702339000	4.953509000
6	10.254214000	11.124059000	5.650730000
1	9.731606000	11.187040000	4.700325000
7	3.597882000	11.842646000	5.308610000
1	4.283800000	11.392089000	4.681395000
7	5.144875000	13.590788000	5.340017000
7	7.794697000	14.393676000	4.661300000
7	6.265221000	13.828018000	2.427121000
7	4.549027000	12.516758000	1.539238000
1	4.831204000	11.830960000	2.255889000
8	7.554223000	11.978365000	5.820540000
6	11.854923000	11.287870000	-0.613434000
6	13.164535000	10.871626000	-1.129314000
6	13.805119000	11.583704000	-2.176584000
6	15.049164000	11.191316000	-2.672703000
6	15.672142000	10.075815000	-2.115518000
6	15.087718000	9.343333000	-1.083576000
6	13.840652000	9.741657000	-0.599936000
1	15.595032000	8.486638000	-0.657169000
17	17.308463000	9.557080000	-2.758943000
1	15.515988000	11.739013000	-3.482127000
1	13.311037000	12.439475000	-2.621274000
1	13.393311000	9.185125000	0.215014000
6	11.519239000	12.713117000	-0.545083000
6	12.513951000	13.692689000	-0.285404000
1	13.537703000	13.382593000	-0.113032000
6	12.202120000	15.050900000	-0.213702000
1	12.972808000	15.781391000	-0.000497000
6	10.879749000	15.446336000	-0.407684000
17	10.464058000	17.230660000	-0.316766000
6	9.864984000	14.526710000	-0.668541000
1	8.845167000	14.856180000	-0.823691000
6	10.188591000	13.170594000	-0.733780000
1	9.404611000	12.456931000	-0.956914000
6	10.890559000	10.279754000	-0.163431000
6	10.826616000	8.997869000	-0.772791000
6	9.914599000	8.031420000	-0.346672000
1	9.871640000	7.064074000	-0.832109000
1	11.482588000	8.766327000	-1.603643000
6	9.985448000	10.542298000	0.897220000
1	9.988113000	11.493382000	1.413352000
6	9.072178000	9.582077000	1.335688000
1	8.413558000	9.830354000	2.157356000
6	9.053148000	8.340868000	0.705579000
17	7.856143000	7.069857000	1.274353000
9	8.329128000	11.941629000	3.126194000

$^{5}TS_{OH,1F}$;

26	6.912249000	11.893126000	3.137491000
8	6.370903000	9.886243000	2.990935000
1	6.943304000	9.618919000	3.747603000
6	4.582785000	8.184937000	6.194453000
1	5.417886000	8.871937000	6.015216000
1	4.580215000	7.431308000	5.397846000
1	4.767489000	7.671213000	7.145551000
6	2.084507000	7.944259000	6.477432000
1	2.221865000	7.413071000	7.426567000
1	2.042308000	7.196777000	5.675627000
1	1.115855000	8.458694000	6.516076000
6	3.272363000	9.965134000	7.408263000
1	4.070539000	10.702718000	7.272685000
1	3.452399000	9.446227000	8.358118000
1	2.320265000	10.502428000	7.495380000
6	3.239899000	8.947270000	6.247019000
6	2.969107000	9.650549000	4.877628000
1	2.898193000	8.871325000	4.113111000
1	1.990733000	10.141245000	4.913151000

6	4.002341000	11.940473000	4.701223000
6	2.898829000	12.628425000	5.270377000
1	1.977721000	12.104655000	5.481717000
6	3.017551000	13.978646000	5.560450000
1	2.175205000	14.513852000	5.986228000
6	4.229318000	14.643651000	5.324403000
1	4.360148000	15.686334000	5.588636000
6	5.280827000	13.924408000	4.757710000
6	6.641041000	14.590105000	4.649941000
1	6.512135000	15.622858000	4.301898000
1	7.044722000	14.653093000	5.666183000
6	7.898361000	14.596971000	2.510549000
1	8.634739000	13.989000000	1.978912000
1	8.307422000	15.601003000	2.691118000
6	6.656507000	14.691279000	1.664196000
6	6.360109000	15.866528000	0.976489000
1	7.004866000	16.731253000	1.073871000
6	5.214037000	15.897397000	0.169887000
1	4.956633000	16.794085000	-0.383696000
6	4.397307000	14.780887000	0.093315000
1	3.503209000	14.800150000	-0.512811000
6	4.742941000	13.614844000	0.829543000
6	2.667346000	12.484511000	0.070395000
1	2.853653000	12.732878000	-0.983963000
1	2.003246000	13.264308000	0.474227000
6	1.917143000	11.129943000	0.115072000
6	0.596359000	11.321152000	-0.669127000
1	0.024488000	10.386393000	-0.682156000
1	0.792210000	11.612600000	-1.708103000
1	-0.029098000	12.094791000	-0.207421000
6	1.582612000	10.735029000	1.571085000
1	2.480853000	10.586320000	2.180359000
1	1.011028000	9.799431000	1.587173000
1	0.976406000	11.510852000	2.054888000
6	2.755714000	10.029198000	-0.570694000
1	3.727904000	9.890363000	-0.087494000
1	2.943452000	10.282753000	-1.620890000
1	2.225300000	9.070098000	-0.543208000
6	8.917494000	13.555086000	4.490621000
1	9.276446000	14.414056000	5.072729000
1	9.639679000	13.362792000	3.699438000
6	8.827947000	12.252249000	5.368316000
6	8.280240000	12.545986000	6.782900000
6	7.308243000	11.697336000	7.335943000
1	6.953210000	10.874000000	6.729692000
6	6.805210000	11.916318000	8.624343000
1	6.054064000	11.244850000	9.029924000
6	7.264802000	12.996346000	9.388077000
1	6.873735000	13.170215000	10.385762000
6	8.237869000	13.849103000	8.852302000
1	8.608090000	14.687478000	9.434763000
6	8.742666000	13.623001000	7.564865000
1	9.511032000	14.287453000	7.181704000
6	10.263874000	11.656755000	5.419684000
6	11.027642000	11.529922000	6.589709000
1	10.625457000	11.858606000	7.540445000
6	12.318862000	10.980481000	6.549009000
1	12.892090000	10.892005000	7.467318000
6	12.865133000	10.550924000	5.336776000
1	13.865036000	10.129366000	5.303797000
6	12.105158000	10.663160000	4.162530000
1	12.515471000	10.321766000	3.216957000
6	10.817219000	11.202430000	4.203309000
1	10.219275000	11.276803000	3.298885000
7	3.988903000	10.601324000	4.427845000
1	4.812952000	10.226683000	3.939939000
7	5.159794000	12.619246000	4.399298000
7	7.635063000	13.886237000	3.797423000
7	5.878079000	13.574273000	1.594597000
7	3.942628000	12.506176000	0.798541000
1	4.210696000	11.721156000	1.373116000
8	7.979401000	11.313537000	4.695181000
6	6.848498000	8.157442000	1.804402000
6	8.223505000	7.920313000	2.310331000
6	9.339579000	7.923544000	1.442702000

6	10.618691000	7.596780000	1.900091000
6	10.789552000	7.271522000	3.242886000
6	9.726446000	7.283073000	4.143826000
6	8.453032000	7.604939000	3.673144000
1	9.881670000	7.041301000	5.187775000
17	12.452327000	6.791394000	3.836673000
1	11.457369000	7.580350000	1.215274000
1	9.207400000	8.148514000	0.392586000
1	7.625738000	7.592824000	4.374021000
6	6.692789000	8.697916000	0.423749000
6	7.301319000	9.911467000	0.033097000
1	7.834176000	10.524445000	0.758567000
6	7.203036000	10.365797000	-1.286900000
1	7.662122000	11.304051000	-1.574839000
6	6.509172000	9.599002000	-2.218834000
17	6.386883000	10.194199000	-3.951323000
6	5.906226000	8.391590000	-1.878730000
1	5.382645000	7.801772000	-2.620764000
6	5.997427000	7.950505000	-0.555283000
1	5.543094000	7.002705000	-0.294814000
6	5.776098000	7.231498000	2.264937000
6	6.060891000	5.935960000	2.750673000
6	5.037854000	5.040663000	3.082501000
1	5.275166000	4.047477000	3.442746000
1	7.086001000	5.601569000	2.842498000
6	4.418530000	7.594370000	2.119206000
1	4.174567000	8.583837000	1.757373000
6	3.387057000	6.710797000	2.439984000
1	2.351620000	7.004008000	2.318156000
6	3.716510000	5.443964000	2.920917000
17	2.369665000	4.276159000	3.340706000
9	8.449327000	11.854281000	1.877570000

$^{5}P_{OH,1F}$:

26	6.244822000	11.837980000	2.898775000
8	7.063791000	8.341031000	3.223503000
1	7.571169000	9.216764000	3.158649000
6	2.970658000	8.174359000	5.128322000
1	4.011103000	8.504946000	5.035404000
1	2.629556000	7.836931000	4.141901000
1	2.948436000	7.311844000	5.805195000
6	0.608423000	8.807802000	5.757535000
1	0.538929000	7.949924000	6.436568000
1	0.234502000	8.491388000	4.775618000
1	-0.055964000	9.594003000	6.138163000
6	2.558102000	9.737459000	7.064716000
1	3.581087000	10.127937000	7.026615000
1	2.552244000	8.878784000	7.747257000
1	1.909551000	10.510261000	7.495128000
6	2.069231000	9.307550000	5.664401000
6	2.084568000	10.508085000	4.667657000
1	1.703752000	10.151981000	3.702786000
1	1.384368000	11.275944000	5.014864000
6	3.888544000	12.232811000	5.005600000
6	3.171257000	12.995487000	5.967162000
1	2.188612000	12.683477000	6.289306000
6	3.742173000	14.146638000	6.484647000
1	3.192023000	14.739417000	7.207836000
6	5.028256000	14.544106000	6.081794000
1	5.496125000	15.433367000	6.486237000
6	5.702546000	13.749978000	5.159249000
6	7.148452000	14.044897000	4.817859000
1	7.337471000	15.122947000	4.914315000
1	7.768022000	13.546239000	5.568399000
6	7.405082000	14.588142000	2.413642000
1	8.073604000	14.324957000	1.586601000
1	7.710046000	15.580716000	2.773297000
6	6.002447000	14.658864000	1.850878000
6	5.452686000	15.881563000	1.482526000
1	5.980728000	16.803981000	1.690370000
6	4.206662000	15.882986000	0.829958000
1	3.745447000	16.821096000	0.539740000
6	3.564585000	14.687715000	0.557936000
1	2.602566000	14.680024000	0.064940000
6	4.169612000	13.461221000	0.956993000

6	2.378880000	12.080810000	-0.081628000
1	2.413581000	12.747079000	-0.954087000
1	1.497829000	12.372457000	0.512942000
6	2.172617000	10.630520000	-0.597231000
6	0.875522000	10.629660000	-1.440973000
1	0.670842000	9.622695000	-1.822081000
1	0.961960000	11.306328000	-2.300100000
1	0.012955000	10.946070000	-0.841379000
6	2.011289000	9.642122000	0.582239000
1	2.916831000	9.589304000	1.195121000
1	1.803614000	8.634274000	0.202443000
1	1.172004000	9.938016000	1.224789000
6	3.361933000	10.200460000	-1.486393000
1	4.297746000	10.173558000	-0.918255000
1	3.492104000	10.892744000	-2.327524000
1	3.187258000	9.198316000	-1.895533000
6	8.973976000	12.987468000	3.477743000
1	9.656241000	13.599177000	4.081776000
1	9.326195000	13.029744000	2.446599000
6	9.025811000	11.473388000	3.897336000
6	8.985208000	11.262732000	5.427710000
6	8.235786000	10.201853000	5.965719000
1	7.681522000	9.555530000	5.297427000
6	8.204847000	9.970149000	7.346723000
1	7.619790000	9.142761000	7.736930000
6	8.920966000	10.795043000	8.222012000
1	8.893355000	10.616830000	9.292292000
6	9.677845000	11.850678000	7.700688000
1	10.243495000	12.497054000	8.364692000
6	9.711751000	12.078814000	6.318822000
1	10.317742000	12.897912000	5.944801000
6	10.336750000	10.908270000	3.280823000
6	11.513318000	10.688707000	4.014440000
1	11.530826000	10.865737000	5.082720000
6	12.679190000	10.230278000	3.382857000
1	13.576689000	10.066937000	3.971765000
6	12.689135000	9.985251000	2.006038000
1	13.592367000	9.633374000	1.517560000
6	11.518904000	10.198502000	1.263093000
1	11.509272000	10.006134000	0.194610000
6	10.357588000	10.652570000	1.895694000
1	9.447944000	10.792461000	1.324256000
7	3.395954000	11.108021000	4.414206000
1	3.948673000	10.715888000	3.644345000
7	5.147266000	12.631462000	4.617662000
7	7.589615000	13.558918000	3.478068000
7	5.375189000	13.470222000	1.623627000
7	3.607957000	12.253076000	0.695550000
1	4.053095000	11.439345000	1.137160000
8	7.894458000	10.835871000	3.274969000
6	7.600039000	7.241011000	2.441303000
6	8.766019000	6.641731000	3.254897000
6	10.085982000	7.095835000	3.094443000
6	11.117463000	6.645461000	3.928767000
6	10.810784000	5.740217000	4.938122000
6	9.513235000	5.283098000	5.149446000
6	8.497537000	5.744763000	4.305594000
1	9.291990000	4.588407000	5.950363000
17	12.156708000	5.136393000	6.034295000
1	12.128705000	7.006649000	3.790749000
1	10.327544000	7.817387000	2.324570000
1	7.484804000	5.399992000	4.475807000
6	7.981740000	7.713881000	1.018636000
6	7.299545000	8.810133000	0.461381000
1	6.548546000	9.332477000	1.044884000
6	7.556799000	9.221169000	-0.852435000
1	7.029913000	10.067264000	-1.277187000
6	8.493538000	8.517595000	-1.604034000
17	8.838823000	9.055186000	-3.328270000
6	9.169063000	7.413038000	-1.095592000
1	9.884863000	6.871106000	-1.701356000
6	8.900888000	7.013255000	0.219973000
1	9.423044000	6.148771000	0.613197000
6	6.432108000	6.248970000	2.244199000
6	6.656919000	4.907620000	1.892800000

6	5.588078000	4.040797000	1.636006000
1	5.767693000	3.006001000	1.371588000
1	7.667805000	4.522011000	1.820854000
6	5.112236000	6.719409000	2.320039000
1	4.944914000	7.760612000	2.565514000
6	4.031990000	5.866070000	2.061230000
1	3.014057000	6.232445000	2.116362000
6	4.292310000	4.540575000	1.727917000
17	2.879644000	3.411600000	1.392118000
9	4.937601000	10.460365000	2.195776000

$^5TS_{F,1F}$:

26	7.183179000	12.576202000	3.533222000
8	6.237020000	10.868840000	3.106815000
1	6.718078000	10.023597000	3.066068000
6	5.414619000	8.421813000	5.975806000
1	6.199524000	9.132670000	5.694248000
1	5.082979000	7.900397000	5.068500000
1	5.855021000	7.678449000	6.651228000
6	3.135812000	8.112683000	7.018478000
1	3.530540000	7.351216000	7.701645000
1	2.766519000	7.600948000	6.120491000
1	2.282027000	8.596665000	7.509726000
6	4.725244000	9.841254000	7.947853000
1	5.468267000	10.611836000	7.716309000
1	5.190285000	9.113025000	8.623764000
1	3.894544000	10.315708000	8.485127000
6	4.232311000	9.143293000	6.661996000
6	3.602891000	10.177275000	5.676727000
1	3.228300000	9.622794000	4.806541000
1	2.729586000	10.639201000	6.149489000
6	4.580032000	12.498767000	5.610674000
6	3.750179000	13.033452000	6.632999000
1	3.019792000	12.407983000	7.126385000
6	3.900174000	14.359964000	7.006484000
1	3.272022000	14.773183000	7.789159000
6	4.870858000	15.158793000	6.386613000
1	5.021988000	16.191286000	6.678592000
6	5.657093000	14.586587000	5.385440000
6	6.665902000	15.462549000	4.671026000
1	6.113345000	16.093932000	3.964513000
1	7.124911000	16.141184000	5.400500000
6	7.859596000	15.302950000	2.527857000
1	8.713226000	14.800138000	2.068525000
1	8.048344000	16.385622000	2.542432000
6	6.626728000	14.994337000	1.709788000
6	6.083459000	15.926902000	0.829863000
1	6.508390000	16.920116000	0.751585000
6	4.968284000	15.543415000	0.063716000
1	4.506760000	16.250898000	-0.617460000
6	4.451024000	14.263519000	0.179790000
1	3.588794000	13.965920000	-0.400660000
6	5.060685000	13.340893000	1.080213000
6	3.592507000	11.487243000	0.350242000
1	3.775350000	11.777105000	-0.695189000
1	2.612678000	11.906298000	0.630498000
6	3.496483000	9.939652000	0.402015000
6	2.378339000	9.522550000	-0.583375000
1	2.257310000	8.433157000	-0.581982000
1	2.615850000	9.835929000	-1.607522000
1	1.416491000	9.969876000	-0.303005000
6	3.125784000	9.457686000	1.823246000
1	3.915232000	9.698652000	2.541360000
1	2.983191000	8.370105000	1.828092000
1	2.192299000	9.923579000	2.163197000
6	4.831994000	9.297564000	-0.038976000
1	5.643775000	9.557506000	0.648173000
1	5.114552000	9.630525000	-1.044987000
1	4.740273000	8.204731000	-0.056542000
6	9.042228000	14.649252000	4.606586000
1	9.295633000	15.605186000	5.083513000
1	9.787192000	14.454751000	3.834934000
6	9.106771000	13.443412000	5.606370000
6	8.517319000	13.754311000	7.007727000
6	7.844832000	12.731049000	7.696898000

1	7.699947000	11.785737000	7.189325000
6	7.364121000	12.930086000	8.995438000
1	6.847817000	12.123305000	9.507469000
6	7.542992000	14.164534000	9.633673000
1	7.168726000	14.322248000	10.640450000
6	8.211771000	15.192361000	8.959409000
1	8.358823000	16.154605000	9.440965000
6	8.699010000	14.986796000	7.661378000
1	9.223499000	15.801382000	7.173109000
6	10.601819000	13.068549000	5.808733000
6	11.572023000	14.027818000	6.155513000
1	11.290916000	15.065793000	6.299466000
6	12.912316000	13.660863000	6.331762000
1	13.645658000	14.416495000	6.597294000
6	13.305119000	12.325247000	6.173152000
1	14.343099000	12.040039000	6.313530000
6	12.345901000	11.363651000	5.833566000
1	12.637994000	10.325366000	5.706970000
6	11.007226000	11.733976000	5.649535000
1	10.257441000	11.003137000	5.374520000
7	4.512669000	11.208167000	5.183359000
1	5.147852000	10.961589000	4.399826000
7	5.515224000	13.299903000	4.985990000
7	7.727424000	14.748299000	3.907278000
7	6.120528000	13.740921000	1.858187000
7	4.647397000	12.051670000	1.196520000
1	5.170566000	11.456905000	1.864271000
8	8.417050000	12.351772000	5.007015000
6	10.043277000	11.678384000	0.778675000
6	10.374957000	10.442311000	1.520038000
6	11.709401000	10.090091000	1.818237000
6	12.011890000	8.898941000	2.485173000
6	10.967666000	8.058251000	2.859525000
6	9.634953000	8.365485000	2.584607000
6	9.347975000	9.554543000	1.914501000
1	8.839441000	7.697326000	2.890639000
17	11.354250000	6.497931000	3.736053000
1	13.040195000	8.634440000	2.697266000
1	12.522917000	10.733815000	1.508193000
1	8.319377000	9.822698000	1.712774000
6	11.009729000	12.797920000	0.811957000
6	11.588155000	13.198184000	2.039042000
1	11.287293000	12.705383000	2.954371000
6	12.519140000	14.235403000	2.096426000
1	12.945467000	14.536445000	3.045250000
6	12.877529000	14.878437000	0.911540000
17	14.099504000	16.241146000	0.977527000
6	12.337353000	14.515770000	-0.318914000
1	12.638788000	15.021460000	-1.227694000
6	11.401819000	13.477046000	-0.362239000
1	10.995515000	13.182340000	-1.321656000
6	9.132392000	11.571317000	-0.385154000
6	9.016624000	10.369958000	-1.117707000
6	8.196321000	10.287333000	-2.247498000
1	8.126121000	9.364515000	-2.809538000
1	9.587203000	9.497988000	-0.822876000
6	8.388507000	12.692415000	-0.818458000
1	8.439430000	13.613392000	-0.254490000
6	7.561524000	12.621577000	-1.939638000
1	6.983511000	13.482110000	-2.252159000
6	7.481701000	11.416338000	-2.636950000
17	6.400153000	11.317246000	-4.112940000
9	8.718064000	12.485864000	2.081551000

$^5P_{F,1F}$:

26	7.176066000	12.467457000	3.590569000
8	6.406433000	10.647948000	3.176454000
1	6.943966000	9.842129000	3.280727000
6	5.342196000	8.487174000	6.133968000
1	6.159353000	9.143301000	5.813719000
1	5.000050000	7.918258000	5.259836000
1	5.743596000	7.775173000	6.865223000
6	3.029230000	8.348670000	7.139835000
1	3.371446000	7.609598000	7.873836000
1	2.651662000	7.805179000	6.264395000

1	2.192162000	8.902529000	7.583851000
6	4.681798000	10.051417000	8.004690000
1	5.475280000	10.761913000	7.749213000
1	5.084722000	9.341911000	8.737912000
1	3.866866000	10.605711000	8.486849000
6	4.182544000	9.302394000	6.750398000
6	3.624180000	10.305240000	5.693266000
1	3.257667000	9.722480000	4.837723000
1	2.755161000	10.823706000	6.112072000
6	4.677178000	12.588822000	5.540722000
6	3.851675000	13.207323000	6.517687000
1	3.099730000	12.635013000	7.042035000
6	4.038272000	14.550403000	6.809334000
1	3.416425000	15.026379000	7.560719000
6	5.030888000	15.290980000	6.148674000
1	5.197032000	16.337332000	6.375324000
6	5.808619000	14.642375000	5.190128000
6	6.835227000	15.438827000	4.400840000
1	6.287334000	16.015941000	3.644855000
1	7.305978000	16.170684000	5.069174000
6	8.012502000	15.051475000	2.266301000
1	8.900356000	14.547746000	1.872248000
1	8.165566000	16.136087000	2.167386000
6	6.828454000	14.617774000	1.434412000
6	6.283484000	15.454917000	0.465522000
1	6.655183000	16.464721000	0.341965000
6	5.241539000	14.953010000	-0.336033000
1	4.782004000	15.584092000	-1.089775000
6	4.797725000	13.653273000	-0.163190000
1	3.993843000	13.262174000	-0.771250000
6	5.400088000	12.831862000	0.836260000
6	4.064238000	10.852264000	0.181708000
1	4.279391000	11.072190000	-0.874301000
1	3.049604000	11.231511000	0.386351000
6	4.055694000	9.309569000	0.352611000
6	3.020648000	8.747185000	-0.651216000
1	2.968226000	7.655198000	-0.571719000
1	3.292258000	8.998748000	-1.683975000
1	2.019075000	9.148924000	-0.453218000
6	3.635314000	8.915972000	1.788166000
1	4.363293000	9.268167000	2.525815000
1	3.563292000	7.824668000	1.874796000
1	2.653690000	9.339875000	2.035687000
6	5.449854000	8.722347000	0.031655000
1	6.205940000	9.083408000	0.736646000
1	5.767187000	8.996787000	-0.982290000
1	5.423973000	7.627614000	0.093578000
6	9.208422000	14.586701000	4.377771000
1	9.450534000	15.555802000	4.833526000
1	9.950825000	14.396958000	3.600026000
6	9.334348000	13.395224000	5.389103000
6	8.747131000	13.700355000	6.792336000
6	8.164576000	12.647336000	7.519577000
1	8.094305000	11.677106000	7.043744000
6	7.677223000	12.845727000	8.815615000
1	7.232622000	12.015151000	9.356110000
6	7.759975000	14.109124000	9.416349000
1	7.380549000	14.266023000	10.421324000
6	8.339951000	15.165996000	8.706665000
1	8.411649000	16.151208000	9.158015000
6	8.831290000	14.961978000	7.409476000
1	9.278184000	15.803974000	6.891581000
6	10.851403000	13.096714000	5.557449000
6	11.761918000	14.071692000	6.005570000
1	11.413984000	15.062197000	6.279417000
6	13.127061000	13.780510000	6.117569000
1	13.813540000	14.547622000	6.463509000
6	13.606357000	12.504696000	5.791254000
1	14.663762000	12.276914000	5.884278000
6	12.706281000	11.525625000	5.351707000
1	13.064727000	10.531256000	5.101475000
6	11.341833000	11.822100000	5.232461000
1	10.632355000	11.080259000	4.888144000
7	4.587189000	11.278353000	5.180067000
1	5.242091000	10.953265000	4.442944000

7	5.636846000	13.331834000	4.888088000
7	7.880355000	14.643335000	3.695318000
7	6.389722000	13.346691000	1.644900000
7	5.047060000	11.535563000	1.025887000
1	5.529870000	11.031196000	1.799572000
8	8.686790000	12.273289000	4.805658000
6	10.699750000	11.709481000	0.200931000
6	10.821713000	10.273253000	0.704347000
6	12.004744000	9.540513000	0.524250000
6	12.093555000	8.207518000	0.945428000
6	10.985693000	7.627882000	1.553990000
6	9.797853000	8.328043000	1.752977000
6	9.719155000	9.655810000	1.320498000
1	8.950715000	7.852730000	2.231713000
17	11.092068000	5.886228000	2.115025000
1	13.008017000	7.645485000	0.803385000
1	12.867016000	10.000642000	0.056479000
1	8.799835000	10.207747000	1.477635000
6	12.019992000	12.474021000	0.232391000
6	12.596000000	12.765527000	1.482704000
1	12.100229000	12.455092000	2.395146000
6	13.807121000	13.456275000	1.567669000
1	14.244622000	13.680426000	2.532504000
6	14.436718000	13.844158000	0.385856000
17	16.028417000	14.747758000	0.486989000
6	13.899162000	13.567465000	-0.866207000
1	14.407631000	13.877992000	-1.770239000
6	12.679205000	12.880286000	-0.935936000
1	12.253603000	12.665297000	-1.908451000
6	9.943605000	11.821543000	-1.121159000
6	9.959640000	10.784919000	-2.066616000
6	9.282646000	10.916723000	-3.286017000
1	9.299222000	10.114670000	-4.013111000
1	10.496375000	9.866176000	-1.863224000
6	9.233511000	12.999555000	-1.411447000
1	9.208432000	13.808202000	-0.692523000
6	8.544202000	13.140962000	-2.619407000
1	7.989443000	14.045641000	-2.833827000
6	8.584739000	12.093360000	-3.536671000
17	7.686561000	12.271460000	-5.124489000
9	9.840854000	12.405616000	1.201495000

Data for the reaction of diphenylmethyl with 1_{Cl} and 1_{Br}:

$^{5}Re_{1Cl,PP}$:

26	6.076384000	12.471705000	3.096475000
8	4.650683000	11.160922000	2.938457000
1	4.892416000	10.304477000	2.537447000
6	3.715617000	8.804860000	5.879306000
1	4.602687000	9.248284000	5.413218000
1	3.026906000	8.501108000	5.080482000
1	4.030594000	7.901712000	6.415500000
6	1.784180000	9.133822000	7.471680000
1	2.065599000	8.234664000	8.032055000
1	1.066422000	8.837274000	6.696418000
1	1.275875000	9.817562000	8.163262000
6	4.021114000	10.196900000	7.966602000
1	4.894480000	10.713744000	7.554946000
1	4.374406000	9.306245000	8.500513000
1	3.544127000	10.860079000	8.699001000
6	3.034551000	9.797597000	6.848718000
6	2.549471000	11.054947000	6.063140000
1	1.816221000	10.723682000	5.317124000
1	2.016925000	11.725997000	6.744396000
6	4.174498000	12.957357000	5.733973000
6	3.842055000	13.632974000	6.940441000
1	3.093605000	13.221716000	7.601916000
6	4.502252000	14.801850000	7.275348000
1	4.254574000	15.314111000	8.198967000
6	5.501847000	15.312774000	6.433160000
1	6.049066000	16.211330000	6.689695000
6	5.786797000	14.628745000	5.255485000
6	6.806824000	15.216200000	4.303243000
1	6.306960000	16.004131000	3.728575000
1	7.600621000	15.704567000	4.880113000
6	7.467213000	14.889329000	1.960286000
1	8.051638000	14.225630000	1.316779000
1	7.967686000	15.865546000	1.992073000
6	6.082902000	15.018231000	1.377468000
6	5.757320000	16.064299000	0.523326000
1	6.474339000	16.850497000	0.323583000
6	4.487457000	16.056693000	-0.079894000
1	4.202073000	16.853701000	-0.758074000
6	3.601106000	15.027369000	0.182145000
1	2.632415000	15.005874000	-0.295752000
6	3.973702000	13.985944000	1.081047000
6	1.841138000	12.785585000	0.712915000
1	1.979506000	12.594596000	-0.363589000
1	1.279059000	13.724787000	0.803391000
6	0.965272000	11.653743000	1.308617000
6	-0.345805000	11.618659000	0.486157000
1	-1.017538000	10.845798000	0.876849000
1	-0.145540000	11.391820000	-0.568200000
1	-0.870719000	12.580606000	0.536528000
6	0.631380000	11.961901000	2.785491000
1	1.539349000	12.053677000	3.389710000
1	0.013307000	11.162915000	3.212425000
1	0.072785000	12.902717000	2.869187000
6	1.668475000	10.280956000	1.190058000
1	2.572508000	10.230953000	1.805006000
1	1.947015000	10.072814000	0.149427000
1	0.993196000	9.484196000	1.524428000
6	8.735522000	13.686085000	3.713652000
1	9.379113000	14.463982000	4.138754000
1	9.201537000	13.330676000	2.793809000
6	8.584533000	12.445178000	4.650497000
6	8.459213000	12.782389000	6.154990000
6	7.691871000	11.939390000	6.977381000
1	7.149696000	11.120636000	6.520583000
6	7.620695000	12.154348000	8.357595000
1	7.021853000	11.491063000	8.974112000
6	8.317033000	13.219125000	8.944302000
1	8.261925000	13.387054000	10.015139000
6	9.087868000	14.061464000	8.136266000
1	9.635705000	14.888790000	8.576711000
6	9.161558000	13.842467000	6.754085000
1	9.779505000	14.507029000	6.160238000

6	9.818192000	11.527154000	4.469603000
6	11.123929000	12.035841000	4.591587000
1	11.287448000	13.084680000	4.817177000
6	12.233776000	11.196796000	4.434473000
1	13.234376000	11.606776000	4.530825000
6	12.054632000	9.834299000	4.160850000
1	12.914736000	9.182928000	4.042719000
6	10.757380000	9.321163000	4.044727000
1	10.606498000	8.266926000	3.833410000
6	9.647303000	10.161669000	4.196572000
1	8.641951000	9.772232000	4.098563000
7	3.593529000	11.791052000	5.347399000
1	3.915075000	11.401453000	4.446496000
7	5.131672000	13.493007000	4.892821000
7	7.411480000	14.265471000	3.318208000
7	5.213095000	14.013108000	1.680019000
7	3.147384000	12.945863000	1.364106000
1	3.545187000	12.182854000	1.928473000
8	7.406511000	11.748529000	4.211353000
6	12.175156000	12.977528000	-0.370745000
6	13.610946000	12.871122000	-0.574463000
6	14.305320000	13.638620000	-1.554917000
6	15.675506000	13.486966000	-1.761707000
6	16.415069000	12.564512000	-1.005555000
6	15.754198000	11.784699000	-0.045298000
6	14.382429000	11.926051000	0.162662000
1	16.312141000	11.063774000	0.544729000
1	16.168805000	14.081090000	-2.525105000
1	13.751299000	14.331237000	-2.177277000
1	13.901507000	11.314836000	0.917321000
6	11.409889000	14.166411000	-0.743908000
6	11.972451000	15.472194000	-0.681412000
1	12.993995000	15.592693000	-0.339737000
6	11.224256000	16.604466000	-1.005732000
1	11.682495000	17.586175000	-0.932878000
6	9.886628000	16.483271000	-1.409437000
6	9.305950000	15.208242000	-1.473530000
1	8.272263000	15.097808000	-1.786710000
6	10.047011000	14.072828000	-1.140802000
1	9.574207000	13.100145000	-1.214259000
17	7.237647000	11.562093000	0.910711000
1	17.481820000	12.449977000	-1.167984000
1	9.307652000	17.364579000	-1.665869000
6	11.465536000	11.825310000	0.303561000
1	11.734619000	10.859688000	-0.142869000
1	11.736916000	11.765215000	1.369243000
1	10.379719000	11.926114000	0.268925000

^{5}TS$_{Cl,1Cl,PP}$:

26	6.930347000	13.242786000	3.673199000
8	5.583819000	12.027623000	2.822644000
1	5.916077000	11.322342000	2.237888000
6	4.285528000	8.918569000	4.637865000
1	5.210489000	9.480132000	4.465375000
1	3.721443000	8.895937000	3.696469000
1	4.554661000	7.886965000	4.895569000
6	2.127967000	8.769327000	5.941358000
1	2.335539000	7.723145000	6.195620000
1	1.535815000	8.780755000	5.017470000
1	1.513823000	9.196448000	6.744424000
6	4.245163000	9.529302000	7.089192000
1	5.168783000	10.112162000	7.003182000
1	4.517034000	8.498815000	7.349196000
1	3.656120000	9.941196000	7.918088000
6	3.446750000	9.557896000	5.768059000
6	3.071329000	11.022496000	5.381022000
1	2.482741000	10.984309000	4.454536000
1	2.413123000	11.440300000	6.150065000
6	4.655510000	12.880034000	5.995571000
6	4.105259000	13.118434000	7.284398000
1	3.282467000	12.518974000	7.647477000
6	4.650439000	14.111203000	8.083888000
1	4.237892000	14.292536000	9.071288000
6	5.740015000	14.865939000	7.626944000
1	6.194760000	15.631167000	8.244721000

6	6.237749000	14.600785000	6.350329000
6	7.370446000	15.461628000	5.822429000
1	6.941061000	16.432575000	5.545884000
1	8.075363000	15.656481000	6.640275000
6	8.206320000	15.974230000	3.569339000
1	8.875555000	15.580592000	2.798004000
1	8.635190000	16.910271000	3.955614000
6	6.860108000	16.237371000	2.936045000
6	6.466810000	17.522445000	2.571100000
1	7.098343000	18.373671000	2.794296000
6	5.233198000	17.676120000	1.914709000
1	4.886245000	18.664902000	1.632455000
6	4.453976000	16.566870000	1.628778000
1	3.500580000	16.679439000	1.131104000
6	4.913502000	15.271985000	2.008547000
6	3.024178000	14.127420000	0.885410000
1	3.165904000	14.805546000	0.031297000
1	2.163799000	14.511658000	1.457615000
6	2.654739000	12.729001000	0.321152000
6	1.410333000	12.917727000	-0.579311000
1	1.094971000	11.955873000	-1.000097000
1	1.623353000	13.598289000	-1.413147000
1	0.568293000	13.329319000	-0.008741000
6	2.308711000	11.745616000	1.463619000
1	3.176595000	11.558312000	2.103691000
1	1.979467000	10.785232000	1.047866000
1	1.495225000	12.140984000	2.085270000
6	3.818475000	12.163678000	-0.525972000
1	4.713576000	12.002053000	0.083489000
1	4.081026000	12.849718000	-1.340803000
1	3.532106000	11.202703000	-0.970515000
6	9.424753000	14.307964000	4.941364000
1	9.987166000	14.927862000	5.652459000
1	9.979234000	14.284483000	4.001715000
6	9.291047000	12.819499000	5.414753000
6	8.997920000	12.673092000	6.933475000
6	8.124996000	11.657307000	7.357613000
1	7.638532000	11.057234000	6.599123000
6	7.882060000	11.435292000	8.717594000
1	7.203535000	10.643051000	9.020583000
6	8.506103000	12.232386000	9.686042000
1	8.316720000	12.064523000	10.741810000
6	9.379537000	13.247136000	9.277556000
1	9.872218000	13.872178000	10.016584000
6	9.627083000	13.460696000	7.914227000
1	10.320284000	14.246166000	7.632621000
6	10.639952000	12.098450000	5.137448000
6	11.880111000	12.657265000	5.497502000
1	11.927790000	13.628133000	5.979917000
6	13.076068000	11.973282000	5.244824000
1	14.022595000	12.424277000	5.527731000
6	13.053329000	10.712518000	4.634526000
1	13.979515000	10.178006000	4.446808000
6	11.822682000	10.146831000	4.275042000
1	11.791671000	9.168658000	3.803759000
6	10.628851000	10.836872000	4.521153000
1	9.671239000	10.418988000	4.236872000
7	4.201783000	11.917906000	5.146316000
1	4.685238000	11.844456000	4.228822000
7	5.707856000	13.650519000	5.543067000
7	8.105151000	14.935552000	4.635413000
7	6.098829000	15.137851000	2.693519000
7	4.235322000	14.134132000	1.707694000
1	4.654216000	13.245250000	2.046805000
8	8.257425000	12.213702000	4.650256000
6	9.444734000	11.706519000	-0.757866000
6	10.876786000	11.994980000	-0.618589000
6	11.637644000	12.458184000	-1.723375000
6	13.017565000	12.643731000	-1.616589000
6	13.673215000	12.383799000	-0.407475000
6	12.938012000	11.924071000	0.696770000
6	11.564283000	11.723197000	0.592304000
1	13.434738000	11.729699000	1.641277000
1	13.580015000	12.982504000	-2.480070000
1	11.152525000	12.626269000	-2.676587000

1	11.006634000	11.411380000	1.466721000
6	8.643827000	12.369392000	-1.783699000
6	8.882063000	13.721925000	-2.144496000
1	9.640865000	14.284428000	-1.614774000
6	8.113195000	14.348512000	-3.122019000
1	8.301798000	15.387317000	-3.370405000
6	7.088538000	13.644687000	-3.773460000
6	6.832379000	12.310837000	-3.427524000
1	6.043437000	11.761079000	-3.929322000
6	7.589484000	11.682643000	-2.438089000
1	7.381526000	10.648212000	-2.193613000
17	8.524211000	13.200063000	1.359306000
1	14.744406000	12.534442000	-0.324258000
1	6.492300000	14.133544000	-4.536606000
6	8.932200000	10.427045000	-0.152205000
1	9.150481000	9.595258000	-0.841281000
1	9.433443000	10.196982000	0.787366000
1	7.856996000	10.455795000	0.023311000

$^{5}P_{Cl,1Cl,PP}$:

26	6.193170000	13.077634000	3.221835000
8	4.609903000	12.152467000	2.412669000
1	4.648894000	11.251661000	2.038407000
6	2.810486000	9.460682000	4.605166000
1	3.839602000	9.761012000	4.379623000
1	2.221632000	9.548603000	3.683345000
1	2.821014000	8.404500000	4.900463000
6	0.748474000	9.902938000	5.994868000
1	0.706335000	8.852050000	6.304532000
1	0.134772000	10.013921000	5.091936000
1	0.295718000	10.507887000	6.790882000
6	3.041317000	10.160177000	7.020575000
1	4.075386000	10.490170000	6.873347000
1	3.062802000	9.105010000	7.320573000
1	2.611598000	10.734843000	7.850675000
6	2.210503000	10.332591000	5.730795000
6	2.182937000	11.826273000	5.280449000
1	1.546173000	11.894936000	4.388732000
1	1.698225000	12.429030000	6.056383000
6	4.218145000	13.240899000	5.715879000
6	3.855793000	13.614732000	7.038108000
1	2.956649000	13.221674000	7.490403000
6	4.680331000	14.470396000	7.752155000
1	4.412194000	14.752040000	8.765353000
6	5.860704000	14.962913000	7.174997000
1	6.520919000	15.625557000	7.721840000
6	6.167850000	14.578052000	5.870479000
6	7.387610000	15.179234000	5.193744000
1	7.124501000	16.205822000	4.907588000
1	8.196959000	15.262444000	5.930120000
6	8.071265000	15.428089000	2.839395000
1	8.590524000	14.880106000	2.046266000
1	8.711151000	16.274650000	3.129009000
6	6.765075000	15.940736000	2.279482000
6	6.614641000	17.275944000	1.918834000
1	7.413350000	17.985363000	2.097726000
6	5.403733000	17.669869000	1.320878000
1	5.247580000	18.705942000	1.038602000
6	4.407002000	16.737140000	1.093072000
1	3.471574000	17.033844000	0.639443000
6	4.614649000	15.377772000	1.474740000
6	2.438800000	14.667376000	0.537318000
1	2.650635000	15.242963000	-0.376043000
1	1.769714000	15.287467000	1.156263000
6	1.673190000	13.383338000	0.121350000
6	0.417427000	13.836812000	-0.661168000
1	-0.170770000	12.966004000	-0.973046000
1	0.692779000	14.399224000	-1.562018000
1	-0.225698000	14.475237000	-0.042435000
6	1.231680000	12.578571000	1.366300000
1	2.096155000	12.216184000	1.931560000
1	0.635085000	11.709508000	1.063000000
1	0.614520000	13.196966000	2.030706000
6	2.555047000	12.501370000	-0.792366000
1	3.446756000	12.147824000	-0.265247000

1	2.878382000	13.059154000	-1.680342000
1	1.992129000	11.623000000	-1.130455000
6	9.068359000	13.599537000	4.175775000
1	9.730141000	14.011054000	4.948768000
1	9.627903000	13.608061000	3.238262000
6	8.705609000	12.093708000	4.435553000
6	8.360438000	11.770353000	5.908183000
6	7.473607000	10.709169000	6.167919000
1	7.022852000	10.200394000	5.324837000
6	7.169314000	10.323475000	7.477164000
1	6.484463000	9.498515000	7.649818000
6	7.745369000	10.995569000	8.564357000
1	7.509335000	10.698833000	9.581561000
6	8.629282000	12.051695000	8.323047000
1	9.084487000	12.582624000	9.153854000
6	8.935253000	12.432484000	7.007876000
1	9.633501000	13.249002000	6.858427000
6	9.955521000	11.272521000	4.004271000
6	11.059103000	11.062352000	4.849323000
1	11.040232000	11.427964000	5.869989000
6	12.187309000	10.364529000	4.396642000
1	13.026984000	10.209689000	5.067889000
6	12.232002000	9.864240000	3.090113000
1	13.104311000	9.320061000	2.741169000
6	11.138147000	10.070779000	2.239096000
1	11.158508000	9.686808000	1.223136000
6	10.013654000	10.769946000	2.692749000
1	9.159678000	10.928825000	2.046866000
7	3.478728000	12.408771000	4.932342000
1	3.834732000	12.236717000	3.971847000
7	5.375078000	13.741731000	5.154872000
7	7.875159000	14.477614000	3.973879000
7	5.790491000	15.009237000	2.092203000
7	3.694893000	14.407629000	1.245003000
1	3.927294000	13.453075000	1.595237000
8	7.603911000	11.777771000	3.584930000
6	6.541489000	8.349449000	0.555372000
6	7.177668000	9.325281000	-0.422652000
6	8.082419000	8.857554000	-1.394933000
6	8.766775000	9.753655000	-2.226553000
6	8.555223000	11.130008000	-2.106600000
6	7.659018000	11.606803000	-1.139922000
6	6.983303000	10.716041000	-0.302373000
1	7.483240000	12.673034000	-1.038803000
1	9.460736000	9.369782000	-2.967227000
1	8.264359000	7.795117000	-1.498627000
1	6.286383000	11.103961000	0.430816000
6	6.409667000	6.926824000	0.060584000
6	5.811585000	6.663417000	-1.189825000
1	5.429862000	7.488257000	-1.781308000
6	5.689122000	5.357067000	-1.663972000
1	5.220596000	5.175543000	-2.625639000
6	6.166631000	4.282308000	-0.899342000
6	6.762660000	4.529631000	0.340482000
1	7.137021000	3.706394000	0.939992000
6	6.879866000	5.840941000	0.819863000
1	7.347969000	6.005379000	1.782164000
1	9.078914000	11.823717000	-2.756141000
1	6.072437000	3.266105000	-1.267921000
6	7.091808000	8.516423000	1.968801000
1	8.144284000	8.205665000	1.975171000
1	7.064082000	9.560523000	2.287052000
1	6.540390000	7.909843000	2.690518000
17	4.578739000	8.953195000	0.771309000

$^{5}TS_{OH,1Cl,PP}$:

26	8.417022000	13.106293000	3.740167000
8	7.451692000	12.237171000	2.130455000
1	7.339302000	11.296161000	2.382759000
6	4.258514000	9.585653000	3.545256000
1	5.184164000	9.899170000	4.041215000
1	4.480281000	9.400412000	2.488020000
1	3.939267000	8.637685000	3.995406000
6	1.866235000	10.185094000	2.993988000
1	1.499200000	9.252554000	3.438651000

1	2.047640000	10.002332000	1.927509000
1	1.070446000	10.936004000	3.080662000
6	2.862379000	10.863987000	5.208693000
1	3.753831000	11.200831000	5.748564000
1	2.532982000	9.920343000	5.661491000
1	2.067735000	11.604209000	5.362691000
6	3.156559000	10.655502000	3.706387000
6	3.596174000	11.988880000	3.017516000
1	3.697994000	11.793147000	1.944445000
1	2.791373000	12.726575000	3.124658000
6	5.057078000	13.404069000	4.518748000
6	3.993385000	14.083558000	5.167687000
1	2.976492000	13.962285000	4.821406000
6	4.276965000	14.909215000	6.244950000
1	3.474481000	15.448668000	6.737398000
6	5.597060000	15.034376000	6.702670000
1	5.837608000	15.640580000	7.567937000
6	6.600075000	14.332926000	6.032095000
6	7.986547000	14.277663000	6.634625000
1	8.174547000	15.196609000	7.206821000
1	7.980256000	13.455409000	7.356167000
6	9.749709000	15.304518000	5.257132000
1	10.701661000	15.044189000	4.785219000
1	9.956179000	15.955022000	6.119538000
6	8.924297000	16.055218000	4.235804000
6	8.967094000	17.444782000	4.177655000
1	9.520507000	18.006070000	4.920396000
6	8.291285000	18.086893000	3.128435000
1	8.312499000	19.168485000	3.046747000
6	7.585106000	17.342666000	2.197396000
1	7.049668000	17.837695000	1.401783000
6	7.545825000	15.927216000	2.317410000
6	6.285727000	15.579181000	0.169907000
1	6.493023000	14.793874000	-0.565875000
1	6.828677000	16.468449000	-0.169644000
6	4.752999000	15.870736000	0.138038000
6	4.415965000	16.362825000	-1.289579000
1	3.340937000	16.555604000	-1.385363000
1	4.692984000	15.613505000	-2.041911000
1	4.947961000	17.293482000	-1.524313000
6	4.351683000	16.952901000	1.163882000
1	4.631959000	16.659110000	2.181463000
1	3.265530000	17.105524000	1.143766000
1	4.824512000	17.916199000	0.938589000
6	3.970941000	14.574356000	0.434926000
1	4.235336000	14.180199000	1.420891000
1	4.189140000	13.804495000	-0.316408000
1	2.890555000	14.762474000	0.417196000
6	10.081161000	13.012497000	6.174397000
1	10.345902000	13.206157000	7.222983000
1	10.976157000	13.120729000	5.561302000
6	9.546486000	11.547918000	5.964681000
6	8.619565000	11.085593000	7.124615000
6	7.343016000	10.578290000	6.838288000
1	7.028442000	10.552921000	5.803187000
6	6.501094000	10.129608000	7.864321000
1	5.516233000	9.741639000	7.620332000
6	6.923140000	10.180944000	9.198161000
1	6.271223000	9.835743000	9.994685000
6	8.198689000	10.679300000	9.495845000
1	8.540816000	10.719802000	10.525711000
6	9.039846000	11.121796000	8.468192000
1	10.030488000	11.487125000	8.721274000
6	10.741809000	10.562063000	5.912969000
6	11.928803000	10.760805000	6.640244000
1	12.060277000	11.647505000	7.251569000
6	12.969115000	9.822573000	6.585851000
1	13.879813000	9.998805000	7.150544000
6	12.837874000	8.667080000	5.807992000
1	13.643245000	7.940358000	5.766274000
6	11.657804000	8.459656000	5.080567000
1	11.545652000	7.568370000	4.470037000
6	10.623092000	9.399183000	5.132670000
1	9.715073000	9.255157000	4.559718000
7	4.868169000	12.555571000	3.462632000

1	5.712771000	12.325986000	2.935549000
7	6.353601000	13.571898000	4.935932000
7	9.104610000	14.033891000	5.683179000
7	8.230238000	15.307804000	3.331672000
7	6.855195000	15.128268000	1.446739000
1	6.923067000	14.122236000	1.594582000
8	8.841061000	11.505282000	4.726611000
6	8.164010000	11.179882000	0.087571000
6	8.329565000	12.320551000	-0.823894000
6	7.682856000	12.349457000	-2.087930000
6	7.933161000	13.376423000	-3.000142000
6	8.826893000	14.405610000	-2.677249000
6	9.474402000	14.393589000	-1.433161000
6	9.238156000	13.365771000	-0.520555000
1	10.164892000	15.189458000	-1.173377000
1	7.441249000	13.364529000	-3.967153000
1	7.023136000	11.540266000	-2.374287000
1	9.728146000	13.386437000	0.446304000
6	6.992731000	10.305296000	-0.030797000
6	5.707950000	10.829217000	-0.325097000
1	5.580680000	11.901820000	-0.397952000
6	4.608993000	9.987362000	-0.490832000
1	3.636113000	10.411217000	-0.716261000
6	4.757652000	8.598042000	-0.360871000
6	6.013172000	8.062107000	-0.045057000
1	6.135525000	6.989378000	0.059716000
6	7.114882000	8.901800000	0.129123000
1	8.080150000	8.463965000	0.351749000
17	10.807502000	13.390931000	2.732081000
1	9.021001000	15.202957000	-3.387194000
1	3.903550000	7.943253000	-0.497129000
6	9.404017000	10.639214000	0.746266000
1	9.992559000	10.076839000	0.004113000
1	10.034427000	11.442731000	1.127520000
1	9.176262000	9.965610000	1.574564000

$^{5}P_{OH,1Cl,PP}$:

26	7.445839000	12.370768000	3.820895000
8	5.605120000	7.793711000	1.087528000
1	6.213492000	8.486390000	1.435319000
6	4.881148000	7.895672000	4.843982000
1	5.660671000	8.576874000	5.203925000
1	5.154200000	7.567747000	3.833958000
1	4.870874000	7.017810000	5.501264000
6	2.418387000	7.576857000	4.381582000
1	2.366939000	6.727648000	5.072958000
1	2.644257000	7.184387000	3.382257000
1	1.426384000	8.045316000	4.348214000
6	3.160454000	9.081315000	6.265655000
1	3.884038000	9.827497000	6.610486000
1	3.179946000	8.243134000	6.973355000
1	2.158408000	9.526826000	6.305020000
6	3.497825000	8.584422000	4.843495000
6	3.494938000	9.759170000	3.817442000
1	3.659093000	9.334070000	2.820433000
1	2.507124000	10.232183000	3.802206000
6	4.375606000	11.996826000	4.620320000
6	3.165831000	12.414606000	5.231194000
1	2.301070000	11.767489000	5.227151000
6	3.117396000	13.648315000	5.861011000
1	2.198682000	13.971159000	6.339077000
6	4.254767000	14.468880000	5.897051000
1	4.241256000	15.425739000	6.404680000
6	5.412702000	14.026444000	5.261401000
6	6.625423000	14.943343000	5.230784000
1	6.417956000	15.739247000	4.504846000
1	6.721453000	15.433131000	6.207567000
6	8.635520000	15.079506000	3.798101000
1	9.616371000	14.610583000	3.666692000
1	8.804970000	16.119824000	4.109344000
6	7.905451000	15.051584000	2.478152000
6	7.816783000	16.194651000	1.695939000
1	8.228071000	17.131567000	2.049862000
6	7.180766000	16.099888000	0.445138000
1	7.091203000	16.974801000	-0.189547000

6	6.662327000	14.890145000	0.025134000
1	6.163132000	14.813683000	-0.929321000
6	6.775535000	13.744798000	0.863630000
6	5.706368000	12.163545000	-0.794672000
1	6.107561000	11.179712000	-1.067212000
1	6.043389000	12.863288000	-1.566661000
6	4.150128000	12.073469000	-0.814734000
6	3.737678000	11.603757000	-2.229299000
1	2.646869000	11.523406000	-2.302885000
1	4.164878000	10.619961000	-2.459300000
1	4.076877000	12.311080000	-2.996809000
6	3.509060000	13.447286000	-0.519841000
1	3.817579000	13.827277000	0.460704000
1	2.415521000	13.361772000	-0.517224000
1	3.783603000	14.188898000	-1.280426000
6	3.674406000	11.046160000	0.235808000
1	3.925115000	11.380590000	1.248508000
1	4.140878000	10.065822000	0.078747000
1	2.586250000	10.919748000	0.180484000
6	8.823222000	13.945718000	5.985855000
1	8.736990000	14.683907000	6.791942000
1	9.843063000	14.009451000	5.601874000
6	8.645320000	12.470684000	6.487762000
6	7.528390000	12.282104000	7.542274000
6	6.922330000	11.016501000	7.647859000
1	7.213573000	10.246287000	6.943898000
6	5.964040000	10.754322000	8.631671000
1	5.516412000	9.767270000	8.696655000
6	5.582012000	11.758327000	9.532815000
1	4.836882000	11.556816000	10.295982000
6	6.174621000	13.020976000	9.439274000
1	5.890689000	13.809572000	10.129697000
6	7.141788000	13.278168000	8.456046000
1	7.587157000	14.266502000	8.421526000
6	9.997760000	12.052124000	7.129188000
6	10.466430000	12.639228000	8.318578000
1	9.858522000	13.369874000	8.842323000
6	11.709002000	12.277774000	8.853084000
1	12.054536000	12.742066000	9.771996000
6	12.500918000	11.317110000	8.210583000
1	13.462345000	11.033281000	8.627436000
6	12.038621000	10.724072000	7.029369000
1	12.642091000	9.974714000	6.525300000
6	10.798421000	11.091803000	6.491992000
1	10.426872000	10.642759000	5.579371000
7	4.524555000	10.785734000	4.011569000
1	5.435779000	10.590182000	3.606973000
7	5.473550000	12.828252000	4.618954000
7	7.914665000	14.301180000	4.849108000
7	7.388990000	13.844013000	2.093757000
7	6.306617000	12.521674000	0.495526000
1	6.539686000	11.745151000	1.109396000
8	8.389261000	11.662333000	5.341381000
6	6.392230000	6.721176000	0.451959000
6	7.121300000	7.312983000	-0.768040000
6	8.510427000	7.226791000	-0.947338000
6	9.124156000	7.795103000	-2.074193000
6	8.357580000	8.459039000	-3.035864000
6	6.967767000	8.551106000	-2.866687000
6	6.358977000	7.982981000	-1.744894000
1	6.361954000	9.059994000	-3.610185000
1	10.200182000	7.716441000	-2.193979000
1	9.127915000	6.718526000	-0.216302000
1	5.283912000	8.052205000	-1.618217000
6	5.384780000	5.646967000	0.016347000
6	4.044277000	5.690017000	0.428086000
1	3.708232000	6.520473000	1.034985000
6	3.152520000	4.675251000	0.054292000
1	2.117554000	4.725284000	0.379200000
6	3.588882000	3.602834000	-0.731342000
6	4.926726000	3.553107000	-1.146351000
1	5.276812000	2.728372000	-1.759471000
6	5.814951000	4.568152000	-0.777294000
1	6.845946000	4.526212000	-1.115771000
17	7.524003000	10.191047000	2.446444000

1	8.832444000	8.897802000	-3.907642000
1	2.897603000	2.817021000	-1.019353000
6	7.344053000	6.134370000	1.513234000
1	6.751083000	5.783250000	2.361933000
1	7.913142000	5.286207000	1.121152000
1	8.047494000	6.894063000	1.870344000

$^{5}Re_{1Br,PP}$:

26	6.110258000	12.596821000	3.188089000
8	4.768434000	11.231144000	2.932336000
1	5.053253000	10.411725000	2.485871000
6	3.845351000	8.716024000	5.740778000
1	4.727756000	9.227088000	5.339771000
1	3.218341000	8.404242000	4.895532000
1	4.184021000	7.812253000	6.261295000
6	1.818121000	8.875663000	7.238349000
1	2.122452000	7.972511000	7.780052000
1	1.157480000	8.569856000	6.417270000
1	1.237706000	9.503037000	7.926747000
6	3.960033000	10.044221000	7.888060000
1	4.819762000	10.626808000	7.540930000
1	4.339256000	9.155821000	8.407694000
1	3.406674000	10.649242000	8.617018000
6	3.057831000	9.631385000	6.705683000
6	2.541236000	10.888025000	5.938777000
1	1.861791000	10.544163000	5.148893000
1	1.941895000	11.505466000	6.615386000
6	4.076558000	12.882760000	5.743033000
6	3.653103000	13.501463000	6.951414000
1	2.895026000	13.032302000	7.561597000
6	4.235587000	14.689903000	7.354436000
1	3.917600000	15.159273000	8.279319000
6	5.248591000	15.274552000	6.579132000
1	5.739480000	16.188523000	6.889749000
6	5.622577000	14.643847000	5.396490000
6	6.661805000	15.307701000	4.519880000
1	6.160768000	16.101984000	3.955212000
1	7.410242000	15.797209000	5.153574000
6	7.432582000	15.108973000	2.200741000
1	8.079937000	14.507131000	1.556316000
1	7.878304000	16.107036000	2.299515000
6	6.068603000	15.192737000	1.565014000
6	5.728368000	16.249569000	0.730294000
1	6.414767000	17.074055000	0.584148000
6	4.483878000	16.204673000	0.078136000
1	4.188639000	17.008190000	-0.588012000
6	3.635458000	15.130538000	0.277559000
1	2.687305000	15.081404000	-0.237877000
6	4.019578000	14.080205000	1.161149000
6	1.951162000	12.806531000	0.685369000
1	2.133992000	12.632723000	-0.387442000
1	1.356869000	13.726388000	0.765577000
6	1.089117000	11.642463000	1.237391000
6	-0.187722000	11.575123000	0.364508000
1	-0.849162000	10.777377000	0.721271000
1	0.060296000	11.365994000	-0.683346000
1	-0.744524000	12.519517000	0.403265000
6	0.690828000	11.926663000	2.702946000
1	1.572836000	12.039060000	3.341361000
1	0.080963000	11.106213000	3.099824000
1	0.102343000	12.850169000	2.773661000
6	1.837452000	10.292494000	1.134335000
1	2.719217000	10.262876000	1.782214000
1	2.160654000	10.102102000	0.103233000
1	1.174232000	9.473035000	1.436534000
6	8.680042000	13.888687000	3.955713000
1	9.272426000	14.682633000	4.423201000
1	9.192049000	13.575662000	3.044978000
6	8.544244000	12.619549000	4.854635000
6	8.348365000	12.916170000	6.360782000
6	7.557917000	12.044967000	7.129569000
1	7.046837000	11.231166000	6.630315000
6	7.425735000	12.226379000	8.510283000
1	6.810016000	11.541716000	9.085484000
6	8.082456000	13.285339000	9.150388000

1	7.979534000	13.427547000	10.221454000
6	8.876881000	14.155421000	8.395815000
1	9.395436000	14.977995000	8.878626000
6	9.012537000	13.969341000	7.013690000
1	9.649552000	14.651921000	6.461698000
6	9.819433000	11.753974000	4.707708000
6	11.102237000	12.319946000	4.817830000
1	11.222023000	13.382946000	5.001132000
6	12.246650000	11.521464000	4.699946000
1	13.228982000	11.975666000	4.785759000
6	12.125425000	10.143533000	4.478225000
1	13.012262000	9.524073000	4.389810000
6	10.850973000	9.573468000	4.373577000
1	10.745018000	8.506652000	4.201574000
6	9.706756000	10.372947000	4.486106000
1	8.718719000	9.939487000	4.395923000
7	3.575376000	11.701228000	5.296593000
1	3.958015000	11.359168000	4.399743000
7	5.042957000	13.492203000	4.962786000
7	7.346699000	14.420448000	3.525105000
7	5.236183000	14.139450000	1.804742000
7	3.226845000	13.000578000	1.386101000
1	3.638336000	12.237059000	1.939998000
8	7.415267000	11.888152000	4.351590000
6	12.226897000	12.926893000	-0.442708000
6	13.578201000	12.623264000	-0.891289000
6	14.114011000	13.134371000	-2.108851000
6	15.394083000	12.790604000	-2.541359000
6	16.196295000	11.924476000	-1.782751000
6	15.688495000	11.396339000	-0.586836000
6	14.405725000	11.729620000	-0.152149000
1	16.296641000	10.722803000	0.009563000
1	15.764974000	13.189123000	-3.480919000
1	13.501104000	13.776216000	-2.730655000
1	14.042306000	11.313389000	0.780263000
6	11.523219000	14.146273000	-0.834361000
6	12.221329000	15.357387000	-1.106225000
1	13.300493000	15.379124000	-1.009097000
6	11.545542000	16.528071000	-1.451165000
1	12.111372000	17.435865000	-1.638016000
6	10.146282000	16.542288000	-1.543046000
6	9.433753000	15.365370000	-1.271094000
1	8.349775000	15.359546000	-1.338563000
6	10.103098000	14.192801000	-0.915820000
1	9.517612000	13.301683000	-0.720674000
35	7.423619000	11.756216000	0.971263000
1	17.192901000	11.660386000	-2.121228000
1	9.622126000	17.452670000	-1.815419000
6	11.567040000	11.963283000	0.517029000
1	11.764515000	10.920076000	0.244338000
1	11.948524000	12.099708000	1.542191000
1	10.485132000	12.096261000	0.572145000

$^5TS_{OH,1Br,PP}$:

26	8.478605000	13.155384000	3.698949000
8	7.503609000	12.177693000	2.134572000
1	7.463016000	11.245854000	2.439241000
6	4.464831000	9.473613000	3.709618000
1	5.373190000	9.845437000	4.196604000
1	4.700936000	9.256592000	2.661714000
1	4.181885000	8.531399000	4.194856000
6	2.051733000	9.951652000	3.128990000
1	1.721228000	9.025069000	3.612947000
1	2.244233000	9.730650000	2.071880000
1	1.226003000	10.672835000	3.181922000
6	3.010190000	10.759817000	5.315606000
1	3.884263000	11.154919000	5.843733000
1	2.717943000	9.822204000	5.805267000
1	2.183949000	11.471095000	5.436756000
6	3.319435000	10.502951000	3.824002000
6	3.708698000	11.823700000	3.083101000
1	3.815021000	11.590149000	2.018259000
1	2.877673000	12.535452000	3.164120000
6	5.115783000	13.365910000	4.505939000
6	4.023000000	14.007002000	5.144120000

1	3.009469000	13.795711000	4.833723000
6	4.271982000	14.910357000	6.166565000
1	3.444835000	15.419561000	6.650454000
6	5.589133000	15.152521000	6.578579000
1	5.807408000	15.824223000	7.400547000
6	6.624597000	14.481936000	5.922892000
6	8.021921000	14.591120000	6.496797000
1	8.173472000	15.611004000	6.874940000
1	8.051906000	13.932710000	7.369664000
6	9.828341000	15.430337000	5.067777000
1	10.758517000	15.104712000	4.592561000
1	10.076586000	16.134774000	5.875090000
6	8.992768000	16.127295000	4.017691000
6	9.036885000	17.510321000	3.873785000
1	9.610384000	18.113909000	4.566437000
6	8.331528000	18.089984000	2.807433000
1	8.351181000	19.164668000	2.660365000
6	7.597351000	17.293243000	1.943785000
1	7.038838000	17.741232000	1.136136000
6	7.561891000	15.887467000	2.148675000
6	6.221050000	15.414396000	0.074095000
1	6.401206000	14.590661000	-0.625764000
1	6.741678000	16.286508000	-0.337778000
6	4.686269000	15.696460000	0.094634000
6	4.280227000	16.093951000	-1.344456000
1	3.200734000	16.276452000	-1.401816000
1	4.526974000	15.298782000	-2.059298000
1	4.794989000	17.009241000	-1.662831000
6	4.325958000	16.841608000	1.065878000
1	4.649204000	16.612511000	2.087293000
1	3.239639000	16.993199000	1.081758000
1	4.787263000	17.788281000	0.760249000
6	3.927038000	14.419221000	0.510446000
1	4.237951000	14.087189000	1.505773000
1	4.114491000	13.603557000	-0.199782000
1	2.845838000	14.602583000	0.530886000
6	10.088266000	13.212540000	6.183152000
1	10.352127000	13.470736000	7.218030000
1	10.995746000	13.255807000	5.579852000
6	9.541480000	11.741159000	6.061667000
6	8.491934000	11.387588000	7.140268000
6	7.379638000	10.608552000	6.783144000
1	7.274459000	10.320507000	5.745163000
6	6.429300000	10.223466000	7.736148000
1	5.578841000	9.619678000	7.433737000
6	6.570850000	10.614258000	9.073783000
1	5.832833000	10.320265000	9.813597000
6	7.677608000	11.385501000	9.447592000
1	7.804631000	11.692015000	10.481521000
6	8.630509000	11.762650000	8.491261000
1	9.487351000	12.346309000	8.813186000
6	10.765215000	10.781546000	6.168513000
6	11.077243000	10.029718000	7.313968000
1	10.445610000	10.083920000	8.192068000
6	12.199442000	9.188190000	7.340783000
1	12.415755000	8.614790000	8.237542000
6	13.031403000	9.082500000	6.222307000
1	13.899065000	8.429991000	6.242638000
6	12.730448000	9.828173000	5.073919000
1	13.367831000	9.756582000	4.197086000
6	11.610213000	10.664628000	5.047031000
1	11.379816000	11.242101000	4.159125000
7	4.960604000	12.453847000	3.499264000
1	5.808712000	12.245399000	2.965878000
7	6.409162000	13.642039000	4.880979000
7	9.146232000	14.218148000	5.596716000
7	8.273105000	15.328911000	3.179003000
7	6.848221000	15.037559000	1.347373000
1	6.931287000	14.041688000	1.546175000
8	8.967737000	11.615280000	4.753469000
6	8.136133000	11.047081000	0.079268000
6	8.243220000	12.143716000	-0.892303000
6	7.533639000	12.106448000	-2.122872000
6	7.725043000	13.091534000	-3.093347000
6	8.621583000	14.143308000	-2.864869000

6	9.333153000	14.194764000	-1.657580000
6	9.155829000	13.209010000	-0.687446000
1	10.027978000	15.007122000	-1.471047000
1	7.185531000	13.028934000	-4.032492000
1	6.872300000	11.277530000	-2.339702000
1	9.698937000	13.280251000	0.247561000
6	6.977119000	10.149902000	0.045122000
6	5.673385000	10.637669000	-0.226639000
1	5.520292000	11.704582000	-0.328796000
6	4.589454000	9.767718000	-0.335742000
1	3.601750000	10.163298000	-0.547110000
6	4.772916000	8.386675000	-0.168675000
6	6.047780000	7.887456000	0.129333000
1	6.196698000	6.821461000	0.263311000
6	7.134745000	8.755430000	0.246889000
1	8.115359000	8.345196000	0.453677000
35	10.973202000	13.426784000	2.509147000
1	8.769695000	14.908183000	-3.620153000
1	3.930370000	7.709872000	-0.262210000
6	9.408667000	10.559566000	0.714894000
1	9.997110000	10.004620000	-0.032689000
1	10.022576000	11.389452000	1.067935000
1	9.224334000	9.894624000	1.560707000

$^{5}P_{OH,1Br,PP}$:

26	7.497976000	12.383983000	3.863727000
8	5.575347000	7.742509000	1.009336000
1	6.199305000	8.421491000	1.356659000
6	4.930729000	7.852552000	4.827497000
1	5.717853000	8.525962000	5.185223000
1	5.196331000	7.525949000	3.815059000
1	4.916140000	6.972421000	5.481638000
6	2.465777000	7.556580000	4.365109000
1	2.410610000	6.700494000	5.047650000
1	2.686679000	7.173134000	3.361176000
1	1.476781000	8.031769000	4.339184000
6	3.220824000	9.039604000	6.261237000
1	3.948862000	9.779631000	6.609865000
1	3.237316000	8.195972000	6.962461000
1	2.221213000	9.489987000	6.306057000
6	3.553013000	8.552140000	4.834760000
6	3.558337000	9.735464000	3.818969000
1	3.731357000	9.318985000	2.819630000
1	2.570189000	10.207330000	3.798786000
6	4.420725000	11.974648000	4.635784000
6	3.202314000	12.380543000	5.237647000
1	2.345727000	11.722746000	5.231763000
6	3.134397000	13.616149000	5.861611000
1	2.208920000	13.929520000	6.332867000
6	4.261083000	14.450849000	5.899979000
1	4.232410000	15.410489000	6.401675000
6	5.428572000	14.019457000	5.274390000
6	6.626796000	14.954674000	5.243461000
1	6.413483000	15.737543000	4.505196000
1	6.705922000	15.458573000	6.214534000
6	8.652086000	15.108775000	3.834038000
1	9.636657000	14.645523000	3.709820000
1	8.813361000	16.150744000	4.144260000
6	7.928169000	15.074046000	2.511279000
6	7.835160000	16.216340000	1.728299000
1	8.241421000	17.155309000	2.082607000
6	7.200508000	16.118379000	0.477369000
1	7.107526000	16.992396000	-0.158025000
6	6.687005000	14.906096000	0.058591000
1	6.187966000	14.827027000	-0.895655000
6	6.804858000	13.761823000	0.897703000
6	5.745536000	12.178908000	-0.765887000
1	6.150401000	11.196092000	-1.036619000
1	6.085098000	12.879553000	-1.535942000
6	4.189731000	12.085081000	-0.794200000
6	3.785777000	11.625676000	-2.214575000
1	2.695827000	11.538929000	-2.293275000
1	4.220575000	10.646952000	-2.451661000
1	4.123394000	12.342239000	-2.974170000
6	3.543469000	13.454741000	-0.491458000

1	3.845640000	13.827362000	0.493865000
1	2.450198000	13.366111000	-0.495583000
1	3.819865000	14.203399000	-1.244398000
6	3.711421000	11.048092000	0.245571000
1	3.964635000	11.370742000	1.261497000
1	4.173195000	10.067432000	0.076895000
1	2.622690000	10.926648000	0.190264000
6	8.828735000	13.997935000	6.033057000
1	8.724580000	14.745150000	6.828724000
1	9.852313000	14.067946000	5.660090000
6	8.658666000	12.527746000	6.548542000
6	7.526083000	12.339170000	7.586073000
6	6.918881000	11.073675000	7.684089000
1	7.220762000	10.302934000	6.985239000
6	5.946696000	10.812565000	8.654540000
1	5.498755000	9.825350000	8.714761000
6	5.551244000	11.817628000	9.548621000
1	4.795149000	11.616827000	10.301127000
6	6.144845000	13.080375000	9.462146000
1	5.850559000	13.869803000	10.147270000
6	7.126042000	13.336449000	8.492756000
1	7.571631000	14.324892000	8.462860000
6	10.004129000	12.127817000	7.215792000
6	10.454164000	12.738637000	8.400424000
1	9.836920000	13.476884000	8.902070000
6	11.690371000	12.391720000	8.958740000
1	12.021524000	12.874299000	9.873512000
6	12.494422000	11.422234000	8.345196000
1	13.450890000	11.149671000	8.780557000
6	12.050744000	10.805866000	7.168809000
1	12.663871000	10.049702000	6.687050000
6	10.816898000	11.158975000	6.607471000
1	10.459361000	10.692532000	5.697926000
7	4.583954000	10.762813000	4.031507000
1	5.500695000	10.569831000	3.638126000
7	5.509592000	12.818337000	4.638944000
7	7.929326000	14.328221000	4.881829000
7	7.418189000	13.863428000	2.127689000
7	6.338801000	12.537464000	0.527691000
1	6.582571000	11.758550000	1.133237000
8	8.429886000	11.706562000	5.406127000
6	6.342190000	6.665858000	0.354693000
6	7.043158000	7.254598000	-0.883109000
6	8.428219000	7.170156000	-1.092292000
6	9.016751000	7.737365000	-2.233005000
6	8.228704000	8.398728000	-3.178921000
6	6.842723000	8.488968000	-2.980005000
6	6.258990000	7.921766000	-1.844517000
1	6.220296000	8.995690000	-3.711142000
1	10.090078000	7.660097000	-2.375732000
1	9.062112000	6.664272000	-0.373820000
1	5.186781000	7.990076000	-1.695155000
6	5.320372000	5.595172000	-0.054233000
6	3.992500000	5.639824000	0.396530000
1	3.675618000	6.469593000	1.014666000
6	3.088492000	4.627304000	0.046996000
1	2.063444000	4.678584000	0.401757000
6	3.500033000	3.555437000	-0.752682000
6	4.825159000	3.504028000	-1.206415000
1	5.155844000	2.679659000	-1.830699000
6	5.725482000	4.516991000	-0.861792000
1	6.746205000	4.473976000	-1.229931000
35	7.705280000	10.080593000	2.462980000
1	8.684031000	8.836911000	-4.061359000
1	2.799272000	2.771338000	-1.021829000
6	7.318249000	6.078401000	1.393607000
1	6.744954000	5.726044000	2.255307000
1	7.879278000	5.231245000	0.988117000
1	8.026959000	6.840257000	1.735297000

$^{5}TS_{Br,1Br,PP}$:

26	7.249733000	13.451273000	3.794088000
8	5.842751000	12.669907000	2.614982000
1	6.138757000	12.094590000	1.885511000
6	4.184427000	9.327245000	3.537046000

1	5.165858000	9.813670000	3.560901000
1	3.692301000	9.601278000	2.594838000
1	4.340483000	8.241575000	3.534042000
6	1.934247000	9.077464000	4.656871000
1	2.032283000	7.985405000	4.651341000
1	1.413607000	9.374415000	3.737532000
1	1.302895000	9.355123000	5.510602000
6	4.021853000	9.312144000	6.058119000
1	4.994356000	9.804135000	6.168364000
1	4.188197000	8.227783000	6.060173000
1	3.410654000	9.559915000	6.934902000
6	3.324817000	9.747264000	4.751038000
6	3.104532000	11.292172000	4.730220000
1	2.577141000	11.543267000	3.800164000
1	2.435989000	11.571309000	5.551332000
6	4.810244000	12.760489000	5.859373000
6	4.204207000	12.725510000	7.144847000
1	3.306346000	12.147023000	7.309655000
6	4.789635000	13.419554000	8.192158000
1	4.334427000	13.390684000	9.176984000
6	5.972867000	14.142649000	7.984906000
1	6.458358000	14.673385000	8.795185000
6	6.523761000	14.154695000	6.703153000
6	7.766062000	14.990389000	6.457110000
1	7.451518000	16.040551000	6.420096000
1	8.431354000	14.896930000	7.323811000
6	8.801946000	15.956398000	4.451439000
1	9.469284000	15.694162000	3.624171000
1	9.305766000	16.702892000	5.081986000
6	7.531914000	16.529223000	3.870030000
6	7.295931000	17.900941000	3.845876000
1	7.998943000	18.586596000	4.302858000
6	6.123370000	18.359888000	3.220943000
1	5.897098000	19.421004000	3.199874000
6	5.250331000	17.459147000	2.633324000
1	4.342550000	17.805500000	2.158898000
6	5.551204000	16.065972000	2.669021000
6	3.608196000	15.481434000	1.247826000
1	3.857325000	16.345546000	0.615245000
1	2.766340000	15.787815000	1.890111000
6	3.124583000	14.339747000	0.313665000
6	1.941149000	14.899203000	-0.511757000
1	1.549300000	14.128980000	-1.186214000
1	2.253598000	15.756085000	-1.121593000
1	1.121834000	15.224829000	0.141265000
6	2.640293000	13.122113000	1.134732000
1	3.460191000	12.675990000	1.706400000
1	2.233094000	12.352768000	0.466972000
1	1.847056000	13.415347000	1.834353000
6	4.260209000	13.913314000	-0.645344000
1	5.115699000	13.511514000	-0.092833000
1	4.609128000	14.765160000	-1.242394000
1	3.905051000	13.138381000	-1.335725000
6	9.749514000	13.868015000	5.388351000
1	10.331792000	14.223339000	6.249000000
1	10.352274000	14.012420000	4.490083000
6	9.432697000	12.335859000	5.461594000
6	9.007119000	11.843538000	6.870732000
6	8.062377000	10.806721000	6.961634000
1	7.620792000	10.437592000	6.044383000
6	7.692884000	10.273232000	8.200840000
1	6.960576000	9.472283000	8.244898000
6	8.260785000	10.769709000	9.381757000
1	7.973062000	10.359613000	10.344887000
6	9.205801000	11.798990000	9.305922000
1	9.656280000	12.193171000	10.212108000
6	9.578990000	12.326433000	8.061803000
1	10.321376000	13.117451000	8.038962000
6	10.724221000	11.562735000	5.072922000
6	11.953575000	11.793933000	5.717182000
1	12.026116000	12.525652000	6.514933000
6	13.101252000	11.079887000	5.350894000
1	14.041073000	11.276798000	5.858137000
6	13.038030000	10.113240000	4.339378000
1	13.926179000	9.555450000	4.058889000

6	11.817506000	9.873057000	3.695975000
1	11.755291000	9.121632000	2.913838000
6	10.672658000	10.595763000	4.056801000
1	9.725478000	10.430465000	3.559276000
7	4.321705000	12.099016000	4.775171000
1	4.850786000	12.215588000	3.887707000
7	5.958434000	13.500795000	5.659183000
7	8.522282000	14.698644000	5.204493000
7	6.676496000	15.623502000	3.325495000
7	4.775250000	15.132024000	2.060073000
1	5.078604000	14.143537000	2.161939000
8	8.406477000	12.070557000	4.512667000
6	9.860538000	11.992619000	-0.607161000
6	9.731717000	12.885267000	-1.768287000
6	9.045124000	12.466923000	-2.936586000
6	9.009989000	13.274328000	-4.076271000
6	9.646055000	14.520825000	-4.078809000
6	10.328230000	14.953612000	-2.930349000
6	10.378151000	14.147149000	-1.796587000
1	10.812545000	15.924256000	-2.920382000
1	8.492775000	12.924460000	-4.963401000
1	8.580089000	11.489301000	-2.964268000
1	10.865315000	14.511695000	-0.900337000
6	8.854684000	10.971690000	-0.331614000
6	7.480730000	11.193594000	-0.616856000
1	7.171713000	12.150614000	-1.019372000
6	6.522938000	10.222435000	-0.333854000
1	5.478211000	10.418847000	-0.550280000
6	6.904673000	8.997550000	0.238143000
6	8.254265000	8.759468000	0.530034000
1	8.557043000	7.814498000	0.968074000
6	9.216034000	9.733953000	0.259339000
1	10.254360000	9.524393000	0.484521000
35	9.033644000	13.819245000	1.496765000
1	9.613021000	15.149391000	-4.962413000
1	6.157476000	8.242127000	0.456475000
6	11.232386000	11.847398000	-0.000242000
1	11.804875000	11.111345000	-0.588392000
1	11.790017000	12.782955000	-0.041487000
1	11.196839000	11.506976000	1.034723000

$^5P_{Br,1Br,PP}$:

26	6.029181000	13.302228000	3.510933000
8	4.646734000	12.007261000	2.823970000
1	4.944233000	11.201648000	2.359302000
6	3.750095000	9.025306000	4.976084000
1	4.619700000	9.596569000	4.632994000
1	3.036299000	8.962654000	4.144600000
1	4.081820000	8.007472000	5.214615000
6	1.842871000	8.885293000	6.625953000
1	2.110147000	7.851833000	6.876572000
1	1.107229000	8.855692000	5.812137000
1	1.360294000	9.331399000	7.504962000
6	4.106596000	9.710797000	7.382193000
1	4.991143000	10.305379000	7.129989000
1	4.439096000	8.692894000	7.620732000
1	3.653852000	10.136174000	8.286703000
6	3.099284000	9.686440000	6.212460000
6	2.636151000	11.132744000	5.853721000
1	1.898707000	11.058574000	5.043121000
1	2.113300000	11.567797000	6.711878000
6	4.276052000	13.022185000	6.136551000
6	3.968591000	13.292390000	7.498046000
1	3.239055000	12.693116000	8.024169000
6	4.637260000	14.314456000	8.154253000
1	4.414189000	14.517420000	9.196795000
6	5.606983000	15.076202000	7.482950000
1	6.147893000	15.868761000	7.985908000
6	5.862293000	14.784613000	6.144069000
6	6.832770000	15.654854000	5.362901000
1	6.290801000	16.564413000	5.073819000
1	7.642353000	15.975242000	6.031669000
6	7.322212000	16.004867000	2.973484000
1	7.927401000	15.582455000	2.164341000
1	7.753927000	16.983749000	3.229202000

6	5.912767000	16.184562000	2.459136000
6	5.451467000	17.434845000	2.059517000
1	6.073927000	18.312862000	2.181271000
6	4.165466000	17.519483000	1.495661000
1	3.770679000	18.480385000	1.182012000
6	3.398865000	16.377595000	1.342191000
1	2.406896000	16.435435000	0.915859000
6	3.918818000	15.118826000	1.767802000
6	1.931636000	13.890751000	0.952286000
1	1.982754000	14.444941000	0.003060000
1	1.156578000	14.379915000	1.565107000
6	1.465974000	12.444793000	0.634476000
6	0.123497000	12.557180000	-0.126935000
1	-0.262628000	11.559566000	-0.366116000
1	0.246257000	13.107721000	-1.067958000
1	-0.631720000	13.076155000	0.476663000
6	1.245358000	11.639242000	1.936391000
1	2.182078000	11.513248000	2.488440000
1	0.851476000	10.642636000	1.701211000
1	0.520326000	12.143299000	2.588413000
6	2.503381000	11.728009000	-0.260655000
1	3.464182000	11.616228000	0.251994000
1	2.673167000	12.287739000	-1.188993000
1	2.146778000	10.726881000	-0.530879000
6	8.762676000	14.461990000	4.260898000
1	9.374791000	15.058378000	4.950154000
1	9.232928000	14.524666000	3.277582000
6	8.749538000	12.935862000	4.630875000
6	8.647165000	12.654997000	6.151880000
6	8.011987000	11.470714000	6.567611000
1	7.564520000	10.839835000	5.809410000
6	7.947258000	11.120753000	7.920025000
1	7.453692000	10.199231000	8.214555000
6	8.515332000	11.953742000	8.893906000
1	8.464144000	11.685227000	9.944560000
6	9.150223000	13.134643000	8.496135000
1	9.594440000	13.791521000	9.238231000
6	9.218113000	13.479086000	7.138292000
1	9.723530000	14.399538000	6.866759000
6	10.090632000	12.338772000	4.114935000
6	11.335881000	12.737208000	4.634914000
1	11.384248000	13.449711000	5.451845000
6	12.527943000	12.210654000	4.122332000
1	13.479329000	12.531460000	4.536592000
6	12.495287000	11.270419000	3.084083000
1	13.419364000	10.859250000	2.689149000
6	11.259861000	10.864559000	2.564708000
1	11.220639000	10.133772000	1.761647000
6	10.068599000	11.397263000	3.074155000
1	9.106048000	11.094729000	2.681155000
7	3.693422000	12.035618000	5.402038000
1	4.009684000	11.937390000	4.415538000
7	5.215368000	13.792237000	5.483564000
7	7.397828000	15.054515000	4.121208000
7	5.169138000	15.050293000	2.344988000
7	3.232185000	13.958987000	1.621421000
1	3.684518000	13.099114000	2.005548000
8	7.662408000	12.337188000	3.940557000
6	6.604074000	7.959522000	-0.357522000
6	8.008808000	7.649334000	-0.856021000
6	8.239195000	7.358209000	-2.213722000
6	9.506966000	6.956652000	-2.655709000
6	10.566133000	6.839652000	-1.751919000
6	10.347567000	7.120539000	-0.395576000
6	9.083979000	7.516184000	0.046198000
1	11.161444000	7.034190000	0.316946000
1	9.659423000	6.736795000	-3.707384000
1	7.429552000	7.432050000	-2.928524000
1	8.933907000	7.747509000	1.094453000
6	5.632616000	8.442955000	-1.412304000
6	5.959097000	9.545877000	-2.229971000
1	6.897959000	10.065339000	-2.072370000
6	5.083848000	9.987528000	-3.222366000
1	5.353922000	10.840289000	-3.836494000
6	3.857925000	9.335280000	-3.422990000

6	3.520947000	8.242356000	-2.619822000
1	2.576635000	7.728164000	-2.765635000
6	4.399185000	7.800644000	-1.620563000
1	4.113556000	6.948608000	-1.016591000
1	11.548993000	6.534241000	-2.095507000
1	3.176694000	9.678536000	-4.194599000
6	6.080839000	6.850614000	0.558370000
1	5.950152000	5.933531000	-0.029821000
1	6.801079000	6.636938000	1.349553000
1	5.126754000	7.118317000	1.016790000
35	6.798402000	9.648894000	1.004802000

Data for the reaction of phenylmethylmethyl with 1_{Cl} and 1_{Br}:

$^5Re_{1Cl,PM}$:

26	6.465029000	12.132498000	3.099545000
17	8.010381000	11.255619000	1.255300000
8	5.115408000	10.814847000	2.711825000
1	5.374867000	9.949833000	2.337462000
6	3.738718000	8.554191000	5.717075000
1	4.699358000	9.022088000	5.474459000
1	3.315724000	8.145740000	4.790689000
1	3.932187000	7.716450000	6.397794000
6	1.430919000	8.873951000	6.692697000
1	1.585664000	8.044463000	7.392649000
1	0.968899000	8.467046000	5.784392000
1	0.721879000	9.574977000	7.151196000
6	3.398223000	10.112992000	7.675331000
1	4.328572000	10.654004000	7.472236000
1	3.629654000	9.288833000	8.361178000
1	2.710094000	10.794951000	8.190386000
6	2.775011000	9.568703000	6.371761000
6	2.470373000	10.729152000	5.374885000
1	1.960470000	10.297767000	4.504467000
1	1.762469000	11.426870000	5.834596000
6	4.087796000	12.665536000	5.285543000
6	3.475253000	13.407117000	6.332225000
1	2.602181000	13.014201000	6.832838000
6	4.019144000	14.618161000	6.722876000
1	3.555981000	15.183135000	7.524885000
6	5.178904000	15.101593000	6.098917000
1	5.641355000	16.030149000	6.410612000
6	5.736357000	14.351610000	5.066864000
6	6.959040000	14.898496000	4.360298000
1	6.630637000	15.715389000	3.707872000
1	7.630469000	15.341712000	5.104366000
6	8.021877000	14.523789000	2.178957000
1	8.708336000	13.846857000	1.663147000
1	8.508929000	15.502080000	2.281378000
6	6.757844000	14.639051000	1.365934000
6	6.588386000	15.662449000	0.442752000
1	7.337180000	16.438015000	0.341046000
6	5.435524000	15.644054000	-0.360514000
1	5.278809000	16.415652000	-1.106626000
6	4.500988000	14.635300000	-0.209589000
1	3.628825000	14.605368000	-0.846098000
6	4.699763000	13.627336000	0.778563000
6	2.572838000	12.483944000	0.201223000
1	2.796808000	12.051145000	-0.787338000
1	2.152376000	13.481313000	0.029039000
6	1.467290000	11.632393000	0.879831000
6	0.256050000	11.619756000	-0.084467000
1	-0.562443000	11.028443000	0.341803000
1	0.523738000	11.178185000	-1.052407000
1	-0.118075000	12.635488000	-0.263247000
6	1.047031000	12.278312000	2.218638000
1	1.889347000	12.332564000	2.915105000
1	0.247933000	11.694372000	2.691005000
1	0.671119000	13.296600000	2.057231000
6	1.941919000	10.178615000	1.106374000
1	2.789000000	10.123306000	1.797516000
1	2.244153000	9.716819000	0.157724000
1	1.126445000	9.578023000	1.527500000
6	8.955147000	13.365111000	4.159209000
1	9.513380000	14.162923000	4.661473000
1	9.573339000	12.972295000	3.351184000
6	8.626762000	12.167186000	5.103973000
6	8.221642000	12.585189000	6.538655000
6	7.219461000	11.865103000	7.209368000
1	6.699446000	11.080296000	6.674299000
6	6.891721000	12.160088000	8.537521000
1	6.114921000	11.590191000	9.038086000
6	7.558476000	13.185311000	9.219862000
1	7.303274000	13.416107000	10.249236000
6	8.561574000	13.906552000	8.562377000
1	9.090886000	14.700742000	9.079861000
6	8.894581000	13.604445000	7.235796000

1	9.692638000	14.166762000	6.762497000
6	9.863550000	11.243846000	5.219818000
6	11.160044000	11.765759000	5.377067000
1	11.325572000	12.838143000	5.394183000
6	12.260827000	10.911704000	5.518255000
1	13.254858000	11.332615000	5.633779000
6	12.081507000	9.523231000	5.512027000
1	12.934300000	8.861253000	5.624185000
6	10.792785000	8.997010000	5.358820000
1	10.641718000	7.921839000	5.352106000
6	9.693041000	9.850716000	5.211254000
1	8.696648000	9.448400000	5.078121000
7	3.638354000	11.452543000	4.870251000
1	4.142941000	11.036284000	4.070401000
7	5.198208000	13.179059000	4.639312000
7	7.723646000	13.925005000	3.516441000
7	5.837790000	13.650167000	1.554226000
7	3.804818000	12.624943000	0.987183000
1	4.113626000	11.859642000	1.602407000
8	7.552962000	11.442058000	4.483333000
6	8.111552000	6.591912000	2.658997000
6	6.738323000	6.671811000	2.306251000
6	5.775639000	5.834558000	2.954053000
6	4.427798000	5.882223000	2.612386000
1	3.720981000	5.231123000	3.117900000
1	6.116893000	5.147581000	3.723665000
6	6.254003000	7.563836000	1.296630000
1	6.951294000	8.215258000	0.781277000
6	4.901067000	7.600403000	0.962440000
1	4.560325000	8.276927000	0.184319000
6	3.976253000	6.763802000	1.613114000
1	2.925758000	6.795789000	1.343839000
1	8.386871000	5.873192000	3.428142000
6	9.208581000	7.384577000	2.016279000
1	10.136450000	7.307428000	2.590128000
1	9.426006000	7.028505000	0.995366000
1	8.956745000	8.450092000	1.925353000

$^{5}TS_{OH,1Cl,PM}$:

26	6.800733000	12.285618000	3.471859000
8	5.675449000	10.629476000	3.307167000
1	5.759835000	10.003488000	4.052707000
6	3.512721000	8.989672000	6.259180000
1	4.534655000	9.373012000	6.156745000
1	3.231922000	8.500519000	5.317930000
1	3.520971000	8.227074000	7.046924000
6	1.105312000	9.537030000	6.788700000
1	1.092615000	8.792400000	7.593214000
1	0.764811000	9.045459000	5.868569000
1	0.381556000	10.321566000	7.043600000
6	2.971563000	10.799759000	7.930516000
1	3.961800000	11.256369000	7.824347000
1	3.026150000	10.060014000	8.738650000
1	2.264033000	11.579608000	8.237893000
6	2.525921000	10.123543000	6.615744000
6	2.460749000	11.157771000	5.448478000
1	2.098823000	10.633651000	4.555246000
1	1.712907000	11.922611000	5.684442000
6	4.161176000	13.028879000	5.477821000
6	3.389858000	13.902846000	6.286202000
1	2.422824000	13.595356000	6.657372000
6	3.888079000	15.161054000	6.588220000
1	3.298849000	15.842929000	7.192530000
6	5.148666000	15.547627000	6.114924000
1	5.561059000	16.523069000	6.344266000
6	5.881471000	14.635805000	5.352557000
6	7.295871000	15.009928000	4.957291000
1	7.316796000	16.073342000	4.682475000
1	7.917373000	14.911476000	5.850837000
6	7.839225000	14.900764000	2.554432000
1	8.475716000	14.347118000	1.857210000
1	8.210223000	15.932396000	2.630413000
6	6.430466000	14.891524000	2.005242000
6	5.878803000	16.007371000	1.386208000
1	6.432986000	16.936238000	1.334004000

6	4.585071000	15.893894000	0.848559000
1	4.113696000	16.751306000	0.379878000
6	3.901266000	14.691100000	0.918878000
1	2.901661000	14.613676000	0.516388000
6	4.522864000	13.568803000	1.535391000
6	2.616165000	12.009034000	1.067636000
1	2.376050000	12.709202000	0.262739000
1	1.854420000	12.141606000	1.852283000
6	2.488216000	10.574209000	0.483147000
6	1.051674000	10.439823000	-0.077313000
1	0.896787000	9.436471000	-0.490571000
1	0.868657000	11.167229000	-0.877878000
1	0.303340000	10.600556000	0.708846000
6	2.698401000	9.507883000	1.583291000
1	3.689730000	9.577620000	2.042613000
1	2.592165000	8.502229000	1.158330000
1	1.945085000	9.616281000	2.374327000
6	3.505668000	10.376198000	-0.662310000
1	4.533813000	10.504184000	-0.309063000
1	3.332989000	11.102225000	-1.466773000
1	3.411659000	9.370197000	-1.088166000
6	9.290242000	13.709735000	4.204026000
1	9.903040000	14.513050000	4.634931000
1	9.743762000	13.406187000	3.260615000
6	9.247636000	12.440874000	5.133787000
6	9.016535000	12.802885000	6.617434000
6	8.078060000	12.080017000	7.370239000
1	7.518496000	11.298106000	6.873454000
6	7.865115000	12.366046000	8.724833000
1	7.132560000	11.793604000	9.286385000
6	8.590127000	13.384435000	9.354833000
1	8.423807000	13.609895000	10.403572000
6	9.537262000	14.107969000	8.618657000
1	10.112165000	14.896021000	9.095541000
6	9.750444000	13.816676000	7.265349000
1	10.502212000	14.379554000	6.720421000
6	10.586409000	11.666485000	4.956985000
6	10.874630000	11.116159000	3.691123000
1	10.165064000	11.248904000	2.881005000
6	12.049753000	10.391347000	3.471447000
1	12.249518000	9.979728000	2.486265000
6	12.964678000	10.190081000	4.514537000
1	13.876697000	9.626062000	4.344463000
6	12.685994000	10.721218000	5.776893000
1	13.380215000	10.569402000	6.598178000
6	11.507932000	11.451221000	5.995597000
1	11.315894000	11.847202000	6.984844000
7	3.728113000	11.793152000	5.091909000
1	4.321631000	11.316577000	4.411772000
7	5.406430000	13.407728000	5.025762000
7	7.914663000	14.200803000	3.870849000
7	5.762863000	13.708033000	2.103231000
7	3.948840000	12.331012000	1.588844000
1	4.455556000	11.607439000	2.100273000
8	8.173898000	11.611293000	4.657330000
6	6.980108000	8.671314000	2.516696000
6	6.939934000	7.893079000	3.715515000
6	7.653703000	8.355368000	4.863038000
6	7.648821000	7.614401000	6.042736000
6	6.942961000	6.402354000	6.114781000
6	6.228763000	5.935380000	4.997329000
6	6.218458000	6.668409000	3.814377000
1	5.683363000	4.999487000	5.057419000
1	8.192535000	7.976516000	6.909040000
1	8.167342000	9.313486000	4.809617000
1	5.666397000	6.296380000	2.958750000
6	6.362902000	8.266767000	1.224981000
17	8.186972000	11.742525000	1.184235000
1	7.671442000	9.504567000	2.474183000
1	6.286608000	9.124949000	0.556264000
1	7.005536000	7.521295000	0.726481000
1	5.374904000	7.812099000	1.339777000
1	6.945407000	5.826053000	7.034122000

$^{5}P_{OH,1Cl,PM}$:

26	7.085694000	12.421416000	3.614690000
8	4.752301000	7.366073000	3.280374000
1	5.354247000	8.130749000	3.123122000
6	4.625402000	8.585895000	6.596156000
1	5.494069000	9.254508000	6.625295000
1	4.697581000	7.963812000	5.696519000
1	4.677990000	7.931146000	7.474807000
6	2.105384000	8.414691000	6.614474000
1	2.125113000	7.788401000	7.514067000
1	2.132996000	7.752107000	5.740680000
1	1.151526000	8.957815000	6.602643000
6	3.253872000	10.280529000	7.870019000
1	4.079458000	11.000779000	7.883302000
1	3.335558000	9.661705000	8.772117000
1	2.309426000	10.835882000	7.929790000
6	3.306019000	9.390677000	6.609146000
6	3.190618000	10.244712000	5.307235000
1	3.143269000	9.554558000	4.457431000
1	2.249760000	10.805971000	5.318475000
6	4.389539000	12.479674000	5.360181000
6	3.338310000	13.201697000	5.985004000
1	2.407409000	12.709383000	6.226526000
6	3.525452000	14.540621000	6.286109000
1	2.723580000	15.102570000	6.753097000
6	4.752110000	15.166911000	6.005591000
1	4.925428000	16.203886000	6.265843000
6	5.754149000	14.415481000	5.399281000
6	7.144559000	15.005349000	5.241181000
1	7.066620000	16.091396000	5.098267000
1	7.665377000	14.847936000	6.190946000
6	7.929558000	15.183516000	2.897467000
1	8.722100000	14.799281000	2.246220000
1	8.135939000	16.247697000	3.079287000
6	6.612020000	15.036476000	2.175375000
6	5.983932000	16.147969000	1.629671000
1	6.401371000	17.136225000	1.775715000
6	4.802952000	15.954167000	0.893137000
1	4.279011000	16.802524000	0.466261000
6	4.308814000	14.676495000	0.710931000
1	3.401959000	14.517985000	0.145930000
6	4.996358000	13.565199000	1.281493000
6	3.411124000	11.994259000	0.204744000
1	3.555983000	12.505255000	-0.757950000
1	2.489987000	12.398670000	0.650681000
6	3.192721000	10.487330000	-0.084764000
6	1.986099000	10.395775000	-1.051608000
1	1.774078000	9.348040000	-1.292599000
1	2.188579000	10.925470000	-1.990524000
1	1.084068000	10.828402000	-0.601198000
6	2.855130000	9.717256000	1.211857000
1	3.696129000	9.700580000	1.910971000
1	2.603537000	8.676338000	0.975754000
1	1.990905000	10.166235000	1.717442000
6	4.437130000	9.873508000	-0.768240000
1	5.315664000	9.897436000	-0.115035000
1	4.680461000	10.412463000	-1.692465000
1	4.243994000	8.825989000	-1.029483000
6	9.384106000	14.090708000	4.581089000
1	9.791559000	14.896079000	5.204762000
1	9.981492000	14.051197000	3.668207000
6	9.509593000	12.677752000	5.249910000
6	9.154424000	12.657171000	6.759730000
6	8.524778000	11.515037000	7.285207000
1	8.267436000	10.714006000	6.603332000
6	8.229812000	11.416094000	8.649305000
1	7.743490000	10.523084000	9.030579000
6	8.557931000	12.462586000	9.521182000
1	8.326557000	12.389458000	10.579182000
6	9.189103000	13.603573000	9.013555000
1	9.450701000	14.422532000	9.676813000
6	9.488823000	13.697464000	7.647209000
1	9.985816000	14.593211000	7.288965000
6	10.982941000	12.213288000	5.089750000
6	11.260164000	11.021959000	4.401910000
1	10.425563000	10.454919000	4.009171000

6	12.579777000	10.585056000	4.229536000
1	12.773703000	9.658052000	3.697803000
6	13.645622000	11.337986000	4.736743000
1	14.668926000	11.001423000	4.602018000
6	13.379376000	12.530415000	5.422119000
1	14.196873000	13.123424000	5.821285000
6	12.059423000	12.962092000	5.600134000
1	11.879321000	13.881778000	6.146959000
7	4.307155000	11.157315000	5.044947000
1	5.098063000	10.766228000	4.542743000
7	5.568015000	13.113477000	5.042082000
7	7.979500000	14.400722000	4.163145000
7	6.128130000	13.763095000	2.039148000
7	4.556073000	12.294354000	1.076872000
1	5.084056000	11.531204000	1.491633000
8	8.650195000	11.796871000	4.534301000
6	4.875436000	6.390520000	2.196573000
6	6.279977000	5.809670000	2.106339000
6	6.807152000	5.429491000	0.861030000
6	8.077780000	4.848271000	0.770855000
6	8.840574000	4.646455000	1.928303000
6	8.324836000	5.029729000	3.173597000
6	7.052302000	5.606122000	3.261857000
1	8.913460000	4.881819000	4.073897000
1	8.473333000	4.561720000	-0.198768000
1	6.222800000	5.592834000	-0.040994000
1	6.654297000	5.914106000	4.222559000
6	3.818304000	5.319242000	2.476971000
17	6.519970000	10.095889000	2.674631000
1	4.639431000	6.883841000	1.242480000
1	2.824661000	5.775979000	2.529231000
1	3.818307000	4.562898000	1.685589000
1	4.027759000	4.824818000	3.431617000
1	9.828183000	4.201147000	1.860057000

$^{5}TS_{Cl,1Cl,PM}$:

26	6.266910000	11.990751000	3.533245000
17	7.707187000	10.936411000	1.572601000
8	4.771760000	10.735106000	3.185020000
1	5.012974000	9.825345000	2.930258000
6	2.829604000	9.010829000	6.205660000
1	3.856298000	9.290439000	5.943546000
1	2.360847000	8.565147000	5.318721000
1	2.874723000	8.241737000	6.986339000
6	0.574328000	9.815002000	7.011358000
1	0.557139000	9.037810000	7.784558000
1	0.077982000	9.414091000	6.118474000
1	-0.015004000	10.666445000	7.375002000
6	2.688192000	10.811669000	7.970808000
1	3.711951000	11.144205000	7.766347000
1	2.730321000	10.047980000	8.757117000
1	2.120963000	11.665593000	8.361372000
6	2.028333000	10.237514000	6.698535000
6	1.971717000	11.316432000	5.571869000
1	1.482723000	10.866493000	4.698052000
1	1.329405000	12.141056000	5.897414000
6	3.814796000	13.027996000	5.468545000
6	3.160938000	13.991274000	6.284802000
1	2.180793000	13.786888000	6.691335000
6	3.791526000	15.194826000	6.556540000
1	3.290380000	15.938544000	7.167668000
6	5.077243000	15.445515000	6.055919000
1	5.598886000	16.368624000	6.279414000
6	5.690100000	14.458513000	5.283440000
6	7.138095000	14.666648000	4.879176000
1	7.261553000	15.697518000	4.522975000
1	7.740547000	14.580718000	5.788813000
6	7.843352000	14.369003000	2.533520000
1	8.446296000	13.698093000	1.913203000
1	8.372950000	15.328614000	2.614895000
6	6.507552000	14.561889000	1.856110000
6	6.227838000	15.702333000	1.109460000
1	6.941659000	16.515492000	1.061291000
6	4.998877000	15.762552000	0.429325000
1	4.738098000	16.644415000	-0.146663000

6	4.114562000	14.698218000	0.494528000
1	3.163376000	14.744077000	-0.017467000
6	4.459078000	13.546024000	1.259767000
6	2.455420000	12.284773000	0.519789000
1	2.639278000	12.691247000	-0.484370000
1	1.623129000	12.864466000	0.951779000
6	1.995611000	10.810737000	0.352787000
6	0.780040000	10.821147000	-0.605250000
1	0.397744000	9.803425000	-0.745514000
1	1.054834000	11.218535000	-1.590153000
1	-0.035208000	11.435920000	-0.203647000
6	1.562566000	10.208576000	1.709863000
1	2.400694000	10.158376000	2.411954000
1	1.179378000	9.190785000	1.566239000
1	0.763816000	10.808782000	2.164031000
6	3.127526000	9.958691000	-0.266014000
1	4.001566000	9.915475000	0.391725000
1	3.446302000	10.372487000	-1.230967000
1	2.781034000	8.931993000	-0.435006000
6	8.943085000	13.035448000	4.315630000
1	9.651351000	13.762733000	4.735245000
1	9.386403000	12.590162000	3.424078000
6	8.632708000	11.865322000	5.313540000
6	8.495219000	12.356032000	6.781662000
6	7.337988000	12.049610000	7.513909000
1	6.551103000	11.498769000	7.015139000
6	7.203936000	12.457739000	8.847494000
1	6.300013000	12.210355000	9.396610000
6	8.226457000	13.181947000	9.471440000
1	8.123071000	13.500211000	10.504181000
6	9.388337000	13.489901000	8.750919000
1	10.191249000	14.047008000	9.224390000
6	9.522572000	13.076107000	7.420451000
1	10.439502000	13.309482000	6.888044000
6	9.784437000	10.829577000	5.268995000
6	11.125203000	11.189572000	5.045416000
1	11.397334000	12.225133000	4.869062000
6	12.136884000	10.218999000	5.038376000
1	13.165160000	10.518088000	4.858634000
6	11.825544000	8.872902000	5.258843000
1	12.608990000	8.121567000	5.253741000
6	10.492131000	8.504239000	5.484411000
1	10.237995000	7.462471000	5.656386000
6	9.483511000	9.474123000	5.487678000
1	8.449115000	9.196977000	5.651066000
7	3.270517000	11.825707000	5.138072000
1	3.801051000	11.255063000	4.451938000
7	5.073355000	13.293479000	4.964763000
7	7.691964000	13.720457000	3.866754000
7	5.642292000	13.518522000	1.959954000
7	3.670323000	12.441523000	1.322322000
1	3.985597000	11.686008000	1.958545000
8	7.421739000	11.249060000	4.888360000
6	8.383376000	8.334040000	1.452582000
6	7.134097000	7.656348000	1.611406000
6	6.572396000	7.526747000	2.915100000
6	5.361175000	6.869268000	3.110840000
1	4.949568000	6.779166000	4.110619000
1	7.105043000	7.953500000	3.759158000
6	6.421253000	7.090814000	0.517388000
1	6.822316000	7.170931000	-0.486471000
6	5.212213000	6.429791000	0.722981000
1	4.683516000	5.999982000	-0.121355000
6	4.673881000	6.317444000	2.015432000
1	3.731002000	5.803296000	2.168634000
1	8.907336000	8.601602000	2.363800000
6	9.172802000	8.373420000	0.185765000
1	9.680871000	7.407009000	0.030746000
1	8.551575000	8.556273000	-0.695902000
1	9.942864000	9.145662000	0.231495000

$^5P_{Cl,1Cl,PM}$:

26	6.062662000	11.767893000	3.524816000
17	7.223571000	9.517771000	0.190473000
8	4.511865000	10.566177000	3.101159000

1	4.668078000	9.609631000	2.994187000
6	2.546515000	9.062804000	6.193137000
1	3.592605000	9.258574000	5.931838000
1	2.040308000	8.672604000	5.300903000
1	2.527624000	8.281420000	6.962525000
6	0.370030000	10.037121000	7.024648000
1	0.294202000	9.260510000	7.794875000
1	-0.160007000	9.680402000	6.132413000
1	-0.148630000	10.930933000	7.394175000
6	2.563181000	10.848298000	7.980522000
1	3.606357000	11.110015000	7.771375000
1	2.557023000	10.070406000	8.753973000
1	2.063324000	11.734235000	8.391515000
6	1.851584000	10.344567000	6.706249000
6	1.875621000	11.438033000	5.592565000
1	1.340519000	11.043285000	4.719133000
1	1.311926000	12.312311000	5.934501000
6	3.867641000	12.975587000	5.498260000
6	3.318137000	13.989134000	6.329973000
1	2.328263000	13.876999000	6.749022000
6	4.062108000	15.128854000	6.593909000
1	3.640164000	15.912385000	7.215042000
6	5.357020000	15.268599000	6.070113000
1	5.959501000	16.143914000	6.282291000
6	5.866910000	14.231601000	5.289542000
6	7.322379000	14.279424000	4.859695000
1	7.580873000	15.309763000	4.578471000
1	7.922423000	14.037868000	5.742883000
6	7.802776000	14.014990000	2.445324000
1	8.369350000	13.353753000	1.780407000
1	8.358047000	14.961593000	2.517677000
6	6.456075000	14.259852000	1.804176000
6	6.201155000	15.435567000	1.105200000
1	6.932854000	16.234075000	1.090004000
6	4.978316000	15.547737000	0.419339000
1	4.742081000	16.454686000	-0.127652000
6	4.071713000	14.501722000	0.440794000
1	3.126751000	14.584035000	-0.078279000
6	4.381753000	13.317946000	1.173843000
6	2.317648000	12.168419000	0.431094000
1	2.521404000	12.516567000	-0.591891000
1	1.548188000	12.839128000	0.848301000
6	1.722175000	10.737601000	0.333967000
6	0.494331000	10.820527000	-0.604645000
1	0.021117000	9.836312000	-0.699838000
1	0.784268000	11.155296000	-1.608364000
1	-0.255820000	11.519727000	-0.214197000
6	1.263050000	10.234388000	1.722798000
1	2.107172000	10.143278000	2.413603000
1	0.790515000	9.248672000	1.630050000
1	0.526876000	10.920813000	2.160725000
6	2.759599000	9.760341000	-0.265329000
1	3.639189000	9.665760000	0.379449000
1	3.095206000	10.102699000	-1.252236000
1	2.317619000	8.763624000	-0.384669000
6	8.956658000	12.559583000	4.086029000
1	9.693785000	13.200188000	4.588627000
1	9.383168000	12.241404000	3.132646000
6	8.647805000	11.257993000	4.894816000
6	8.413190000	11.550114000	6.403479000
6	7.246377000	11.099068000	7.037549000
1	6.510002000	10.573071000	6.443573000
6	7.038347000	11.326214000	8.404943000
1	6.128601000	10.966753000	8.877187000
6	7.996110000	12.010595000	9.161803000
1	7.835014000	12.188902000	10.220455000
6	9.170967000	12.457401000	8.541277000
1	9.926533000	12.981564000	9.118875000
6	9.379142000	12.222586000	7.177458000
1	10.307606000	12.553704000	6.721388000
6	9.848759000	10.282023000	4.809911000
6	11.162275000	10.694184000	4.524153000
1	11.384431000	11.736170000	4.321380000
6	12.216909000	9.769288000	4.496003000
1	13.222341000	10.110946000	4.268991000

6	11.977640000	8.417137000	4.758888000
1	12.793723000	7.701663000	4.739468000
6	10.671719000	7.995278000	5.049125000
1	10.472720000	6.948197000	5.257668000
6	9.622176000	8.918594000	5.073297000
1	8.612393000	8.591207000	5.288591000
7	3.209712000	11.836085000	5.147882000
1	3.666892000	11.232541000	4.434973000
7	5.141288000	13.125240000	4.990064000
7	7.718622000	13.339281000	3.771192000
7	5.569371000	13.230346000	1.866905000
7	3.552054000	12.244899000	1.214747000
1	3.827732000	11.470829000	1.855565000
8	7.495136000	10.655346000	4.302571000
6	8.003254000	8.470898000	1.706911000
6	7.011131000	7.425183000	2.112663000
6	6.331950000	7.582579000	3.337294000
6	5.425608000	6.607895000	3.774153000
1	4.914142000	6.734185000	4.723289000
1	6.540369000	8.461240000	3.942766000
6	6.759608000	6.281885000	1.332003000
1	7.263361000	6.153681000	0.379428000
6	5.849112000	5.314732000	1.767420000
1	5.659962000	4.438436000	1.155765000
6	5.180512000	5.474249000	2.989949000
1	4.476020000	4.720193000	3.326120000
1	8.072179000	9.255415000	2.461717000
6	9.360119000	7.989033000	1.230389000
1	9.854704000	7.475988000	2.064755000
1	9.281935000	7.288773000	0.394290000
1	9.989575000	8.829069000	0.926407000

^{5}Re$_{1Br,PM}$:

26	6.383961000	12.420214000	3.696246000
8	4.904659000	11.178887000	3.605170000
1	5.107846000	10.233896000	3.475763000
6	3.070263000	9.687818000	6.658888000
1	4.082117000	9.936619000	6.319179000
1	2.524010000	9.251017000	5.812966000
1	3.153191000	8.921469000	7.438817000
6	0.909071000	10.549493000	7.642584000
1	0.942155000	9.788366000	8.430952000
1	0.334701000	10.139861000	6.802060000
1	0.366219000	11.418799000	8.034954000
6	3.116705000	11.508811000	8.409641000
1	4.120057000	11.834545000	8.113946000
1	3.224906000	10.746233000	9.190624000
1	2.592770000	12.366791000	8.849141000
6	2.340194000	10.936935000	7.203713000
6	2.209823000	12.005876000	6.075350000
1	1.613444000	11.569533000	5.263488000
1	1.642404000	12.862651000	6.451670000
6	4.102725000	13.635235000	5.727945000
6	3.615091000	14.607627000	6.643746000
1	2.704687000	14.422375000	7.194872000
6	4.326166000	15.776927000	6.845751000
1	3.958748000	16.516459000	7.549349000
6	5.527186000	15.996107000	6.155248000
1	6.112977000	16.893250000	6.313276000
6	5.962611000	15.023923000	5.258993000
6	7.221291000	15.299089000	4.464450000
1	6.970861000	16.036520000	3.693371000
1	7.960116000	15.773467000	5.118754000
6	8.121831000	14.447580000	2.346634000
1	8.685525000	13.609493000	1.925597000
1	8.724689000	15.359768000	2.255834000
6	6.822395000	14.581278000	1.592516000
6	6.647101000	15.524474000	0.589187000
1	7.433122000	16.232472000	0.358610000
6	5.421956000	15.535140000	-0.103104000
1	5.240233000	16.275591000	-0.874670000
6	4.444143000	14.604431000	0.198899000
1	3.498221000	14.614207000	-0.323636000
6	4.684988000	13.633093000	1.214548000
6	2.575182000	12.410488000	0.733105000

1	2.779567000	12.593497000	-0.330005000
1	1.792150000	13.121521000	1.039654000
6	2.016905000	10.967297000	0.862448000
6	0.779139000	10.879527000	-0.062916000
1	0.331016000	9.881144000	-0.003963000
1	1.052335000	11.067114000	-1.108596000
1	0.014875000	11.610976000	0.227849000
6	1.581383000	10.667877000	2.315906000
1	2.433870000	10.675381000	3.002687000
1	1.115095000	9.676828000	2.372417000
1	0.848249000	11.406661000	2.663439000
6	3.073735000	9.936270000	0.402993000
1	3.963354000	9.959846000	1.041167000
1	3.390951000	10.133658000	-0.628511000
1	2.656307000	8.922967000	0.441178000
6	9.030587000	13.536880000	4.479182000
1	9.709163000	14.326678000	4.821819000
1	9.559326000	12.936886000	3.736861000
6	8.611546000	12.561186000	5.628207000
6	8.254763000	13.247703000	6.963017000
6	7.296540000	12.644653000	7.797275000
1	6.794213000	11.750248000	7.450191000
6	6.988878000	13.188095000	9.048410000
1	6.245852000	12.705164000	9.675658000
6	7.635489000	14.348871000	9.493810000
1	7.396579000	14.771863000	10.464338000
6	8.595492000	14.954282000	8.676879000
1	9.108634000	15.851082000	9.009940000
6	8.904430000	14.406467000	7.423741000
1	9.663470000	14.892941000	6.820591000
6	9.771657000	11.554980000	5.844472000
6	10.881154000	11.859365000	6.651930000
1	10.925038000	12.800616000	7.187941000
6	11.934634000	10.946612000	6.790742000
1	12.782424000	11.198741000	7.420532000
6	11.892520000	9.713817000	6.128895000
1	12.706781000	9.004655000	6.241219000
6	10.790806000	9.403954000	5.321678000
1	10.747359000	8.453389000	4.798446000
6	9.740260000	10.318053000	5.178106000
1	8.896144000	10.092699000	4.539274000
7	3.474612000	12.454813000	5.493838000
1	3.929639000	11.828650000	4.810602000
7	5.271070000	13.876825000	5.025240000
7	7.839700000	14.120455000	3.779861000
7	5.858956000	13.677313000	1.932214000
7	3.805475000	12.640266000	1.498412000
1	4.074497000	11.987816000	2.248162000
8	7.457310000	11.852832000	5.134628000
6	6.993721000	6.852583000	2.017458000
6	8.289030000	6.276454000	2.110719000
6	9.448765000	7.115608000	2.087640000
6	10.728518000	6.576942000	2.176037000
6	10.911860000	5.186194000	2.292216000
6	9.788829000	4.341546000	2.316960000
6	8.501812000	4.867525000	2.228069000
1	9.924336000	3.267580000	2.405990000
1	11.591934000	7.235325000	2.152310000
1	9.308202000	8.189437000	1.996737000
1	7.646947000	4.199467000	2.249194000
6	5.714586000	6.070726000	2.020411000
35	7.773654000	10.926136000	1.898878000
1	6.935627000	7.936206000	1.942497000
1	4.851358000	6.728422000	1.883827000
1	5.687624000	5.316196000	1.219378000
1	5.565100000	5.521212000	2.963393000
1	11.911566000	4.769648000	2.360885000

^{5}TS$_{OH,1Br,PM}$:

26	6.778848000	12.306095000	3.480307000
8	5.653193000	10.654247000	3.293355000
1	5.731970000	10.004203000	4.017754000
6	3.501067000	9.001587000	6.255529000
1	4.525105000	9.379824000	6.154106000
1	3.215711000	8.521029000	5.311245000

1	3.506031000	8.233441000	7.037885000
6	1.097909000	9.558936000	6.793921000
1	1.082824000	8.809783000	7.594184000
1	0.753212000	9.074746000	5.871493000
1	0.379180000	10.346113000	7.054593000
6	2.973648000	10.803401000	7.940114000
1	3.966638000	11.254633000	7.835387000
1	3.025194000	10.057734000	8.742984000
1	2.271446000	11.585392000	8.254002000
6	2.521431000	10.138590000	6.621681000
6	2.459273000	11.180568000	5.461396000
1	2.092859000	10.664658000	4.565294000
1	1.716716000	11.948408000	5.703711000
6	4.170803000	13.041117000	5.498633000
6	3.408088000	13.911902000	6.318848000
1	2.442781000	13.604649000	6.694452000
6	3.911907000	15.166395000	6.626296000
1	3.328875000	15.845447000	7.239602000
6	5.170180000	15.553412000	6.146232000
1	5.586239000	16.526563000	6.378467000
6	5.895044000	14.645429000	5.372535000
6	7.305777000	15.019516000	4.965751000
1	7.325428000	16.082987000	4.692171000
1	7.936137000	14.919209000	5.852689000
6	7.823108000	14.915631000	2.557684000
1	8.452960000	14.363986000	1.851927000
1	8.193433000	15.947169000	2.633202000
6	6.410296000	14.904019000	2.019585000
6	5.849718000	16.019842000	1.409491000
1	6.397675000	16.952605000	1.362102000
6	4.554625000	15.901883000	0.874909000
1	4.076124000	16.759803000	0.414620000
6	3.879106000	14.694147000	0.937988000
1	2.878444000	14.612981000	0.538840000
6	4.509074000	13.571025000	1.545060000
6	2.617370000	12.002587000	1.051205000
1	2.387618000	12.696687000	0.237883000
1	1.845201000	12.139691000	1.824534000
6	2.500411000	10.563106000	0.475817000
6	1.071688000	10.422023000	-0.102678000
1	0.924606000	9.415683000	-0.511474000
1	0.897736000	11.144152000	-0.909980000
1	0.312961000	10.586088000	0.672641000
6	2.698964000	9.504115000	1.585230000
1	3.688487000	9.572852000	2.048730000
1	2.590276000	8.495921000	1.166930000
1	1.942366000	9.621839000	2.371695000
6	3.533428000	10.358385000	-0.654568000
1	4.556841000	10.486372000	-0.287253000
1	3.372544000	11.080539000	-1.464982000
1	3.444141000	9.350244000	-1.076304000
6	9.289515000	13.715693000	4.189147000
1	9.904887000	14.513090000	4.626675000
1	9.737300000	13.424621000	3.239161000
6	9.252361000	12.434424000	5.101155000
6	9.009640000	12.776367000	6.587790000
6	8.068853000	12.042709000	7.326996000
1	7.513505000	11.264162000	6.820256000
6	7.847199000	12.312970000	8.683737000
1	7.112812000	11.732340000	9.234351000
6	8.566068000	13.325852000	9.329431000
1	8.393025000	13.539185000	10.379563000
6	9.516070000	14.059652000	8.606921000
1	10.086274000	14.843656000	9.095820000
6	9.737717000	13.784169000	7.251742000
1	10.491222000	14.355198000	6.717820000
6	10.598052000	11.671317000	4.924927000
6	10.913373000	11.153375000	3.651725000
1	10.219999000	11.299328000	2.829883000
6	12.096065000	10.439206000	3.436370000
1	12.315924000	10.052853000	2.445196000
6	12.992633000	10.215768000	4.490715000
1	13.910379000	9.660277000	4.323785000
6	12.687249000	10.713899000	5.760428000
1	13.366444000	10.544847000	6.590779000

6	11.501851000	11.433236000	5.974615000
1	11.290505000	11.804321000	6.969454000
7	3.730272000	11.810666000	5.106027000
1	4.317067000	11.336035000	4.418527000
7	5.414968000	13.419609000	5.041497000
7	7.911719000	14.210695000	3.871071000
7	5.750186000	13.714606000	2.111621000
7	3.943277000	12.329638000	1.587807000
1	4.447335000	11.606500000	2.104191000
8	8.186588000	11.603467000	4.606001000
6	6.996222000	8.648059000	2.536608000
6	6.935087000	7.889960000	3.743954000
6	7.625696000	8.372556000	4.898136000
6	7.601696000	7.648139000	6.087452000
6	6.899071000	6.434114000	6.162036000
6	6.207069000	5.947517000	5.038357000
6	6.215882000	6.663313000	3.845602000
1	5.664178000	5.010719000	5.102565000
1	8.126515000	8.023895000	6.959411000
1	8.136614000	9.332016000	4.839023000
1	5.680804000	6.278453000	2.984998000
6	6.404675000	8.226212000	1.239265000
35	8.251827000	11.726959000	0.951567000
1	7.670780000	9.495814000	2.500800000
1	6.380133000	9.067280000	0.545325000
1	7.033526000	7.442656000	0.783595000
1	5.398782000	7.807714000	1.338293000
1	6.886133000	5.870923000	7.089251000

$^5P_{OH,1Br,PM}$:

26	6.678855000	12.219210000	3.117977000
8	6.372511000	9.349656000	4.589136000
1	7.141045000	10.004444000	4.549209000
6	3.509374000	8.641439000	6.814543000
1	4.509558000	9.086410000	6.794533000
1	3.491417000	7.820150000	6.088558000
1	3.341278000	8.214575000	7.810723000
6	1.041073000	8.990054000	6.426426000
1	0.811172000	8.508854000	7.384261000
1	1.024396000	8.216690000	5.648225000
1	0.241444000	9.709618000	6.209315000
6	2.400731000	10.761485000	7.599781000
1	3.350199000	11.305785000	7.647212000
1	2.236701000	10.286973000	8.575066000
1	1.594199000	11.487720000	7.444182000
6	2.420881000	9.688032000	6.489524000
6	2.677887000	10.314819000	5.080139000
1	2.669874000	9.500851000	4.345373000
1	1.841554000	10.973813000	4.822000000
6	4.103266000	12.389908000	5.038684000
6	3.071408000	13.271164000	5.469325000
1	2.069688000	12.902307000	5.633218000
6	3.374497000	14.600193000	5.713346000
1	2.593058000	15.275145000	6.046428000
6	4.692626000	15.070248000	5.561383000
1	4.956064000	16.093554000	5.798541000
6	5.649853000	14.184002000	5.083944000
6	7.110004000	14.578064000	4.992085000
1	7.204561000	15.671477000	4.990798000
1	7.605627000	14.212003000	5.895703000
6	7.835886000	14.938979000	2.658951000
1	8.492721000	14.511520000	1.892990000
1	8.247349000	15.916142000	2.948230000
6	6.451436000	15.103884000	2.081361000
6	5.935409000	16.353050000	1.757522000
1	6.513588000	17.249622000	1.943269000
6	4.644073000	16.415419000	1.205097000
1	4.201765000	17.375158000	0.960364000
6	3.934760000	15.250019000	0.966919000
1	2.944961000	15.287758000	0.533864000
6	4.527246000	13.996275000	1.286025000
6	2.589617000	12.729666000	0.366272000
1	2.582073000	13.335861000	-0.552484000
1	1.830478000	13.152108000	1.040342000
6	2.173854000	11.282749000	-0.009150000

6	0.746061000	11.364121000	-0.601163000
1	0.402563000	10.367283000	-0.899831000
1	0.721539000	12.009427000	-1.487957000
1	0.034490000	11.762429000	0.132530000
6	2.152768000	10.373693000	1.241462000
1	3.149384000	10.260939000	1.682273000
1	1.793029000	9.373265000	0.973443000
1	1.483860000	10.780693000	2.009471000
6	3.131588000	10.699665000	-1.075656000
1	4.157089000	10.608130000	-0.701461000
1	3.148196000	11.333623000	-1.970950000
1	2.795284000	9.699908000	-1.375449000
6	9.183207000	13.460742000	4.119289000
1	9.758885000	14.184552000	4.711254000
1	9.685525000	13.333333000	3.157899000
6	9.133926000	12.051269000	4.807296000
6	8.940266000	12.165505000	6.345326000
6	7.877686000	11.513715000	6.990295000
1	7.175430000	10.940765000	6.401892000
6	7.717352000	11.603772000	8.380039000
1	6.887447000	11.090497000	8.856736000
6	8.616197000	12.348556000	9.150770000
1	8.490972000	12.419375000	10.226659000
6	9.684405000	12.999383000	8.519006000
1	10.393970000	13.576584000	9.104052000
6	9.845725000	12.904124000	7.132719000
1	10.692109000	13.400803000	6.669241000
6	10.471855000	11.311880000	4.550065000
6	11.697810000	11.987127000	4.406635000
1	11.742375000	13.070262000	4.448591000
6	12.888928000	11.277353000	4.201207000
1	13.822653000	11.819964000	4.088310000
6	12.875962000	9.879875000	4.138654000
1	13.798175000	9.330099000	3.978891000
6	11.660195000	9.198312000	4.281864000
1	11.631986000	8.114025000	4.233564000
6	10.472424000	9.908591000	4.484514000
1	9.535010000	9.377224000	4.584833000
7	3.938624000	11.043077000	4.898608000
1	4.775912000	10.481024000	4.707424000
7	5.348933000	12.893924000	4.757873000
7	7.823185000	13.994996000	3.813516000
7	5.762127000	13.945275000	1.882052000
7	3.907043000	12.814867000	1.007116000
1	4.479464000	11.978504000	1.066336000
8	8.058569000	11.322367000	4.201896000
6	6.677427000	8.149891000	3.804233000
6	7.649804000	7.233051000	4.535598000
6	8.581976000	6.474396000	3.806576000
6	9.448929000	5.587597000	4.457768000
6	9.397800000	5.452650000	5.851383000
6	8.475984000	6.209986000	6.586555000
6	7.608158000	7.092829000	5.933107000
1	8.434886000	6.114444000	7.667456000
1	10.163257000	5.008667000	3.880252000
1	8.630398000	6.580814000	2.725893000
1	6.902308000	7.688344000	6.501471000
6	5.351675000	7.448031000	3.497469000
35	6.670138000	10.678894000	0.977303000
1	7.127869000	8.470160000	2.856529000
1	4.685663000	8.124264000	2.951601000
1	5.525248000	6.558571000	2.882236000
1	4.859714000	7.135742000	4.423744000
1	10.071481000	4.769064000	6.358566000

^{5}TS$_{Br,1Br,PM}$:

26	6.183643000	11.907344000	4.154773000
8	4.650191000	10.703951000	4.232507000
1	4.802454000	9.743043000	4.286664000
6	2.394607000	10.296240000	7.593291000
1	3.466895000	10.403730000	7.393878000
1	1.974888000	9.611514000	6.845152000
1	2.278064000	9.831673000	8.579900000
6	0.169086000	11.471785000	7.811112000
1	0.001755000	11.025538000	8.798431000

1	-0.278965000	10.808199000	7.060669000
1	-0.364160000	12.430509000	7.779265000
6	2.277595000	12.597124000	8.629369000
1	3.343482000	12.776792000	8.450090000
1	2.173813000	12.145276000	9.623512000
1	1.763858000	13.566489000	8.646217000
6	1.680946000	11.666509000	7.551348000
6	1.832785000	12.295775000	6.131755000
1	1.357530000	11.619271000	5.410188000
1	1.273507000	13.236414000	6.092319000
6	3.876231000	13.693624000	5.663716000
6	3.310716000	14.925135000	6.096330000
1	2.302035000	14.956034000	6.482592000
6	4.063641000	16.085014000	6.018162000
1	3.630313000	17.028824000	6.332666000
6	5.385515000	16.039834000	5.546737000
1	5.998843000	16.931603000	5.501333000
6	5.905051000	14.805705000	5.162323000
6	7.377634000	14.694873000	4.829102000
1	7.715937000	15.621147000	4.346246000
1	7.916223000	14.621005000	5.778013000
6	7.842295000	13.865850000	2.547834000
1	8.367582000	13.045255000	2.048268000
1	8.415008000	14.790170000	2.391115000
6	6.474314000	13.976922000	1.917663000
6	6.201888000	14.922201000	0.933832000
1	6.942708000	15.666115000	0.668029000
6	4.946534000	14.877728000	0.302542000
1	4.691840000	15.611738000	-0.454842000
6	4.029433000	13.897453000	0.645165000
1	3.059275000	13.863417000	0.169112000
6	4.365275000	12.943058000	1.648236000
6	2.305220000	11.603740000	1.275198000
1	2.465617000	11.774876000	0.202244000
1	1.488823000	12.274468000	1.588920000
6	1.833917000	10.133782000	1.451620000
6	0.594546000	9.944590000	0.544246000
1	0.199673000	8.927153000	0.645993000
1	0.847886000	10.105730000	-0.510879000
1	-0.204669000	10.645845000	0.815161000
6	1.431470000	9.852083000	2.918318000
1	2.282016000	9.961181000	3.598961000
1	1.050687000	8.827966000	3.015053000
1	0.637995000	10.537914000	3.241601000
6	2.945945000	9.153390000	1.011679000
1	3.836492000	9.246803000	1.642056000
1	3.243822000	9.341187000	-0.027592000
1	2.588102000	8.118995000	1.079974000
6	9.002071000	12.834638000	4.492859000
1	9.787604000	13.569355000	4.713279000
1	9.343872000	12.186389000	3.685204000
6	8.687878000	11.915902000	5.726121000
6	8.702750000	12.691195000	7.071909000
6	7.597557000	12.622604000	7.934518000
1	6.737896000	12.044103000	7.621951000
6	7.606465000	13.293115000	9.164722000
1	6.741508000	13.227411000	9.818239000
6	8.721816000	14.044676000	9.552428000
1	8.728716000	14.566037000	10.504518000
6	9.832758000	14.115027000	8.701111000
1	10.706451000	14.690332000	8.992074000
6	9.824328000	13.440444000	7.474976000
1	10.702933000	13.494192000	6.839823000
6	9.740215000	10.785528000	5.821968000
6	11.086629000	10.970788000	5.461964000
1	11.428085000	11.923115000	5.069211000
6	12.013208000	9.927686000	5.595843000
1	13.047416000	10.088986000	5.307143000
6	11.608736000	8.684500000	6.094613000
1	12.325996000	7.876293000	6.197286000
6	10.268644000	8.491875000	6.456494000
1	9.942657000	7.530222000	6.841529000
6	9.344547000	9.532929000	6.320355000
1	8.304321000	9.387194000	6.584938000
7	3.209562000	12.507962000	5.687985000

1	3.694823000	11.702255000	5.259102000
7	5.169400000	13.664416000	5.185905000
7	7.766297000	13.518216000	3.995593000
7	5.574788000	13.030099000	2.296042000
7	3.543498000	11.916293000	1.991582000
1	3.848135000	11.327829000	2.783392000
8	7.395819000	11.346866000	5.513711000
6	8.423791000	8.201797000	0.540929000
6	9.602487000	7.694333000	1.181882000
6	10.841832000	8.378216000	1.023719000
6	12.007181000	7.901427000	1.616230000
6	11.978704000	6.726814000	2.387441000
6	10.768052000	6.036600000	2.557483000
6	9.596302000	6.508772000	1.968402000
1	10.742397000	5.128776000	3.151834000
1	12.940772000	8.438029000	1.480847000
1	10.865889000	9.287629000	0.430370000
1	8.670358000	5.963027000	2.111347000
6	7.139828000	7.439466000	0.425905000
35	7.495040000	10.252007000	2.204479000
1	8.567642000	9.025957000	-0.150123000
1	6.334230000	8.084358000	0.069160000
1	7.249208000	6.609950000	-0.292316000
1	6.825044000	7.000691000	1.378147000
1	12.887721000	6.355326000	2.848845000

$^{5}P_{Br,1Br,PM}$:

26	6.386153000	11.849417000	3.791100000
8	4.992243000	10.414411000	3.675845000
1	5.273730000	9.480605000	3.671049000
6	3.375874000	9.184971000	7.101914000
1	4.380152000	9.459008000	6.759430000
1	2.892190000	8.602449000	6.307408000
1	3.480598000	8.537924000	7.981231000
6	1.127296000	10.014533000	7.898303000
1	1.180711000	9.369125000	8.782990000
1	0.612412000	9.457686000	7.105195000
1	0.514358000	10.888251000	8.153914000
6	3.235261000	11.223802000	8.587367000
1	4.229751000	11.567619000	8.281787000
1	3.354452000	10.586682000	9.472414000
1	2.645004000	12.100488000	8.881891000
6	2.544640000	10.440552000	7.450010000
6	2.385830000	11.335237000	6.180917000
1	1.870372000	10.744045000	5.412999000
1	1.730492000	12.180310000	6.416514000
6	4.158165000	13.064526000	5.728125000
6	3.518349000	14.121925000	6.431232000
1	2.572946000	13.956607000	6.928012000
6	4.115427000	15.372349000	6.469136000
1	3.622199000	16.186371000	6.990544000
6	5.354913000	15.585949000	5.845556000
1	5.844892000	16.551718000	5.880841000
6	5.961686000	14.508827000	5.200917000
6	7.377053000	14.664747000	4.675691000
1	7.497238000	15.673879000	4.257274000
1	8.045630000	14.603716000	5.540104000
6	7.718328000	14.134684000	2.282350000
1	8.325311000	13.468338000	1.659616000
1	8.136857000	15.147176000	2.184398000
6	6.309840000	14.107980000	1.733883000
6	5.847557000	15.127011000	0.906694000
1	6.456914000	16.003630000	0.723628000
6	4.580349000	14.977823000	0.315189000
1	4.184120000	15.757608000	-0.327091000
6	3.834249000	13.835689000	0.550965000
1	2.856397000	13.717290000	0.104906000
6	4.354156000	12.821398000	1.408472000
6	2.437418000	11.310082000	0.988225000
1	2.531006000	11.535251000	-0.084060000
1	1.601558000	11.919646000	1.370078000
6	2.055901000	9.811242000	1.125448000
6	0.776618000	9.590089000	0.283116000
1	0.447588000	8.547053000	0.357194000
1	0.955342000	9.814529000	-0.775793000

1	-0.043688000	10.228692000	0.634463000
6	1.758461000	9.448962000	2.599554000
1	2.645963000	9.571767000	3.228324000
1	1.430609000	8.404587000	2.672514000
1	0.956800000	10.082323000	3.000999000
6	3.190428000	8.913647000	0.579497000
1	4.113297000	9.037620000	1.155384000
1	3.408174000	9.154265000	-0.468649000
1	2.897135000	7.857994000	0.630656000
6	9.177594000	13.082441000	3.987615000
1	9.852264000	13.875691000	4.337121000
1	9.576016000	12.691136000	3.049543000
6	9.109132000	11.879261000	4.983543000
6	8.943031000	12.349943000	6.455504000
6	7.892054000	11.856316000	7.241741000
1	7.191766000	11.167592000	6.787143000
6	7.750535000	12.247886000	8.580179000
1	6.929195000	11.853276000	9.171469000
6	8.661466000	13.141271000	9.155303000
1	8.552289000	13.446590000	10.191323000
6	9.722531000	13.633089000	8.382307000
1	10.441749000	14.319624000	8.818957000
6	9.864436000	13.235502000	7.048126000
1	10.707052000	13.608939000	6.473319000
6	10.422656000	11.059064000	4.922234000
6	11.639318000	11.574097000	4.441940000
1	11.701251000	12.588570000	4.063473000
6	12.802852000	10.789500000	4.442106000
1	13.730253000	11.207925000	4.062593000
6	12.771310000	9.478964000	4.927652000
1	13.671801000	8.872834000	4.929774000
6	11.563563000	8.956091000	5.413354000
1	11.525969000	7.940273000	5.795705000
6	10.405446000	9.738798000	5.409061000
1	9.470838000	9.332063000	5.776014000
7	3.639175000	11.812217000	5.599717000
1	4.135881000	11.162908000	4.957204000
7	5.377105000	13.286761000	5.121603000
7	7.824241000	13.643643000	3.684947000
7	5.582677000	12.991878000	2.008215000
7	3.688746000	11.665383000	1.659860000
1	4.107891000	11.035067000	2.375549000
8	8.013340000	11.056653000	4.578766000
6	8.661537000	8.611374000	2.265322000
6	7.786659000	7.541580000	2.842458000
6	7.181301000	7.771276000	4.094486000
6	6.393770000	6.779338000	4.693493000
6	6.195526000	5.552987000	4.047536000
6	6.792441000	5.319386000	2.800025000
6	7.583180000	6.304971000	2.202387000
1	6.639922000	4.371148000	2.294522000
1	5.938839000	6.964386000	5.661687000
1	7.351369000	8.723925000	4.590116000
1	8.029143000	6.117568000	1.230732000
6	10.029901000	8.180597000	1.767262000
35	7.665301000	9.489040000	0.604609000
1	8.703892000	9.474763000	2.930447000
1	10.578547000	9.025366000	1.343450000
1	10.602143000	7.795912000	2.621336000
1	9.968530000	7.391199000	1.012966000
1	5.583000000	4.785317000	4.509451000
